# Disability disclosure in healthcare settings for individuals with developmental disabilities: A qualitative study of patient and caregiver perspectives

**DOI:** 10.1371/journal.pone.0329328

**Published:** 2025-08-07

**Authors:** Ashley Falcon, Andrew Porter, Brady Wallace, Jenna Tatavitto, Gillian Aaronson, Arnina Wiles, Rachel Ryan, Lindsey Rosenbloom

**Affiliations:** School of Nursing and Health Studies, University of Miami, Coral Gables, Florida, United States of America; Father Muller Charitable Institutions, INDIA

## Abstract

**Background:**

People with disabilities experience significant healthcare disparities, including missed opportunities for preventive, inaccessible services, and inadequate communication with providers. These challenges often lead to unmet healthcare needs and poor health outcomes. Disability disclosure is one strategy that may aid in closing this healthcare equity gap, though limited research sheds light on patient and caregiver feelings towards and preferences for disclosure.

**Objective:**

This study assessed comfort with and preferences for disability disclosure within healthcare settings among individuals with developmental disabilities and caregivers of individuals with developmental disabilities.

**Methods:**

An exploratory qualitative research design was employed, utilizing semi-structured interviews with 22 participants (10 patients and 12 caregivers) in South Florida. Data were transcribed and analyzed through thematic analysis to identify key themes related to disability disclosure in healthcare settings.

**Results:**

Five main themes emerged. Two themes centered on the downside of disclosure (harm avoidance and disclosure utility), while two themes illuminated the upside of disclosure (disclosure necessity and reduced stigma). The final theme focused on disclosure preferences.

**Conclusions:**

Comfort with disability disclosure among patients and caregivers was largely motivated by a desire to avoid perceived pitfalls and secure quality healthcare. Findings confirm the persistence of inadequate healthcare delivered to patients with disabilities, and the beneficial role disability disclosure can play in addressing current deficiencies. With support of healthcare system leadership and other salient stakeholder groups, further research can inform development, implementation, and evaluation of disclosure systems that facilitate equitable care delivery and improve health outcomes among patients with developmental disabilities.

## Introduction

People with disabilities (PWD) face significant healthcare barriers, including difficulties in scheduling, facility and service access, and inadequate provider communication [1–4]. Such challenges result in higher levels of unmet healthcare needs. For instance, PWD often receive inadequate preventative care, experience higher misdiagnosis rates, and face failures in reasonable accommodations [5–9]. These deficiencies yield not only greater levels of dissatisfaction with healthcare among PWD but also worse health outcomes, including increased morbidity and mortality [7,8]. With 27% of Americans reporting disabilities, addressing these healthcare inequities is crucial [10].

Incorporating disability status within electronic health records (EHR) could address healthcare inequities by enabling patients to specify needs and encouraging healthcare systems to identify and address disparities. Despite federal mandates, there remains a lack of standardized disability data collection in healthcare [11]; recent efforts to establish a national standard for EHR disability documentation are promising but have faced criticism for lack of inclusivity [7,11,12].

As various methods for collecting disability status are developed, it’s crucial to consider how PWD react to these efforts, particularly given their past experiences of discrimination in healthcare [9,13]. To date, there is limited research on patient and caregiver perspectives. Using data from a cross-sectional survey of patients with and without disabilities across three healthcare settings, Morris et al. [14] found that 95% of respondents felt collecting disability information was important and 77% reported feeling comfortable or very comfortable with healthcare organizations collecting this information. In 2021, focus groups consisting of patients with diverse disabilities that assessed perspectives concerning healthcare accommodations yielded four main challenges to equitable care delivery and actionable recommendations to address them [15]. This study aims to enhance our understanding of the factors influencing patients’ and caregivers’ willingness to share disability-related information by qualitatively exploring their comfort and perceptions of disability disclosure within healthcare settings.

## Methods

### Design

An exploratory qualitative research design using semi-structured interviews was employed. Thematic analysis was the methodological orientation applied to gain a comprehensive understanding of study participants’ perceptions of disability disclosure within healthcare settings [16]. This study was approved by the University of Miami Institutional Review Board (Protocol 20181115). All participants provided written informed consent prior to participation.

### Participants

A purposeful sample was recruited in South Florida from March 26, 2019, to March 5, 2020. Eligible participants were adults over 18, either diagnosed with a developmental disability or caregivers of someone with a developmental disability, who had received care in healthcare settings. Local disability organizations facilitated recruitment by distributing digital flyers. Interested individuals contacted study staff and were screened via telephone or email. Interviews were scheduled for eligible participants.

### Data collection

An experienced, female qualitative researcher (AF) conducted interviews in private locations on a university campus or at participant-chosen sites, ensuring privacy in public settings (e.g., coffee shops). Participants provided demographic information (patient age, sex, disability) and responded to questions from a semi-structured interview guide co-designed with disability advocates. This guide focused on their healthcare experiences, comfort with disability disclosure, and disclosure preferences. The interviewer, who had no prior relationship with participants, audio recorded the sessions and took notes. Interviews averaged 50 minutes, and participants were compensated $50.

### Data analysis

Audio recordings were transcribed verbatim by a third-party company. Data were analyzed using Guest’s thematic analysis approach, which involves transcription, familiarization, systematic coding, theme development, theme review, and interpretation [16]. Three trained coders independently reviewed the transcripts to generate preliminary codes and convened regularly to refine themes and categories using a coding tree. Initial coding was followed by the collaborative identification and refinement of themes through an iterative process of reviewing coded data and revising theme definitions. The coding and thematic development process was recursive, allowing the team to refine codes and themes across the dataset for consistency and depth.

Microsoft Excel was employed to manage the data. Coded excerpts were arranged in a spreadsheet format, with rows representing participant excerpts and columns indicating assigned codes, themes, and categories. This structure enabled efficient filtering, comparison, and refinement of codes across transcripts, supporting the iterative analysis process. No new codes were identified after the 20th interview, with two additional interviews confirming data saturation. Representative quotes that best illustrate the phenomenon of interest were identified.

Analytical credibility was bolstered by a consensus-driven approach where coders engaged in ongoing discussions to reconcile discrepancies and ensure interpretive consistency. Researcher reflexivity was embedded in the team’s analytic process. During discussions, coders actively reflected on their assumptions by considering potential alternative explanations for participant comments, thereby remaining open to perspectives that challenged their initial interpretations. The team’s collaborative approach and regular peer debriefings served as informal credibility checks, helping to surface potential biases and maintain the methodological rigor and trustworthiness of the findings.

## Results

### Participant characteristics

All eligible participants completed one interview (n = 22). Ten participants were adults (aged 18 or older) with one or more developmental disabilities, while 12 participants were adult caregivers of individuals with one or more developmental disabilities. Eleven caregivers were mothers of the patient and 1 was the patient’s sister. Patients, participating directly or through caregiver proxy, were predominantly male (54.5%) and ranged in age from two to 52 (mean = 22.6, SD = 14.3). Autism spectrum disorder was the most reported disability. Patient characteristics can be found in Tables 1 and 2.

**Table 1:** Characteristics of Directly Interviewed Patients (n = 10).

Age (years), mean (SD)	37.4	(10.6)
**Sex**, n (%)		
Male	3	(30.0%)
Female	7	(70.0%)
**Self-reported disability**,* n (%)		
Autism Spectrum Disorder	4	(40.0%)
Cerebral Palsy	2	(20.0%)
Deaf	0	(0.0%)
Down Syndrome	1	(10.0%)
Learning Disability	2	(20.0%)
Spina Bifida	3	(30.0%)
Visual Impairment	0	(0.0%)

*Total percentages for self-reported disability exceed 100% given that some participants self-reported more than one disability; SD = standard deviation.

**Table 2:** Characteristics of Individuals with Developmental Disabilities Represented by Caregivers (n = 12).

Age (years), mean (SD)	16.8	(11.1)
**Sex**, n (%)		
Male	9	(75.0%)
Female	3	(25.0%)
**Reported disability**,* n (%)		
Autism Spectrum Disorder	8	(80.0%)
Cerebral Palsy	0	(0.0%)
Deaf	2	(16.7%)
Down Syndrome	2	(16.7%)
Learning Disability	1	(8.3%)
Spina Bifida	0	(0.0%)
Visual Impairment	3	(25.0%)

*Total percentages for self-reported disability exceed 100% given that some participants self-reported more than one disability; SD = standard deviation.

Five themes emerged from an analysis of the interview data (S1 File). These themes highlight the upsides and downsides of disclosing a patient’s disability status, and preferences regarding disability disclosure in clinical settings. Table 3 lists identified themes, subcategories, and representative quotes.

**Table 3:** Themes, categories, and selected quotes.

Themes	Categories	Quotes
**Perceived Downside of Disclosure**		
**Harm avoidance**	Inferior care for the disabled	“It’s sad that you go certain places and if you have a person with special needs you’re lower down on the totem pole than another person because they may feel that you don’t need it as much as the other person.” (Quote 1)
	Unintended consequences	“...with disability often comes a lot of fear and a lot of anxiety because we’re still a people and not too long ago we were institutionalized because we were different. And so often people are afraid to disclose because if I say too much am I going to make myself too vulnerable, which then is going to create a whole bunch of problems for us. So if we make the cocoon more welcoming and less fear of oh my god baker acting or something bad happening to me then I think it’s a beneficial environment for everybody concerned.” (Quote 2)
**Disclosure utility**	Provider receptivity	“I found that some of them are just like “I’m the doctor, and this is what I’m gonna say, and I’ve gone to school” and that’s it. “ (Quote 3)
	Information disuse	“Everything that they asked me for, any kind of medical documentation I provided. Because I provide that, why aren’t things being done? You ask me for this, I go get it. I just feel like I’ve been doing and doing and doing and doing.” (Quote 4)
	Incapacity to accommodate	“Maybe space out your appointments every hour and don’t make an appointment every half hour. [Because] all of a sudden you have to wait in the waiting room for two hours with two kids that have disabilities, and then a mother who is about to rip off their head because there was no personalization.” (Quote 5)
	Relevance to care	“...I can choose to disclose or not to disclose. Yea because it depends on whether my physical condition is related to that [disability].” (Quote 6)
**Perceived Upside of Disclosure**		
**Disclosure necessity**	Countering provider assumptions	“Sometimes doctors if I go with anyone else they tend to talk to that person or they talk to me slowly...How.... are.... you. Like I cannot comprehend and I’m thinking in my mind that’s a misconception, you think I have a physical and mental disability here but it is only physical.” (Quote 7)
	Filling provider disability knowledge gaps	“So if I see that they’re not asking me something that’s directly relevant and it would help me to get from point a to point b in their understanding and get better medical care, you kinda have the responsibility to try to jump the hurdle.” (Quote 8)
	Sharing personal expertise	“Well I always feel like it’s an obligation of mine. Uh, and I guess I do expect that if I go in with some information to help you, that you are able to help me with you know at least following my lead right? So even if you’re clueless, you could at least like I said, follow my lead or even ask questions to you know. Uhm, because they you know obviously out of respect for them I, don’t, I don’t tell you, you don’t know what you’re doing ever because I’m the one coming to you. So the doctors know their field and respect that always. But what they don’t know is [my son]. And so that’s, I always try to give them like a glimpse of what he is. So that he can treat him the best way possible.” (Quote 9)
	Improved care coordination	“This is a team of doctors taking care of one case. They need to talk to one another and communicate because as the patient, I don’t want to cause any problems and I have because I’ve been like well you’re saying I can’t go home but the doctor that just came in said that I can, so which one is it? It turns into like this whole big mess where I get blamed for like causing all this drama” (Quote 10)
	Securing accommodative assistance	“But if it’s a changing table that just won’t go down at all, then most likely I’m going to have to ask for assistance and then depending on who the doctor is or who the nurse is, that can turn into like a whole show where they’re like oh well let me go get help and let me let me get this guy and that guy.” (Quote 11)
	Provider as gatekeeper	“You know you can’t go anywhere without referrals; that’s kind of where it starts.” (Quote 12)
**Reduced stigma**	Increased acceptance	“It’s become more average, more out there that there’s so many people out there that have special needs that it’s become more acceptable now. Now, thank god, back twenty years, back, it wouldn’t have been like, as easy as it is now, I mean people were, were frowned for you having a child that has special needs. I mean I, my, my boss, asked me why I would do that myself.” (Quote 13)
**Disclosure Preferences and Considerations**		
**Patient-centered**	Patient empowerment	“I personally like [my son] to be able to do as much on his own as possible. So when I’m going to get into the doctor’s office, I try not to answer the questions. I’ll help him, but I want him to be able to do it.” (Quote 14)
	Patient capacity	“...I’m the patient, I’m the one that needs to be answering. Now if there’s something that I don’t understand, okay. That’s where you come in, a new answer, and you speak for it, but not all day long, because that’s how I need to show people that I am capable of talking and moving around, with things, and I don’t have to depend on others to go anywhere with me, being with 24/7 like a caretaker or anything because I go out a lot and I do things all on my own.” (Quote 15)
**Assessment validity**	Self-Identified Disability	“And in fact, [An acquaintance] was talking today about when he got diagnosed with autism, which was quite late in life. He absolutely didn’t think he was disabled...I think saying something like ‘Would you like us to know anything else about you’ or ‘Is anything important else about yourself that you would like us to know’ for example might be [better].” (Quote 16)
	Stigma Avoidance	“...If you ask the more generic question, “do you have a disability?” I think you’d get me to answer yes but you’d get that person in that wheelchair who says no I don’t, I only have issues with my knee and I can’t go out at night because I really can’t see to drive at night, but I’m not blind, because there’s still stigma associated with disability. “ (Quote 17)
**Assessment utility**	Process flow	“No, I think obviously first you should fill out a form, and then follow up with verbally speaking about what you answered yes to or no to.” (Quote 18)
	Functional specificity	“Instead of asking, does he have a disability? I want them to ask specifically. Like behaviors, do thorough evaluations, and take their time, actually reading it, and the questions that you did answer yes for, asking more about them, which none of them do that.” (Quote 19)

### The downside of disclosure

While most participants were generally comfortable with disclosing disability status, some expressed concerns about potential harm and questioned the utility of such disclosures.

#### Harm avoidance.

Several participants expressed concerns that disclosing a disability might lead to the patient receiving inferior healthcare due to providers deprioritizing their needs. One caregiver described a sense that her child was viewed as less deserving of care simply because of their disability (see Table 3, Quote 1). Moreover, some participants feared that disclosure could lead to unintended negative consequences. In one instance, a patient shared concern about certain disclosures leading to a potential loss of autonomy (see Table 3, Quote 2).

#### Disclosure utility.

A key concern was if disclosed information would be used, with several participants skeptical about providers’ receptiveness to shared information. One patient recounted disregard for patient input, describing encounters where providers dismissed their contributions based solely on professional authority (see Table 3, Quote 3). Participants also expressed frustration that solicited information they shared with providers was often unused. A caregiver recounted, repeatedly submitting requested documentation, only to see no resulting action or follow-through from providers, leading to a sense of exhaustion and futility (see Table 3, Quote 4). Likewise, participants noted that some providers lacked policies or protocols to accommodate disclosed disabilities. One caregiver observed that standard scheduling practices failed to account for the needs of patients with disabilities, resulting in heightened stress for families (see Table 3, Quote 5).Alternatively, some participants felt that disclosure was unnecessary unless directly relevant to their reasons for seeing a provider. For instance, a patient shared that they based their decision to disclose on whether their disability had a direct bearing on the care being sought, viewing disclosure as situational rather than routine (see Table 3, Quote 6)

### The upside of disclosure

Despite reservations, participants highlighted situations where disclosing their disability was necessary for improving healthcare experiences and reducing disability-specific stigma.

#### Disclosure necessity.

The majority of participants discussed the need to disclose disability status to correct any misconceptions providers might have. One patient shared that disclosure helped clarify the nature of their disability, especially when providers made inaccurate assumptions about their cognitive abilities based solely on physical appearance (see Table 3, Quote 7).Several participants also highlighted the importance of disclosure in addressing providers’ often limited knowledge of specific disabilities, which could impair the quality of care they provide. One participant demonstrated this point by describing moments when disclosure became necessary to fill knowledge gaps and ensure the provider had sufficient context to deliver appropriate care (see Table 3, Quote 8). Beyond ensuring that providers had necessary disability-specific knowledge, many participants also felt it was their responsibility to educate providers about the patient’s specific needs, or what the authors refer to as the patients’ and caregivers’ ‘personal expertise.’ This was of particular importance for participants who acknowledged the significant variability in accommodative needs even among individuals with the same disability. A caregiver explained that while she respected providers’ medical expertise, she saw it as her role to offer insight into her child’s unique profile, enabling more individualized and effective care (see Table 3, Quote 9). Building on this, some participants viewed disclosure as essential for effective care coordination among multiple providers. One patient emphasized that when providers lacked shared understanding of a patient’s disability, it could lead to conflicting recommendations and confusion, ultimately placing the burden of reconciliation on the patient (see Table 3, Quote 10). In addition to contexts in which participants sought to use the sharing of information as a means of enhancing their healthcare experience, necessity also stemmed from instances where disclosure was unavoidable. For instance, some participants discussed how they had to disclose their status, given the need to secure appropriate accommodation. One patient highlighted this potential need when describing situations where inaccessible equipment required them to explain their disability to obtain basic assistance (see Table 3, Quote 11). Likewise, the role of the provider as a gatekeeper to other services was also identified by participants as a circumstance in which disclosure was inescapable. One participant explained that disclosure was a prerequisite for obtaining necessary referrals, making it an unavoidable part of navigating the healthcare system (see Table 3, Quote 12).

#### Reduced stigma.

Participants with more long-term experience accessing healthcare often noted increased comfort in disclosing disabilities, although not all shared this sentiment. One caregiver reflected on the cultural shift over time, contrasting current norms of acceptance with earlier experiences of stigma and judgment surrounding disability disclosure (see Table 3, Quote 13).

### Disclosure preferences

Overall, participants expressed similar preferences for how to disclose disability status. These preferences centered on ensuring a patient-centered approach and ensuring that assessments were valid and utilitarian.

#### Patient-centered.

Patients and caregivers alike stressed the importance of directly involving the patient in the disclosure process, viewing it as empowering and critical to maximize autonomy. One caregiver shared her intentional effort to step back during medical encounters, allowing her son to take the lead in communicating his needs and building self-advocacy skills (see Table 3, Quote 14). This sentiment was also shared by patients, who expanded on this perspective to reinforce the idea that engaging in disclosure dialogues would provide patients with opportunities to demonstrate their capacity, and challenge potentially harmful provider-held assumptions that could diminish care. One patient made this case by emphasizing that answering questions independently was not only a matter of accuracy, but a way to affirm competence and reduce unnecessary reliance on others (see Table 3, Quote 15).

#### Assessment validity.

Several participants emphasized the need for disclosure assessments with suitable wording that accurately identifies individuals with disabilities, noting that terms like ‘disability’ may not resonate with those who do not self-identify as disabled. One caregiver reinforced this point with an anecdote about a friend’s late autism diagnosis and proposed using more open-ended questions that invite patients to share relevant information without relying on potentially alienating labels (see Table 3, Quote 16).Some participants linked the need for cautious wording in disclosure assessments to ongoing societal stigma around disability, suggesting that stigma could deter individuals from identifying with the disability label due to its negative impact on healthcare delivery. One patient explained that despite clear impairments, some individuals may reject the term ‘disability’ to avoid negative stereotypes, underscoring the value of more inclusive and nuanced language (see Table 3, Quote 17).

#### Assessment utility.

Most participants highlighted the need for assessments that can practically and effectively inform care delivery. Participants repeatedly acknowledged the need for a structured procedure when disclosing disability status. Specifically, they favored a brief, initial assessment followed by a more in-depth follow-up. One caregiver suggested starting with a form-based approach, then supplementing it with a verbal discussion to clarify responses and ensure mutual understanding (see Table 3, Quote 18). Participants stressed that disclosure assessment questions should have utilitarian specificity that supports accurately identifying accommodative needs. They found broader questions about disability status less useful due to the wide variability in needs; focused questions were seen as more effective in guiding care delivery. One participant described their preference for targeted inquiries, which were seen as too vague to drive meaningful care adjustments (see Table 3, Quote 19).

## Discussion

This study explored how patients and caregivers perceive disclosing disability status within healthcare settings. All participants reported comfort with disclosing their or their loved one’s disability status, aligning with previous findings that patients, both with and without disabilities, generally support being asked about disability in healthcare contexts [14,17]. Participants offered nuanced views on disclosing disability, highlighting both upsides and downsides. The primary disclosure concern was the potential for harm, such as receiving inferior care or facing discrimination, which is a well-documented issue in the existing literature [14]. In a study by Ali et al. [9], half of the participants with mild and moderate intellectual disabilities and their carers reported experiencing discrimination and negative attitudes from healthcare providers. Similarly, Bacherini et al. [18] found that a significant number of healthcare providers expressed frustration, pity, and dissatisfaction towards people with disabilities, reflecting a lack of confidence in their care. A national survey by Iezzoni et al. [19] found that most U.S. physicians perceive those with significant disabilities as having a lower quality of life, with few acknowledging unfair treatment within the healthcare system, while VanPuymbrouck et al. [20] identified implicit biases among healthcare providers, termed aversive ableism, despite outwardly minimal prejudice. Participants’ anecdotes of their own healthcare experiences mirrored the existing literature, emphasizing an overall desire to disclose only when disability-specific information was relevant to the care being provided. The existing foundation of knowledge may also help to understand participants’ skepticism about whether some providers would be receptive to receiving disclosed information. Nonetheless, available research from clinicians and clinical staff demonstrates that there is support for collecting disability information within healthcare systems [21,22]. Likewise, existing efforts to collect disability information within healthcare settings show promise that complements negative narratives [17,23–25].

Participants questioned not only provider receptivity to disclosure but also whether providers had the intention or capacity to effectively use shared information to tailor care. While most participants primarily attributed disuse to a non-specific perception that providers do not read disclosed information, there are several possible explanations for disuse. During focus groups with Australian case workers and allied health providers who work with adults with severe communication impairments, Hemsley et al. [22] identified concerns about whether providers would invest the additional time needed to use a personally controlled EHR. The inconsistent and unstandardized recording of disability-specific information within EHRs has created substantial barriers to effectively using this data across multiple healthcare settings [21,26]. To address this, integrating disability disclosure into EHR systems through structured intake forms or standardized fields can facilitate consistent documentation and accessibility of this information to relevant care providers, potentially enhancing its utility in clinical decision-making. Moreover, embedding prompts and decision support tools within EHRs can encourage providers to act on disclosed information and offer appropriate accommodations [27]. Provider knowledge gaps and misconceptions about disabilities contribute to the underuse of disability information. Pelleboer-Gunnick et al. [28] found that health professionals often misjudge the capabilities of those with intellectual disabilities, a finding echoed by Bacherini et al. [18] who noted a persistent lack of understanding among physicians regarding intellectual disabilities and associated healthcare needs. Moreover, studies indicate that clinical staff frequently lack knowledge about local services and resources needed for appropriate referrals and accommodations [9,21].

Despite potential drawbacks, participants recognized the benefits of disability disclosure, primarily as a proactive tool to enhance healthcare experiences by sharing crucial information. Patients and caregivers alike actively corrected providers’ misconceptions and filled knowledge gaps, using their ‘personal expertise’ to facilitate better care coordination and secure necessary accommodations. Evans [29] found similar practical benefits of disclosure in non-healthcare settings, although other identified forms of disclosure were less evident in this study. The variable success of disclosure in achieving accommodations aligns with findings from recent studies on its implementation in healthcare settings [24,25].

Participants preferred patient-centered disclosure methods that enhance the validity and utility of assessments, supporting the accurate collection of disability information without stigmatization. They recommended avoiding the term ‘disability’ to mitigate stigma, a point supported by Pinto et al. [30], who found that direct questions about disabilities can be problematic. This highlights the critical role of terminology in shaping disclosure behavior and data accuracy, especially for those most resistant to “disability” as a label. Employing functional descriptors may improve willingness to disclose and the quality of data collected [31]. Morris further confirmed the presence of classification discrepancies when using general versus specific disability assessment questions [14]. As for which clinical staff should collect disclosure information, participants in this study did not express any variability in comfort with disclosure based on which clinician collected disability information, though a previous survey of patients with and without disabilities showed lower levels of comfort with front desk staff assuming this role [14].

A majority of participants endorsed following up brief, initial assessments with specific questions that could more practically inform care delivery by linking needs to available accommodations, though participants were unable to provide specific suggestions. When assessing patient perceptions of a series of six disability questions from the American Community Survey (ACS) endorsed by the U.S. Health and Human Services for use in healthcare settings, Morris et al. [14] reported that 28% of patients believed that the questions did not identify all disabilities, with developmental disabilities among those most frequently omitted. A more recent study assessing preferences for healthcare accommodation among patients with diverse disabilities shed light on relevant considerations that could inform the development of data collection tools, including environmental, communication, and administrative task accommodations [15]. Halkides et al. [23] showcased one healthcare system’s efforts to functionally assess disability status via EHRs by collecting disability classifications that are more expansive than those identified by the ACS questions and linking them with available accommodation options.

Findings should be viewed with consideration of the study’s limitations. The purposive sample from South Florida, while achieving data saturation, may limit external validity and not represent perspectives of patients with unrepresented developmental disabilities or those outside the region. Despite focusing solely on patients with developmental disabilities and their caregivers, the results align with studies involving various patient groups. Nonetheless, the insights gained provide valuable information on patient and caregiver preferences and comfort with disability disclosure.

The predominately offensive nature of disability disclosure identified among study participants speaks to the crucial need to address deficiencies throughout healthcare systems, from how providers and clinical staff are currently prepared to care for patients to infrastructure and policy climates that are more likely to exacerbate rather than prevent health disparities for patients with disabilities. Working diligently to collect disability information presents an opportunity to address these deficiencies by providing a systematic means of recording and addressing the needs of patients with disabilities. It is equally important that these efforts meaningfully involve patients with disabilities and incorporate their input along with that of the many other stakeholder groups needed to produce effective systems. For instance, in this study, while there was a significant overlap of experiences and preferences between patients and caregivers, the unique perspective of the caregiver as an advocate for and defender of patient autonomy was a salient observation that reinforced patients’ anecdotes about poor healthcare experiences.

## Conclusion

Our study enhances the growing body of literature advocating for a more inclusive and responsive healthcare system for individuals with disabilities. Findings highlight the complexity of disability disclosure within healthcare settings and the need for more nuanced approaches that prioritize patient autonomy and tailored communication strategies. This research underscores the importance of aligning disclosure practices with patient preferences, thus fostering a supportive and effective environment for patients and caregivers. Based on the limited specificity of logistical input about disability disclosure gleaned from participants, there is a clear need for further research to explore patient preferences regarding the development and implementation considerations (e.g., when, where, how, how often, and by whom) for collecting disability information. This should include providing patients and caregivers with proposed examples or models to critique and identifying any nuances or divergence of preferences among various groups of patients with accommodative needs across different healthcare settings. Moreover, implemented efforts should be continuously evaluated for their effectiveness in informing care delivery and meeting patients’ accommodative needs. Expanding this research to include more geographically diverse populations and to assess the long-term outcomes of disclosure practices will help determine how they address existing health disparities and their implications for healthcare costs. Achieving this is only possible with the sustained support of healthcare system leadership, which includes integrating disability data collection into EHRs, building out referral networks and accommodation offerings, and providing training and accountability for all clinical staff. An effective disability disclosure system would then provide ongoing quality assurance and improvement data to inform healthcare system operations, ultimately paving the way for improved healthcare experiences and outcomes for this underserved population.

## Supporting information

S1 File Interview Transcripts. (ZIP)
