# Supplementary material for: Disability disclosure in healthcare settings for individuals with developmental disabilities: A qualitative study of patient and caregiver perspectives
Source: PLoS One. 2025 Aug 7;20(8):e0329328. doi: 10.1371/journal.pone.0329328 (PMC12331114; doi:10.1371/journal.pone.0329328)
Supplement: S1 File — (ZIP) [file pone.0329328.s001.zip › Transcripts/2019.03.26 Interview 01 Transcript.docx]

I: Interviewer F: Female key informant

**I: I’m actually gonna see if I can record it on Zoom as well since I know they have that option…let’s see here…Okay! So it’s actually recording already–perfectly. So…um… I’m Ashley (laughs) um..and..uh..just really looking to get some input from you about the healthcare experiences you’ve had with your daughter thus far…um…so just I guess just a general opening question is tell me about a typical day with your daughter**

F: Um..so my daughter was born with..um..bilateral hearing loss so she was identified at birth…um…she’s been aided since she was two years old so..um..and she’s developmentally delayed in regards to like (inaudible) or things like that because of the speech and the hearing…um…where she just turned four and she’s in the “why” stage and it usually happens I think at two (enthusiastically) or something much younger. Or three. So it’s pretty interesting, she has such a large vocabulary now, to do the “why” stage it’s more entertaining I should say for myself

**I: (laughs) Okay**

F: So she’s…uh…I think almost a typical four year old…uh…there are some things that we have to adjust to our life and to our everyday life…uh…I have to remember that in the morning…um… that she really can’t hear me unless she has her implant on…um…and the extra added background noise on anything…um…can be a little bit difficult. She’s still learning, she was just implanted on one ear in August…um…so if I’m in the other room, she’ll be walking through the house, “Mom! Where are you? Where are you?”…and I’m like “I’m over here I’m in like”….and until she like gets the direction of the sound, it’s a little struggle

**I: Mhm…okay, okay…uh…I’m just taking notes if that’s okay with you just so I can make sure I’m not missing anything.**

F: Of course

**I: Alright so..um…so I don’t know necessarily the best way to do this but whether to think about individual healthcare settings or think about it in total…so I guess if you had to think about your satisfaction level with the healthcare she has received, what ranking would you give if zero was completely negative, and 10 was completely positive?**

F: Um right now in her life…right now…I can say…10…100%..I’m so positive and very happy with the system, but there were times when I was absolutely at a zero…or a one

**I: Mhhm…so that’s good to hear the 10 right now…so tell me…um…what makes you want to rank it so high at a 10–what are those positive healthcare experiences if you could give me specific examples to kind of help me kind of understand that?**

F: Of course. We…um….we…um…my daughter was born in New Hampshire, so we relocated to Florida when she was 13 months old–2016…um…we relocated to south of Sarasota, about two and a half hours west of Orlando…um…we had a great team in New England, we went to [Hospital]…um…they understood her, they had a program, it was great– though we drove three hours one way to go to the program…um…because we lived in a remote area. We moved to Florida…um…and trying to get set up with the services was difficult…um…you know…I’d reach out to other families and go “Hey, Who do you go to”, “Who do you go to”, and we went to one ENT, we went into the office, and he said something we didn’t like and we walked out (laughs)…we finished the appointment obviously, and on the way home my husband was like “We’re not going back”, and I was like “Oh, wow. This is good!”…um…so through that tradition we had good people along the way, but we didn’t have a cohesive team for all of the things. So currently why I ranked it a 10, just recently moved in October to the panhandle, and we drive five and a half hours to drive to Orlando to go to the (inaudible)…and we love her audiologist, we love her ENT…we love her speech therapist, her speech therapist is (inaudible) the computer, her ENT will call me on her cellphone, and text me for things. We had an issue…my daughter, a month ago maybe, had an ear infection and we went to the pediatric hospital, they sent us home. I sent him an email, and he called me within five minutes and was like “No, go back, I’m calling them” and through this process I have another child who is 16 months old, he passed the newborn hearing screening…um… and we had to do a follow-up…we did a follow-up, they had a satellite office in Pensacola which is an hour and twenty minutes away, and they said that they suggested ear tubes because he’s had double ear infections, and I said “Okay”. I sent to her my daughter’s ENT, completely different insurances, never had seen my son, he says “Absolutely, your daughter is getting implanted on the fourth, we’ll do his surgery on the fifth.”

**I: Mhm.**

F: What more could I ask for?

**I: Great, okay…um…so…so…then in that sense they became aware of-of your daughter’s um-um-uh hearing concerns by you contacting and seeking them out rather than them kind of engaging, but I would say, you know, when you make that initial contact, what type of questions, or what was the interaction like for them to get whatever needed information that -uhh- that you would say they needed in order to give proper care and any accommodations for her care?**

F: Um, I’m a crazy mom so…um…I have a copy–I have a three ring binder – I have a copy of every audio, every MRI, whatever it was. So the first meeting on appointment with any therapist, with any doctor, I always bring a copy…um…and usually I have it printed out for them, a copy to keep in their files. So, um, but he actually read– her ENT ACTUALLY read…you know I know they’re busy, I know healthcare is crazy–I get it. They deserve a million dollars for what they’ve given to my daughter, and what they’ve done to so many children–you know or opportunities, like he’s talented, like,…um…that’s why we drive five and a half hours. People–there’s ENT’s near us…people are like “Oh my gosh. Why do you drive?”…I say “Because”….and the other thing is if I’m talking to the speech therapist, she’s already talked to the audiologist, the ENT, they all agree, so they’re all talking together, if they see themselves on the floors, the hallway, at lunchtime, or whatever it is, they already know what’s happening. If I think there is a drop in the hearing, and she’s done a hearing (inaudible), and sometimes we have her hearing tested at nine o’clock, two o’clock, the next one is a speech therapy session, by the time I walk into that 2 o’clock appointment, her speech therapist has already looked at the audiogram, the audiologist has already called her, and told her there was a drop in hearing.

**I: Mhm…Mhm…and so you said…yeah I like this idea of a cohesive team, is that everyone in the same place working together, or they just happen to mesh well but don’t work together? How is that cohesive team factor in?**

F: Um…it’s interesting because, um, when my daughter was first diagnosed, um, we had different people at different places–our audiologist was an hour away, and then we went–we started to go to [Eye & Ear], and then we ended up going to [Boston Hospital], but at [Boston Hospital] the audiologist, the speech therapist, and the ENT all talked together because it was–yes it was in network or in the same hospital, but they worked with the same kids, like, every kid they saw either saw this audiologist or this audiologist. So when I went down… when we came to Florida, we did [Florida Hospital], but [Florida Hospital] was a pediatric hospital–they didn’t have anybody in the area for an ENT, so they outsourced an ENT, and that’s why…and I’m fine with that–whatever…but the problem was that there was that disconnect…so then when we finally went to (inaudible), and the ENT has showed me that, and he’s told me this, he says “we’re trying to model [Boston Hospital]”–it’s because it works so well. Like my daughter thought she’d need orthotics due to early steps, so they made us do all of this stuff, we bought the orthotics, blah blah blah, and I was like “No, I don’t think she really needs it, I need an orthopedic referral” I went onto (hospital name) website, her patient portal had everything, the next time we went to the ENT he was like “Why was she seeing an orthotics, why didn’t she see the orthopedic specialist?”, and I was like “It was nothing to do with her hearing loss…nothing.” But it was so nice that they all got together and they all had that connection. I don’t know, some companies…you know I work for a company…some companies the communication’s great, sometimes it stinks, but I think it’s partially–number one its the company and the atmosphere that they grow but it’s also the person…and I think the three people that helped me deal with (daughter’s name) are wonderful. Her primary care physician is not related to that hospital at all…um…and they’re actually connected to the pediatric hospital that we went to and had a bad experience, but she understands, so I always tell her please do a referral to the (hospital name) in Orlando, and she understands and she trusts them to do that.

**I: Mhm…So I know…I wanted to ask what accommodations if any…um…the healthcare providers make for making the experience positive for your daughter…I know–I’m thinking that was directed towards somebody who plays a more active role, maybe a little bit older, an adult, as far as communication or anything like that. So, I mean given that she’s younger maybe that doesn’t apply as much, but any thoughts about accommodations they have for, you know, talking to her about what’s happening to her, or, you know, what treatment she’s going to be getting, or asking her how she’s doing… anything like that that you might share?**

F: Um…Her ENT…like…we go in and he treats her like a four year old–he doesn’t treat her like a 10 year old, an eight year old, or whatever. She’ll go in and she’ll play with the things that go in her ears, or she’ll sit on his lap while he’s looking in her ear…like those type of things…they have that connection–she actually loves to go see him. Like we’ll pull off the highway and we’ll see the hospital, and she’ll say “Dr. Richard. Dr. Richard. Dr. Richard.” And for her to have that comfort…um…and to trust in him at four years old…after he’s not just checked her over, he’s prodded and poked her ears…like it’s not just a check-up, physical, move on. The audiologist, when they’re putting implant or changing the hearing aids–I don’t know what it feels like, I’m sure part of it is discomfort, trying to get your brain to adjust, you know, but she literally trusts them, she trusts them probably as much as I do…and I know kids can be trusting, but for them to understand and go down to her level to talk to her about “Oh, look at this, and look at this”, and we had that discussion to add the second ear, you know, me and my husband were like “Okay yeah”, and I said to my daughter, because she won’t wear her hearing aide, after the cochlear implant, she wouldn’t wear it, and I said… um…”Would you like another ear” like the one that she has, and she was like “Yeah!”, and I was like “Yeah…I don’t really know”, and then like a couple days later she was like “Dr. (inaudible) is gonna get me another ear”, and I was like for her to be able to put that stuff into confidence and connection–she really gets that it helps her.

**I: Mhmmm…Okay, great. So, I wanna go back–you had mentioned that you had gone to one location and they had said something, and you didn’t like it, and you didn’t return…so I guess that would be a nice segway into, you know, what made you rank earlier times as lower, so if you, so if you mind just starting with sharing that story, do you mind sharing what was said in particular?**

F: Oh yea (laughs). It was interesting because it was the first ENT appointment when we moved from Massachusetts, you know, from New England, and the ENT–we walked in, and I tried to do my research because I don’t wanna waste their time. I need - we need somebody who sees a deaf and hard of hearing child, or a child with hearing aids at least once or twice a week. Some ENTs…a lot of their caseload’s that, and sometime they see one once a month…um… but my daughter also has enlarged vestibular aqueducts…um…so…um…it’s connected to the hearing loss but it also can cause balance issues…sometimes she’s like a drunken four year old but not really drunk…um…so…we walked into the office, it was an hour and a half away, we walked into the office, we get in there, and the doctor’s like “Hey what are you here for?”, and I’m like “We’re trying to establish service.” I gave him my report, and he says “Oh” and I explained to him about the EVA and he’s like “Okay”, um “we just came from [Boston Hospital], we moved um”.. “oh that’s a phenomenal hospital”, and I was like “Yeah”, but they were waiting for her hearing to drop, um she qualified for a cochlear implant, but then she disqualified herself because of fluctuating hearing loss…uh…and we’re just waiting for it to drop again. And the first thing out of his mouth was “Oh my gosh…[Boston Hospital] is a phenomenal hospital…blah blah blah” and then the next thing out of his mouth was “I would never implant a child (inaudible).” So I’m like “You just said this hospital”, like we saw the top surgeon that teaches at Harvard, and she specializes in EVA, so and like you just said “they’re a phenomenal hospital, like Oh my God”, and he didn’t even go into an example of why he wouldn’t, it was just like (claps), and then end of conversation. And I was just like–and it’s okay for people…and some parents don’t want that for their child and that’s okay, but we had told him in the conversation that’s what we’re waiting for, like, this is where we think is the best thing for our family and for my daughter, and he as just like, shut off, black and white, you know, the TV off, done, finished. And I was like, I don’t want a provider like that regardless of anything, I want you to tell me why, so then I can make the sound decision myself.

**I: Absolutely. So what other–yeah–in thinking about that lower ranking from previous times, what other things kind of come to mind that made it more of a negative healthcare experience from your perspective or maybe something your daughter told you?**

F: Yeah, um, I–when we first found out she had hearing loss, we went to [New Hampshire Hospital], in New Hampshire–I had her on a Friday, they waited a month for the appointment, whatever. We didn’t tell anybody, I had no clue kids had hearing aids, I was like whatever, so we go to the appointment, you know I’m holding her, I’m a first time mom, my husband has two previous children, and I’m like “(inaudible) Whatever”, you know, and we get in, and we’re going over the results from the audiologist – this was the head audiologist at [New Hampshire Hospital], and we’re going over the results - and she’s like…and I was like “what does this mean?” and she’s like “she needs hearing aids”, and all of the sudden I started to downpour tears, like I don’t even know what she said after that. So I remember her giving me a piece of paper, and she’s telling me she needs hearing aids, and it was like $6700. I signed the paper and we walked out, and I was just like…so I get into the car with my husband and I’m like, “What? Like no nothing?…like hearing aids and that’s it and they just expected us to go on with our day?” My brother has cerebral palsy, so I sort of get it, but I’m like…I felt like the wind was knocked out of me…and I was just like “Okay”. We ended up not going back there–we went to another audiologist…um…who fitted her…um…but what I found…um…is some audiologists–and I don’t know because I’m not an audiologist, but and maybe this is every profession so I shouldn’t partake it just to that…um I found that they.. it’s better for my piece of mind, I guess in routine, for them to be able to find out if they do children and adults–I think for my daughter and my situation I need them to do deafs with children..um… because they’ll say “Oh yes, I can do it”, but it’s just a different approach and it’s a different thing, um… Can you hold on just one second?

**I: Sure, sure.**

F: ….and uh oh…the other negative…um…I was in a hospital room, and I just delivered her and I didn’t know they do a new born hearing screening, because I’m like whatever, I don’t know, so I overhear the nurse saying that she didn’t pass, and they use the word “referred”, which I totally hate that word by the way, like to my dying day I’ll hate that word…um…so…the next time she came in she was like “We’re gonna do it again”, and I said “Ooh, can I go with you, I’d like to see what it is”, you know–I was just curious, you know, so she says - the nurse was like “If you’re very quiet.” I said “okay I’ll be quiet.” What was I going to do, like I’m curious. So I go into like the room that they’re in there, and they have my daughter in that little, you know, bassinet thingy–whatever you call it, and I was standing there in a hospital gown, and umm she’s helping another girl (video call loses audio)

**I: (laughs) No problem, there you are.**

F: So we’re there, and I’m standing there and, you know, literally had my daughter (inaudible) and she’s training another nurse how to do the test, and she’s like umm.. hold on one second I’m sorry.

**I: No problem.**

F: So umm she – so I’m standing there and they’re doing the test and she says to the other nurse, she says, “This baby is not gonna pass.” I was like “What?”

**I: So not even speaking to you**

F: Right, and I was standing right there I’m like “what…I’ve never failed anything in my life, what the hell’s my kid, this baby..like not even 72 hours old, what did she not pass?. So then I get the I get the – and I was like “What does this mean?”, and then she hands me the pamphlet like this, and she’s like “here…all of the information you can get from here”, and I was like “What?” but I was like “eh whatever, like maybe - I don’t know”, and then I went, you know, so then they said “They’ll call you on Monday”–it was Saturday–“They’ll call you on Monday to make an appointment”, but she made me feel like it wasn’t a big deal, “So I was like oh, whatever” my husband was like “What’s the matter?”, I was like “yeah, she didn’t pass, but they’ll call us on Monday for another appointment–we’ll have to go get another appointment..whatever, not a big deal”. And then when we find out Oh my God, and then you find out insurance doesn’t pay for hearing aids–that’s a whole other mess, Holy Jesus!

**I: So would you say at any time…um…your daughter having these umm health considerations have negatively impacted how she’s been treated that you can - any examples that come to mind in regards to that? Whether it be by the healthcare provider, or really anyone, it could be front office or other medical staff as well.**

F: Yeah…um–um–yeah…this is interesting. So about a month ago, my daughter was slotted to have her second cochlear implant surgery on the 14th of March, um, about a month ago maybe, maybe a little less, um… she - we have a lady who takes her to school, and picks her up and drops her to daycare… and, um…they’re like “Oh, her ear’s red”, the one that’s implanted. She never - she’s had it since July – never complained like she (inaudible). So the teacher was like “Oh, I sent her to the nurse, the nurse said it’s a little red, it’s not a big deal” Is this nurse even an RN? Like it’s her implanted side, like!, anyway, the lady who picks her up picked her up from school was like “I don’t think her ear is right.” So she calls me (inaudible) and I’m like “What side is it?”, they’re like “It’s the implanted side”, and I’m like “Okay, I have to make this decision, is it one lady overreacting, or is it…I’m gonna go. I was like “I gotta go, I’m nervous.” I get home and her ear is sticking out and it’s all red in the back. And I’m like, number one I’m like, the school nurse is an idiot, like– well I shouldn’t say idiot, but I’m like, “Are you kidding me?”. And my daughter was complaining her ear hurt. So I call my husband, we both (inaudible) I’m a half an hour away from work our house, he’s like an hour and fifteen, so he calls me and he’s like “Okay, we’re going”. So we drive an hour and 20 minutes to the pediatric hospital because that was like…even though her ENT said go to the closest, I said “no, I’m going to the pediatric”, because maybe there is a pediatric ENT audiologist. And again, we get there, nobody - it’s a Friday night Friday afternoon- there’s nobody in the emergency room, we walk right in, “what are you here for?”. Her ear is red, she’s complaining, blah blah blah. We walk in, they check you into the back, you know, in the emergency room like curtain area, whatever. And the nurse goes “what’s the matter?” I say her ear is sticking out, it’s red, and she has an implant. “Oh she has an implant?” I say “Yes”. She says “well did she get it done at (name of hospital)”, I said, “No we go to Orlando to get it done”–“Oh (condescending tone)”. And I was like “What the hell does it matter like she’s three, just help her, you’re a nurse!”. She checks her ear, she’s like “Oh yeah, she has an ear infection”. They discharged us, and she said “If it gets any worse, you have 12 hrs to call the wards in Orlando (inaudible).” So then, that’s when I emailed her ENT, he wrote back, we were getting off the exit to go to the house and I just didn’t see it, and he says “No. I’m calling the hospital back, I’m writing a letter, and you’re gonna write a letter, and you’re sending her back there.” (inaudible) Do you know we waited 4 hours in the emergency room before she was seen again? And they didn’t know what to do, they were like “I don’t know–we’ll have to wait for your doctor”–blah, blah, blah. I’m like “I don’t get it–it’s not like your first year med students–like you’re a doctor!”

**I: Mhm, so that was the ER? Okay, wow.**

F: Yeah! (laughs) I’m tellin’ ya.

**I: And so…and you’ve mentioned in the past a couple of times that when someone wasn’t giving you the quality of healthcare that you felt was appropriate you’ve kind of just found someone better, you’ve taken strides to seek out the right folks–is in those instances like in the ER or what not, are there any other ways that you’ve responded to those negative experiences–you know, directly confronting or having any communications with them to identify with them that you’re not getting what you need?**

F: Um…the nurse–I asked her..,but she was like…not approachable–like she was like I don’t wanna say “shut down”, but she was like over and done with it. You know, thinking like it wasn’t a big deal. I don’t know, from now what I understand, the (inaudible) at [hospital] had a clash because the (inaudible) where I went to at the clinic was actually in the same location, and something happened, and then now they moved to a new location–regardless, I don’t know–um. But usually before I– I don’t wanna say “write somebody off”, but before that happens like I ask, because I wanna educate myself, this is a lifelong thing, this is what my daughter has. You know, I’ve been very fortunate…I’ve joined a lot of advocacy groups, I’m on a lot of boards, and that’s just me as a person, but I wanna help educated other parents–I wanna help other children–like I– it’s part of what I need to do, but I found that some of them are just like “I’m the doctor, and this is what I’m gonna say, and I’ve gone to school” and blah blah blah and that’s it. And I’m like “It doesn’t have to be that way”. Yes, you might medically know what needs to be done, but you also need to understand maybe your approach or your way of thinking is not the right fit for that family. You know, when we were in [Hospital], and we went to Fort Myers, and I was seeking a new speech therapist, it’s a specialized therapy, and it’s very very hard all over the country to get them, um and I tried to get it, and we went in, and it’s focusing on learn to listen, so it’s not on sign language or anything like that, but we do do sign language because if its not working or (inaudible). Umm but I tell them upfront, “I know it’s against that policy or protocol, but just to let you know, we do do that, you know–I don’t think it will hinder her”, or whatever. They have their own opinions, some of them, and some of them are like, “It’s your choice, it’s your family”. And I remember one in [Hospital], the first time we went to go meet her, and it was the last time, it was close to Halloween, and she was dressed as a witch, and my daughter was like two maybe, and she had gone to this type of therapy since she was three months old, so she got it–like she gets it–and she was so stand-ofish towards my daughter, and she’s like “You’re doing that wrong, you’re doing that wrong, and you’re doing that wrong.” And I was like “I’ve been doing this for two years, like two years I’ve been doing this type of therapy. I’m not an expert by any means, but I’ve been doing this since she was three months old. Like I sort of get it but your approach, if I’m wrong, tell me I’m wrong but don’t be so aggressive about it”. And that’s, that’s where I find that, you know, I’m an expert in my field, and you’re an expert in your field. I find that sometimes we get too cocky in any type of profession, and like hey but you have to understand is that there are so many things that you know, whether it’s my field or your field or somebody else’s field, you know, when we go home, she still has hearing loss, it doesn’t snap and go away. You know, when you go home you don’t take work–I try not to take work with me, but this is part of my everyday life, this is what she is–and I adjust everything of my life to her.

**I: Mhm, right. So would you say that you’re–you’re comfortable with sharing about your daughter’s condition with healthcare providers–um in general–there’s no–or do you have reservations about it at all like concerns moreso because of how you think that they might treat her or anything like that?**

F: No I think I’m pretty good. In the beginning I was sort of wishy washy because I didn’t know a lot of stuff but I’ve really educated myself; I’m not an expert by any means umm but I’m pretty comfortable. My husband is like wishy washy— I wish he would get off work, I joke but I really don’t joke, I’m like if something ever happens to me and I can’t talk or I’m dead we need to grab her binder— Like if the house is burning down, with everything else, you need to grab the binder. He’s like what and I was like her whole life is in that binder. Like there’s results, there’s evaluations, there are tests, like you know? He’s like, oh okay no problem. I’m like NO, you don’t understand, it’s really important.

**I: Mhm. So and this might maybe you want to think about this broadening it to to other types of developmental disability as well, if if you have any thoughts, but you know, one of the things that we’re interested in doing is, you know, if we know that there are health disparities because of poor quality of care received, you know, one of the things we’ve talked about doing is somehow identifying disability status or accommodative needs so that that can better inform healthcare practice. So in that sense, you know, do you think – or what would be the appropriate way of identifying people with disabilities and their needs in a given healthcare setting?**

F: (inaudible)

**I: I guess that could be how, when, where. (at same time) Sorry, go ahead.**

F: I think it’s so low…well it’s high incidence but people just don’t see it. Like every - I think we’ve taken, in general, glasses–I wear glasses, you wear glasses, whatever - I think we’ve just taken into consideration that glasses– technically like you can’t.. you’re wearing them because you can’t see–like, some people wear them for whatever, but like, I need to see. So, I think we just don’t educate our social society enough, and yes, where the hospitals like in the healthcare division in general are aware, but they’re not aware. Like they work at, like we go in and there’s accommodations for X, Y, and Z in that practice, they get it. But if I go like with my son, or if I go to another, they have no clue. Like when my daughter, when we went to find the new PCP and our first PCP actually said something to me, and he said to me “I’m gonna be honest with you, I don’t really know about infant hearing loss…I know a little bit”, he says, “But you’re gonna be the expert in that field right now to communicate to me”. And I’m like “Oh my God…like what” Like I don’t know…bit it’s just - and I don’t expect the doctors to be experts on everything, they have to have general whatever, you know, but I think just in society in general as well as health care professionals we have to understand that just because it’s not what we see every day doesn’t mean it’s less important or we shouldn’t accommodate. We should accommodate, we should try to find out how, and I think part of it is they don’t ever ask, like they don’t–and I know they’re busy, like I get it I know, I know they’re busy. Like to get in to see her ENT, like they come in on their days off and they stay late–her Audiologist doesn’t work on Mondays, she switched her day! I don’t want her to do that–like I really don’t want her to do that–but she understood because we were driving five and a half hours and we had an appointment that same day, and they made sure all three appointments were all on the same day so we wouldn’t have (inaudible) a whole day. But those accommodations to understand that number one she’s four years old, her speech is delayed, I have no clue half of the time what she’s saying, I get the gist of the conversation, but to understand that they have to go with her, and it’s not me, you know, they have to accommodate the change towards her, and the more that you get her on your side, the appointment’s gonna go faster and she’s gonna be more cooperative.

**I: Right, absolutely…So in the maybe the initial stages of, you know, finding a healthcare provider and seeing them, you know, how do you think this type of information should be collected? Is that something that they should ask you in person…over the phone before you even come, is there, you know, something in like patient registration forms, or what do you think might be the best route for collecting this information?**

F: I think…well I know most of the hospitals, or doctors, or whatever now…I don’t remember this ever being the case, but now they send you a packet before you go, like you have to fill out all of these papers and all this mess–sometimes they’ll send it to you in email or mail it–if they have a question: “What are you looking for in this type of provider?”–I want, personally, if I was going to go see an ENT, I wanna make sure that he is exposed to other deaf or hard of hearing children, I wanna make sure that he is not an adult audiologist or a PCP, like my thing is it would save me money, appointments, and everything, because if I went into the PCP, and she wasn’t gonna listen to me in regards to a referral because my daughter’s current PCP is connected to a hospital, and they wanna refer to a hospital–I don’t wanna go to that hospital, I wanna go to my people. So, if they said–and I would like that on the thing–I’d say hey listen, I’m looking to establish practice, and I’m looking for a connection with my doctor, but also that they’re gonna respect my decision, I wanna go where I wanna go. And they’d say “no”, they’d tell me when I first went, we have a referral to you, and I’m like, worst case scenario in my head, and I didn’t tell her this, I’m like oh my god, if they don’t let me go, I’m gonna have to find another PCP, and you know what happened? The lady I was checking out with called me back and said “The referral has already been sent to the doctor that you want”, and I’m like “Oh my God”, like I want you to listen to me, that’s it, I want you to listen.

**I: Mhm. Absolutely. So, um, I guess, um, so we’re getting at, yeah, some of the things that you would share with them about your expectations but is there anything you would hope that they would ask you ahead of time about her care or things that she might need as far as how she likes to be interacted with, other needs, any changes to communication or physical environment–anything like that?**

F: I think that if they understood..um…what I wanted…um…I think it would be beneficial. I think…I just want them to listen! I don’t –I expect to learn from them, you know. And each parent to patient wants different things from their providers…so…um…I can only speak for myself… I’m probably a crazy mom, and I laugh when I tell people I’m a crazy mom, but like when my daughter was diagnosed at six months old–she was diagnosed at birth–but at six months old I left, and I joined (inaudible), so I’m the executive director, I’m on the (inaudible) of Florida, I’m on the Eddy group, like I’m–advocacy classes, like I’m the crazy mom. Not really the crazy mom, but I’m the crazy mom. And it doesn’t – and not every mom is like that, not every dad is like that, my husband he’s like - he’s involved but he copes and he deals with things differently, so the more information for him, is not good, like is not good. He just wants the general consensus, and how he – and how the provider wants to get here, he won’t ask questions. Um..I wanna know why the provider wants to get where they want to go, um, and that’s okay. But I think if they understood where, what the patient wants, and what the patient’s expectations are, I think it just helps. Not every doctor and every patient is gonna mesh well, um, but if they understand it’s a four year old, and I’m telling you like I’ve been - we just saw another ENT to check up with her ear because they didn’t want us to go all the way, whatever, she was great–like she was fine–like he played with my daughter, like when we walked in, he stooped to her level, like he got her, she’s a kid, like she got her–you know. And part of the whole thing is like, yes, I wanna make sure that you know what you’re doing and yes absolutely that you be supportive, but you’re supposed to educate me as well. You know, you’re the professional, but you need to help me get to where I need to be.

**I: Mhm, right. And do you feel like they should, you know, follow up, you know, do you feel like that type of information is just a one and done when you first are received as a new patient or do you feel like that’s something that they should do on a regular basis to make sure that any change, obviously as she’s growing older she might have changing needs, is that something that you would want them to check in on on a regular interval, and what would that interval be if so?**

F: I think yes, um, and it depends on the needs of what, you know, the thing is. Her ENT suggested a developmental pediatrician, so we went and saw one, and then she had me followup, and he asked (inaudible), and I was like “Oh yeah, she’s done he doesn’t expect to see her for (inaudible)”, –“Okay, fine” So when her next follow up is in like two years or whatever it is, he just wants periodically to check her in to make sure that that professional is watching her to make sure that she is where she needs to be. And that’s part of, you know–and sometimes that’s six months, sometimes it’s a year, sometimes it’s five years, you know, like it depends on the severity (inaudible). But to have the trust in the other professional to put in your calendar in the chart, you know, to pop a year from now: “Hey, [daughter’s name] had an eye appointment a year ago, she’s due for an eye appointment now, just to check”, I don’t know.

**I: Mhm.**

F: I know they’re busy, I really know they're busy, and I told my husband, he thinks I’m funny, I said if her ENT told me to lay in front of a train I probably would lay in front of a train because I would truly, truly truly, give so much. And my brother has cerebral palsy - my mom came down this summer while my daughter had surgery and my mom doesn't like hospitals. She’ll like faint at the site of blood. And she would take me into the activation appointment, and she says… and she met the ENT and she says, he’s genuine. I understand why you talk so highly of him. I said I know, because I feel like he cares for her. It’s not his kid but I think he would like adopt her, not really but you know? I had that connection with him. And I would call him from my house…I’m very easy to be like… I understand you guys are busy, I understand that you have these… but if you don’t understand what I what I need, it’s not necessarily what I want, sometimes it’s what I need.

**I: Mhm, and I’m curious, I don't know if you would know this but, you know, is is are these health care providers that you found extremely rare or or do you think that would be more common and also are you aware of any trainings that they had, whether in school or after school, that would have prepared them for this quality of care?**

F: One moment…um I find um because the first hospital I took her when she got her hearing test, it was not a pediatric hospital. Umm and then I took her to [Mass Hospital], which is not a pediatric hospital, they do pediatrics, but, I have personally found that, and I could be totally wrong, but, I have found that pediatric hospitals and pediatric staff are completely different than non-pediatric hospitals so I’m adamant about taking my children to pediatric hospitals. The level of care, the level of staff, umm it’s just, you know, when we went to [Pediatric Hospital], I realized it wasn’t - it’s not really a pediatric hospital. They took over the wing when the other one moved out so they’re a pediatric hospital, but you can tell this wasn’t… you can just tell. I didn't know what it was when I went in, but like when we got out of the car and walked into the doors the nurse came in and had toys for my daughter and they had her chart already ready and they sneaked out and they were doing jumping jacks with her. They… that’s what I expect in a pediatric hospital. Like I don't expect to go in and say “hi my name is doctor blah. I’m here to see whats wrong? Please sit on the table.” Like she’s three or four, like you have to, you know, you have to give a little bit to get a little bit.

**I: Right, right. And so what are their primary communication methods with her now?**

F: Verbal.

**I: Okay, okay. So those are all my questions. Given kind of the train of thought and where we’re going here, is there anything else that you would want me to know that I should know that might help guide these efforts?**

F: Umm I think, you know, with what you’re doing, I think it’s an awesome idea. I think it’s a really broad… I think it’s gonna be, umm, because, you’re not just focusing on pediatric, you’re just focusing on health care in general.

**I: Mhm.**

F: Right?

**I: Right, correct.**

F: Umm… I’m thinking maybe if you get more grants further down the line it would be nice to see, to compare, general… well… and then I know parents, you know, that do go to general hospitals and not just specialized pediatric. I would love to see the difference in connection of what a pediatric ENT.. the connection and the relationship they have, than with someone who is a pediatric adult ENT, PSP, a hospital, whatever it is. I bet you it’s gonna be completely different.

**I: Mhm.**

F: Umm and not everybody can deal with kids. I get it. Like I even need a breather. I just, I personally found, not that they’re more cold, but like, you’re at a kid hospital, like, or you’re dealing with a kid, like the kid’s sick. Usually they’re because they're sick, or something is wrong, or they’re not feeling well or whatever. But like, you know, there’s some things that you're like oh my gosh. People are in it, you know, in health care, I’m sure the majority of them are there because they want to help, they want to do better, there are some who are there for the pay check, umm but, to be able to do that, you know, you have to have that empathy and that (inaudible) mentality. You know, you have to have it.
