# Supplementary material for: Disability disclosure in healthcare settings for individuals with developmental disabilities: A qualitative study of patient and caregiver perspectives
Source: PLoS One. 2025 Aug 7;20(8):e0329328. doi: 10.1371/journal.pone.0329328 (PMC12331114; doi:10.1371/journal.pone.0329328)
Supplement: S1 File — (ZIP) [file pone.0329328.s001.zip › Transcripts/2019.08.16 Interview 02 Transcript.docx]

I: Interviewer M: Male informant F: Female caregiver informant

F: (recording seems to begin mid-sentence) he understands him

M: I live independently

F: Yea

M: Like my brother

F: Yep mhm. . . he does

M: He’s in California

F: Did that answer your question properly?

**I: Yea, yea absolutely. Um so have, you know, you talked about umm good bed side manner and having a kind of a personal touch and getting to know patients and things like that. Umm and also your more proactive side of having conversations and finding the right referrals and finding the right doctors and not sticking with one unless they were right. Umm but what, what if any accommodations beyond that would you say a doctor might offer knowing that there might be unique needs?**

M: (clears throat)

F: . . . well . . . I kind of feel like the answer to this is nothing. I feel like you’re asking the wrong person here because I don’t have a lot of special needs.

**I: Sure.**

F: Um . . . so if you’re, someone’s in a wheelchair, obviously your doctor’s offices are compliant. . . umm

M: wheelchair means (inaudible)

F: Something that’s a little off topic but having information umm in the office brochures and information and being knowledgeable about things . . . where to send, this this being very knowledgeable about where to send the family. In addition to, let’s say you’re going to a specific specialist and he is the right or she is the right person, but they can still point you in other areas to get information be it online or a local organization or they can have, you know have a have the knowledge of send it so this is an example, it’s not really quite the same. But the Med Waiver is a major source of funding that many people don’t know about it, and none of the trainees, rarely do they know about the Med Waiver and it’s a huge source of funding so . . . the services for him to live independently at fifty-seven thousand dollars . . .

**I: Hmm.**

F: Right?

M: But (interjection)

F: That’s just the services, for the support, it’s the same as assisted living.

M: But Sunrise (interjection)

F: You can’t do it unless (**inaudible**) you won’t believe how many people don’t know about it. There’s a huge waiting list and you can get on it at age 3.

M: But Sunrise mom (interjection)

F: So every pediatrician should be … yes, I know. I’m sorry I wasn’t meaning to. Sunrise Community is the agency that runs his house so ...

**I: Okay.**

F: For doctor’s offices to be able to give you information about, you know let’s say you’ve got cerebral palsy, well this is the local support group, and or you’re intellectually disabled you know you can get on this waiting list and it’s a very important waiting list and this is the agency for persons with disabilities, you know. A lot of what I know I’ve learned by seat of the pants, you know there’s no one place, and even if there were you wouldn’t know it. You can’t even do these half of these things till you’re ready, the moment has come. So, if he’s four and someone is going to start talking to me about what he has to do when he’s eighteen, it’s too far away, I’m busy getting through each day.

**I: Mhm.**

F: So sometimes you can only deal with it when the time is right for your family whatever that is. But if you have a, especially a pediatrician who, well usually they’re with the pediatrician for years from birth to whenever they make you leave. (laughs)

**I: You’re right. (laughs)**

F: So they will see everything and they are especially important I think in disseminating knowledge.

**I: Right, so it would be helpful if they had that knowledge to refer out.**

F: Ummm back in the day I sent him to the Miami, the [School A].

M: My favorite.

F: Because there was a waitlist.

**I: Okay.**

F: So he started that when he was 3 weeks old.

M: There’s some people that I know that left.

F: But the [School B], all these other places, there was a two year waitlist. Well, that’s two years too long. So, two years down the road when they started calling and offering a place by then I’m already been at the [School A] for two years where he still goes cause know we just made it be adults as well.

M: Silent campus know.

F: Cause you develop this incredible relationship with your therapists, I mean when they leave you cry. With your doctors. At that age it’s so emotional, it’s so emotional. And umm that’s (**inaudible**) probably getting off tangent, off target here.

**I: No no, thats (inaudible)**

F: You have very close uhh emotional relationship with the therapists, with the teachers. We found out, that’s what I was going to tell you, we found out about that school by accident. Because it happened that one of [doctor] partners had been on the board and it happened that someone else had told us about it. Remember this is 32 years ago. But there’s no brochure, of course now everything is online, but there’s nothing in the doctor’s office to tell you; it was an accident. Right

M: [Person A] (**inaudible**)

F: We might have found out about it four years down the road or something, so having that information . . . I particularly go back to pediatricians offices there (**inaudible**) it really would be fabulous.

M: [Person A], [Person A] (**inaudible**) my favorite

F: Yea

**I: So do you, do you find that umm getting that information from a healthcare provider or a doctor would be umm better received than if it was just somewhere else or you would want to receive it from wherever it comes from?**

F: Wherever it comes from, but it often hasn’t come from a doctor. It’s often come from the parents

**I: their parents and support**

F: and that’s very important, but you know, it would be wonderful if it came from doctor’s offices, I think. I think they should be a prime source as well, you know.

**I: Right, okay.**

F: That helpful?

**I: Yea, absolutely. So no, so no negative experiences. That’s that’s great to hear. So you feel though that you worked hard to get to that point by choosing your doctors well?**

F: Yea, I think. You know, when I go in and I meet them I know right away.

**I: Mhm.**

F: It doesn’t take very long.

M: And she has a thing that goes in her car, right mommy?

F: Oh, a tag. A tag, yea.

M: Yea.

F: But, you know your friends will say to you. Like recently, I discovered that his eye doctor had moved to New York and we had fallen in between the cracks.

**I: Mhm.**

F: And I was filling out his guardianship thing and (**inaudible**) annual report and I’m like huhh how come it’s been two years since we’ve seen [doctor].

M: She’s my favorite.

F: Well then, I didn’t know where to go. I could no longer ask her way to go, cause she wasn’t there. So I asked one of his roommate’s mothers who’s my really really good friend where her son went.

M: (**inaudible**)

F: So that’s where he goes now and I felt that anyone that she referred was good enough for me cause I respect her and trust her.

M: [Friend A], right?

F: No no, [Friend B]. [Friend B] goes to the same eye doctor as you. Now [Doctor].

M: Yea.

F: Your new one. You may not remember his name yet.

M: No

F: Cause (**Inaudible**) so . . .

**I: Mhm**

F: So yea. So I mean I trust, you know. When a friend says watch this movie, you know it’s a friend who

**I: who has your best interests in mind too.**

F: (**inaudible**)

**I: So so so question then. It sounds like umm this good bed side manner is something that they would deliver to any patient and is not so much umm specialized training that they’ve had in in working with with patients that have any form of disability?**

F: No, I think it’s who they are as a person.

**I: Okay.**

F: Maybe that’s why they went into it. They want to help people.

**I: Mhm.**

F: Umm . . .

M: The next president of the United States. (**inaudible**)

**I: Oh yea? Haha**

F: Somebody umm . . . I’m just thinking. Did they? I don’t think back then cause none of them are young.

M: (**inaudible**)

F: They didn’t do the LEND Program, hahaha.

**I: No, wasn’t around.**

F: But I think that’s a really great program

**I: Yep, yea.**

F: Because you know it’s people don’t . . .

**I: That training doesn’t, it’s not readily available.**

F: It’s just an eye opener I think. People like it. I mean the trainees seem to like it. My little bit anyway, I mean I have started on every class because I wanted to know what the whole thing was about and I think they like all of it.

**I: Mhm. So ummm, one of the things that we’re looking at, we know based on research that’s been done, we know people with different types of disabilities have poor healthcare experiences. So that, you know, is one of the things we’re looking at. Umm. But in order to do anything about that we have to, you know, in the process of providing healthcare document disability status. Umm so one of the questions that I have is you know, is do you have any recommendations or any suggestions for how we would identify disability status in the hospital or in the doctor office? (9:27)**

F: Umm so somebody walks in who is not obviously . . .

**I: Mhm, yea if it’s not visibly apparent.**

F: Well you fill out, you fill out, you’re always filling out umpteen pages of stuff.

**I: Mhm.**

F: You can have a question on there about . . . umm I’m thinking how to word it . . . maybe something like is there anything else you’d like to tell me, like us to know about yourself.

**I: Mhm. So keep it general?**

F: Well, you can’t say . . . I mean to have a question that says ‘Do you have a disability?’ is a little bold, isn’t it.

**I: Right, okay. Mhm. Well let me show just as an example.**

F: And in fact, [Person B] was talking today about when he got diagnosed with autism, which was quite late in life, he absolutely didn’t think he was disabled, he didn’t like associating with the idea of disability. You know, so that could be off putting.

**I: So asking you directly wouldn’t, would probably not be . . .**

F: I think it might be. What do you think? I mean, I think saying something like ‘Would you like us to know anything else about you’ or ‘Is anything important else about yourself that you would like us to know’ for example might be . . . umm

**I: A better way to, or a more sensitive way you would say-**

F: Yea, I’m thinking it might be.

**I: Mhm.**

F: It might be. That might be the way to do it. Because if it’s not going to come up in conversation, what’re you going to say? You could also, you could have that on the uhhh stuff at the beginning, on the form. But you could also ask it again in person, the same question could be asked verbally. Umm because they do get spoken aloud, you know like I’m doing now, spoken. Because umm they ask you things umpteen times, you go in, you’ve already filled it out, and then they do a check, especially when you’re having any kind of procedure like an MRI. I mean they must ask you the same questions at least twice that you already wrote down, and they’re obliged to do that. So you could have a double check with a nurse or whoever is it. What do you think?

**I: Mhm. Well that’s, I mean I have I certainly have ideas, but I wanted more so for yours. But let me, let me at least show you not not that this is a suggestion but just to give you an idea, you know umm. These are questions that umm a survey thats used to collect the census data uses to assess or to determine disability status. So those are the questions that are kind of the starting point for suggestion when thinking about if we were going to ask something, what would it look like. And we talked about in different formats, would it be in electronic medical record, would it be verbally in the patient intake form. So that’s the general question, what, uhm, how do you ask it? Obviously, you said more general might be more sensitive. Which would be one way to do it. This would be getting at more specifically different types of disability.**

F: [AM02]?

M: Yes, mommy.

F: If you were answering these questions, ‘Are you deaf or do have a serious difficulty hearing’?

M: What does that mean?

F: Well you know what that means.

M: Yea.

F: Are you deaf?

M: No.

F: Do you have difficulty hearing?

M: (**inaudible**)

F: So the answer to that is no. Right, okay?

M: Only my grandfather did . . .

F: Are you blind or do you have difficulty seeing even when wearing glasses? What’s the answer?

M: I could see.

F: So the answer’s no.

M: No.

F: Okay.

M: Next question.

F: Because of a physical, mental, or emotional condition do you have serious difficulty concentrating, remembering, or making decisions?

M: Mmm . . .

F: That would be a yes or a no. So, first of all, let’s take the end of it. Do you have difficulty concentrating, remembering, or making decisions?

M: I make the right choices for everything foods.

F: So the answer would be no. Do you have difficulty walking or climbing stairs? It’s a yes or a no [AM02], you don’t have to think about it. Do you have a problem getting up the stairs?

M: No.

F: Okay. Do you have a problem dressing or bathing?

M: No way, I do it by myself.

F: Can you do errands on your own?

M: Yes.

F: No, no. Errands. Errands all by yourself. Can you go a doctor’s office and go shopping all by yourself?

M: Parent supervision.

F: There you go. So ummm again, if you had him to answer it.

M: Last one.

**I: Absolutely.**

F: I think you’re assuming that the parents are going to answer these questions.

M: You forgot the last question.

**I: Yea, this is, yea this is probably not what we would use. We would probably do something shortened, easier to understand. This is more getting at, you know, are we capturing the right things, is there anything else that we would (inaudible) asking that question. You know what else would you like to tell me, is there something else that we need to know that wouldn’t be captured in those types of questions? (14:52)**

F: Right, right, right. I personally like him to be able to do as much on his own as possible. So when I’m going to get into the doctor’s office, I would try not to answer the questions. I’ll help him, but I want him to be able to do it. So if there were a form that he could understand, I would prefer that. I understand that’s not the probably the majority of cases, but . . . I remember we had someone from special Olympics come to learn, and she was saying how difficult it was because not everyone is in [AM02]’s position.

M: Oh yea I’m also in Best Buddies.

F: Yea, well. I don’t just mean that. I mean having parents who are involved and who can give him all kinds of things that many people can’t get. So if you’re intellectually disabled and you’re living in a group home and you don’t have any family, you’re in a completely different situation. And what, uhh I’ve forgotten her name was talking about, was the difficulty because to do special Olympics they have to have ummm fill out a medical form and have it signed. Well they don’t have anyone to even get them to the doctor’s office. So she had I think done a presentation over at, well I went to see if UM could, I don’t know, do something, help in some way because people were not being able to participate and this is one of the few things that they’ve got in their lives. (16:25)

**I: Just because the form was in the way.**

F: Just because of this form. And so, that’s a whole different segment of the population there that can’t help themselves.

**I: I’m sure.**

F: And don’t have a me.

**I: Mhm, to help out.**

F: So you want reach that population.

**I: Mhm. Okay.**

F: I don’t know how you do that because it’s a barebones existence.

**I: Mhm.**

F: They probably get SSI, pay 500 dollars in rent, get food stamps, and they have a grand total of 221 dollars left over. No, 271 dollars.

**I: Right. So there might be a threshold that we might not be able to do something without a caregiver you’re thinking?**

F: Yes, and from my understanding from what she said was in a group home, ummm you’ve got shifts and more than one person to care for and even knowing that you have to do it or it needs to be done if the individual is not able to tell you ummm. (**inaudible**) it sounded dire and you wished you could solve the problem. And where are the guardians and is it just a bank, I don’t know, you know just a trustee, I don’t know.

**I: Mhm.**

F: Anyway, that was . . .

**I: Right.**

F: So you’re probably never going to survey anyone like that?

**I: No, not probably because then we wouldn’t be able to do informed consent.**

F: Well they wouldn’t be responding, they wouldn’t be receiving the email.

**I: Mhm, absolutely, only through a caregiver if at all.**

F: Yea.

**I: So umm so we we answered yes to to this last one, you know, if some form of of asking the question, whether it be in a general sense or more specific sense, was wasmdone and and there was a yes like that, what what would you expect a doctor to how would you expect the doctor to to respond or or what would you expect the doctor to do for you because you said yes?**

F: So, if the doctor asked you

M: Mhm.

F: if you could . . . uhh what was it I forgot what it was . . . shopping or something. Doctor’s office. If you could go shopping and go to your doctor’s appointments on your own and you said no right? Because you don’t go on your own, do you?

M: No, I don’t.

F: So you say no. And then she might say,’ why not?’ What would you say.

M: Let me talk to my parents.

F: No, you’d say . . . Why don’t you go on your own? Because you have down syndrome?

M: Yea.

F: And what does Down Syndrome mean?

M: Disability.

F: It’s a disability. Makes it sometimes harder to make choices and decisions.

M: Yea.

F: That’s me putting words in your mouth, I know.

M: Hm.

**I: Mhm.**

F: I just said he can do anything he wants, it just takes him a bit longer than most people.

**I: Mhm.**

F: Because although he has a disability, doesn’t view himself as the same.

**I: Oh no.**

F: Its quite hard to explain. We found when we did the SALT training it was quite difficult to explain that bit.

**I: Oh yea? Did you like SALT? (20:03)**

M: Yea.

F: How many times did you do it?

M: I don’t know.

F: Well you did it twice. Because he was in the very first cohort and it changed completely. So it was much shorter and I said to [Person C] do you think Alexander would be a candidate to do it again because it’s so completely different now and she said absolutely. So he did do it twice, with [Person D].

**I: Oh, you did it with [Person D]?**

M: Yea.

F: The first time I think.

**I: Okay.**

F: I tried to remember but he knows [Person D] that way. And also from the social cog.

M: Yea.

F: Yea.

**I: [Person D] was in my class when I went through ETLP.**

F: There you go.

**I: Yea, yea. He’s great. And he’s also working on this project with me too.**

F: Oh nice, nice. He always asks after [AM02].

M: Yea.

**I: Oh yea?**

F: Yea.

**I: [Person D] is a great guy. Umm so just back to that question one last time, would you expect the doctor to react or respond or ask follow up questions or anything in particular that would be helpful to [AM02]?**

F: I’m going to ask you, if the doctor is seeing him on his own or the individual on his own or with the parent, cause then it’s completely different.

**I: So let’s tackle them both then.**

F: Okay. So if I’m there, and it’s not obvious, then yes I think it would the doctor should say “oh why not? is there something I should know? I might be able to give you information” you know whatever however they want to say it. But yes, I think a doctor’s role should be holistic. It’s not just a sore throat, it’s everything, it’s the mind, it’s . . . I think it should be a holistic approach.

**I: Mhm.**

F: Me, but. So, yea I think then if you’re going to be seeing someone who’s on their own, same thing really. I think it should be a holistic approach. And yes, I think it would be great if they gave information and asked questions like that, more probing questions.

**I: Mhm.**

F: Perhaps, yes. Is that answering?

**I: Yea, absolutely, I mean it’s just getting a sense of what you think might be necessary. So you said before, [AM02], you might need a little extra time to make decisions or do certain things, do you feel your doctor’s give you that extra time?**

M: Uhhh, mommy?

F: That’s too difficult? That question?

M: Yea.

F: Hm . . . It just hasn’t arisen.

**I: Because of their good nature?**

F: It hasn’t because of the nature of why . . . for example, when we went down and had him tested for sleep apnea we had to go spend the night. He and I had to spend the night, right. We slept there.

M: Yea.

F: We slept there.

M: (**inaudible**)

F: No, you didn’t like it but you did it, didn’t you?

M: Yea.

**I: Oh that’s when they do the tests, you had a mask on?**

F: Yea, twice they do it, I can’t remember why. Why it was twice. But it was twice. And ummm so then I knew that there was a procedure you could have done, an implant. And I never thought that he . . . I just before I’d ever gone to [doctor] I thought (**inaudible**) not gonna happen, you know.

**I: Mhm.**

F: And he’s conservative and he doesn’t want to do surgery unless it’s absolutely necessary. Umm (**inaudible**) was super comfortable with him. So when [doctor] explained about the CPAP, what did you say?

M: What the heck.

F: Yea, do it.

M: (**inaudible**) (23:50)

F: Went home, took like a (**inaudible**). Strange.

M: And I even, and I even do something on my (**Inaudible**)

F: (**inaudible**) cause I thought he was having too many incidents and I wanted to check it cause he’s not in my house, so I asked him to write it down so they keep a chart. And so we went back to see [doctor] and he said everything was fine. Does [doctor] use a CPAP?

M: Yes.

F: He does.

M: Yea.

F: So that is also a nice thing cause they’re like hey guys we’re the same, you know.

M: And my dad to.

**I: You all use the same one? Same kind?**

F: Not the same masks.

**I: No?**

F: I asked [doctor] if he became a sleep doctor because. And he said “No! I didn’t find out till we were in medical school and everyone had to try it out. And I’m like oh, suddenly I find out I have sleep apnea.” Which he didn’t know and he’s thin. So you know, it’s not necessarily that you’re overweight.

**I: Right. Mhm.**

F: Umm so that was kind of funny. So I remember when he was having a psychological for the umm to join the social cup group. And we were waiting for Dr. . . .

M: You forgot?

F: (**inaudible**) anyway he’s super nice. So we were waiting there

M: You forgot his name?

F: [Doctor].

M: Yea, [Doctor].

F: So we were waiting there in the waiting room and he comes out and he leans down and he says “Hi [AM02], I’m [Doctor].” You know, and that was perfect. They were just like best buddies which meant he was relaxed, liked him, and that is . .

M: Nice and slow.

F: that sets the tone for everything to follow.

**I: So it kind of reduces any chance for (inaudible)**

F: We are equals here. Big time.

**I: Mhm.**

F: We’re friends, we’re equals, I understand you. Ummm that kind of thing for him. And I though, oh I like him already, he knows what to do.

**I: So they always you would say they always get you understand you?**

M: Mhm.

**I: You get along in that way?**

M: Yea.

**I: Is there any time where you have to like correct them because they didn’t get it right or they’re wrong about something?**

M: Mmm.

F: Don’t have to look at me. She’s asking you the question. Have you been comfortable with all of your doctors?

M: Yea.

F: They seem to know what you need?

M: I’m not the one to correct people which (**inaudible**) do.

F: Yes, Alexander is not assertive and he doesn’t like umm . . .

**I: So you don’t like speaking? (inaudible)**

F: He does not. He is learning. He is learning to speak up. He’s learning that his opinion is as good as anyone else’s, that he’s an equal.

**I: Absolutely.**

F: Umm and this is one of the things that when I have time I talk about is that people his age umm if you think about it, the adults in their life have always been authority figures. Everybody is an authority figure, they’re always being told what to do.

**I: Right. And you’re taught to listen to your elders kind of.**

F: So when [AM02] moved out, we had a few . . . I suddenly realized that I had to teach him and (**inaudible**) you can have an opinion that’s different, just be polite when you express it. But you can have a different opinion, but he doesn’t want to hurt any one’s feelings, he doesn’t want anyone to get angry.

M: Or yell.

F: He doesn’t like people yelling.

M: Or shouting.

F: Umm but you can say anything you want, right?

M: Yea.

F: As long as you’re?

M: Polite.

F: Exactly.

**I: So you feel like you’re getting more comfortable with that? (27:36)**

M: Mhm.

**I: That’s good.**

F: It’s a work in progress.

**I: Mhm.**

M: Yea.

F: It’s quite interesting, I didn’t know that I was going to have to do that. I just found out once he moved out, you notice like huhh, I didn’t realize that. You know. But that’s his personality not everyone’s like that.

**I: Mhm.**

F: So, uhhh . . . So, no I don’t think that we’ve had any doctors that tried to get him to do something he didn’t want to do. We use logic and we tell him what’s going on, and he usually understands that and makes the right choice.

M: Yep.

F: He listens. He may say I don’t like shots.

M: I don’t.

F: But he still has them.

**I: Oh, yea? You put up with it? You let them?**

F: He knows it’s the right thing to do.

M: Yea, right.

F: Nobody likes shots.

**I: No.**

M: Welcome to my club.

**I: Absolutely. So anything else that I should know in terms of what makes the doctor experience a good one? Or things you would tell a doctor to do in order to be better?**

M: Mommy?

F: Okay, so it’s treating people like they’re individuals and human beings. So umm . . . did you read that book umm by uhhh something like Breath for Air? Did you read it?

**I: No, I don’t believe so.**

F: I would highly recommend this book. It’s written by a neurosurgeon who got lung cancer. And ummm he’d almost gone into literature I think. And this is a really excellent book. But what he talks about there is treating patients like people. *When Breath Becomes Air* by Paul Kalanithi, have you heard of it?

**I: No, I haven’t.**

F: It’s an (**inaudible**) by Abraham Vergese. This is it, *When Breath Becomes Air*. Was an excellent book. So in there he talks about, alright for example, it’s one night where they’d been on duty and there’s a patient (**inaudible**) and they’ve had like umpteen patients, and one of the other girls on residence or whatever says “oh my god, I hope he dies so we don’t have to deal with another person (**inaudible**).” And then of course she felt terribly terribly terribly terribly guilty.

**I: Mhm.**

F: And it was, that was just something about seeing the person as a person or seeing them as another . . . you know. Umm . . . so this is an excellent book, I highly recommend it.

**I: Yea, thank you. So I mean those are all my questions, other than [AM02] how did you get into Tae Kwon Do?**

M: Uhh (**inaudible**) baby.

F: I want to tell you one other book that’s very good. By this is

**I: Okay.**

F: You know, so, there’s ways and ways of communicating these things. I’ll give you an example that has got nothing to do with disabilities other than the fact that I had got diagnosed with something. I had gone to see a neurologist, I had an MRI. So over the phone, he said to me “well, it’s either inflammation or a tumor.” On the phone, right?

**I: Mhm.**

F: And I’m scheduling to go see [Doctor], not [Person E], the husband, is it [Person F]? I don’t know. Over at UM. Anyways, so that was took like two or three weeks, my husband was out of the country. And he basically left me curled up in a fetal position because tumor is a very scary word.

**I: Absolutely.**

F: Did he say to me, it’s an ependymoma, they’re usually benign, they’re very slow growing? No, nothing.

**I: Right, no . . .**

F: I found that out through my sister who had done a residency with a top neurosurgeon in England and she got my (**inaudible**) and she got me that information. I have so blanked this doctor out that I don’t even know his name, I never went back to him.

**I: Understandable.**

M: (**inaudible**)

F: There’s a doctor and it’s the same for someone who’s got a disability . . . imagine if they had a person with a disability . . . there’s ways of presenting information, there’s ways of

**I: not escalating things**

F: scaring

**I: when it’s possibly not even necessary.**

F: Yes, yes, At the same time, they you know, when [AM02] was born, they won’t tell you. They say we, “it’s possible,” but until the tests come back, they will never tell you. And that’s just, they won’t. It’s not going to change. They’re probably not allowed to, I don’t know. Ummm, but a, I just wanted to say that because I remember that. Somebody explaining something to you simply, in a way that you could understand, that will . . . my doctor tried for three years to get me to take Synthroid, and then finally he explained it to me in a way that made me agree with him.

**I: (laughs)**

F: But I have no symptoms!

**I: Why didn’t you say that?**

F: No, I knew that. But then he explained how okay yes, but you’re thyroid is going to give out. It’s a question of when. It might go (claps). Or it could go slowly if you take a low dose now, you could (**inaudible**). Oh yea okay that makes sense, yes okay I’ll do it. Finally. It took him forever to convince me. But explain to me in a way that made sense to me. Umm so . . .

**I: So same . . process**

F: Same thing really. We are talking about people aren’t we?

**I: Absolutely. And I think that’s the fundamental, that’s where we’re getting at, it’s just about dignity and respect. More than anything else.**

F: It is. It is. And I think nowadays all your patients are such doctors themselves because they’ve read it, they’ve googled it. And they have opinions. Why are you doing that to her?

M: Huh?

F: Why are you doing that?

M: Me and [Person G] do that. Remember?

F: Anyway, yea we google it and we have an opinion. Whereas before we didn’t have opinions, we just took what the doctor said, right. Now you probably got patients that are way too opinionated.

**I: Right, self-diagnosing and all that, yea. So that’s why you’re wearing Elvis’s shirt, because it’s his, the day, it’s the anniversary . . .**
