# Supplementary material for: Disability disclosure in healthcare settings for individuals with developmental disabilities: A qualitative study of patient and caregiver perspectives
Source: PLoS One. 2025 Aug 7;20(8):e0329328. doi: 10.1371/journal.pone.0329328 (PMC12331114; doi:10.1371/journal.pone.0329328)
Supplement: S1 File — (ZIP) [file pone.0329328.s001.zip › Transcripts/2019.08.22 Interview 04 Transcript.docx]

I: Interviewer F: Female

**I: Alright so we’re recording. So just for the record, just to confirm you‘re okay being recorded?**

F: Yea, of course.

**I: Okay, great. Awesome. Thank you. Alright so let’s, let’s jump right in.**

F: Yea.

**I: Um so I do know a little bit about you but all that is off the record so I want to try to capture some of those things that you shared that were really insightful from before.**

F: Yea.

**I: Um so first off if you had to kinda just say overall, have you had positive and negative experiences or one or the other in healthcare settings?**

F: I definitely have had both.

**I: Okay**

F: Um, I think that in my opinion healthcare is something that requires a lot of compassion and a lot of understanding, and I just, I truly feel that not everybody is cut out for it. Um I’ve encountered many nurses, both in, you know, in dialysis centers and otherwise like in hospital settings that were just very umm rude is the best word to put it, you know. Very rude. Didn’t want to hear my opinion. I’ve been disabled for my entire life which is 35 years and I know a lot about my health and many times when I try to speak up, depending on who the care taker is, you know, I’ll, I’ll get a different response. Many are, are open to it and are like thank you for letting me know, and others are like let me do my job like you don’t need to tell me what to do. And so and I get very offended in that because it’s like no I do need to tell you what to do because my health is a lot different than maybe the last patient that you’re used to. You know, so, I do like to speak in, in medical settings and not always is it well received and I think that has a lot to do with the fact that, you know, there isn’t like a prerequisite of compassion in the medical field and I think that there should be. I think maybe there should be some sort of sensitivity training that will umm...umm maybe monthly or yearly for nurses to make sure that they keep that compassion. I mean I know that it’s a high, intense environment and so you’re probably tired all the time and you just want to get things done, but that doesn’t mean that you need to like disregard someone else’s feelings that’s not there because they want a vacation, they’re there because they’re sick. You know, so...I mean some people may fake being sick and really want a vacation but that’s not my case (laughs).

**I: Right, right. Um so I want to delve deep, I mean you’re talking about compassion, understanding kind of those prerequisites but can you tell me more umm whether it be specific examples or just additional things that are in respect to the healthcare providers, the physical environment, anything at all. The entire experience that made it unpleasant or bad.**

F: Um, I think okay like for example, one of my biggest pet peeves is like if I go to a doctor’s office and they need to like weigh me or put me on the table, and they’re like well can you stand? And I’m like if I’m in a wheelchair why would you think I could stand? Don’t you think I would be walking with a walker or something like if I could stand. And like they like just kind of like made me feel bad, you know. Like not that I’m not okay with the fact that I’m in a wheelchair, just when somebody like makes it almost seem like it’s like a bad thing it does kind of like dig a little knife in there you know. Like you’re like oh well they like think I’m less than because I like need help. You know?

**I: Mhm.**

F: And like some just don’t listen and they’ll ask like several times and I’ll get like frustrated I’ll be like NO I CANNOT WALK. Like I have to like say it like slowly and they’re like oh okay so you need assistance and I’m like well yea if you’re going to weight me yea but, you know, to get on the table I’m okay. Like I can transfer fine. Umm sometimes if the table is too high, I will ask for assistance, um you know, but normally I try to do it myself.

**I: Mhm. And um do you have any other insights or experiences with not just the doctor but is that the same case for nurses or other front desk, like all like the staff?**

F: No, yea this is, this is all the same. You know umm and I’m trying to think like…and also like doctors tend to like be really surprised with like my age because, I don’t know if it’s a spina bifida thing, but like we all tend to look really young. Like I know a lot of other people with spina bifida and we all look like we’re teenagers even though we are like in our 30s or 40s.

**I: Mhm.**

F: And they they tend to like talk down to me until I say my age.

**I: So you’re not sure if that’s an age…so you think it’s an age thing until you tell?**

F: Yea, yea. Like until I say, you know, my age then they’re like oh. Or like they’ll talk to my mom until she’s like well she’s she’s 35 so you can talk to her, you know.

**I: Not just ableism but ageism as well.**

F: Yea.

**I: Okay.**

F: A lot of it. Or maybe it could be, you know, ableism. Maybe they’re like oh maybe she doesn’t know so maybe just talk to my mom.

**I: Mhm, right.**

F: Yea, it might be a mix of both. It might be because I look really young and I’m in a wheelchair so they’re like oh like talk to mom, you know.

**I: Do you, do find, so do you find when they talk to your mom….well first, do you, do you typically have someone there with you?**

F: Yea, my mom usually likes to come to my doctor’s appointments because she likes to be like informed

**I: Sure.**

F: With what’s going on with my health.

**I: And do you feel like they always direct toward her and like kind of disregard you?**

F: Um, I think if they’re a new doctor, they do. I think if it’s someone that I’ve seen continuously,

**I: They know the drill.**

F: Yea, then they know to talk to me and or to talk to both of us, you know, like they’ll direct whatever they’re saying to both of us as opposed to just her. But if it’s a new doctor that’s usually how it goes, they talk to her.

**I: And do you feel like if if you maybe had a less than pleasant experience the first time do you just not go back or do you do you go back and they get better? What has been your experience?**

F: Umm I think when I go back they usually like make a joke like oh well I know you’re an adult so I’ll talk to you. Like that’s how they

**I: And is that joke okay or…?**

F: Yea, yea that’s usually how they joke about it and I’m just like okay whatever, you know, and I just let it go. Umm one thing that I really do not like about being in the hospital and I really want to make a point to this, I always have a *team* of doctors because I have so much going on. So it’s whatever I went in there for, let’s say it’s a pressure sore (unclear 7:54) then I have a cardiologist because I have my heart stent then I have, you know, whatever kind of doctor, an infectious disease doctor. They all come in at different times, and they all tell me something different. As opposed to like when I’m going to leave. Like one doctor will be like you’re cleared in my section so I I’ll put in your discharge papers another doctor will come in 20 minutes late, we found this in your chart and you’re not going anywhere. And then the next one will come in and be like well you’re doing good so you can go tomorrow and I’m like…and they like don’t *talk* to each other.

**I: Right.**

F: And I’m like this is a team of doctors taking care of one case. They need to talk to one another and communicate because me as the patient, I don’t I don’t want to cause any problems and I have because I’ve been like well you’re saying I can’t go home but the doctor that just came in said that I can, so which one is it? You know? And he’s just like well which doctor was it? And I’ll forget some of their names because they’re so many of them. And I’m like I don’t know, he’s an infectious disease or like, he’s like well there’s like 40 people in infectious disease you need to know the name. I’m like I don’t know (laughs)! You know, so it turns into like this whole big mess where I get blamed for like causing all this drama and I’m like

**I: You’re just trying to get clarity.**

F: I’m just trying to get answers because all five of you are coming in here at different times saying different things and this happened to me twice actually. Once in (hospital 1) for an entire month, I had a pressure sore this big and because the infection was going down, umm one doctor said I could go and then the other doctor was like no but it needs to close first and blah blah blah you can’t go anywhere, so and that happened literally every day for like a month and I was like I just got like fed up I was like dude I just want to leave like someone discharge me, you know. And then another time in (hospital 2) umm I had, this is most recently this was um October of 2018 to February of this year, I have a dialysis graft in my leg and it caught so the doctor had to put in a new graft and he tried to put it around the clotted graft but I guess he didn’t know that the clotted graft was also infected so it made the new graft infected and long story short, he knew he admitted to me that he knew that he should’ve just taken everything out of the old graft and put in the new one, but he said he didn’t want to put me through that long of a surgery, so instead you know what ended up happening, I had four minor surgeries and had to keep going to the hospital.

**I: So, you think he made a decision based on you having spina bifida versus just a general call?**

F: I think he just based-I think he’s such a wonderful person and we’ve known each other for like thirteen years that he wanted to make it easy for me not realizing that he actually made it much harder for me. Um, so, I can’t really blame him for that because I know that he had good intentions but he, he just made the wrong decision based on, you know, maybe based on my size, on my disability, on the fact that he’s known me for so long. There are a lot of factors that I think were put into it, but it was really difficult for me that literally every-I would say every three weeks I was having another surgery for four months.

**I: Mhm, right.**

F: And, and it was a lot on my body, I mean, I and I became like really depressed like just wanting it to be over, you know. Not my life, but just the situation. I wanted the situation to be over because I just didn’t understand why there were *all* these complications. Like this man has been a surgeon in vascular surgery for I don’t even know how many years, like 30 years, so why are you messing up so much? But he told me that my case is so different than anybody that he has dealt with because most doctors that do care about me, they, they treat like a pediatric. So, they do things very slowly, very lightly. And sometimes that’s good because it’s easy on my body, but it’s also bad because they can also prolong things and make me go through a lot, you know. Umm so it’s kind of like a Catch 22 with me. And I think that’s what he was doing, he was treating me as pediatric due to my size umm and that’s why like it I had to go through multiple surgeries, because he was trying to be too delicate. But I think that there has to be a balance where, you know, you understand that my health is fragile but you also don’t put me through more unnecessary obstacles that I need to face.

**I: Do you, do feel like those situations when they happen are maybe partially due to a lack of knowledge about spina bifida or something else?**

F: I definitely do. I definitely do because I have had doctors say that like that I’m their first, you know, spina bifida patient that they’ve ever had or, you know, so I do think that that is a case, you know, is a situation and I see it three times a week in dialysis too like... I mean there’s some- I don’t know if you know much about dialysis works but they, they weigh you at the beginning to see how much fluid you have on your body and how much they need to take out, because you have a certain number that they goal that they want you to get to during treatment. So, based on whatever you are when you come in, that’s the amount that they take out plus .5 because they have to give you saline at the end of the treatment to bring your blood pressure back up. So, for me, I can’t take out more than 1.5 kilos total, let’s say, because I am so little. There are some nurses that will see my weight and if I’m a little bit more than that they’ll want to take out like 3.0 kilos and if I don’t speak up, they don’t listen, you know. So, it’s like I have to be like no like I know I’m a little heavy today but I can’t take out 3.0, you know, like that’s not going to happen. So, there’s times that it’s good and there’s times that it’s bad that like I have to be treated that way, but in certain cases like if *I* don’t speak up about it then then people won’t know, you know. So that’s why like I appreciate what my surgeon tried to do, he tried to be really gentle with me and do things like in the easy way but in the end, it backfired and I ended up having four-five surgeries in four months and was in and out of the hospital. You know, I would- this was the bad part, like I would- I said I had surgeries like every three weeks but there were times where I was in the hospital for two weeks, so it was like I would get out for a week and then go back in and have another surgery. And it was just, it was crazy.

**I: Right.**

F: It was a crazy time. And so glad it’s over umm and, you know, he was like I don’t want to see you for like another 10 years. Like I’ve had nightmares about your case like I’m done like

**I: You and me both right (laughs)**

F: please be okay for like at least 10 years with your graft and then I will work on you again. I need time to recover. So, like he actually, you know had a rough time with it, you know. So, I think that that’s just really my, my only complaints. Just to sum up everything is I would like to have doctors from different avenues of medicine, you know, communicate more when they’re working on the same case. I also would like, you know, a lot of sensitivity training and understanding that... I may look like a kid but I’m very very knowledgeable of my condition, of what I can and can’t handle, and, you know, I would like people to just listen to me more and be maybe be more respectful of, you know, my knowledge instead of just shutting it down and being like hey let me do my job, you know, because I do encounter that a lot, more so than the compassionate nurse that’s like – or doctor – that’s like, you know, oh well you’re very knowledgeable. I mean I do get that- and they’re like why aren’t you in medicine, I’m like because I easily throw up at the sight of like other people throwing up and that’s why I never went to medical school (laughs) like I can’t deal with people throwing up.

**I: Right (laughs).**

F: But I mean other than that I probably could be a doctor, I mean honestly I know a lot about like nutrition, medical stuff.

**I: Is that just a personal interest or…?**

F: No! Just when you grow up with the condition that I’ve had, and *everything* that I’ve been through and all the different factors of my illness- like I said I have a heart condition, I have my kidneys failed, I have so many different things, and it’s like you have to know. You have to know your own body at least. And so you have to learn, you know, so many different things of like what you can and can’t eat, what what you’re capable of doing, not to push yourself to much. I mean normally I’m in my manual chair and I’m wheeling, but to wheel from my apartment to here- first of all, probably would’ve taken me a lot longer and second of all, I would have been completely out of breath when I got here.

**I: Absolutely.**

F: Because I have, you know, my heart beat so I have to know, you know, when to take it easy and when to, you know, really push myself. Because there are things that I’ve learned throughout my life because of this condition that I don’t think that if I wasn’t in this position, I would have been bothered to, to know, you know. And I think that’s the issue with doctors is if they don’t encounter it, then they feel like they don’t really need to know about it until they do. And I think if you really care you need to be informed about everything before, you know. And funny story, I’m the youngest of seven

**I: Wow (laughs).**

F: and my mom would read medical books every single time that she got pregnant to make sure that she was aware of everything that a child could go through. No joke, every time she got pregnant, she skipped over the spina bifida section.

**I: Really?**

F: Every time. She was like oh that would never happen. And, and then it ended up happening. So that’s my point, like you need to be informed. And no no offense to her, she’s an amazing mom, the second that I was born she was calling every agency and organization that is under the sun that could, you know, inform her on my needs, so that was her way of dealing with it but I feel like as far as a doctor, if you’re going for medical school for that long- which I don’t know how long it is it’s like eight years or something?

**I: Yea, something, yea.**

F: So, you need to know all different conditions, you know, you’re not a pregnant lady saying “oh that’ll never happen.” You need to, if you’re a doctor, you need to be aware of all different conditions not just the hot button ones like down syndrome, cerebral palsy, and autism which everybody talks about most of the time. There are other ones that maybe aren’t as, you know, publicized that you still need to know about.

**I: Right.**

F: Not saying anything bad about any of those three disabilities, I love people from all sectors of disability but, you know.

**I: (subtle laugh) More inclusive.**

F: Yea, I think it needs to be more inclusive and it needs to be more aware of just every factor of all disabilities. You know?

**I: So, so, you mentioned that you speak up so that, you know, they know, you know, your personal expertise though, you know, that we talked about before. Umm are there any other things that you do to kind of either address bad situations when they’re happening or try to anticipate or prevent them from happening?**

F: Well I’ll tell you a funny thing. When my blood pressure is dropping at dialysis let’s say, I start yawning continuously and it’s not that I do it on purpose, but it’s it just happens in my body I just start yawning and they now know that that’s my signal for come help me, I need saline, my blood pressure is dropping and I’m going to faint. So, there are different ways that I get attention when I need, you know, help medically like and I’ll let people know like this is my signal for, you know, for my blood pressure’s low or like this is my- whatever it is. I mean there are certain ways other than speaking up that I guess you can let someone know like if you know your body and you know why somethings happening, let the person know ahead of time. Like if you see me crying it’s because I’m in pain. If you see me yawning, it’s because my blood pressure’s low. Always let your doctor or nurse know ahead of time.

**I: Right.**

F: Like these are the signals that I’ll give you if something is *really* wrong. You know, umm

**I: And do you give these things like just verbally or by demonstrating or do you actually write it down for them like in a health passport or...?**

F: No, no. I usually just let them know. I usually tell them and then they’ll watch for it like if they’re paying attention. They’ll watch for it and they’ll see like oh okay she’s she’s upset, but let me ask her if she needs medicine. Or she’s yawning, let me see if she needs saline or if she’s just tired. You know, so there’s- so then they know ahead of time like this is what to look for. Umm, you know, but I do think that it is super important to speak up either beforehand or after or during, whatever, just speak up and let the person know, you know, I don’t like what’s going on or I’m not feeling well or whatever the case may be. I mean and it took me a while to do that. Especially when I started dialysis because I was 22 and it was really the first time that I was *that* sick. Like I mean I had been through a lot as a kid, but this was like a live or die kind of situation at the time and I think I was just like so confused as like how it happened all of a sudden that I just had so many mixed emotions that there were times that I didn’t want to speak up and then it took me a while to be like no I have to, to say something depending on what was going on at the time. Umm and now they know like, especially there, that I just never shut up. I mean I told the story about how there’s there’s two doctors that come in, one is so nice he’ll stand at your chair for like 10 minutes talking to each patient, really getting to know how they’re feeling and all that, and the other one, who’s his partner who’s my main nephrologist, he just goes he looks at the chart and he walks away. So there, and he won’t even like really say much, you know. So one day, I think I had had like a massive headache every time I was on the machine for like six months, and like he- sometimes he’ll talk at you, like he won’t really talk to you he’ll just be like okay well this is what needs to be done and duh duh duh duh duh. And then he’ll walk way. That particular day my headache was so bad that I was like, “listen, I have been hearing you talk for 10 years. You’re going to sit down and you’re going to listen to me because I need to talk to you.” And he did, you know. So, I think sometimes- I’m not saying you have to be like rude, but there’s sometimes that you have to really be firm and then like for certain people to want to listen to you. And that might be in every case umm but I think specifically when you’re with a disability sometimes maybe people will overlook you and just be like oh well let me tell her what she needs to do and then I’ll just walk away. No, there are times that I need to tell you what to do because this is not okay. Like a massive headache where my eyes feel like they’re going to explode for three hours is not okay, so I need to tell you what’s going on. And so, he sat down and I think- I think it was like the longest conversation I’ve ever had with him in like 12 years. It might’ve been like 10 minutes and I was just like this is what’s going on... this is what’s going and I made like a list of like all the symptoms I’ve been feeling and, you know, then he was like okay so this is what we’re going to do. And he was like- I think that day he like either extended my time or lowered my speed, he did something and my headache started to like go down. But like if it wasn’t for me finally being like enough is enough you need to listen, I I don’t think that would’ve happened, you know. And I think that people need to not be scared to do that. I know it sounds bad maybe when like they’re- I wasn’t trying to be rude but it was like if you don’t stop him from talking, like you won’t get a word in. Like he’s that kind of doctor so I had to be like, no you’re going to listen (firm tone), you know. So, and you don’t always have to take my approach but that’s just how I felt was the best way to handle it at the time. And it actually turned into a joke because now every time that he comes in, if I do have a situation like that, I’m like “sit”, you know, and so he’s like okay I’m sitting. So, I’ve done it after that, you know, I’ve done it throughout the years after that and he respects it, you know, I think. And like sometimes he’ll come in and he’ll be like oh it’s the brat. Like do you need to talk to me about something? I’m like no I’m good today, like I don’t need to talk to you today, I’m good. So, like he’ll make a joke about it umm because I’ve known him for so long, but, you know. Others may not. If this was a different doctor, maybe they would’ve been offended the first time I did that, you know. So, I think you have to kind of build a rapport with your doctor off-hand and then if you need to get to that point where you’re telling them what to do, then they’ll understand more. But I think if you do it right off the bat, they might be like okay this person’s a little mean. So, you may have to build a rapport first, before you get to that point.

**I: Right, right. So, so what about just the physical environment or like equipment like do you do you do you encounter a lot of barriers in that sense? Can you speak about that a little bit?**

F: Ummm yes. I do think- like for for example, like the receptionist desk in the waiting room is always very very high and I have to be like hi I’m down here, you know. And I have to like wave my hand in the window for them to know that I’m there and I think, you know, it should be more accessible. It should be- it could be a little bit lower. I mean I know most people are tall when they’re standing, but I don’t think it’ll be that hard for them if they have to like write two inches lower than than here, you know. Umm just for me it’s kind of like embarrassing, you know, to have to be like hey I’m down here look at me, you know. So that’s one thing that I definitely think should be changed. Another thing umm is the changing table. I do think that all of them should have the ability to go up and down, because not all of them do. I mean there are some that I encounter that are just really high and I’m like well can it go down a little bit? and the nurse will be like no or the doctor will be like no it can’t. I’m like oh well then in that case I do need help because I can’t get up like that high, you know. Umm if you could bring it down, maybe to like there then yea I can hop my way up because I have good arm strength. But if it’s like all the way up here, like that’s not going to happen, you know. So even like that inch or two that matters to me because I know what I can and can’t do as far as like, you know, my upper body strength is very strong and I can get myself on if it’s at a reasonable height. But if it’s a changing table that just won’t go down at all, then most likely I’m going to have to ask for assistance and then depending on who the doctor is or who the nurse is, that can turn into like a whole show where they’re like oh well let me go get help and let me let me get this guy and that guy. And I’m like dude am I like 400 pounds, why do we need like 5 people to carry me. Like I don’t understand like you guys are giving me like a complex that I’m like really fat (laughs), you know. And they’re like oh no we just we don’t want to drop you. And I’m like okay, you know, like one person one strong guy can like throw me over a shoulder and throw me, you know, like it’s not like that hard I weight 78 pounds, you know, and like they- so I don’t like that. I don’t like the whole like show of like

**I: Making a thing about it.**

F: Yea. Or like I don’t like- yes I mean that’s- those are the two things that like I really don’t like as far as like the clinical aspect of it like the office itself.

**I: Right.**

F: I definitely think those two things- if you’re going to have like a table to lay on it has to be one that goes up and down, and also the reception desk needs to be lowered. Or have, maybe not the whole desk, but have like a section where there’s like the handicap section. I hate that word but that’s what it’s called, you know. Umm or the accessible section where it’s like there’s a dip in the desk and that’s where I can talk to you. But I don’t think all of them have that. At least I haven’t encountered, you know, all of them to have that. Umm...

**I: So umm I know you’ve mentioned some things that you wish certain healthcare providers have that don’t, the sensitivity training and the bedside manners and that kind of stuff.**

F: Yes.

**I: Umm is there anything else that you would add pulling from your good experiences, other things that that make it a positive experience for you?**

F: Well I I think it’s just everything on the flipside where the person listens and the person respects my knowledge of my body and really wants to get to know me. I mean I’ve had nurses that have really been kind to me enough where it made me so comfortable that by the end of my hospital stay I gave them a book that I wrote as like a thank you for for all their care in me, you know. So, I think if you build that relationship and that friendship almost with your patient, it just makes them much more comfortable with you and it it really gives like positive, you know, energy in the room. Like I was in the ICU in, I’m trying to remember because it was during my time where I had four surgeries back to back, I think it was in October or might have been November, I can’t remember. But my ICU nurse was amazing. And I ended up, you know, giving her a book. I was like give this to your granddaughter, like I learned so much about her and she learned about me that I felt comfortable enough to do that when I got out of the ICU. And, you know, she she was just a kind and caring person and I- I know that it’s because of the environment that she’s in- we’re in the ICU so obviously you have to have that. But I just wish that maybe- maybe even the nurses that work in the ICU could train those that don’t to like be more like that, you know, be more kind, and be more gentle with your words and the way that you speak. And like I think that that would have a huge impact if like maybe every nurse had to- had to work in the ICU at least once, you know, to kind of get that feeling of okay this is why I’m here, I’m here to care for people. And whether someone’s in the ICU or not, they still need that same

**I: level**

F: level of decency and respect that I’m giving these people that are like faded, you know, or really like sick, you know.

**I: So, let’s say you go to the doctor, whatever setting, what is that experience like? What do they say to you, what do they ask you to give you that that best experience possible?**

F: Umm I just think when they talk to you while they’re doing things like as far- like it could be in two ways. They could either talk to you and explain to you everything that they’re doing, or like if you- if it’s something I’ve already been through and it’s like routine, if they talk to me and want to like get to know me, and they’re like “so what do you do?” and then I’ll tell them “I’m a disability advocate and I write books and I do this and I do that” and they want to get to know me so it’s, you know, that I find really good because it’s like oh they have an interest in understanding like who I am and what I do. And so and that puts me more at ease if it’s like a new setting but like- like I said if it’s something routine that I’ve done like let’s say it’s I’m going to get dialysis at like another clinic or I’m in the hospital and I’m getting dialysis, if it’s something that I’m used to doing, I don’t need them to tell me like “okay I’m sticking the needle in now” or “I’m doing this”, they know everything that they’re doing. But if they’re like “so what’s your name?” and, you know, “What do you do?” and like, I’m like oh this is cool like I like this environment because they’re friendly and they want to know, you know, who I am, what I do and I think that that’s- just being more personal and more personable is just like really important because umm for me, it it keeps me at ease when I’m in like a new environment. It’s just getting to know- having someone wanting to get to know me and then getting to know that person. Like like my surgeon that I mentioned that did all that craziness, I didn’t know that he has a son with autism until I was like constantly in his office for like four months and then he like opened up and then I was like oh wow you know like I do disability advocacy and like all that stuff so… So it made me feel, even though I’ve known him for so long it took me a while because I don’t see him very often unless there’s a problem like my dialysis graft so the fact that I was continuously seeing him made it easier for us to finally have that openness where we talked about his life, my life, you know, and that like made it more easy to like feel okay to talk about the medical stuff too. Because I’m like oh I know him now, you know. Even after all these years, I didn’t feel like I knew him because I maybe saw him twice before all the craziness that went on, you know, for four five months. So…

**I: Do you feel- do you feel it takes a lot like a lot of time like within, you know one of the things we’ve talked about is limited time of healthcare providers, do you feel like it takes a long time to do that- to build that rapport?**

F: Umm I think it it also depends on the patient. Like I’m a very bubbly, open person. So a lot of the time I come in with good energy even if I am feeling sick I try to come in with good energy. So I think it depends on the patient. Because if you’re in like a positive frame of mind then I feel like whoever is taking care of you is going to be more open to talking to you as opposed if you go in there with like a chip on your shoulder and you’re really rude, then maybe people will be like oh well I don’t really want to take care of this person but it’s my job, you know. Umm and that’ll kind of stop them from wanting to talk to you. So I think it it also has to do with the patient, where you need to kind of build like a a perseverance in yourself where when you’re faced with these medical issues, you still have a positive outcome about life. Where it- when you go to the doctor you’re not like angry and screaming at them all the time, you could just be like calm and be like “hey what’s up?,” you know, “this is what I’m feeling today” or, you know, like... And I think that it goes both ways. Like we have to be, you know, in a positive state of mind and they have to be more compassionate, so it kind of goes both ways. And even- see but I do think that it kind of depends more on them than us in a way because even if you are in a bad mood, the doctor still needs to be compassionate. But I just think that it’s easier to get to a point of umm comfort and comfortability- is that a word (laughs)- to a- if if you’re in a good mood and if you’re positive, I think it’s easier to get to that point if you keep that kind of frame of mind regardless of what you’re going through.

**I: Right.**

F: So I think it goes both ways.

**I: So so let’s say you you go visit a doctor, that doctor doesn’t really know anything or little much about spina bifida like what do you- what would you hope that they would do or how would you hope they handle your visit?**

F: Umm well usually I mean I think it’s crazy when like a doctor will be like “so I see in your chart that you have spina bifida and you’ve had it since birth?”. And I’m like, “yea, it’s a birth- I’m like yea, it’s a birth defect”. And they’re like “oh yea! Yea yea it’s a birth defect, I’ve heard of it before”, you know, I’m like…okayyyy, you know. So then I’ll like- I mean sometimes I’ll tell them the magical tree story that my mom told me when I was four, which I asked her, you know, why do my siblings walk and I don’t? And she goes, well everyone has a magical tree inside of them called the spine and it’s because it’s long and tall. And she goes and it has these magical leaves called nerves that help you move your arms and legs, and you’re missing some of your magical leaves to move your legs. And that’s how I explain it to people. So if you haven’t heard of spina bifida that’s what I’m going to say to you.

**I: (laughs)**

F: Like and like some people think I’m like talking down to them but that’s really the easiest way to like explain it to anybody. I mean I do it when I do my my readings at schools, I do it- so even sometimes I’ll do it to doctors- I’ll be like this is what I was told spina bifida is, you know, but I feel like they they’ve heard of it and they like know what it is, but some may not know that it’s from birth, some may think off-hand that I was in like an accident if they don’t read the chart right away. So I have to like go through, you know, telling them, you know, that no, indeed I do have spina bifida and this is what it is so. And it doesn’t really bother me, it just it kind of... baffles me because I’m like you went to so much medical school how do you not know like every condition under the sun. And some are very well informed and they- depending on their field. Like if I go to a urolo- like a urologist, they’ll know everything about, you know, my urinary pouch and why and the, you know, the bilateral reflux in the kidneys and everything that I went though up until the point of getting, so they understand. So I think it depends on your field of medicine- that like you’ll be more knowledgeable of the condition, but I don’t think that that should be the case. I think- no matter what your field of medicine is, you still should be knowledgeable about every kind of case you could encounter. You know, like I said, my urologist is really really good, he understands everything that I’ve been through. He, you know, explains to me why I can’t revert back to catheterization which I would love to do as an adult and not have a bag, but he’s like, you know, the fact that your bladder is hasn’t been used in 30 years, it’s probably down to like the size of a pea and it just wouldn’t be okay to like put your bladder through all that again 30 years later umm, so that’s- he talks to me and he understands, you know, why things are the way they are. And so that’s good, but my- I hope we get to a point where all doctors are as knowledgeable and not just because they’ve encountered it time and time again.

**I: Because they were trained.**

F: But just because they were trained to know, you know, like even…my cardiologist is good too. He’s more like- he’s more one of those doctors that doesn’t really know maybe too much about my condition as far as my disability, but in his field, he made sure he doesn’t give me prescriptions that are too heavy for me. So he makes sure he treats me in a pediatric way. So there there are doctors that do both. Like one could be completely knowledgeable about the condition, others are just like okay she’s the size of maybe an 8-year-old, so let me treat her like a kid as far as like the prescription that I give her and all that. So it really- it varies. But I do think that knowledge of all disabilities and all conditions is really really prevalent and really important for any doctor, no matter what your field is, you know, umm I mean that’s really all I can say on that.

**I: Umm one of the things umm we’re talking about when we talk about, you know, helping to address the health disparities for patients with disabilities is, you know, well how can you assess something if you’re not measuring it? So one of the questions is, you know, if we were going to assess disability status, you know, would that be okay? How would we do it? So first let me just ask, is that is that something that you feel most people with a disability would be uncomfortable with?**

F: What do you mean by disability status?

**I: So excellent question- that could mean anything. So that’s that’s the main question when we when we’re doing the work that we’re doing we’re kind of exploring, you know, what that might look like to be most useful. So rather than a yes/no question, we’d probably look at um different types of accommodative needs for (inaudible) um so that those could be linked to whatever services or adjustments are needed for that patient.**

F: Yea.

**I: Soo those are just some ideas. So let me actually show you. So this is kind of a starting point of of the conversation- so this right here, these are six questions that actually the census uses to assess disability status.**

F: Uh huh.

**I: Umm and so, there’s not a whole lot out there. So this is kind of like a all we have to begin with. So I just want to show these to you to get your thoughts. So first one is about um sensory difficulties, uh as is the second one- deafness, blindness, impaired sight and/or hearing. Umm then next one is because of physical, mental, or emotional conditions do you have serious difficulties concentrating, remembering, or making decisions?**

F: Uh huh.

**I: Uh do you have serious difficulties walking or climbing stairs?**

F: Uh huh.

**I: And do you have difficulty dressing or bathing? And last one is, because of all these conditions, do you have any difficulty doing any like errands or those those types of, you know, going to the doctor, those activities of daily living- independent living we would say?**

F: Okay so the two that jumped out at me is definitely do you have serious difficult walking or climbing stairs. That to me, I’d be like what’s *serious* difficulty? Like umm, you know, I just think like... does that mean like you need little assistance, a lot of assistance? Umm I know for me, umm I mean obviously I can’t walk at all, as far as climbing stairs though, depending on who I’m around if I’m comfortable enough, I’ll be like can you carry my chair up the stairs? If I’m not comfortable with you, I’ll be like you know what just carry my chair and I’ll hop up the stairs like on my butt, I’ll just scoot up the stair. So it’s it’s more on me in those types of situations- whether or not I feel comfortable around you enough. The other one- but still I think that that needs to be reworded because do you have serious difficulty…

**I: So some type of definition of serious or…?**

F: Yea, Just be like- just flat out be like can you walk?

**I: Mhm.**

F: You know like umm, like or “how much assistance do you need when it comes to walking?”, “Do you use a walker?”, you know like, or “are you in a wheelchair all the time?” Like be more specific because I, at least for me, I think that these questions are a little bit strange. Like are you blind or do you have serious difficulty seeing even when wearing glasses. You could be like, “I see that you’re wearing glasses, is it just for reading, is it just for…?” Like be more umm personal or “do you have a condition? Like are you legally blind? Are you...? Can you tell me more about it?” Like something like that, you know. Or same with deaf. You could be like “Do you use a hearing aid? Can you read lips? Do I need to do sign language? Do you need an interpreter?” Be more personal, ask them more like direct questions as opposed to this because some people might get offended by the wording. Umm also the do you have difficulty dressing or bathing. That to me, I mean just for me because I *can* do all that stuff myself, I might be like but why are you asking me like do you really think I’m like that like incapable of taking care- like I might get offended. Just because I’m very proud of myself and I’m very grateful for my abilities to be able to take care of myself in those respects cause it takes a lot. It’s not easy. But I mean I have to basically do gymnastics to get into the bath because I’m very short- I have a very small bathroom so I have to like swing my body around from my chair to my shower chair- it’s like literally like I could be in the Olympics.

**I: (laughs)**

F: (laughs) It’s crazy so…and even dressing, it takes a lot of rolling around. And it’s a lot of effort. But for someone like me who takes pride in the fact that I can do that, I may get offended that someone was asking that to me. Umm so maybe clarify, you know, just be like umm, see I don’t know how, like is- does this, well first I’m going to ask you like this do you have difficulty dressing or bathing, is that directed to someone in a physical sense? Like can you physically do it, or is it like also directed to people that may have an emotional condition that just like may be depressed and not want to get out of bed? You have to kind of...

**I: So these are directed toward every person, they’re not given to any one in particular. So this is just their way of trying to capture forms forms of disability-**

F: So I guess you could say-

**I: without asking about a certain condition.**

F: So I guess you could say like “Do you need to bathe? If so, do you need my assistance?” like or “Do you need help putting on your gown?” like instead of asking them like “Do you have difficulty dressing or bathing” and turning it into a negative, just be like “Hey, you’re gonna go in surgery now and we need to put the gown on you, do you need help or do I just like stand back and let you do it?” You know, so doing it in a more like personal way as opposed to just being like “You have difficulty doing that? Because if so, then you’re kind of an invalid and I’ll help you”, you know. (laughs)

**I: So maybe if this is behind the scenes or something that is like a checkbox, not reading it or saying this specifically**

F: Yea, yea

**I: but engaging in conversation, collecting information, and then…**

F: Yea. Just be like “Hey, you know, we’re going to take you down to X-rays and you haven’t bathed today. Do you want me to bathe you before you go or do you want to do it yourself?”, you know, like something more like engaging as opposed to like putting a person down and just automatically being like “Do you have difficulty doing this?” Like, you know. To me, I would be offended. I don’t know if everybody is like me but because I’m very like “yea! I can do it” I’m like

**I: Don’t tell me I can’t, right?**

F: Yea. I’d be like “excuse me, do I look like I can’t bathe, do I look like I can’t get dressed?” you know like, I would be offended. (Phone rings)

**I: Do you need to take that or...?**

F: No. Let me just see who it was, it might have been my mom... I’ll call her later.

**I: Umm I know we’re getting close to the hour mark so I don’t want to take up too much time. This has been fantastic by the way so thank you.**

F: (laughs) Thank you.

**I: Umm so I mean any other thoughts? I mean, so we have a sense of, you know, make it more personal so that sounds like more like conversational with the healthcare provider**

F: Yea.

**I: As opposed to a patient filling out a form it sounds like**

F: Umm yea, yea. I think it would be good for someone to come in and maybe ask these questions in a more conversational way umm just for me, I mean I feel like that would be a better way to handle it as opposed- because some of these I just think are like rudely written. I mean it’s just it’s my personal opinion. Umm I’m like I try to at least in my writing, I try to be as nice as possible.

**I: Mhm. So so question here, you know, whether if these questions are better questions that are trying are trying to get the same thing. Like are there any other things that you would be- you would want to be asked in a conversation to capture needs that you want them to know that you have?**

F: Umm, amazing.

**I: Like for example, I guess, would something like like number four umm like what would capture the adjustable table or something like that for example.**

F: Umm maybe like “Do you need assistance with transporting onto the table or are you okay, like can you do it independently?” Something, something like that and then it can bring up the topic like normally I *can* do it myself, but this table just happens to be a little too high so I am going to need a little assistance. I will need someone to hold my legs and kind of push me up while I push myself, you know, propel myself up. So-

**I: Right.**

F: And then you can explain to them, you know, that kind of stuff. So yea like something like that in regards to the changing table like, you know, “do you need help with the transfer or are you good?”, you know. I mean that would be a good question to ask because a lot of us may need some help, you know. Umm...

**I: So any other things that you would want it to capture that it’s not?**

F: No, no. I just do think that these need to be written in a more personal and almost less offensive way. Umm I I think, you know, a lot of the time people- people can take offense especially if they’re very- umm... they can take offense either way, whether they’re prideful of where they are with their condition or if they’re insecure about their condition. So you have to be sensitive either way. Because there’s people like me that’s like I am alpha woman and I am, you know, strong and I can do it and I’m cool, duh duh duh. And then there’s people that may be new to their condition or may not have accepted their condition even from birth and they’re like oh well you know, this makes me uncomfortable that this person is saying this to me. So I think it- you have to be sensitive either way and that’s why I think that these need to be, you know, a little more nicely written.

**I: More positive outcomes or perspectives**

F: Yea more positive questions

**I: More capable versus incapable**

F: Yea

**I: Alright. So that was my my last question other than, you know, we talked about format being more conversational. Like how often should this be assessed? How often- I mean once you’ve done it with one healthcare provider, you don’t need to do it again?**

F: I feel like it should just be one and done. I mean basically if you keep going to the same doctor continuously, then they just need to know like what you can and can’t do. Unless your condition, god forbid, has gotten worse then maybe they can ask it again like “okay you just broke your foot and I see you have a cast on, do you need more help than last time?”, you know like something- if you see that there’s a condition change then maybe maybe ask the question. But if it’s the same person and they’re in the same, you know, physical state or whatever then I don’t think you need to keep asking. But if there’s differences within that person then maybe you can ask it again.

**I: Alright. That’s it. Any other last words of wisdom for healthcare providers that you have not yet addressed that you want to get out?**

F: (laughs) No I think I got it all. I hope whoever listens to this doesn’t think I’m a mean person.

**I: (laughs) No, not at all. Not at all.**
