# Supplementary material for: Disability disclosure in healthcare settings for individuals with developmental disabilities: A qualitative study of patient and caregiver perspectives
Source: PLoS One. 2025 Aug 7;20(8):e0329328. doi: 10.1371/journal.pone.0329328 (PMC12331114; doi:10.1371/journal.pone.0329328)
Supplement: S1 File — (ZIP) [file pone.0329328.s001.zip › Transcripts/2019.08.26 Interview 07 Transcript.docx]

***2019.08.26 Interview 07 part 1.mp3***

| SPEAKER1 | 00:00 | All right , hit record . So just for the record , we're OK with you consented to to participate and you're OK with the recording . Yes . All right . So I wanted to just get in a little bit more in depth with regards to health care experiences , good and bad . So so would you say overall , you and your sister's health care experiences have been mostly good , mostly bad , mostly bad . OK . Have you had any good experiences as well ? Or I can probably compliment a few . Yes . Just OK , so unless this jump into the bad experiences . So you tell me a little bit , you know , just , you know , specific examples and what made them bad . Will there be something about the actual doctor or health care providers ? |
| --- | --- | --- |
| SPEAKER2 | 00:45 | The facility , anything at all is basically the facilities because they not prepared to facilitate an interpreter for my sister . And most facilities , if she does , if she doesn't bring a family member that can translate or can sign for her , they basically don't have one or one accommodate . So so basically , I need to make sure that I that I assist with her or my mom or my brother , somebody has to be available to help her . And I don't think I mean , she's old enough to go by herself , but she depends on us . |
| SPEAKER3 | 01:32 | Right . So you've never encountered a setting where they actually were prepared to to be able to translate or interpret what ? OK , and that's what settings this specifically is that primary care , primary care , the urgent care . |
| SPEAKER4 | 01:49 | Any hospital experiences as well as hospitals ? No , no , no . OK , we'll see for her appointments , which would be for her eyesight or hearing . Specialist , you know , right . |
| SPEAKER3 | 02:03 | And has she or anyone in the family taking any , like , into anticipatory actions , knowing that that's problematic , to let them know ahead of time , to let them know ahead of time ? |
| SPEAKER4 | 02:16 | But most officers don't call an interpreter and have them readily available , you know , like they always tell the world somebody from the family needs to come in . You know , that way she can understand what the doctors say . |
| SPEAKER3 | 02:31 | So they don't they just instead of offering it , they say , hey , you need to make accommodations , OK ? Mm hmm . |
| SPEAKER1 | 02:39 | So any any other things beyond that that also kind of make it a bad experience ? |
| SPEAKER5 | 02:47 | No state is is that so bad and and I think that we are not clinically prepared to die , for example , I can interpret something for her but is not clinical , right . |
| SPEAKER3 | 03:03 | You might have I might not know . |
| SPEAKER6 | 03:06 | This are the signs for like medical term saying so I can explain it to her or my mom and explain it to her to the best of what are the best way possible . |
| SPEAKER3 | 03:18 | But it is not medically , you know , so you have concerns that you might not be getting at all or in the way that it needs to be different to understand it correctly . Yes . Have you encountered any any issues with that where you find that maybe understanding or the translation was , you know , not what it needed to be and then maybe the services or the treatment weren't as they as intended ? |
| SPEAKER7 | 03:45 | I usually have to explain it step by step so she can understand me , like , for example , instead of like using the word the medical word , I have to explain it to her what it is that makes sense . |
| SPEAKER3 | 04:01 | And would you say , you know , one of the things we talk about in these conversations have been like issues with dignity and respect . |
| SPEAKER1 | 04:09 | Do you feel that the she feels supported and respected by her health care providers ? OK . Tell me more about that . |
| SPEAKER4 | 04:19 | And I support it because she doesn't feel that they're giving her the time . |
| SPEAKER7 | 04:30 | The doctor , I mean , the times that they were the doctor talks to me and not to her because he relies on me letting her know or translating it for her . So I don't think she feels that , OK , it's me . |
| SPEAKER3 | 04:46 | They're talking to me like she's being ignored . And she's like a third wheel , you know , she's the main person . Exactly . |
| SPEAKER1 | 04:55 | And how about , you know , in terms of time , like actual or reaction to the need for someone to translate ? Like how how is the rapport as far as like them allowing the necessary time to to have the information deliver to you , to translate to her ? Are they are they patient ? They give the extra time ? Do they make you feel rushed ? How's that ? Well , it's always rush . Would you say more or less . So then then like if you went for a checkup yourselves for more . Yes , more so . Mm hmm . |
| SPEAKER3 | 05:31 | And any other things about each other ? Well , I suppose , you know , are there any other actions that you take to anticipate ? I mean , obviously , you said that you or someone in the in the family needs to accompany her to make sure that she gets all the information she needs . Anything else you do ? |
| SPEAKER4 | 05:47 | We have even called like a week before to let them know that my sister is dead in case they don't know , in case , you know , even though most of the appointments are not her first appointment , you know , she's out and you are a new patient . But we also let them know a week before we actually call and let them know . But they just always tell us that they don't make those type of accommodations , that if they call for a translator , it takes longer or they I mean , it's just it's always kind of like a game , like they always say , yes , we'll call one and then I have to call again . And then they say , yes , no , I couldn't reach one . |
| SPEAKER3 | 06:30 | And so would you say that they they have the ability in-house , they have that resource and it's just them not using it because it's not convenient is I think they could , because for my knowledge , they have like interpreters . |
| SPEAKER5 | 06:46 | Florida , like the they do have interpreters . |
| SPEAKER1 | 06:50 | I just don't think they use them , you know . So you think where do you think the breakdown is ? You think that it's more as best as you know , is it them not putting in the requests in a timely manner , not having enough resources so that they can stuff everything that they need to have any ? |
| SPEAKER7 | 07:08 | I just don't think they I just don't think they . They . They understand the necessity of it . So it's not an urgent matter for them . No , it's not . |
| SPEAKER8 | 07:24 | Creepy . |
| SPEAKER3 | 07:30 | So I suppose you know what , if you had to say what you would do to to make it a more pleasant experience , you know , obviously making sure that the services provided . Was there anything else you you would hope for that would make the experience better for ? |
| SPEAKER2 | 07:53 | It is basically for my sister and annoying her . |
| SPEAKER4 | 07:58 | I think that just by having an interpreter , she will feel like one person , you know , like she will feel more independent and she doesn't need to have one of us there with an interpreter . |
| SPEAKER5 | 08:13 | I think that she will have . |
| SPEAKER4 | 08:16 | A lot more feedback to the doctor and not depend on us , you know , because like I mentioned , we don't I don't . I know I know which . But I don't know the medical terms . I don't . |
| SPEAKER2 | 08:30 | And it's hard to explain it to her and it's hard to explain to the doctor what she's saying . So I think that with an interpreter , I think that will make her life easier , a lot easier in the sense of , OK . She already knows and she'll feel comfortable that we're confident that there's going to be somebody there to , you know , provide that for her . |
| SPEAKER9 | 08:49 | I think that will make a difference for her . |
| SPEAKER3 | 08:51 | And you do know to the best you're really there's ever been any concerns on her and about just like privacy , like maybe she doesn't want to be your brother goes with her and she's like , I don't want to . I don't want to . Yes , yes , of course , yes , because so did you think that she's ever , like , held back ? No . Sure things because of that her privacy ? Definitely . Yes . Yes . |
| SPEAKER10 | 09:20 | I'm just trying down notes so I can make sense to you . Thank you . |
| SPEAKER1 | 09:26 | So . |
| SPEAKER3 | 09:26 | So when when the health care providers do interact with her directly rather than the whoever is there as a caregiver , is there anything different that you would recommend that they do in that sense , again , to make the experience better for ? Someone I'm sorry , just anything in general , you know , obviously you said before it would be helpful if if they spoke directly to her rather than the cafeteria staff there . But anything else that comes to mind that they might not be doing that they could be doing better for the quality of her health care experience ? |
| SPEAKER11 | 10:02 | I believe maybe the the . |
| SPEAKER4 | 10:07 | Having more patience in the sense of communicating the message , you know , like . |
| SPEAKER2 | 10:16 | Taking the time to explain it a little bit better . |
| SPEAKER4 | 10:22 | Just because , you know , my sister is not like for example , she's an incredible world , you know , anyway , for me as a regular patient , when a doctor comes and tells me a bunch of things he like , you get confused and you're like , OK , is that good or bad ? |
| SPEAKER6 | 10:39 | Is that , you know ? And it takes you a while to kind of like assimilate and think , OK , it's OK . |
| SPEAKER7 | 10:48 | Just taking the time to explain and letting them know . And I mean , it's not just learning , OK , you're fine explaining to them this is what you gotta do . And taking the time . Taking the time is to . |
| SPEAKER3 | 11:02 | And have they ever done or taken the time to maybe write things out for her , the would that be helpful ? That would be , yes . OK , so communicate especially those medical terms . Yes . Or she has to T.Y. , OK ? Yes . She has used those before . And is that something that they provide if they had it or she brings with her or if they have it , they should provide it . |
| SPEAKER7 | 11:28 | She doesn't bring it . |
| SPEAKER3 | 11:31 | It's like a typewriter crisis . It's not exactly , but I agree with you . OK . So so so you do pretty much always let them know ahead of time that she's deaf and try to make those arrangements . Is there any other time when they didn't know and they kind of had to figure it out for themselves or should it come this way now ? |
| SPEAKER4 | 12:01 | She was always a nation , essentially , always lets me know so I can come . |
| SPEAKER3 | 12:04 | Right . And does she have or do you have any concerns about them knowing in the sense that they might treat her differently because they know ? |
| SPEAKER12 | 12:15 | They usually do . They treated it . Yeah , how how so tell me about that in the center of . |
| SPEAKER7 | 12:23 | They trigger different the and not in a good way . |
| SPEAKER13 | 12:29 | It's more as of like . What is it ? What's the word I'm looking for ? |
| SPEAKER3 | 12:36 | Kind of like them making them work double , you know , like you're overburdened and yet you're an inconvenience . Yes , an inconvenience . And do you think that that is more a result of , like , personal pursuit ? I mean , as much as you can tell , we're just speculating here . But their personal perceptions or do you think it's you know , I just you know , I'm limited on a policy level , limited with how much time I have . And I just , you know , I got to get in and out . I go see people fast . What do you think the is the reason behind ? I think it's because they go global and they don't take the time to some more like the policy or like . |
| SPEAKER6 | 13:14 | So instead of the system , the way that the the office is set up , you know , the way that they their work flow . |
| SPEAKER3 | 13:22 | Right . So you get a sense that if the system changed , perhaps they would be less inconvenienced , so to speak . |
| SPEAKER4 | 13:29 | Yeah . And they're more educated on it . |
| SPEAKER10 | 13:32 | Yeah , I think so . Mm hmm . |
| SPEAKER3 | 13:36 | So obviously the the work that we do outside of this interview is talking about how do we kind of identify people with various types of disabilities for the sake of being able to understand it and link it to whether health care is being done in a good or bad way . So what do you think or what do you think your sister thinks about documenting this officially in a medical record ? Do you think she would be fine with that ? Oh , yes . Yeah , yes . OK , no , no concerns or that . |
| SPEAKER4 | 14:07 | No , considering she might be treated differently or know it will make it will make it a lot easier for her . |
| SPEAKER3 | 14:15 | And in that case , what what do you think might be the best way to do that is that , you know , in , you know , filling out an intake form or being asked when communicating with your intake form . Yes . And do you think that it should just be , you know , a question of balance ? So let me give you an example and of course , you can think about this specifically to her or disability in general as well . I'm sure you've seen this before . Right . |
| SPEAKER14 | 14:45 | But the questions from the ex , the census questions , you know , this would be a starting point for conversation , not necessarily the right answer , but the first question , are you deaf or do you have serious difficulty hearing ? |
| SPEAKER6 | 14:59 | Is that something that she will say , OK ? Yes , of course . |
| SPEAKER3 | 15:03 | And do you feel like that wording is appropriate ? Would you recommend any changes to it to to grasp or to capture whatever she wants to for my sister ? I think this is fine . So that's fine . Right . OK . And would you want them to ask anything else , like , you know , as far as you know , OK , you said yes to this . Now , what does that mean as far as what you might need from us to better serve you ? Is there any you need any assistance ? Do you need any assistance ? OK . |
| SPEAKER7 | 15:32 | So just general like that , or do you need an interpreter ? Do you need any assistance for your visit today ? |
| SPEAKER3 | 15:42 | So specifically an interpreter , would you add anything else , like if you had a list of things that you would want beyond that and the other resources you'd mentioned , for example , the T.Y. before , would you want that as an option in addition to now that you have video chatting for ? |
| SPEAKER12 | 16:00 | I seen an not in the medical field , and I seen it in a video chatting , what's it like ? It is like Skype , OK ? |
| SPEAKER2 | 16:11 | And she's able to communicate with them and they communicate with . OK , I mean , but I don't think it's I don't think she's able to use that in a medical setting because it's for privacy . Oh , OK . |
| SPEAKER3 | 16:27 | Yeah , because it's hard to maintain privacy . Right . To privacy might be an issue there . |
| SPEAKER1 | 16:33 | Anything else you could think of , any other resources that would be beneficial that she would be able to say yes or no to if asked for and maybe in a way to . |
| SPEAKER4 | 16:51 | We have tried to T.Y. , we have tried . I don't know some way of messaging , maybe by writing . |
| SPEAKER3 | 17:09 | If a person is not there , you know , like in any family there , she can ride it as well , you know , so you would want or expect that these resources would be able to be available and be available in a sense that you would feel comfortable her going by herself . Yes . Yes . OK . |
| SPEAKER15 | 17:29 | All right . And you would say , see here . |
| SPEAKER3 | 17:37 | OK , so you prefer the intake form versus just being asked ? OK . And do you think it's their responsibility to ask that question or the patient's responsibility to make sure that they know they need to ask that question ? I think that those are all the questions that I have for you and the other last thoughts about , you know , anything that would improve the experience unless either actions that you can take , she can take or that the health care system needs to be taking . |
| SPEAKER2 | 18:19 | I think that once her CHA or anywhere is noted that she is deaf or that she needs assistance , it will be good for them to be proactive and just , you know , do it even before we call . |
| SPEAKER3 | 18:37 | As soon as they know they know they shouldn't they should make it . |
| SPEAKER2 | 18:41 | I don't know something to alert them that he is . |
| SPEAKER9 | 18:45 | I'm sure the deaf community , they may go through that . |
| SPEAKER2 | 18:48 | I mean , and I have heard that in other states , they get interpreters right away for every single thing she does . She used to live in New York and I know that it was available . So I know that it could be possible because you seen say yes or and that's health care setting only or is everywhere . Everywhere , everywhere . They have the . |
| SPEAKER3 | 19:12 | You just call one number and the interpreter comes right away and is that , you know , they would be the ones calling and they cover the costs . Yes . OK , so I'll be OK . |
| SPEAKER4 | 19:25 | So I'm thinking here , maybe that's the issue . The cost . |
| SPEAKER15 | 19:29 | So might be . It might be , yeah . Who funds the service . Right . Have you ever received that service and been charged for it . No . I believe that in New York , maybe the government . The state government . |
| SPEAKER3 | 19:49 | Maybe we don't have those resources here , right , and I guess one other question that kind of came up in my mind is when we talk about continuity of care , let's say you were I don't know if this has been the case . So has your sister ever been in a situation where she had multiple health care providers , even between MDs and Nurse ? And if you have a team of health care providers , how have you experienced the communication between them as far as sharing your sister's needs and , you know , someone new coming in kind of already knowing and and being prepared to deal with ? I have to say it again . So you have to keep on so . So no communication between . |
| SPEAKER15 | 20:35 | Why do you think that that's something that they should be shown to be ? Yes , I'm not kind of dependent on the person there with her . Right . For me , last thoughts . |
| SPEAKER3 | 20:51 | Is said at all . I said it all right . It's just a good . |
