# Supplementary material for: Disability disclosure in healthcare settings for individuals with developmental disabilities: A qualitative study of patient and caregiver perspectives
Source: PLoS One. 2025 Aug 7;20(8):e0329328. doi: 10.1371/journal.pone.0329328 (PMC12331114; doi:10.1371/journal.pone.0329328)
Supplement: S1 File — (ZIP) [file pone.0329328.s001.zip › Transcripts/2019.08.26 Interview 08 Transcript.docx]

**I: Interviewer, P: Interviewee Participant, C: Caregiver**

1. I – So I am putting on the tape recorder and just for the record I am just confirming that you are ok do you consent to being recorded and participating?
2. P – Yes I do.
3. I – Ok thank you. So, First off just let me ask in general if you think about the healthcare that you have received in whatever settings would you say that you had good experiences?
4. P – Good in the sense the attention was good, healthcare is so expensive.
5. I – Uh huh and have you had bad experiences?
6. P – not really
7. I – ok ok
8. P – it is just the question of affordability right
9. I – ok so tell me more about that then
10. P – im lucky because I am working. I have healthcare. When I first came to this country, I was not ah ehhhh, I was just a student, and you know school insurance is not that great, so to get outside insurance I could not afford it
11. I- Ok. And so tell me about you … what settings have you received healthcare in, just so we are clear?
12. P – as a student I received, then when I I did apply for asylum, based on hardship so, in that setting I had good healthcare because I qualified for Medicaid
13. I - ok
14. P - so everything was practically free
15. I - mhm
16. P - so that was fantastic
17. I - right
18. P - but the biggest problem I think in this country is healthcare, affording healthcare.
19. I – so access through costs specifically
20. P – Yes. and it’s so scary to think that and uhhh depending on the politician that pre existing conditions may not be covered, that’s a nightmare. The thought is quite a nightmare. Because my argument is that I could easily have a problem that is not even connected to my disabilities, and you’re not going to cover me? When I first came to this country, they would come and, all these insurance agencies would come and ask me questions. They would ask me, Do you have cancer? Do you have a heart problem? And Do you smoke? Those are the three standard questions. But they had no clue about spina bifida, they had no clue about my disability.
21. I – so they didn’t ask about it?
22. P – nothing. And, oh sorry you don’t qualify. And the biggest problem I have with healthcare when you talk of that umm assisted devices. Chair, I have to pay out of pocket for the most part. That’s my problem with it.
23. I – Sure. And if you don’t mind giving me a ballpark, what would something like that cost?
24. P – this chair is 47,000, I have another chair that is just 50. So those are the types of wheelchairs we have to use.
25. I – Yea, absolutely, to get around. Okay.
26. I – ok so ya, so obviously you say access as a result of cost is an element. So tell me about the actual, your actual healthcare experiences in terms of interactions with the healthcare providers, anything, whether it be a doctor, a nurse, front desk staff. Overall, how has that experience gone?
27. P – overall it has been fantastic. For the most part. Here I go to [hospital]. Because my doctors are there. The only thing I have experienced with the insurance I have now, they say for the specialist is 120, so what they have done is they have considered my primary as a specialist because he is an oncologist, but what I tried to explain to them is he is internal medicine also, so that is why he is my primary, but they don’t get it but so they still keep charging me 120 for it. So that is another issue.
28. I – right, the billing aspect
29. P – the billing aspect of it. but as far as getting treatment and all goes, it has been fine, especially here
30. I – so tell me then how did you come to find and go to the doctors that you see?
31. P – ok the doctor I am going to now, happened to be a friend of mine, in Oklahoma he treated me, where I was before this, and he happened to move to Miami, so I just continued with him
32. I – and how would you say his awareness of spina bifida is?
33. P – very good
34. I – and did he, was it very good before he met you or did he come to learn more?
35. P – I think he knew
36. I – he knew
37. P - he seemed pretty knowledgeable
38. I – mhm. And do you have any insight as far as how he became knowledgeable? Whether he had training in school, or whether was he self-taught, or whether a patient might have taught him?
39. P – I think it’s just probably his training in school. but one bad experience I had was in Oklahoma, because at that time I had bladder incontinence so I went to this doctor and I said what can I do about this. he just turned around and said, there is nothing that can be done, and then it turned out that I came to hear of a surgery in India, and I went to India and got it done and now I don’t have any bladder incontinence.
40. I – so . . .
41. P – and . . sorry? (was interrupted)
42. I – no please go ahead
43. P - And another thing is when I came to hear of this surgery the doctor in India told me you go back and you find out whether you want to do this because it is a very painful surgery and you cannot reverse it. I came and asked several doctors and they all discouraged me from doing it and they thought I was crazy to do it, but I just went against the odds.
44. I – so there was a chance that it might have been painful
45. P – it was very painful, but they made it seem like I won’t have good aftercare, and I won’t be able to prevent infection, I don’t think they were knowledgeable and didn’t give a positive outcome
46. I – so talk to me more about when you say I don’t think they were knowledgeable. Like how?
47. P – Because uhh many doctors didn’t seem to know about that particular surgery when I came back and asked them
48. I – so it wasn’t like they were familiar with it and thought it was a bad idea, they actually probably didn’t even know
49. P – even to this day they think it is a bad idea in the US, it may have been because it is a big liability issue in America, unlike in my country, so people won’t hesitate to do it, but here they are so scared of malpractice, but it was not a very good experience
50. I – so are you seeing any other doctors besides the internal medicine doctor
51. P – no nobody
52. I – so that’s your primary care you would say, at [hospital] and tell me what makes it a positive experience for you, what specific things does he do or say that makes it a positive experience?
53. P – the whole care team is very caring, and attention to detail, and umm they have your interest at heart I think. especially [hospital] is really fantastic.
54. I – so tell me more, you say care team, so who else is interacting with you
55. P – for example if I get admitted at hospital, four or five doctors come and I’ve kept in touch with some of them, like a podiatrist and umm this doctors assistant, yeah I have kept in touch with all of them
56. I – would you say they are all as knowledgeable about spina bifida as he is
57. P – I don’t know. I guess so because some of the things that I got admitted for were related to the.… but some were not
58. I – ok, umm and umm
59. P – the biggest problem is going to be in America is if I need surgery, it’s too expensive I cannot afford it. I can get it done for half the amount in India or less
60. I – so so do u find that when they see you or find out that you have spina bifida that you are treated differently, do you ever feel like you are treated differently?
61. P – no
62. I – ok ok so they always are respectful and all that stuff, is there anything in terms of the physical environment that makes it a good or bad experience when you go to the healthcare provider?
63. P – I think the welcoming environment
64. I – ok. so tell me more about what makes it welcoming, specifically
65. P – people friendly and and attentive
66. I – mhm ok, so are there any physical barriers or anything in the environment or everything is easy to navigate
67. P – easy to navigate
68. I – would you say the equipment that they use is appropriate for you whether it be adjustable or modifications they have all that for you as well
69. P – yeah
70. I – like adjustable tables, or . . . what about services, do you find that they have any and all services that you need, whether it be assistance with transfers or anything like that?
71. P – ya they always ask, they always ask what I need, because unless I say something they are not gonna know what my specific need is. because each one is different
72. I – so how do they ask you? is it informal, formal, verbal, nonverbal, paperwork?
73. P – no, for example if I, as u said transfer, if I need assistance theyv’e always asked me let me know to ask or maybe if I need change of diaper they do without hesitating, they would volunteer, the nurses or the nurses aid
74. I – ok ok, and do you feel like they give you enough time ummm and attention with regards to all your needs?
75. P – for my follow-ups, yes.
76. I – and have you ever felt that umm that you might have, like you said, that you might go for a concern that may be related to having spina bifida or not, do you ever feel like they might misunderstand your needs based on having spina bifida or they might perceive your risk as more elevated than it should be or maybe underestimate it?
77. P – I think the ones in Oklahoma the ones I told you about the doctors in Oklahoma as far as the bladder, when I felt I was not heard
78. I – mhm
79. P – they just brushed me off I felt, with what they had to say they didn’t look into it and say ok let me check this I am not familiar let me check and get back to you. No, it just cannot be done
80. I - so you think that was more of a they didn’t even bother to look at the research
81. P – and I lost a few years because of that to get this done,
82. I – that was few years dealing with an issue that you didn’t necessarily have to if you gotten the treatment
83. P – Yea, because I had the incontinence (inaudible)
84. I - mhm
85. P – so for thirty years I had it
86. I – mhm, and so so you said that they just ask you so there has never been any formal or written method that theyv’e used to ask you what needs you might have or anything about having spina bifida?
87. P – no, but they have done surveys about how the visits were
88. I – oh, like a satisfaction survey?
89. P – but specifically spina bifida, no
90. I – um so so one of the things we are exploring cause we know in general there are health disparities with patients with disabilities relative to patients without disabilities. Where patients with disabilities have less satisfactory healthcare experiences overall. um so you know in the process of trying to address that one of the things we are talking of doing is possible collecting information about disability status when someone comes to a healthcare appointment
91. P - So is this all types of disabilities because I know this is specific to developmental.
92. I - So this is, this is actually all different types of disabilities we are interested in the gamut. So let me show you actually.
93. P - Because I understood it for only for developmental
94. I - That’s definitely part of it. we that’s initially one of our main focuses but since at the same time since anyone can be a patient and anyone can walk through the door I think it is important that we’re looking inclusive as well, all types
95. P - And many people misunderstand they don’t realize spina bifida as developmental, that’s what I realized
96. I - Mhm right ya absolutely i’ve gathered that as well just by chatting with people. Um so if we were going to collect information about disability status, the question is, how would we do it in a useful way, so that we can use that information to evaluate health outcomes uh but also when someone is receiving care, better understand what needs someone may have. Since like you said everyone is different and everyone has different needs. So that’s the question we’re trying to answer and I wanted to kinda get your thoughts. So I don’t have an answer right now, I want to hear more from you. but as a starting point, these six questions are questions that are used on the for the US census to collect information about disability status
97. P - Do you want me to answer them?
98. I – Or just take a look at them ya. So the first two are more sensory, like deafness or blindness or vision impairments
99. P - OK I can answer them if you want
100. I - Sure absolutely go for it
101. P - So you want me to write it or tell you?
102. I - How about I read it and you tell me?
103. P – Ok
104. I - Are you deaf and do you have serious difficulty hearing?
105. P – No
106. I - Are you blind or do you have serious difficulty seeing, even when wearing glasses?
107. P – No
108. I - Because of a physical, mental, or emotional condition do you have serious trouble concentrating or making decisions?
109. P – No
110. I - Do you have serious difficulty walking or climbing stairs?
111. P - Yes
112. I - Do you have difficulty dressing or bathing?
113. P – No
114. I - Because of physical, mental, or emotional condition do you have difficulty doing errands alone such as visiting a doctors office or shopping?
115. P - That depends on what you mean by that because.. it could be a mobility thing issue.
116. I – mhm, if its so ok if
117. P - it depends on accessibility, yes, it could be a physical barrier
118. I – Right, so you’re saying so only if it’s in relation to a physical barrier otherwise your fine
119. P – otherwise I’m fine
120. I - ok. So having gone through those I mean how do you feel, obviously this is just one way that’s been used in the past in general, not specific to the healthcare setting. So what are your thoughts about those questions? Do you find them useful in sharing whatever information that you want to share with a healthcare provider?
121. P - Yes. I’m just trying to remember whether you’ve if you covered all the disabilities in this questionare.
122. I – Right, definitely, that’s one of the questions that we have. some folks might say that we’re missing some stuff there
123. P – (inaudible) For the diversity of it, you’be got, that’s the only thing I have.
124. I - Mhm
125. P - And youv’e said mental also, emotional and mental. So that should cover it but emotional and mental is a broad area.
126. I – Right
127. P – but its not possible to cover specific. so I think it’s pretty good.
128. I - So the wording is OK
129. P – it’s fine
130. I - there’s nothing that is confusing or offensive
131. P - It’s quite sensitive it’s not insensitive, the language
132. I - Ok sensitive ok ok
133. P - And it’s politically correct, right, that’s the most important
134. I – So would you.. in thinking about if you were going to be asked questions or you’re disability status assessed, would you prefer something more specific like this or would you perfer more general question? how would you like to be asked?
135. P – Specific
136. I - Specific ok.
137. P - Because Close ended questions are better. With open ended you can go on and on and on and it becomes too General, too broad.
138. I - And is there any additional question or more nuance that you would want to be asked to make sure that they capture information that is helpful for you to share with them?
139. P - . . . (very long pause) I don’t know if this is correct but as a person with a disability, what are some of the needs you will have more than a person without a disability? Specifically, what are some of the needs to access healthcare. Because definitely having a disability you have more health issues.
140. I – Mhm. So tell me if that was a question you were asked how would you answer it for a healthcare provider?
141. P - I would first talk about affordability. And to be able to access equal healthcare as a person with a non disability. For the simple reason I was talking about pre-existing conditions. Why should I be treated differently. Because there are many illnesses I can have that have nothing to do with my disability.
142. I - So that’s more so from the kind of insurance standpoint then the direct care you receive once you have insurance
143. P - Yes. And also, as I told you some doctors were not knowledgeable about the specific need that I had. or concerned.
144. I - And how did you get the sense that they were not knowledgeable?
145. P - When I asked them what can be done, they said nothing. That’s a very vague answer. It’s like asking a lay person or a layperson answering me and saying oh nothing. I would think that at least you would try to find out something even if you did not know. I felt that I was just shut down
146. I - So do you think that, we talked a little bit about maybe they didn’t have the information about that particular type of surgery. Um but do you feel
147. P – But at that time I didn’t know about the surgery, so I was asking in general
148. I – Ok. So but do you feel like maybe they didn’t know about that particular surgery or. Or different things that could be done but do you think that they had a basic understanding of what spina bifida is?
149. P - I don’t know. in their case, I felt they didn’t
150. I – Right. Just maybe, I mean through, anything in particular like how they were interacting with you or what they seemed to suggest that they knew or lacked in knowledge
151. P - Yes I thought they lacked in knowledge
152. I – Um so anything else you can think of that you would add to being asked needs about needs to when you go to the healthcare provider needs you might have
153. P - . . . For example how do I prevent if I was to get a bed soak, how was I to, I’ve had that a couple times. And I would like to know, of course I know I have to use a cushion and as I am aware I have to get it treated, but how do I prevent something like that happening in future
154. I - Right
155. P - Because if I got an adequate answer it wouldn’t be occurring again, right. so things like that
156. I - Mhm
157. P - So there there was a bit of lack of knowledge there.
158. I – Mhm. And do you always . . .
159. P- And
160. I – Sorry, go ahead.
161. P- And people in wheelchairs are prone to that.
162. I – Mhm.
163. P - I’ve been able to control it, but still.
164. I - Right. When you do go to the healthcare provider, do you tend to go alone or with someone?
165. p – I go alone. Now that she’s here she comes with me but usually um I was living alone, my mom used to come visiting from India, but I stayed alone.
166. I - And do you find out that when she’s with you, the interaction of the dynamic changes at all.
167. P - OK this is one thing I do remember now. Sometimes I’m not talking about the healthcare provider I have now but sometimes doctors if I go with anyone else they tend to talk to that person or they talk to me slowly
168. I – Slowly, how do you mean?
169. P - How.... are.... you. Like I cannot comprehend and I’m thinking in my mind that’s a misconception, you think I have a physical and mental disability here but it is only physical
170. I - OK so they are making an assumption about your intellectual capacity
171. P - And I would think as a healthcare provider you would be more ......that insensitivity is there, sometime.
172. I - Ok
173. P - And talking to another person when I’m right there
174. I - So like asking your mom how you’re doing even though you’re right next to her
175. P - Ya. It’s happened to me before
176. I - Ok ok
177. P - And another thing is a couple times I got cellulitis so I would ask them what’s the cause of it. “oh it’s your age and it’s your disability, and the fact that you have diabetes”. I would get the standard answer
178. I - So you think that sometimes things would be attributed to spina bifida even though they probably weren’t
179. P - Probably not. I’m sure anybody will get cellulitis right? It not that. It’s like you don’t have an answer but you’re giving me an answer
180. I – Like a package, yea. Do you ever feel like um any healthcare, like you ever denied any type of healthcare or attention because of your disability?
181. P – No
182. I - So you have generally been asked questions for example some people with disabilities especially those that are with mobility difficulties will say that they’re not often asked about their sexual health in screenings for example, so that’s not been your experience?
183. P – (seems to have nodded no)
184. I – Ok, mhm. So tell me if you were to go to your doctor, like tell me, just walk me through the experiences as far as what would be the perfect best highest quality experience assuming affordability, starting with that and then going from there.
185. P - I think that the best thing would be giving solutions like for if something it’s a chronic problem and it keeps occurring over and over again, giving me a solution right there. I think the magical answer would be just telling me giving me answers right away instead of hesitating. At the moment, the care that I’m getting couldn’t get better.
186. I – Ok.
187. P - But from past experiences it has not always been this high-quality experience. Maybe it’s because it’s [hospital] and it’s supposed to be one of the best. But I don’t know whether it’s because of that would it be any different at any other hospital. Something like [hospital 3] or [hospital 4] or something.
188. I – Right right
189. P – Like [hospital 2] also though it is a public hospital, the attention is good.
190. I - So yeah tell me more about the attention, you said attentiveness was an important part of what made your experiences positive. So what, how were they demonstrating the attentiveness?
191. P - Asking me questions. Asking me if I had questions. Making time for me having the time of the day for me. And focusing only on me.
192. I – Ok.
193. P – Because sometimes doctors can just brush you off.
194. I - So you’ve never felt rushed?
195. P- no.
196. I - They always make time for any questions that you have?
197. P – yea
198. I - do you feel like that they give or that you need time more than the average patient, or not really?
199. P – No
200. I - How about in terms of attentiveness, is do they do anything to build a rapport or a personal connection with you or is that not really part of it when you talk about attentiveness.
201. P - Yes they do build a rapport
202. I - So they ask you personal questions and get to know you you would say
203. P - Yes
204. I - Ok mhm ok. Um so these questions right here, so going back to that for a second. So there’s nothing else that you would be asked and you feel like this especially the two questions you answered yes to capture everything and say it in the right way.
205. P – Which one
206. I – 4 and 6
207. P – What you can add to the six is… um explain how.
208. I - Explain how, so details?
209. P – yea
210. I - And that would capture like you said...
211. P - OK because some people may be able to do the shopping but not be able to visit the doctors office, lack of transportation to get to the doctors office, the medical transportation
212. I - Right so you could provided STS could take you there? Or you could provided that you had your mobility aid
213. P - Yes. In this question I think there are too many things covered
214. I – Too many things as far as this first part of this sentence you’re pointing to?
215. P - Yes because each thing it will be different
216. I - So its too too much variety in that question
217. P – Because you cannot be specific in this, this is you can tend to be vague
218. I – Ok
219. P - Yes I do and then what? tell me more and how
220. I – So ok let me offer a different way of doing this then, another one that’s being considered by healthcare systems. So rather than asking specific questions, um maybe it would be a conversation that the healthcare provider has with you just in general and with listening to your responses, has checkboxes that they would mark as far as yes or no’s to various accommodative needs or difficulties. So, for example instead of asking questions it might be a checkbox that says mobility aid used, or a checkbox that says social interaction difficulty
221. P - For example visually impaired, I have low vision. I need a magnifying glass. I need glasses.
222. I – Mhm. Right, so it would kind of be impaired vision, a checkbox.
223. P – Yes.
224. I - Ok. So tell me um do you have a preference of either the full written sentences or something else like that instead, the checkboxes with just listings....
225. P – Checkboxes.
226. I – ok
227. P - because you break it down, right?
228. I – Just more straightforward, you think
229. P - More closed ended questions is better, you can always ask for more
230. I – Right ok. And would you how would you want this information to be collected. Would you want it to be on a patient intake form perhaps. That you fill out? Or would you prefer it to be asked of you or through a dialogue with your healthcare provider that they document? How would, what would be the...
231. P - It can be done after the visit, but not in an intake form and I’ll tell you why.
232. I – Ok
233. P - The reason is first of all in the intake form sometimes you’re not.... maybe in the first visit you have the time because they have to wait for you to complete it. follow up visits I’ve never finished up that form fully.
234. I - You’re talking about the satisfaction survey or…?
235. P - No no no no no. The intake form. What are all, how has your condition improved and specific conditions.
236. I – Mhm.
237. P - Ok so. If I’m gonna look at this after that I’m gonna say “oh god i’m not going to bother about that.”
238. I - Mhm
239. P - I can guarantee you nobody’s gonna look at it
240. I - after the fact.
241. P – Yes, because it is too much paperwork in somebody’s mind, it’ll be in my mind.
242. I - Ok. So you are saying don’t do it after, you have to do it at the beginning?
243. P - Don’t do with the intake forms
244. I – Ok don’t do it with the intake, so then when would be..?
245. P - You can ask soon after the visit, right there, “do you mind just doing this?”
246. I - So right after they visit, before they leave the visit... so like upon check out, so to speak
247. P - Or since we are in the electronic age, “can I mail you this and could you fill it up?”
248. I - So you think that would be OK, you prefer to be mailed or emailed?
249. P – Yea, emailed. Not even mailed, not snail mail haha. Because trust me, I won’t mail it back I’ll forget about it or something like that. You got a better chance on email.
250. I – Ok.
251. P – It’s like when you send your satisfaction survey you can ask these questions also.
252. I – So but do you think that waiting till after the appointment would be the best time point? If this information, so do you think . . . I guess let me back up and ask. Do you think having this information is important for them to have before they have your visit with you.
253. P - I was just thinking, you can do like they have the pre-and post test. So when you call up to make, when they call you to make the appointment with and remind you “ok you got your doctors visit on this day”, also let me take some information to update and if anything has changed, and do you mind answering these questions pre-? And the post can be as I told you, you email it after the visit. So before the visit and after the visit you have it both.
254. I – Mhm.
255. P - Has anything changed.
256. I – In terms of these aspects of these questions
257. P - During your visit.
258. I - Anything else you would add to those questions? Things that you might uh that they might ask you to assess your satisfaction with their care?
259. P - Besides the rating scale?
260. I – Yeah, just in thinking of your experiences now that you say are very positive relative to your experiences in Oklahoma that were not positive. What would you have liked to be asked in order to make sure that Oklahoma knew that they has to do some improvments?
261. P - And how would you like to have done differently?
262. I – The care? Or what specifically?
263. P - That could be a question, how. Lets say I, my experience I’m saying now is different from Oklahoma, how is it different and what would you suggest could be done to make it different?
264. I - Mhm. So in keeping with your closed ended type of questions and just narrowing it down, could you give me any specific categories that you might expect to answer in terms of suggesting differences for improvement
265. P - Such as? categories of disability or categories of the services?
266. I – Of the services or the quality of the services...
267. P – Ok um… The nursing care, the doctors team, meaning the doctor and their assistant. And actually starting from the registration, registration, Nursing care, And doctors team.
268. I – Mhm
269. P - Three categories
270. I - So like front office staff as well
271. P – Yes, and the follow up
272. I – and follow up. And has that registration experience always been positive for you as well?
273. P - Yes
274. I - Ok. So tell me you know...
275. P - Sometimes not now, but before the wait time has been long.
276. I – Right, mhm.
277. P - … the bad experience I’ve had is another thing I can think of is [hospital 2] 14th Ave.
278. I – Mhm.
279. P - I broke my leg. I went there. That time I didn’t have much insurance, but I don’t think it was insurance related. They are too crowded so I was left in the hallway whole night and I was discharged only the next evening. It was only a broken leg.
280. I – hm, ok. And you think that service would’ve been the same for anyone regardless of disability status anyway?
281. P - I think so.
282. I - So it was just overcrowding there. Ok.
283. P - And another thing that a problem with healthcare is they give, sometimes they do too many unnecessary tests.
284. I - Tell me more about that.
285. P - I make, as I said, I came went for a leg
286. I – Mhm, a broken leg
287. P – that was broken. And they did blood test they did a scan and they did all kinds of things.
288. I – mhm.
289. P - But I felt was unnecessary.
290. I – Hm. And do you feel that any of that was done because you have spina bifida or they would’ve don’t it regardless?
291. P - My feeling was that it was done regardless.
292. I – Mhm. Ok.
293. P – It was a way of. .. the Money factor there.
294. I – Mhm
295. P - And that I notice quite often.
296. I – (inaudible) reimbursement thing, running of the tab kind of.
297. P- Yea. It happens quite often actually. Think of it. Managed care.
298. I – Haha Manage care exactly.
299. P – that’s our problem right.
300. I - So so tell me, I mean are there, in thinking of yourself or anyone else that you’ve interacted with with a disability, are there any new things that you’ve heard of or encountered that our no-no’s, do not do this, this is not how you interact with me or someone with a disability?
301. P - The definite no-no is don’t talk to somebody else when I’m the patient.
302. I - Mhm
303. P - And it’s always good to be at the same level as far as your looking at a person, the eye level really.
304. I - So asking them or expecting them to come down to your level if that’s the circumstance. Ok… So come to my level.
305. P - You can sit across me and talk to me.
306. I – Mhm. And how is that been, that specifically, in regards to registration, you said that the registration process has been very positive overall. But you know sometimes often times um registration is through like a little window or whatnot, and it tends to be a higher up countertop, is that ever been an issue for you?
307. P - To tell you the truth I’ve never noticed that. That’s a good thing.
308. I - Well I don’t mean, I don’t wanna make it something, I’m just asking in general.
309. P – No, that’s a good point. It’s just that I haven’t observed it so I guess it’s not an issue.
310. I – Ok, ok good to know. Ok. Any other No-no’s that you would uh, kind of if you could train a healthcare provider.
311. P – Yes. make a doctor’s office accessible.
312. I - Ok tell me about that
313. P - Because I had to go see a dermatologist, and guess how we had to go to his office. Through the parking garage and it was right at the back and it had a step.
314. I – Mhm
315. P- It was actually in (city).
316. I - So you had to go through the parking garage and it had a step there too? so how did you manage to get in?
317. P – No, I have a manual wheelchair
318. I – Ok.
319. P - which I use when I go in the car
320. I – Mhm.
321. P - you can tip that chair and go in.
322. I – So you had to do that?
323. P - The person who came with me ..
324. I – helped you navigate the step
325. P – Yea. If I was alone there’s no way I could’ve gone to that office.
326. I – Mhm, ok... Any other types of accessibility issues that you’ve encountered?
327. P - In the healthcare system, no.
328. I – Ok
329. P - Because that was a shocker to me, doctors office. haha.
330. I – Mhm
331. P - I do understand some buildings are old and they don’t have to comply to ADA. But still..
332. I – Mhm, can you think of any any reasons why you would not want to share about your disability status or any needs that you might have, any concerns or reasons why you wouldn’t want to tell a healthcare provider?
333. P – No, I’ve never found any reason to hide the fact
334. I - Mhm ok… So I think yeah those are those are most of my my questions, I guess maybe one other question is when a healthcare provider learns that you have spina bifida is there any particular way that you would expect them to react or respond to that information?
335. P - Spina bifida in particular?
336. I – Mhm.
337. P - Nope.
338. I - So just act like....
339. P - Maybe some, and I’m not (inaudible), maybe some people think that all people that have spina bifida have the same needs. We don’t. It all depends on um the level of the spina bifida. Because I, at my job have encountered a couple of people who, some of my consumers have spina bifida. And I look at them and I say, to myself, they have a lot of needs, I’m lucky.
340. I – Mhm.
341. P - So I don’t know if healthcare providers assume that. It’s like all disabilities are the same.
342. I – (inaudible) certain type kind of in the same situation
343. P - And that we have the same needs. It’s not true.
344. I – Mhm. So..
345. P - I’m fortunate I can take care of myself. some people cannot with spina bifida.
346. I - Mhm. So avoid assumptions about capabilities.
347. P - Yup.
348. I - So in that case you think asking these types of questions or the checkboxes whatever format would kind of
349. P - give you a clue about,
350. I – give them more insight into not making those assumptions. Ok. So so based on that idea of even two people with spina bifida have different needs or different abilities would you kind of reflecting in that thought process, I’m looking at these questions again, any other thoughts about or any changes or modifications?
351. P - …That would make it very lengthy right? that’s the thing.
352. I – Ok well but tell me, even so, even if it was to make it lengthy, what are the thoughts that you’re having?
353. P – That is for each disability how a differs, the needs right, you’re talking about.
354. I – mhm sure.
355. P - You could ask how would your needs differ from a person who is probably,.. obviously if it’s like a visually impaired person they need their equipment is different… but besides that in terms of mobility in terms of taking care of themselves, how do they differ in terms of taking care of yourself? How do your needs differ from someone who is probably hard of hearing? Or who is visually impaired? For example hard of hearing people they feel that they’re not being acknowledged. Because I’ve made this mistake myself, when I’m talking to them, I look at the interpreter.
356. I - So similar to what we were talking about before with the caregiver focus
357. P – Yea. So I should be actually looking at him or her when the interpreter is the one interpreting... so even if that person is looking at the interpreter I should be looking at that person to show that I am listening to you I am focused on you.
358. I - Mhm ok. So in thinking about just what you said would you want or would you suggest in that situation specifically, maybe a check box or a question that asks you know, do you have an interpreter or something like that?
359. P – Yes
360. I - Ok
361. P - In case of a physical disability, what are the challenges of accessibility? I tried to find a house, I took one year to find a house because every place I went to was not accessible.
362. I – Mhm
363. P – And you would think in an age of ADA that everything is accessible. No. You cannot assume
364. I - So maybe even something like that as a follow up to number four here about um difficulty walking or climbing stairs, maybe a checkbox as far as whether mobility aid is used or whether you had difficulty even entering the physical space of where ever the healthcare provider is.
365. P - And how accessible was your healthcare provider
366. I – Mhm
367. P - considering your disability.
368. I – Mhm ok. And one other question about um any follow-up that you’ve received with healthcare um, have you received any educational materials that give you information about medication or treatment or any plan of healthcare, like things self-care things that the doctor was asking you to do?
369. P – Mmm…
370. I - Or instructions of any kind?
371. P - ..Ya
372. I - Ok and um. I guess a question like. Were those kind of general information
373. P - General
374. I - or was anything like specific to...
375. P – not specific, general
376. I - do you feel like there was anything that it would’ve been better if it was more specific to spina bifida?
377. P - … I would like to, I still am confused about the cause of spina bifida.
378. I – Mhm
379. P - I really am confused. Because only when I came to America I heard that it’s lack of folic acid in the mother. Which I still don’t agree with.
380. I - Ok. So what are your thoughts then?
381. P - Of the cause of it? It’s got a hereditary factor I know. I’ve seen it in families. But I have no clue what the cause could be.
382. I - But you don’t think it’s the folic acid?
383. P - No. Because I know. folic acid is in so many things, so many vegetables...she’s eaten um haven’t you been eating vegetables so much, Mom?
384. C – We normally we eat a lot of vegetables. We don’t eat a meal without vegetables.
385. I - Mhm
386. P - Another thing that confuses me is that I’m the third child
387. I – Mhm
388. P – (inaudible) my brothers are fine, but I’m the third child.
389. I – mhm
390. P – So What are they saying that with each pregnancy the folic acid is less in the mother? I don’t get it.
391. I – Right, cause you probably had the you’re saying you probably had the same diet you did as with your two siblings
392. P – Yea. And she had a lot of vegetables, and it had a lot of folic acid in it. (Inaudible) medication. But now it’s the obsession of folic acid folic acid. I don’t agree with it.
393. I - So you would’ve liked more information on other potential causes then.
394. P - And I wish there was a chapter, a spina bifida chapter in Miami. I really search for that.
395. I - A chapter, how do you mean?.. like a ..
396. P - A support group.
397. I - A support group. ok so there is there is none that you’re aware of.
398. P – In Orlando, not in Miami.
399. I - Ahh ok
400. P - I really wanted to join a support group it’s good to know each others experience having a similar disability.
401. I - Sure. Are you in touch with anyone since you can’t do it locally, anything online or anything like that?
402. P - No.
403. I - Mhm.
404. P - I at one time was thinking of starting one but it’s a process.
405. I - Hmmm ya. And what you said it would be helpful to kind of similar experiences, is that specific ..
406. P - For example healthcare experiences another person you speak to may not have the same experience as me. Each person‘s experience is different.
407. I – Is that more so just to learn from each other‘s experiences or..
408. P – and coping.
409. I – and coping.
410. P - Somebody can help me and I can help them.
411. I – Mhm
412. P - Even for the families it could be good.
413. I - Sure. Mhm ok. well is there any, I mean, those are all my questions, I mean, any other thoughts...
414. P - I hope I was of help.
415. I - Of course you were, always. Any other last thoughts, anything I should know that you didn’t talk about?
416. P - No but if I do I definitely will call you.
417. I - Ok awesome.
