# Supplementary material for: Disability disclosure in healthcare settings for individuals with developmental disabilities: A qualitative study of patient and caregiver perspectives
Source: PLoS One. 2025 Aug 7;20(8):e0329328. doi: 10.1371/journal.pone.0329328 (PMC12331114; doi:10.1371/journal.pone.0329328)
Supplement: S1 File — (ZIP) [file pone.0329328.s001.zip › Transcripts/2019.08.29 Interview 05 Transcript.docx]

I: Interviewer P: Parent

**I: Alright so, I’m hitting record and just for the record, I’ve gone through the uhh the informed consent that you signed, but you’re okay to to participate and to be recorded?**

P: Yes.

**I: Alright, thank you. So so in general, you know, I want to know about healthcare experiences that you’ve had, you know, interacting with various healthcare settings–whether that be regular doctor visits, or any specialty visit, or urgent care, hospital, all those things. So, umm, overall would you say you’ve had good *and* bad experiences with the healthcare setting, or one more so than the other?**

P: Overall, I think it started off being kind of uhh you know we would just go there and uhh you know it was “Next!” you know they see you and then its “Next!” you know. No no umm no feeling you know they want to they just want to recommend medications and move you forward. I think as a parent, umm I had to become extremely diligent and kind of yell a little and push buttons and I’m sure I was not on the, you know, the doctor’s favorite list. But you know something, if you have to push to be an advocate for your children, and I think now they understand. They understand me and they understand that when I walk into an office now they better have done their research before we showed up as to, I don’t know, new things going on in the industry or not just push pills. I wish I would’ve brought you uhh (**inaudible**) and I sometimes have to put an email together for all the doctors that they do see and tell them uhh look, this is the neurologist, the gastro, the endocrinologist, the geneticist, the ear institute. You know, it might be a group of ten doctors saying uhh, you know, I just want to make sure you’re all talking to each other. Because I don’t know if all your pills are contraindicatory of… I mean imagine this blown up though (shows something). I can’t (inaudible) here because I’m not with them today, but this is one full sheet like you’re seeing right here…

**I: Sure.**

P: of all the medications just my 19 yr old is taking.

**I: Right, mhm. So you’ve got them by specialty? Mhm.**

P: And so I’m like you know something, for somebody who weighs, oh my god she weighs … I think 88 or 90 pounds. To pump up a kid with that many…

**I: Yeah, that’s crazy.**

P: I want to make sure that the geneticist knows what the cardiologist…you know so that if somebody goes oh gosh. I don’t know, the neurologist goes oh wait a minute, this and this are not going to mix or…um

**I: Right that’s twenty…yea I’m counting at least twenty-four different medicines on there.**

P: Correct. And then this is my other daughter (shows another sheet of paper). I mean it’s not as much as.. and meanwhile she’s more uhh she’s got more issues. On top of it all, when she was two, fell off of her bed in the middle of a hurricane from a very small bed, and suffered a brain injury. So on top, I had more complications and yet my younger daughter umm seems to be uhh…so I think if it weren’t for parents…oooof…demanding uhh more conscientious decisions for our children, I think it has been because I’ve pushed them to their limit.

**I: Right.**

P: And now they just know, you know something, I better, like I said, I better quickly check the girls records before I come in because if I come in and I know that they haven’t, they’re not up to speed then I’m like why did you just waste my time.

**I: Right. So in general, it wasn’t like that to start. But as you’ve continued to interact with them they’ve kind of, you’ve made it clear that this is how you need to operate.**

P: Correct, correct.

**I: Ok. And I just wanted to go back to the, you said ‘no feeling’, can you tell me what you meant by that?**

P: I think they want to, you know, see a patient, quickly make a diagnosis, and push you out. I felt no care, I’d come in crying, you know obviously I don’t know what’s going on with my children, why was one being born with… and I was constantly being uhh well something here, here are some pills it’ll stop the epilepsy, and come see me in six months.

**I: Mhm.**

P: Well but is somebody going to look into…it took them 15 years for somebody to actually say you know something, why don’t we look into this case, it’s you know very odd. Umm I don’t know I feel like the I feel like uhh we’re just uhh “bring in, ching ching, next! next!.”

**I: Uh huh. So you felt like they were only dealing with umm what their specialty focuses on and not looking at anything else or referring out to other individuals that might help?**

P: Exactly. Yea, they see you, they take their notes, they give you a medication, they say come back and see us in six months.

**I: Right.**

P: And I understand. I mean there is, especially at [Hospital 1]. I mean I understand there are a lot of children. Uhh but then space them out, maybe space out your appointments every hour and don’t you know don’t make an appointment every half hour. And then realize all of a sudden you have to wait in the waiting room for two hours with two kids that have disabilities, and then a mother who is about to rip off their head because uhh either they’re, they’ve got reflux so they’re vomiting in the waiting room waiting for…there was no personalization.

**I: Mhm, right. Okay.**

P: Now they know that if I have an appointment at 9, I swear and I’ve seen it which is kind of terrible, I’ve seen them skip over people because they know I’ve come in and I’m in on time…

**I: Ohh, so they haven’t had that conversation or push as you’ve had..**

P: …and they sometimes blow the people off that are in the middle just to attend to me because they know that I might make a scene, and that’s kind of terrible. So then I’ve now jumped over people who have been waiting there probably an hour longer than me but if my appointment in 9 o’clock and he’s the one that’s been delayed, that not my issue.

**I: So you think based on that you know they haven’t necessarily “learned the lesson”, they’re just kind of providing attention to you because you’ve made it a point where you know that’s the expectation, but they haven’t really learned and applied that to all their care.**

P: No

**I: Okay**

P: And frankly, I think the biggest problem is their phone call services now.

**I: Mhm.**

P: Oh my god, doctor. I-I-I get on the phone to call any doctor and it takes–the other day waiting for the neurologist’s office to pick up and I had to take a picture of it, it was 45 minutes.

**I: Mhm.**

P: Uh only because I–I have demanded their emails. I took a picture and I sent it to whoever I’m about to discuss anything with.

**I: Mhm.**

P: And I’m like 45 minutes on a telephone? To ask a very simple question. And yet they’re like oh my gosh yes we’ll look into it. I know they don’t.

**I: So 45 minutes to get that and then no follow up. Mhm. Okay. So yeah, tell me more about the–what have you experienced that you would call a bad experience, you know, just adding to what you’ve already told me? Are there any things you can think of that you would single out with regards to how you are treated by anyone on the clinical staff or front office staff that kind of, you would say that needs to be improved to make this a better experience for you?**

P: (sighs) I-I-I swear I think that they are just getting greedy and I think that they’re instead of the old-fashioned way, you know, you make an appointment at 9, at 10, at 11 take the time to listen to your patient and their needs. You know something, maybe do a little research, follow-up. Or have a, you know something, have an intern or somebody who is um rotating, so you know something, umm these children have umm something odd can you do a little uhh research on children who are deaf that might also … I think that they are just … I think they’re just booking.

**I: Mhm. So kind of a lower threshold than bare minimum.**

P: They’re booking let’s say every… yea at like every half hour they’re pushing people in. It’s a quick review. Okay so has anything changed in six months? Yes, no. okay. Uhh you’re still on the following medications? Yes. Okay. Well you know something if nothing’s changed, that’s great…

**I: Mhm.**

P: ummm why don’t we check up uhh in six months? No you know something I’ve heard that there’s something up and coming up in the market… or not that I believe in medical marijuana, but you know something I you know obviously that went to Califor-, umm Colorado, that uhh, you know, I just have one little girl in my mind, that Charlotte’s Web who, you know, the parents took that risk and went out there and they did try it and now the child doesn’t even have any seizures, she talking walking.

**I: Mhm.**

P: Versus… I don’t know where they came from, it might have been, did I hear from Miami?...umm imagine the parents had to do like their own research

**I: Mhm**

P: Instead of

**I: Mhm.**

P: A medical professional saying, look this might be a little out of the way or a little over the top, but why *don’t* you try it out? You know.

**I: Mhm**

P: It was up to the parents to take that risk to do that for their child.

**I: Mhm**

P: I don’t think enough research is being done.

**I: And do you find, you know you said that now they will take you sooner because they know that you have a higher expectation, do you feel like they’ve done anything on that end with more research or is it still on you to do the research?**

P: I think that they are obviously attending to me because they know that I-I–but then when I go in and say has anything been umm I don’t know, is there anything out there that’s new, upcoming, new medication, no medication? Umm they’re like, all they keep telling me is in your case Mrs. (Interviewee) it’s different because your kids have something that’s..it’s so.. it’s a mutation that’s that’s not even really studied

**I: Mhm**

P: it has been really…And yet if you looked it up in the medical journal because they did send me the actual paper that went up

**I: Mhm**

P: It’s four boys and then there’s my girls' study. The four boys don’t have any of the characteristics my kids have. They all have either bigger ears or... and they’ve all had one additional element which is Usher syndrome which they’ve all gone blind. Mine don’t have that.

**I: Mhm**

P: So, I-I, you know, till this day, I don’t even know if that’s what they really have. I don’t know why they were categorized in that.

**I: Right. But the explanation is we...there’s no sense in doing research because you’re in a different category**

P: We are in a different category so therefore we don’t even know what medication to give you

**I: Mhm**

P: Whatever we think is gonna be actually probably good for your kids, your kids have always umm broken the mold.

**I: Right.**

P: I’ll just give you another example. The orthopedist once told us because my daughter, my eldest daughter, is in a wheelchair who doesn’t walk, said look.

**I: Mhm**

P: She was walking with like her leg, her knee moving in..

**I: Mhm**

P: He said we could do something very aggressive, *very* aggressive. But she’ll stand up straight at least and so even if she’ll never walk, she’ll walk with a walker though or something. But she’ll stand up tall and her legs will be straight.

**I: Mhm**

P: He said but it’s very aggressive. It’s an entire femur rotation.

**I: hmm**

P: So I basically have to saw her femur

**I: Mhm**

P: Saw it

**I: Right.**

P: Rotate it. And put her in a cast for three months.

**I: To stabilize it**

P: But you know something, she’ll walk straight.

**I: Mhm**

P: And what do you know, it’s the *one* case in the *entire* (Hospital 1) that they’ve done a femur rotation on a child that didn’t... because my kids seem to break every mold umm

**I: Right.**

P: And yet I don’t blame him at all because at least he–he took that risk to say let’s try it. My husband will never forgive me for it. Umm

**I: But you’d say he was forthcoming with all the uhh you know these are the risks, this is the process, this is all that?**

P: It was 100% guaranteed, is what I was told. He said look..

**I: As far as known, mhm**

P: There hasn’t been one, do you want an interview of th e 50 or 60 kids that I’ve done? It’s 100% guaranteed. She’ll at least stand up nice and straight. It’s a femur rotation, she can’t there’s no way she can pronate if I rotated the entire femur, there’s just no way.

**I: Right, mhm.**

P: Except my daughter.

**I: Right.**

P: So umm, but you know something, he’s the only one that really took a chance then to say let’s try something, nobody else…I think everybody is either afraid to get involved because again my kids break that mold–

**I: There’s no guarantees, mhm**

P: So nobody dares to say anything.

**I: Mhm**

P: And yet, I think so much research should be done. People go in like brand new medical students or whatever, somebody take this up and be then the crusader of, you know, something. Let’s examine this case, you know, it’s a lot.

**I: Right. So–so, tell me umm more umm about how–so you are always the person who accompanies your daughters to medical visits?**

P: Yes and and usually umm a nanny or–or visits two of them when it’s too much, you know.

**I: Right. And are they able to communicate with their healthcare providers?**

P: Yes, yeah.

**I: Okay, Mhm**

P: Either sign language

**I: Okay**

P: or, you know, it’s funny how we when you just grow up with somebody, you know their either their facial expression or their...

**I: Mhm.**

P: so we already kinda understood what more or less they were trying to tell us.

**I: Mhm, right**

P: You know, more or less.

**I: So–so tell what those interactions are like. Do you find that umm that the facilities and the health care providers are–are ready to communicate, they either they know sign language or they have services available? Tell me about that.**

P: Actually that’s another good point. My children are deaf. So and I’m not, I–I mean I want to say I’m 80% proficient, but umm they’re, you know, in school all day so sometimes yeah I will go to a doctor’s appointment and my children are saying something, and I if I don’t know what it is I–

**I: Mhm**

P: and there are no interpreters.

**I: And is that because they just don’t make that available at all? Is that so that’s not even a** **service they offer and it’s just they weren’t able to schedule it, or?**

P: You know something, I don’t think I, now that you really mention that, I don’t think that I’ve, in the 22 years of…I’ve never seen one something a service being provided for that.

**I: Mhm**

P: That’s something that I think…there should always be someone on staff. Even if it’s only one person for the whole…what are the chances really that someone is going to come in deaf. It’s a resource that they should really think about.

**I: Mhm, right. And do, do your daughters make decisions for themselves or do you, so you are their proxy or their?**

P: I have their–we have a legal guardianship set up as of eighteen.

**I: Okay. And do you find umm when they are able to communicate, is it is it always through you because of the lack of interpreters? Or..**

P: Yeah, they immediately, correct, they immediately look at me because they know the doctor doesn’t sign. So, they, you know, they look at me to tell me if the doctor is trying to explain, you know, do you feel okay, does it hurt anywhere? So, I mean if they tell me “this is hurts” or if they tell me it's up here or here, the ear, the stomach, or the knee, or umm, but you know it took me a long time also to learn how to sign. Which you know it’s not that–that easy. Some stuff is, but again if I’m at a doctor’s appointment and they start telling me something that I’m not aware of then I feel terrible. And they get frustrated because they’re telling me the same thing and I’m like…then I’m embarrassed I mean I–I am trying to get up to speed, I’m trying to go back to school for that. It’s just umm the lack of, you know, time too is a is an issue.

**I: Sure, of course. Did you ever find that, you know, because you–you don’t uhh have the ability to translate everything, that there’s ever been a–a consequence about that like uhh lost opportunity or–or anything from that missed information?**

P: Yea, there there has been. My umm my eldest daughter before she had her first seizure, she kept telling me that her head was hurting.

**I: Mhm.**

P: And when I called the doctor, they were like “Well, but is she saying why?”, And I’m like “No, she’s–she’s just signing”.

**I: Mhm**

P: But I don’t know what I, you know, she was trying to tell me something and I–I just said look she’s telling me that her head hurts.

**I: Mhm, mhm.**

P: Umm and then that night she had a seizure.

**I: Mhm.**

P: So I don’t know if obviously she was trying to...

**I: Mhm**

P: I don’t know if she was already feeling something, but she was telling me and I–I all I could say was, you know, all I could tell the doctors was look she’s umm she’s signing that she has a headache.

**I: Mhm, right, right. Umm..**

P: I’m sure she was signing something else, but I–I either I didn’t understand it or umm but I think the doctor maybe could of...and again it’s because they–they don’t have sign language personnel there so it was me just guessing.

**I: Right, as best you can when that service isn’t available. Right. Do you, I mean you mentioned their–their frustration at times in trying to communicate, do you do you sense any other emotions or they communicate anything to you about, you know, the that experience of having to go through someone else to communicate versus them being able to communicate with their healthcare provider?**

P: Yeah, I and I see even their frustration with me when I don’t understand. I can see their frustration.

**I: Mhm.**

P: Their behavior is almost umm I’m not going to say aggressive either but it becomes uhh (sighs) it hurts my heart. That’s why I’m–I’m trying to go as fast as I can and I’m trying to find people maybe they could come to my house to umm

**I: Mhm.**

P: Even if they can stay with me 2 or 3 hours to just help me get through some of the..

**I: Right.**

P: Because they’ll–they’ll keep, you know, signing the same thing like over and over

**I: Mhm.**

P: And I even umm I even had uhh what is that called..placed on my tv..V vechs V E C H. It’s uhh you can actually call in to a service and you could see them, somebody signing

**I: Okay**

P: and I’ll actually tell him. I’ll say umm “My daughter is signing the following uhh sign, can you please tell me what it is?”

**I: Mhm.**

P: And then she’ll sign it and he’ll say oh it’s blah blah blah. And I’m like ohhhh. You know I’ve had to use that service because I’m…

**I: Mhm. So that’s proved helpful to you**

P: So I–I–I think, you know, something, umm that is probably a very big–big deal for me is that everywhere I go, there aren’t..

**I: Not any even offered, it isn’t even a possibility**

P: Never, it’s never even discussed, never offered.

**I: Right. And there’s no other attempts to–to communicate through umm, you know, there’s these technologies that are like boards**

P: Yes, I’ve tried that.

**I: communication boards or writing out, is anything like that ever offered or suggested?**

P: Yeah, it was. And we we did try the board. But umm

**I: It didn’t work out so well?**

P: It didn’t work, no.

**I: What uhh what didn’t work about it? Just uhh so I have a little bit more information**

P: Umm it it you know what I actually had. I had little key rings.

**I: Mhm.**

P: uhh One was just emotions, one was just food, one was just uhh so that they could just go okay here’s the food sign, here’s the emotion sign, so you know like I’m really hungry for pizza, for…

**I: Sure.**

P: but if I didn’t have the rings or they’re at school or whatever then all of a sudden, their–their aggression was to start pulling their hair out because they–they didn’t even in those days they didn’t even know how to sign.

**I: So that, yeah, so when the, so signing kind of took the place of that?**

P: Correct. And then we took the board with us and the board all of a sudden ran out of batteries and I thought you know something, there’s just got to be something really...and that’s why we chose to do the sign language

**I: Mhm**

P: The problem is you’re right. If we’re out and there’s not sign language interpreters, it’s really up to me. So, I was spending (sighs) at least three hours a night going to school to try to get up to speed as fast as I could. And obviously at school I hired a one-on-one to be with her all day.

**I: Mhm**

P: Now the problem is that my 22 year old is out of school and she’s with me. Uhh and she’s signing to me

**I: mhm**

P: And half the times again, when I call in or I’ll call her, she was placed in another school and I’ll say why is (daughter 1) giving me the same sign. I don’t know I don’t understand what that is. She said you know something, she’s–she’s know umm she knows you don’t sign that well so she’s inventing signs just to get you upset, and I’m like ohhhhh.

**I: Ohhhh**

P: So umm, it goes to show me that you know I really

**I: Mhm**

P: I really need to have somebody at all times because then correct as you said what happens if they’re trying to tell me yea I feel a seizure coming on or I feel something really bad in my head, uhhh

**I: But the but the attempts, the key ring, the communication boards, those are all all have been on your end**

P: On my end

**I: not something that is offered or suggested by the healthcare setting or healthcare provider?**

P: No believe it or not the the board was was offered uhh at one point by a school that I was in

**I: Okay**

P: Uhh but you know there’s only eight if here were like eight uhh

**I: It was limited function you would say?**

P: Yea what else. And that’s why I thought oh well let me come up with keychains. But then you know she’s got to flip through them and find, you know, like again I’m hungry or thirsty for Gatorade or do you want water, do you want...it was trying to figure out through all the little uh

**I: Mhm, right. Okay. So umm anything else, you know, we were talking about uhh interactions with the healthcare provider, and that could be the nurse, the doctor, anyone. Umm anything else you can think there? Or we can broaden it to anything about the the physical environment where it be any accessibility issues or equipment that may or may not be appropriate, modifiable, adjustable, anything like that that comes to mind?**

P: … I–I–I think I’m just so disgusted with the whole umm...

**I: Mhm.**

P: I guess it’s not that I’m disgusted, I’m disappointed that they don’t have more for kids with disabilities. I think when I go into the hospital, especially the neurologist, and I see this room full of parents with kids that are, and obviously and they are in worse situations than my girls I mean I’m I, you know, they're either traches or they’re , you know. I don’t see any I don’t feel any compassion. As you said maybe something modified, I don’t know, either paint the room they’re that looking at with, even if it’s big fat clouds or. You don’t feel warmth of...

**I: Mhm**

P: if I had an office that I was seeing children in,

**I: Mhm**

P: I would make it really, that one hour that they’re out of the house or two hours going to see a doctor

**I: Mhm**

P: Then make it even pleasant for the parents, I mean we’re–we’re

**I: Sure.**

P: we’re depressed too. We’re, you know.

**I: Mhm.**

P: I don’t know, I don’t feel that anybody has

**I: is trying to connect you on a human level**

P: empathy. On a, correct, on a human level as a … even if you’re a nurse and you’ve never had children or whatever to say, you know, mom. You know, it’s it’s okay or or can we offer you umm can we bring you a board, a puzzle, uhh..it’s just a cold…just show up for your appointment so that we can charge you, get in, and move out. Sometimes the–the room is so full that not all of us can sit down. There’s carriages and wheelchairs, you know my daughter is in a wheelchair. We don’t fit in a room. So they make us all sit out in a hall, I just think it’s so impersonal.

**I: Right, mhm. Impersonal.**

P: Like you almost wish that (sighs) you know, I hope, you know, you’re like I hope it happens to them one day

**I: so that they understand**

P: so they understand really how cold this atmosphere is

**I: Mhm. And is that pretty consistent across all the settings even–even in pediatric settings that you’ve encountered?**

P: Well imagine I’m in a pediatric, even though they’re 19 and 22, I’m still in a pediatric setting. They–they still won’t release them to any, you know, other ummm like like a (Adult hospital 1) or uhh (Adult hospital 2)

**I: Mhm.**

P: umm and yet, you know, sometimes I’ll see the clowns coming around, and but it’s so infrequent

**I: Okay**

P: Umm

**I: So those services you have seen, but infrequent?**

P: I–I think that, you know, it’d be too bad that uhh there weren’t a class or somewhere in medical school to really teach empathy, to really umm

**I: Mhm**

P: Even when they go in to get labs, the the guy just, you know, the phlebologist wants to hurry up, strap you in. The child doesn’t even know what’s going on, you know, screaming, you know, like hurry up let me just take the blood I’ve got somebody else waiting outside. And they make it so fast and so, and then you’ve got a screaming child and, you know, it’s umm (sighs). There are no social skills really,

**I: Mhm.**

P: There’s no bedside manners.

**I: And then in an instance like that specific example with the–the blood draw umm do you have any sense of whether that’s all kind of in the lacking empathy column, or would you put any weight towards, you know, this system is on a schedule and they’re just trying to keep the schedule and therefore they’re kind of cutting out those things?**

P: They are cutting out. Yea, they are cutting out.

**I: Do you feel..**

P: Do you know what I end up doing then?

**I: Okay..**

P: When I see somebody that acts like that...that’s why I’m sure everybody hates me. I I’m telling you, everywhere I go, sometimes I will hear “Mrs. (parent) is here, hurry!” Umm I’ll stop the phlebologist or whatever, and I’ll say you know something, you know, that’s pretty mean, I have a child here. So you know something, so I’ll maybe say, you know something why don’t you do it for me first, just tighten up my arm and go like this and just pretend you’re doing something, just. And I’ll make them slow down and all of a sudden I–I you feel uhh they’re looking at me like ughhh but I have to make them slow a minute

**I: Mhm.**

P: Because they don’t

**I: They don’t even**

P: I think they’re on like a, they uhh they too know they have to make a

**I: maybe as if they have a quota and they have to keep on schedule**

P: it’s kind like, you know, being in a nail polish place, it’s like hurry up because then they have to ring a bell.

**I: Mhm**

P: I don’t know if you’ve ever been there sometime

**I: mhm**

P: they do your nails so quick and then they have to hit a bell so then the manager will say, oh okay she did it in 30 minutes.

**I: Mhm.**

P: Really…

**I: So same thing for**

P: I’ve had to speak to them to.

**I: Mhm.**

P: You know, that’s that’s not a way to..

**I: Mhm.**

P: to treat another human being, it it’s…

**I: Right. And so you say that obviously you have to take the a proactive roll to kind of advocate, provide information, you’ve got the little sheet with all the medications and specialists**

P: Correct.

**I: Is there anything else that you do kind of anticipatory to–to prepare the–the setting or the healthcare providers or even your daughters for an experience?**

P: Uhh well for some of the doctors I will send them an email, you know, prior saying, you know, I’ll be there next Tuesday.

**I: Mhm**

P: Uhh, again, just so that if there is anything that he should look up. I–I don’t want to just walk in and he’s all of a sudden looking at the screen to see what’s going on. Uhh so that when I do walk in, believe it or not, my biggest, you know uhh, problem has been uhh and again he and I are now are at a good level. The neurologist. Because (sighs) that office is a nightmare and it–it–it really is it’s not getting any better, the phones, the switchboards, or. And he’s like, (parent), you know something, it’s just that’s the way it is now. And I’m like, but you know something, it shouldn’t be that way.

**I: Mhm.**

P: You need to train then everybody below you. Umm. I said–I said I don’t know if you remember but at the way beginning you were you were a… you took the time. He said yeah, we’re, we we now are overloaded with kids. And I’m like well then you then as being the director of this whole unit, uhh need to come up with a plan.

**I: Mhm.**

P: Hire more people, hire… or you know whatever. Uhh (phone rings)

**I: Would you like to take that?**

P: No, I’m just going to send a message. Uhh

**I: So you’re saying…**

P: I–I–I’m not even invited, well, for my older daughter just graduated now from high school, I wasn’t even asked to come to the IEPs. They only wanted to do it, you know, on the phone because, again, it’s another IEP, okay, uhh (daughter 1) is now gonna to do this, and she’s gonna to move on to that class, and then okay sign this Mrs (parent) sign okay thank you.

**I: Ohh they didn’t want to have discussion and…**

P: I’m like what? I’m like I don’t even know what her rights are? I don’t. I–I don’t know the system, you you have to teach me what what am I, what should I be anticipating?

**I: Mhm.**

P: They’re like “Well this is it though. You just need to sign, she needs to move on.”, I’m like *“No,* I–I don’t know.” So, they never–they always they always used to say Mrs. (parent) you don’t have to come in to sit down with us, you we can actually do this over the phone. And I would say no it’s okay, I’ll come up.

**I: You don’t have to, but I want to**

P: I’ll come up, don’t worry.

**I: Mhm.**

P: It’s like nobody wants to deal with me, but it’s because nobody wants to take that additional time to say look this is…I don’t know, somebody talk to me. Somebody say this is you know something, why don’t you look into this for your daughters, why don’t there’s a program that’s…To this day,

**I: Mhm**

P: I–I’m now she’s at home I–I have to find an adult training center for her. The school never prepared me, they’re like look you’re just going to have to do your own homework. And I, but that’s something that isn’t there a–a case worker assigned to her that they should have already known that she’s in a wheelchair, she needs a one-on-one, she had she signs, shouldn’t that have been something that somebody could’ve said look (parent), the best places to go to are here for sign language.

**I: Mhm.**

P: I’m like this is the way you just leave us? We’re we’re that’s it? We’re done? And their like well you’re the mother, you. And I’m like no I think there’s a social worker that’s supposed to walk me through this process.

**I: Mhm.**

P: I–I feel that if that if it’s not for the parent uhhh and unfortunately, you know, the truth is a lot of parents work. I have the–the luxury that I don’t so I–I–I tend to get on the computer to do some research uhh even though I’m shuffling here and then the therapy and back and then pick up from school and then come over here and then there’s another therapy and there’s a.. I–I at least try to sit down and do some research, but there’s moms out there that don’t, you know, have that luxury, you know.

**I: Mhm, absolutely.**

P: Umm when my children were on the school bus uhh and I didn’t even realize that the buses don’t have air, AC.

**I: Mhm.**

P: (haha) You were going to tell Mrs. (parent) that here children were not going to go with AC in the bus. I almost took them to court, they then realized that I was serious, so my children were the only ones in the school that were on an AC bus. And I’d have parents come up to me and say these are kids with traches and–and wheelchair, I felt it was horrible the, and they were like we didn’t have time to advocate so I’m, and their kids were getting off drenched and disgusting. My daughter was coming home with asthma. But you know something, if you don’t…nobody is going to take the time to

**I: So do you feel…do you feel like it’s it’s uhh I didn’t even think this was a problem or do you would you put it more in the camp of, you know, I realized it but we like you said we didn’t have the time or resources?**

P: They don’t, they didn’t.

**I: So they knew.. you would say they were aware and they just did nothing?**

P: I think they were aware, they just, you know, they have to go to work and they–they don’t have time to…so they just say okay this (inaudible) at least I’m getting free bus. Umm but I–I have the time and umm I got all the necessary paperwork, we saw a lawyer, uhh and when they realized that we were serious about going to court, they said okay the (last name) children, two different schools (inaudible) will both have AC in their buses.

**I: So did they fix the whole system, or just those two buses?**

P: Mhm, just us.

**I: Mhm. Interesting. Mhm. So anything…I’m gonna I want to kinda switch gears and talk about positive things, but is there anything else that you can think of that really stands out as far as things that contributed to a negative experience for whatever reason whether it be interactions, conversations, equipment, physical environment?**

P: ….

**I: Mhm.**

P: (phone rings) I mean the... ohh this is their pediatrician’s phone, I’m sorry.

**I: Okay, sure. Mhm.**

P: (picks up phone)

**I: Okay. Something something positive, or?**

P: Well.. just to give you a little difference of opinion uhhh, you know, there’s the doctor’s office that you see for medical insurance purposes and those are ones I think that are, you know, ching ching you come in and go out, and come in and go out.

**I: Mhm.**

P: we have a private pediatrician

**I: Mhm.**

P: Who doesn’t take insurance but from when they were younger, we were so, you know, I felt like we were in the hospital all the time. We were and the–the places that we were seeing again were like a factory, in and out, and no personal attention.

**I: Mhm**

P: There were there were so many things happening with my own kids,

**I: Mhm.**

P: that we decided to go with a private physician who umm, you know, only accepts checks or cash.

**I: Mhm.**

P: And uhh it’s, it’s night and day.

**I: So they take their time?**

P: They take their time to explain things and discuss and the office is never full they make sure almost–maybe it’s because there’s also a very wealthy clientele. I only know because I see the pictures of the kids on the bulletin board and I–I’m like oh there’s Jennifer Lopez kids, the basketball players kids. You never have, you never see who’s in front of you or behind you. Very, very private.

**I: Mhm.**

P: Uhh but, you know like, I–I knew they were going to call me today. We just got another, as I said, another diagnosis from a doctor who said Mom, I see this huge list, and he said let me try to do something different over the summer so that you don’t have to have another…but he did call and say I’m sorry this is, it was like at an emergency level. So, I knew, see they–they took the minute then to call me to say look, we–we just saw the report, you know, we’re sorry that there’s another medication going on but I’m glad you’re in the best of hands and…

**I: Mhm.**

P: They take the time to …

**I: so the bed side manner and the empathy you were talking about before, they express it**

P: and I–I yes, I, you know, hate having to play that that built to see them, (sighs) I have a peace, I have a sensitive peace that–

**I: Is a worth to it that you’re**

P: Correct. That it’s worth it, yeah.

**I: And so if you had to**

P: It’s the temperament.

**I: So what do you think is the difference between? You talked about wealthy clients and non-insurance. Do you think that those are the reasons why it’s a difference experience or what are your thoughts there?**

P: I think that, correct. I think that if you have your own private practice, you–you set your own standard, you know, of…now I remember one of the DM doctors saying I–I decided not to go into the hospital setting because what I saw was it was like a factory.

**I: Mhm.**

P: And she said I wanted to take the extra time because these are the children that I’m going to take care of.

**I: So upping the cost maybe allows for the ability to slow things down, you would..?**

P: Correct.

**I: Okay.**

P: I don’t have the luxury of doing that with everybody, but umm with–with the pediatrician

**I: Mhm**

P: who really is their–and imagine he’s a pediatrician, he’s they only take children but they’ve continued to see my children

**I: mhm**

P: because they love them and they–they know that at least I swear anything comes out or–or any, they’ll call me to say, “You know something, I happen to see in a medical journal that there was a case in uhh Wisconsin uhh that I don’t know was doing something about seizures, and you know something Mrs. (parent) let me see if I can do a little more research and I’ll get back to you.”

**I: Mhm**

P: And then with that information then I would go back to the neurologist, “Did you hear that there was a case scenario”–and they’re like “Where did you get that information from?” I said “My pediatrician gave it to me.” “Oh well I haven’t had the time to, you know, I haven’t had a minute to look at anything.”

**I: Mhm.**

P: Well then what do you have a... do you have uhh what do you call those interns or …

**I: Intern or PA or**

P: PA’s that, don’t you give those assignments of I don’t know. Look you see the kids that we take, you see what some of the triggers are, aren’t they supposed to go out and do some research to better their practice or?

**I: Mhm**

P: And then he always, you know, then remains silent.

**I: He doesn’t offer anything up–or?**

P: No, no. or if I see something in the newspaper or uhh I do get a lot of uhh Psychology Today umm magazines that I–I enjoy reading. But something will come up, I’ll scan it and I’ll send it to him saying, did you read this?

**I: Mhm.**

P: Sometimes he won’t answer, sometimes all he’ll say is “Yes”. You know, not even “Hi (parent)”, “How are you?”, because I’m always very “Hi doctor, how are you? I saw the following article in the paper and I thought maybe you, you know, it would be of interest to you.”

**I: Mhm**

P: You know. I I don’t think they have the time

**I: Mhm.**

P: to do anything.

**I: Right.**

P: But at the pediatrician’s office, they do. They have the time to take a minute to…and they and they love my kids, they’ve always been fascinated by, you know….

**I: Mhm. So it’s more of a yeah the the outlook is more like you said of–of fascination like curiosity, wanting to…**

P: Yes, wanting to say *wow*. These kids are very they really are ummm it’s an odd case.

**I: Mhm. Almost like a like a puzzle if you were like I want to figure this out, I want to figure this out.**

P: Exactly. And imagine at, remember that I was telling you the checklist at the beginning it was first it was deaf, then it was seizures came up, then it was food allergies, then it was as if two years ago all of a sudden we went in for a clearance, uhh gastro wanted to do an endoscopy on my little one, on my 19 year old who suffers from ADHD. My older one doesn’t, but the little one is, you know, an active and she wants to be here and there and, you know, just

**I: Mhm.**

P: this big. And umm we had to go to cardiology and I’m like well we don’t have any cardiology issues, he said yea but I–I need clearance so just…

**I: Mhm.**

P: and when the doctor came out and went like this to me, Mrs. (parent) I don’t want to alarm you but we need to admit your daughter right now to the emergency room she’s having a she’s going into cardiac arrest. And this is me ummm what? And meanwhile my daughter was on the chalkboard, she was kicking a ball, she was…I said what, does she look like she’s in cardiac arrest? They’re like ma’am, she’s in cardiac arrest we need to admit her right now. So when I called the neurologist crying on my way to the emergency room to the neurologist saying this, I said she’s too young to be making a determination I’m sorry she looks like she’s 16 telling me that (daughter 2) is going into cardiac arrest I think you better get over here right now

**I: Mhm.**

P: And he said that’s impossible Mrs. (parent), she’s ADHD. But again, when he arrived and saw the actual uhh sonogram he said wow (parent), the numbers don’t lie. She’s learned to compensate but she–she is going she right now even in school right now she is she is as if she were going into cardiac arrest.

**I: Mhm.**

P: Alright what piece of the, what puzzle is that?

**I: Right.**

P: And she, you know, is in P.E. and she’s kicking balls and she’s…but her numbers…she is right now having heart failure.

**I: So her normal is…yea.**

P: Her outflow is, you know, I’m going to give you a number like 37, when it should be at 60. So you and I might be at 60

**I: Mhm.**

P: but she’s already in 39, which is technically considered cardiac arrest. It’s it’s not, it’s pumping really..

**I: So it’s just a very low (inaudible)**

P: Very, yea. She should she should be wanting to rest, you know, being (inaudible), you know, fatigued and tired. But again,

**I: mhm**

P: And now endocrinology is coming. Out of a blood test that had something to do with something completely..umm something again the pediatrician said, you know something, can I just draw blood from the kids I’m curious about something. And I’m like is this research? I was so excited. She said I I I think I might have found, I don’t know, some correlation or something. I’m like sure!

**I: Mhm.**

P: This might have been just in May.

**I: Mhm.**

P: And these numbers came up umm they’re both of them, again because they’re both like clones of each other, a thyroid number came up extremely elevated umm their triglycerides, I mean their food allergies are severe so they can only have coconut and soy, it’s not like they can have a grilled cheese or a

**I: Mhm.**

P: a fatty McDonalds or…they’re on a very strict diet. And their triglycerides were out of, I mean, again, emergency level not even oh they’re on the cusp or, you know, if it’s a 1-100, maybe at a 150. No, this was something like 500

**I: Mhm.**

P: When the range is between 1-100.

**I: Yea, that’s yea.**

P: It’s like 500. One was 500 one was 525.

**I: So that can’t be explained by diet, yeah.**

P: Uhh and meanwhile the endocrinologist was trying to say I want you to just try something to not give you another pill, but be strict about it Mrs. (parent). Be really strict. And we were semi-strict and still the number didn’t come up. So, because of that pediatrician…so now they’re looking into something having to do with hyperthyroidism or I don’t know what it is

**I: Mhm**

P: but so that’s now “*check*” on the checklist, another issue

**I: Yeah**

P: to look at. But again, it’s–it’s the private

**I: Mhm.**

P: I guess, you know, there’s nothing you can do really about that.

**I: Mhm, right. And tell me umm going back to the, you know, you keep that little piece of paper that, so you make sure they’re talking but how–how is your experience with the coordination of care? Like are–are they talking to each other?**

P: I don’t think so.

**I: Like is does one person does like your primary pediatrician take like ownership of a starting conversations?**

P: She takes ownership, yeah, she takes the ownership. (phone rings) Dear lord I’m sorry.

**I: No problem.**

P: I think that the primary care physician is the one that umm grabs a hold. First of all, they were the ones that really recommended all the people that I should’ve been seeing except for the neurologist. Which I felt was not helping me out, and when they made a phone call, they said you know something please don’t do this to us, you know, we always recommend us to you to our patients, but if you’re not going to give one of our patients the luxury of personal care then we’re going to have to see the top–top–top person.

**I: Mhm.**

P: And meanwhile the top–top–top person, is (doctor) did not have, you know, any more availability and they made *sure* that we did get in. So now that’s why I think the–the people they’ve put now in our path...

**I: mhm**

P: They speak to, you know like, they spoke to the endocrinologist and that’s why they took a minute then to call me who spoke to doctor, I love that. I love that they are letting the doctor know that...

**I: And what about the–the follow through from their–their end towards your PCP in terms of keeping the communication open?**

P: They–they cc everything on the reports

**I: Mhm.**

P: So uhhh you know like let’s say, so (neurologist) last month. I said, this this report will go to my uhh (other doctor) right? And he said, oh yea of course I always carbon copy her. And then I wait like a month and I will call and I’ll say, I’m just curious did you receive the girls’ neurology reports? And she said, yea we received them, all three doctors have reviewed them. And the other way I know that is true is because umm my youngest daughter uhh receives Medicaid, so Medicaid asks for the doctor’s report.

**I: mhm.**

P: And when they do give me the copies doctor, I see all of the is initials on the sheets. So, they all took umm a minute to actually read the report, I see them underline certain things

**I: Mhm**

P: And when I get that report it makes me feel, wow they read it because they underline or… (phone rings) oh man

**I: Mhm.**

P: Exactly, yeah, like I was saying, they underline certain parts of the report with a question mark umm and then I’ll see the question mark ummm tachado uhh

**I: scratched out**

P: scratched out because either they were questioning somebody to go either call the doctor and then they probably got the answer.

**I: mhm.**

P: But it makes me feel I–I love them having to get those copies to send to Medicaid because I see that they’re reading it and asking questions amongst each other, and I–I love that. I know that they are if they’ve got questions, they will follow up them with a particular doctor.

**I: and do you feel like it’s–it’s always been that way? Or are you kind of…**

P: Always been that way. Always.

**I: Okay, great. Mhm. And so, you do you do you consider that a fact of more so this because they–your PCP chose quality referrals? Or...**

P: Correct. They chose quality referrals, but they also they really do follow up with them. They *really* do follow up with them.

**I: Mhm.**

P: And umm when they call me, they’re always like well we’re sorry to interrupt you. And I’m like no, you know, I love it.

**I: Mhm.**

P: Umm they’re like you know we love your children very much. And I said I know that and I–I–I appreciate that you take such good care of them.

**I: Mhm.**

P: Umm so that’s the one thing that I’ve (inaudible) again I know a lot of people don’t have that luxury. Umm but, you know, I know all your questions all have been about the way we’re treated when we go into a

**I: Mhm.**

P: And I do have to say that empathy really is the biggest, biggest ummm factor

**I: Right**

P: I feel that nobody has empathy anymore

**I: Mhm.**

P: Nobody smiles, nobody… I–I don’t know, if I were sitting in the front with a list, you know, oh Mrs. (parent) you’re there we need to…I don’t know, smile or...

**I: Right.**

P: this is your job, you know. Even my old PCP, I hate going into her office. You know, she’s obviously on an insurance plan so I–I have to see her. They’re so mean. There are it’s like 6 of them. And it’s all like a meat market. You see this one quickly to give your insurance, and this one takes your payment, this one answers the phone. It’s–a, it’s a so

**I: it’s like about efficiency over**

P: impractical it’s so ummm and I tell the doctor that all the time, I’m like wow (doctor)… and I’m kind of close with her I too have her number. And she’s like (parent) I–I see literally if you saw how many people I saw a day…she told when it was like 180 people in one day, she said there’s only what 9 hours in a day that I can see patients…

**I: Mhm.**

P: I’m seeing a patient every…she’s again, she’s one of those okay what are you here for, okay, alright, great, alright here’s your sleeping pill. Alright good to see you, bye. I’m like oh…. (inaudible)

**I: Well then let me yea, in that case, let me just jump to the to the last topic umm so ummm we know based on the research that’s available that people with disabilities receive inferior healthcare than those without disabilities or are less satisfied with the care, that type of stuff. So at first, you know, started off with saying well let’s first document disability status so then we can then be aware better informed and then be able to adjust care as needed. Uhh so the question is how–how is best to do that? So first, I guess, do you think that’s appropriate to–to ask about disability status or do you have any concerns about asking that question?**

P: To?

**I: to any patient with a disability**

P: Do I think it’s okay to ask about?

**I: Yeah, do you have any concerns about having someone ask that question or document that information?**

P: *No*, no I think I think that umm no I think that–I think people to ask more questions. I do.

**I: Okay. Mhm. So, I guess from there the question then becomes, you know, how was the best way to ask?**

P: [Long pause] That’s a good question.

**I: Mhm.**

P: Uhhhhh meaning that are you talking umm like let’s say we’re out and somebody ask’s me “May I ask what’s wrong with or what’s wrong with your children?” or are you talking about when you go into a–an office a, doctor’s office

**I: Mhm**

P: and they’re asking you “Well can you tell me more about your children’s disabilities?”

**I: Right so I’m yeah, so this would be only healthcare settings.**

P: Okay, healthcare setting.

**I: Mhm, absolutely.**

P: uhh yes, I think that I think that they should … I think that they need to ask I think they need to ask more questions

**I: Mhm. And are there any...what questions do you think?**

P: I think that there frankly should be ummm kind of like a questionnaire not (sighs) doesn’t have to be 5000 pages because obviously a parent sees that and they’re like oh god

**I: Mhm.**

P: But a form that you could attach then things behind

**I: Mhm**

P: that umm in preparation for your visit

**I: Mhm**

P: so that when you already go into your visit it’s not like you’re starting them to have to start to think about this whole thing. I mean I know that’s usually the way it is though. You know you make an appointment to see a doctor for the first time, and then you just talk about it all when you’re there.

I: Mhm

P: But I find sometimes that to have to explain everything over again it’s–it’s… I wish there were just like a simple questionnaire

**I: So that would capture everything. So, they would have it permanently, you wouldn’t have to repeat yourself.**

P: I wouldn’t have to repeat myself and then maybe it could be a universal one.

**I: Mhm**

P: I don’t know, it’s the one that (hospital 1) has, the same one that (hospital 2) has..

**I: Mhm. Okay. So, I’m going to show you ummm we don’t have the answer, this is work that’s being done**

P: Yeah

**I: but I’ll start up with this as an example. So, these are questions that are actually asked for the U.S. census. So…**

P: See I love the first one already and I love the second one.

**I: Okay. So, so yeah. If you want to take a look. So I guess the question is how yeah how do you feel on this, do these capture what you would want to share and what would you change or add to–to make sure you’re sharing with the healthcare provider whatever you think they need to know?**

P: Okay. (reads paper). These are six good ones.

**I: Mhm**

P: Ummmm

**I: Yeah would you say that those capture everything that you would want to convey initially?**

P: I’m trying to–I’m trying to think

**I: Yeah, mhm.**

P: But again, I look at this and am I looking at this as a mother of my child or am I looking it as me?

**I: Well it would be for the–the individual specifically**

P: So individual. So I look at this and I say okay is–is (daughter) deaf and has a serious? Yes. Is she blind or has difficulty seeing? Uhh, you know, that’s where I would see it.

**I: Mhm.**

P: You know

**I: yeah**

P: So this is IDL’s and ADLS’s as they call it.

**I: Right, right.**

P: Yea so these six are…yes, a good a good beginning, yes

**I: Okay. And tell me, you know, if this is a good beginning are there any other perhaps, you say you checked one of these, is there anything that kind of strikes you as being a necessary follow up question to kind of capture any accommodations for appropriate healthcare services for example?**

P: Oh god, if I had a day to think this over, I swear I would be able to really…

**I: mhm.**

P: Ummm. So, I–I uhhh

**I: And I’d be happy to share this with you if you if you actually want to–to do that thought exercise**

P: Yea, and ummm, you know it’s funny we–we have a good mom's group

**I: Mhm**

P: Uhhh they’re all, again, and it’s all these children that go to where my daughter, where my younger daughter’s going

**I: Mhm**

P: And we all have these questions. We all say to each other, you know like, we’ll say what is your experience going to the doctor? Did they ask you enough questions? Why aren’t they asking you that questions? Since I’m the eldest mother, I’m the one with the eldest child, they are always looking to me

**I: Mhm**

P: for answers. So, they’re always asking me, why isn’t the doctor’s offers uhh office not asking me for more accommodations or more uhh….

**I: Mhm. Mhm.**

P: But this is a good ummm I swear I could give you. I mean I feel like I’m (inaudible) pressure

**I: No, No, No**

P: I swear if I had more time I would probably give you at *least* another

**I: Mhm.**

P: Another two that would be of...

**I: Are you okay if I if I email this to you?**

P: Yes! That would be wonderful.

**I: Okay. Okay, I’ll do that**

P: And I will give you more feedback, I really will. Because we discuss this at every time we meet

**I: Mhm**

P: Ummm yea there’s uhhh I’m telling you there should be one more…do you need a–a an advocate here with you today, you know, do you have an advocate here with you today? Or uhhhh do you have a legal guardian? Do you…I don’t there–there could be something…so that it doesn’t look like also that they were going to take advantage of a

**I: Mhm.**

P: Okay, I like this though

**I: Okay, okay. Yeah so I will follow up with you on this.**

P: Yes, please, Definitely. I like it.

**I: So, so I mean that’s the gist of it so that would be, you know, a universal, as you said, something universal in preparation for visits that would be always available so you wouldn’t have to repeat yourself other than update anything that changed. Is that how you see it?**

P: Yes, yes, yeah.

**I: Okay. Umm and that’s pretty much all my questions and do you have unless you have anything to–to add**

P: You know, I–I think you know something, I have to say to you, thank you for taking your time for
