# Supplementary material for: Disability disclosure in healthcare settings for individuals with developmental disabilities: A qualitative study of patient and caregiver perspectives
Source: PLoS One. 2025 Aug 7;20(8):e0329328. doi: 10.1371/journal.pone.0329328 (PMC12331114; doi:10.1371/journal.pone.0329328)
Supplement: S1 File — (ZIP) [file pone.0329328.s001.zip › Transcripts/2019.09.06 Interview 06 Transcript.docx]

**I:** Interviewer, **F1:** Female Key Informant

1. **I: I’m hitting record now.**
2. **F1:** Okay.
3. **I: Uh. Just for the record um will you um you…you…we went over the informed consent form.**
4. **F1:** I give consent.
5. **I: And you give consent. Thank you. Alright, so let’s jump in. So, um, in general, um you have been to various uh healthcare um providers like a doctor or something else like that?**
6. **F1:** Yeah.
7. **I: Yeah? Um, would you say your experiences, that you’ve had good and bad experiences? Just good? Just bad?**
8. **F1:** Uh, overall, it’s annoying when I get there, and they ask, they ask me “am I with somebody else?”
9. **I: Okay, they, so that’s a common thing that they ask you that, that they ask if you are with someone else?**
10. **F1:** Yeah, they ask “who are you with?”
11. **I: So, they kind of make that assumption that you need to be with someone, you’re saying?**
12. **F1:** Yeah, yeah.
13. **I: Why do you think that they, they ask you that?**
14. **F1:** Because of the way that I sound.
15. **I: Okay. And, what do you mean by that? How do you sound?**
16. **F1:** I have Cerebral Palsy. It affects the speech and they assume that because the way, the way that I talk, um, they assume that um I lack intelligence.
17. **I: Okay, so they think then that you lack intelligence?**
18. **F1:** Yeah.
19. **I: Mhm. And how do you respond when they ask you that question?**
20. **F1:** I get a little bit annoyed (Laughter) because then they, then I have to re-reassert myself
21. **I: Mhm.**
22. **F1:** And I answer them slowly.
23. **I: You have to reassert yourself?**
24. **F1:** Yeah.
25. **I: So, how, how do you do that? Like what do you say? I mean, you say you get upset or annoyed and in general, but how do you respond to them beyond that emotion? Like, do you show them that emotion? Do you say anything to them?**
26. **F1:** I used to, I used to but now, no I don’t.
27. **I: Okay.**
28. **F1:** I don’t anymore because, because that is their ignorance not my ignorance.
29. **I: So, you see it as more their issue and not your issue, so, so you don’t respond to them anymore you just what you just kind of…**
30. **F1:** I just answer them. I am by myself.
31. **I: Mhm. So, you just say I am by myself. You don’t bother engaging them any other way?**
32. **F1:** No, I don’t.
33. **I: What did you, what did you do in the past then?**
34. **F1:** Well I needed to get the why? Why did I need? Why do you think that I need?
35. **I: Mhm.**
36. **F1:** Now, I don’t do that anymore.
37. **I: So, when you did ask them why do you think I need someone here with me, like how, like how would you say they responded to that?**
38. **F1:** They didn’t …they weren’t honest…they didn’t. They do not know how to respond to that.
39. **I: They weren’t honest, or they didn’t know how to respond. So, if they didn’t know how to respond, did they just kind of would not say anything, they would brush it off?**
40. **F1:** They did not say anything. Yeah, they did not say anything.
41. **I: Okay. Do you think that they realized what you were trying to communicate to them when you asked that question?**
42. **F1:** In that moment, at that moment I, I didn’t really care what they were thinking.
43. **I: Ah.**
44. **F1:** I would nod. Yeah.
45. **I: You just wanted yeah, like to make it clear like not okay.**
46. **F1:** Yeah.
47. **I: Okay, are there any other things you can think of that made your healthcare experience less than, than what we would call good quality or high quality?**
48. (Phone Interruption)
49. **F1:** No, nothing bad, nothing.
50. **I: If you need to take that, by all means.**
51. **F1:** No, it’s just my sister.
52. **I: (Laughing)**
53. **F1:** They doing, they do this. My brother in law, he is cooking everyone food.
54. **I: Oh so you get to tell him what you want and how much you want?**
55. **F1:** Right.
56. **I: (Soft Laughing) Is he a good cook?**
57. **F1:** Yeah!
58. **I: Oh, okay. nice alright.**
59. **F1:** Yeah. But, um, the question was “what would make my experience better?”
60. **I: Yeah, are there, are there any instances you can remember, examples that where you had maybe a, a not so good healthcare experience for whatever reason?**
61. **F1:** I remember one time. (pause) The, the first time I went to my physician down the street um.
62. **I: I’m sorry, that is who down the street?**
63. **F1:** The, the doctor’s office.
64. **I: The doctor’s office?**
65. **F1:** Yeah, that I would go to.
66. **I: Okay.**
67. **F1:** Um, the doctor. I would do the, the test and they would, and they wouldn’t even go over the results.
68. **I: So, this is after the test going over the results? Okay.**
69. **F1:** Yeah, yeah. And the doctor, he was looking down and not looking at me when they respond.
70. **I: He was looking down instead of looking at you when he responded to you?**
71. **F1:** Yeah.
72. **I: Okay.**
73. **F1:** He would tell, tell that the, the tests were done, and he was like ma’am it looks like that you have cancer. I have cancer. The way that I feel is that you would not going to give someone bad news like that. You should at least look right at me and turn towards me when you are telling bad news. Cancer is a big diagnosis.
74. **I: Do you think he was looking down because he was giving bad news, or do you think it might have something to do related to having Cerebral Palsy? Or?**
75. **F1:** I don’t know. At that time, I really didn’t even care. I had cancer. And the way that it was delivered it wasn’t (inaudible).
76. **I: Mhm.**
77. **F1:** At that point, I didn’t really look at it that way.
78. **I: Sure.**
79. **F1:** That it was because of CP. Eventually, the way that he would look at me, the way he looked was insensitive.
80. **I: Insensitive right? Because he was looking down instead of at you.**
81. **F1:** Yeah, yeah.
82. **I: And is there anything that you did in that moment to let him know that, that wasn’t the best way, or it just was what it was?**
83. **F1:** It just was what it was. Because what was he gonna do? Tell me that I don’t have uh cancer? It was hard. And he was looking like and that was really, I didn’t really hear anything else.
84. **I: Right, so it was just taking that knowledge in it was–**
85. **F1:** Right, yeah.
86. **I: Mhm, Mhm.**
87. (Pause)
88. **F1:** It was all right. But then I had the condition that was this. My main focus, my main focus was what…
89. **I: Your main focus was what he was telling you, not how he was telling you?**
90. **F1:** Yeah, yeah, yeah.
91. **I: Right.**
92. **F1:** Yeah, yeah.
93. **I: Mhm.**
94. **F1:** Yeah.
95. **I: So, do you still go to that doctor?**
96. **F1:** I still go; I do not see that doctor.
97. **I: Okay.**
98. **F1:** But I think, I think afterwards I bring it up.
99. **I: You actually brought it up later?**
100. **F1:** Yeah, yeah, yeah!
101. **I: Okay. And how did that go?**
102. **F1:** I don’t, I don’t know…Um, for me it really doesn’t matter how it goes down at all.
103. **I: Uh huh, so is it maybe about how, like, how are you doing it for yourself, not like to teach them or correct them?**
104. **F1:** At, at that time, I want, I want them to know that. I want the supervisors to know what is going on because they might help next person that comes around.
105. **I: Right…maybe if it wasn’t good for you, it’s good for the next person.**
106. **F1:** Right, right, right…so…I…(coughing)
107. **I: So, when you say you did it more for you, what do you mean by that?**
108. **F1:** I mean….um, like, um well I mean, what did I mean by that? I think I mean that like as long as I get my, my point across, I think that is fine.
109. **I: That’s satisfying to just communicate.**
110. **F1:** Yeah.
111. **I: To just communicate to them how you felt.**
112. **F1:** Right, yeah.
113. **I: Cause you think, that is it, that it just makes you feel better or you think that it helps them be better or?**
114. **F1:** It helps them be better I hope, I hope.
115. **I: Alright.**
116. **F1:** I hope, yeah. I mean you hope, you know/
117. **I: Mhm, and when you, when you communicated that information to him, did he respond in any particular way?**
118. **F1:** I mean, I don’t recall, I don’t recall um I…I think I said this to the supervisor.
119. **I: Oh, you told the supervisor?**
120. **F1:** Yeah.
121. **I: Not him directly.**
122. **F1:** Yeah.
123. **I: How did, how did the supervisor respond?**
124. **F1:** They were very receptive.
125. **I: Mhm, so they just accepted the information, they didn’t tell you anything they were going to do differently, or speak to him or anything like that?**
126. **F1:** No, no.
127. **I: Yeah. Okay, so beyond that, any other things that you can think of that have, have made healthcare experience unpleasant?**
128. **F1:** I don’t have a lot of experience here it’s just that when I want because I do not go to doctor that much.
129. **I: You don’t go to the doctor often?**
130. **F1:** No.
131. **I: Or?**
132. **F1:** I only need to go to the doctor once a year.
133. **I: Once a year.**
134. **F1:** Or twice a year.
135. **I: Okay, once or twice a year.**
136. **F1:** Yeah, yeah.
137. **I: Okay.**
138. **F1:** I don’t have.
139. **I: Not a whole lot of experiences.**
140. **F1:** No.
141. **I: Mhm, okay…when…when someone in the healthcare setting whether it’s the doctor or the front office desk or the nurse, um, first interacts with you, um, how do they, obviously you said they sometimes make an assumption asking if you are with anyone, but -**
142. **F1:** Yeah.
143. **I: But, how else do they respond to you when they hear you speak?**
144. **F1:** Um um (laughs)… ummm...they, they seem, one example right? I go and I say “good morning” and they do not understand
145. **I: Mhm**
146. **F1:** They turn away or they do not look.
147. **I: So, they make a face or look away?**
148. **F1:** Yeah, I think if they do not understand, they do not ask again.
149. **I: So, they do not ask clarifying questions.**
150. **F1:** Right, right.
151. **I: They just kind of roll with it.**
152. **F1:** Right
153. **I: If they don’t know.**
154. **F1:** I…I know when they do not understand what I am saying.
155. **I: Mhm.**
156. **F1:** So, I…when, when they do not…I ask indirect question.
157. **I: Mhm.**
158. **F1:** So, I know when, when they not paying attention.
159. **I: So, they like, do you like, for example, you ask a question and they just don’t answer it?**
160. **F1:** Yeah, right. And then they do, they, they say something different other than what I had asked.
161. **I: Okay so…okay, so, they, they’re responding with incorrect information or something totally unrelated because they didn’t actually hear you.**
162. **F1:** Yeah, they didn’t, well they *hear* me.
163. **I: But they didn’t understand you.**
164. **F1:** Right, they will say that they do not hear me.
165. **I: Okay.**
166. **F1:** But I know it’s that they didn’t understand what I said.
167. **I: Mhm.**
168. **F1:** Or let me ask it in a different way.
169. **I: Mhm.**
170. **F1:** Yeah.
171. **I: So, tell me in those instances, have you ever, has it, has it ever been problematic as far as not getting the care you needed?**
172. **F1:** Yeah! Yeah! Yeah! Yeah!
173. **I: Or…Tell me more about that.**
174. **F1:** Yeah. (Pause) well, um, this is nothing. I went into the dentist. And said I have a place, one of my teeth it came off.
175. **I: One of the fillings came out?**
176. **F1:** Yeah.
177. **I: Yeah**
178. **F1:** Yeah and I went right. And right when we go. When, when the, the tube, right, it was there. I wanted to know what they were for. They come in walking with whatever I had.
179. **I: Mhm.**
180. **F1:** I get there; I had to get up early that morning because of the walking. Because it is noisy (unclear, 12:01)
181. **I: Right.**
182. **F1:** So, I get up early that morning, riding STS.
183. **I: Mhm.**
184. **F1:** They start to take a long time (unclear 12:14). And…
185. **I: The STS how they go around and around waiting for you? Or waiting for the…bus to pick you up?**
186. **F1:** No, no.
187. **I: To pick up people? Oh, okay, cause it’s going on a route.**
188. **F1:** Yeah. It has. That was really annoying because, because I could’ve with the time, I was taking the bus been there early. I was getting there the early**.**
189. **I: Mhm.**
190. **F1:** And then I had to wait outside and inside the lobby for like a half an hour to an hour.
191. **I: Mhm.**
192. **F1:** I get, I get in there in time and they call me., I sit in chair, get there (inaudible) My tooth. I have the tooth and I need you to put it back on.
193. **I: Right.**
194. **F1:** So, they do not do that there.
195. **I: So, they told you to come for a service that they don’t provide.? Okay.**
196. **F1:** I was…
197. **I: Oh no!**
198. **F1:** Yeah, yeah. That *is***,** I don’t know what that was a lie or a difference. That could be a different
199. **I: Do you think that might have been why that happened was that they asked you to come even though they can’t help them**
200. **F1:** But I told them what it is that I need *then*.
201. **I: Right, and they should have known to say “hey we don’t do this here,” but you wasted all that time with STS getting there waiting just to find out that they can’t help you. Yikes. Is there, are there any other instances that are like that too where communication kind of broke down where they didn’t understand and didn’t ask follow up questions?**
202. **F1:** Yeah, when…when I go to, to, to my doctor.
203. **I: Mhm.**
204. **F1:** I get a prescription for OT and PT**.**
205. **I: Mhm.**
206. **F1:** Yeah, I was there. I told them that I need the prescription.
207. **I: Mhm.**
208. **F1:** And that I was all out. And they had me give them the referral. And they had me do the put what I said and what I need. saying of what I needed
209. **I: So, put what on the referral?**
210. **F1: How many weeks.**
211. **I: Just the details, of how like often, how long, all that stuff?**
212. **F1:** Right.
213. **I: Mhm. And they don’t do it. Okay**
214. **F1:** Yeah, that, that is frustrating.
215. **I: Yeah.**
216. **F1**: Yeah, that and another thing that frustrates me...
217. **I: Mhm.**
218. **F1:** For me is that…one of the times I called...
219. **I: Mhm.**
220. **F1:** No, one of the times, I made an appointment. I made an appointment. When it got cancelled, they give me, they think that I am free. They called and cancelled it and said the doctor is not in. and they think I can do it whenever time they want me to come.
221. **I: So, it’s on their schedule not yours. So, they want their schedule not yours.**
222. **F1:** Right. I do not have nothing to do. I don’t have.
223. **I: Yeah, like no one has got anything to do, it’s always for them.**
224. **F1:** I, they are the office that I go through. They say I can come in the day, and I say I have to check my schedule.
225. **I: Mhm.**
226. **F1:** They do not believe that I have this.
227. **I: So, they make an assumption then that you, that you couldn’t possibly need–have a schedule? That you have a social calendar, for example.**
228. **F1:** Right! Right! Right!
229. **I: That assumption…why do you think that they make that assumption?**
230. **F1:** Because they deal with other people with developmental disabilities that have no, that whatever time they call them, it will be difficult. Other people are in charge of them. They take, they take them there.
231. **I: So, they see other patients with developmental disabilities don’t have a schedule, so they assume the same is true for you.**
232. **F1:** Right.
233. **I: Okay.**
234. **F1:** Right, yeah, that frustrates me a lot.
235. **I: So, it’s kind of lumping you into a category rather than learning specifically about you.**
236. **F1:** Right, right.
237. **I: Okay.**
238. **F1:** That frustrates me because even when they, when I apply for STS at the interview. I know that they were going to deny me at first because, because he reads out the list and the thing that people do.
239. **I: STS is going to deny you?**
240. **F1:** No, I know that they did deny me!
241. **I: Okay, they did at first. Okay.**
242. **F1:** Yeah, because when, when, when the lady interviewed, we got the list and it said yes to all of them. She…I guess she was (inaudible) because…because she think that I didn’t understand everything, know, know that I am not (inaudible) about it, but she thought, she thought that I was and I didn’t understand what he was telling–so when I said yes to everything under the list.
243. **I: So, she didn’t believe that it was going to be yes to everyone. She just thought that you didn’t understand it and so she didn’t trust you.**
244. **F1:** Right, right.
245. **I: Okay.**
246. **F1:** Right.
247. **I: So how did, how did you fix that issue? (Laughter)**
248. **F1:** (Laughter)…they denied me! They said no!
249. **I: Mhm.**
250. **F1:** I mean that was one thing nobody tells me no.
251. **I: Mhm.**
252. **F1:** And I ask that when I know that I deserve, and I know that I deserve it.
253. **I: You know that you deserve it, mhm.**
254. **F1:** Yeah, nobody tells me no, and they did…they denied me.
255. **I: Okay, so you, so you usually don’t get told no. You usually…**
256. **F1:** No, I *get* *told* *no*.
257. **I: But you don’t let it go.**
258. **F1:** Right, right.
259. **I: You get the yes.**
260. **F1:** Until you tell me *why* you said no.
261. **I: Right, so you explain, explain to me why the no?**
262. **F1:** Right!
263. **I: And you have a conversation.**
264. **F1:** I do not accept that, like, the beyond, beyond why she cut me out the first time. Right?
265. **I: Mhm.**
266. **F1:** And she hadn’t said to me why she cut me out
267. **I: And then you were like, “no I actually do understand.”**
268. **F1:** Yeah, right! Right!
269. **I: So, then she believed you after that, after you explained it to her?**
270. **F1:** No, not explained. I didn’t. I, I go around them.
271. **I: Oh, you actually went around them?**
272. **F1:** Yeah, my therapist, my OT. She was downtown, we went down together.
273. **I: Oh, okay, so with the help with your OT?**
274. **F1:** Yeah**.**
275. **I: You got them to reverse their decision.**
276. **F1:** Yeah.
277. **I: Okay, so that’s a positive healthcare experience. Okay.**
278. **F1:** Right!
279. **I: Okay, okay. So, so, tell me, you initially said that sometimes they ask you the question are you with anyone. Do you tend to go to doctor appointments or any healthcare appointments on your own or with someone?**
280. **F1:** By myself.
281. **I: By yourself.**
282. **F1:** Yeah, only when I am… (subtle laughter) getting a shot. I have a phobia.
283. **I: Okay, so when, so you’re saying when you get a shot, that’s when you go with someone? Or?**
284. **F1:** Yeah.
285. **I: Why, why is that? Are you afraid of shots?**
286. **F1:** (Laughter)…yeah, I am afraid! I’m afraid.
287. **I: Yes?**
288. **F1:** Yeah, it’s a phobia.
289. **I: Oh? It’s a straight up phobia.**
290. **F1:** Yeah, I know it’s a phobia.
291. **I: Okay, so it’s serious?**
292. **F1:** Yeah, yeah. I freak out. Yeah, next week, I have to go to the dentist. Going to get a teeth cleaning and -
293. **I: So, that’s another? Do they give you a shot for that? Or?**
294. **F1:** Yeah, yeah.
295. **I: Okay, and, so, who, that person that goes with you is, is supposed to do what? Calm you down? Or what does that person do when they are with you?**
296. **F1:** (Laughter) Hold my hand.
297. **I: Just hold your hand? You squish their hand.**
298. **F1:** I, I get really freaked out.
299. **I: Okay, okay.**
300. **F1:** Yeah.
301. **I: So, in those instances when you’re with someone, um does the dynamic of how you communicate with the healthcare provider change?**
302. **F1:** Yeah!
303. **I: Can you talk about that?**
304. **F1:** Yeah, definitely, definitely. So, whenever anytime I go with anyone, whoever it might be like my sister. Not only for the shot.
305. **I: Mhm.**
306. **F1:** But it makes my sister come with me and we go afternoon and we go after…
307. **I: Mhm.**
308. **F1**: Afternoon. The doctor always give a way (unclear 21:25).
309. **I: Mhm.**
310. **F1:** I thought after they come with me, right?
311. **I: Mhm.**
312. **F1:** They, they want to see that doctor. Once they see that person, they talk that person.
313. **I: So, they talk just to your sister rather than to you?**
314. **F1:** Yeah, yeah.
315. **I: Okay.**
316. **F1:** Yeah, so, so I do not let my sister come.
317. **I: And that’s why, so that’s why, so that’s why you go alone because you are avoiding that?**
318. **F1:** No, not why. But once I went to a restaurant one time.
319. **I: To a restaurant? Okay**
320. **F1:** Yeah, and that happened. And I told my sister, no more, you don’t, you don’t talk for me no more.
321. **I: You don’t talk for me.**
322. **F1**: Yeah, anymore. Because what she tends to do, she says talk to you.
323. **I: So, she actually proactively says stop talking to me, talk directly to her. Is that the same in the healthcare setting too?**
324. **F1:** Yeah! Yeah, yeah.
325. **I: Mhm, okay. And how do they respond to that? Do they just start talking to you then?**
326. **F1:** Yeah, what I think they do is that sometimes she stays outside while I am inside.
327. **I: Okay.**
328. **F1: Yeah.**
329. **I: Only for the shots. So, she stays outside unless there is a shot, and then she comes in and holds your hand?**
330. **F1:** Yeah, yeah.
331. **I: Okay.**
332. **F1:** Yeah, but that, that doesn’t happen. That does not happen in most cases, not what happened throughout my life. One time-
333. **I: Throughout your life…**
334. **F1:** Yeah. That’s why I went to the supermarket.
335. **I: Mhm.**
336. **F1:** And I, I had a man cut the flowers. He understood what I said, right.
337. **I: Right. He understood.**
338. **F1:** Right. Then my sister, my niece, my nephew came over. Only talked to her.
339. **I: Mhm, and not to you?**
340. **F1:** Right!
341. **I: Okay.**
342. **F1**: Right. So, yeah. I understand that that is going to happen.
343. **I: Mhm.**
344. **F1:** But it’s still annoying.
345. **I: Yeah. It’s annoying that they are kind of ignoring you.**
346. **F1:** Yeah, yeah.
347. **I: Mhm. And do you think - So, have you ever had a situation and it was like someone that you still know and, and the dynamic of the relationship has changed where now they do talk to you, and the, they maybe told you why they did that in the first place? I don’t know if that made sense, I can repeat myself.**
348. **F1:** I understand, I understand.
349. **I: Okay.**
350. **F1:** I understand it. But here is someone that I think that is going to accept it.
351. **I: Mhm.**
352. **F1**: And tried to change their behavior and I will tell you about them. I feel that they do not, they’re not willing to accept it and now -
353. **I: It’s about whether they are willing to accept it or not.**
354. **F1:** Right, right. Right, it depends on whether you’re open to–
355. **I: Right.**
356. **F1:** Hearing certain things. And in my – well you said have I ever addressed certain things to certain people and now I have a different openness. Yeah, but, but they weren’t really ready to hear it.
357. **I: Mhm.**
358. **F1:** They weren’t ready to change their behavior.
359. **I: Right, they weren’t ready to change.**
360. **F1:** Yeah, maybe because their response was “Oh, I didn’t mean it like that”
361. **I: They didn’t mean it like that?**
362. **F1:** Right.
363. **I: Okay.**
364. **F1:** So, that means that they acknowledge the accident or what they’ve done or how I see it.
365. **I: So, they acknowledge, and they know what they were doing or not doing.**
366. **F1:** Right, they just said that they weren’t, they weren’t, they never meant it like that.
367. **I: So, they meant no harm.**
368. **F1:** Right, right. But they do again.
369. **I: So, in essence, do you think that they, and this might be different for different people, but what is your general thought, do you think they avoid talking with you because they don’t think that you’re going to understand or they don’t think that they are going to understand you?**
370. **F1:** I think, I think they are worried because they do not want to ask, ask me to repeat myself.
371. **I: Okay, so they don’t want you to have to repeat yourself?**
372. **F1:** Right.
373. **I: And that’s okay.**
374. **F1:** I think that they are more afraid that, that like the taboo that they do not talk about certain things.
375. **I: So, they’re afraid that they are going to talk about something taboo that they’re, they shouldn’t.**
376. **F1:** Right.
377. **I: So, like they’re not allowed to talk about Cerebral Palsy? Or?**
378. **F1:** Right, disability in general.
379. **I: Okay, disability in general. Okay, okay. Do you think that is a taboo topic?**
380. **F1:** Yeah, yeah.
381. **I: Okay, do you think that it should be a taboo topic?**
382. **F1:** No.
383. **I: Or just that it, it shouldn’t be, but it is.**
384. **F1:** Right, it still is a taboo topic for certain people.
385. **I: Mhm.**
386. **F1**: I am a part of this world.
387. **I: You’re a part of this world.**
388. **F1:** Yeah. People with, people with disabilities no matter what the disability is, we are a part of this world.
389. **I: Right.**
390. **F1**: And we deserve, we deserve to, we have rights. We were given them. We have rights and people without disabilities should see give us the opportunity to, to fulfill our abilities.
391. **I: Sure! So, you have rights and you should be able to fulfill your abilities.**
392. **F1:** Right!
393. **I: Absolutely.**
394. **F1:** No matter what the limitation is, we…I am getting...(tearful)
395. **I: You’re getting emotional?**
396. **F1:** No, not emotional. Well, emotional but, but in a way that I…in a way that is my…our emotion, in a way that is in anxiety**.**
397. **I: Mhm.**
398. **F1:** In a …
399. **I: In an enjoyable way? Or?**
400. **F1:** No, in an anxious way.
401. **I: In an anxious way, okay. Well I don’t want to make you feel anxious.**
402. **F1:** No, not you definitely. I understand it.
403. **I: Okay. Mhm. So, um, in terms of healthcare experiences we talked about, you know, when another, uh, person is with you in the room, um, are there any other things that stand out that just weren’t ideal, um, in those communications and that could be direct communication with a healthcare provider, that can also be as far as information they give to you after an appointment like educational information or the, or you mentioned the prescriptions, um, anything like that that kind of gets in the way?**
404. **F1:** Um…
405. **I: Or, you got it all?**
406. **F1:** For, for me, it, it, it. I really don’t think they talk, they, I think that when they say they do not hear me, I clarify that they do not understand what I am saying.
407. **I: So, you make it clear like “no no no, you hear me, you just aren’t understanding me.”**
408. **F1:** Right**!**
409. **I: Okay.**
410. **F1**: And then they, if I, if the, if who I am communicating with do not try after that they do not stop what they’re doing and look at me and give me the attention.
411. **I: Mhm.**
412. **F1:** Then, I know I don’t bother.
413. **I: You don’t waste your time if they kind of don’t give their time and attention and eye contact to listen.**
414. **F1:** Right, I go after that, after I see the doctor, I have a supervisor.
415. **I: So, you usually. So, you are good at giving regular feedback when you encounter those situations?**
416. **F1:** Yeah, yeah.
417. **I: Okay, is there anything, you know, we are talking about doing something after the fact, is there anything you do before the appointment to try to prevent any possible bad experiences?**
418. **F1:** Yeah, yeah with the needles.
419. **I: The needles. Well obviously,, bringing someone with you for needles.**
420. **F1**: No. If I cannot get anyone to come with me, I told them look, I need.
421. **I: You tell them, look I need somewhere there with me**
422. **F1:** No, I tell them that I cannot be in the room with the needles.
423. **I: So, you tell them I can’t even have needles in the room?**
424. **F1:** Yeah. I blindfold myself.
425. **I: You blindfold yourself before a shot?**
426. **F1:** Yeah! Yeah.
427. **I: You do what you got to do.**
428. **F1:** Right.
429. **I: Yeah.**
430. **F1:** Right, I know myself that I’m going to freak out and then I, then I cry.
431. **I: So, it’s yeah, you said phobia, so that’s serious.**
432. **F1:** I cry every time.
433. **I: Mhm.**
434. **F1:** And then I get annoyed that I have to have it.
435. **I: Mhm.**
436. **F1:** I will myself.
437. **I: You worry yourself?**
438. **F1:** I will myself
439. **I: Oh, you will yourself. You prepare yourself. You will and you worry probably.**
440. **F1**: (Subtle Laughter) Yeah, yeah.
441. **I: Okay.**
442. **F1:** Yeah, that is what I do.
443. **I: Mhm… So, so, tell me, I mean, when, when you first meet a healthcare provider and they, they hear you talk, do they ask you questions about, I mean, or do you tell them that you have Cerebral Palsy? Like how does that, how does that taboo conversation happen?**
444. **F1:** They ask. They ask, “what is your condition?”
445. **I: Okay.**
446. **F1:** And I tell them what I have, and then go through this long talk about telling them what it affects.
447. **I: Well, how and what it affects?**
448. **F1:** Yeah.
449. **I: Do you find that they’re pretty knowledgeable about Cerebral Palsy? Or?**
450. **F1**: No!
451. **I: Not at all?**
452. **F1:** No, not really
453. **I: Or a little bit?**
454. **F1:** Not really.
455. **I: Okay, not really, okay.**
456. **F1:** I, I think that what it always is they know what it is, but they do not know that it affects people in different ways.
457. **I: So, they don’t know the diversity of the condition.**
458. **F1**: I don’t think so.
459. **I: Like across different people.**
460. **F1:** That is what in all of, in every occasion that I meet.
461. **I: In everyone that you meet? Yeah?**
462. **F1:** Yeah. They do not understand that it is different for different people.
463. **I: Different for different people. So, you said that you kind of explain it to them specific to you?**
464. **F1:** Yeah.
465. **I: Okay, and what, um, how?**
466. **F1:** But, they still don’t.
467. **I: What?**
468. **F1:** They still don’t, they forget.
469. **I: They still make the assumption “are you with someone?” Um, how I guess, um, do you feel like they are receptive to that information that you share with them?**
470. **F1:** Not really.
471. **I: Not really? Tell me about that. Why do you think that they’re not receptive?**
472. **F1:** Because they do again after.
473. **I: Because they keep on making the assumption? Yeah?**
474. **F1:** Yeah, they do it again.
475. **I: Mhm, and do you think it’s more that they just forget because they only see you one to two times a year? Or?**
476. **F1:** Maybe.
477. **I: But it is probably in your medical record.**
478. **F1:** Yeah, that’s right!
479. **I: They should see it in your file, you think?**
480. **F1:** Right!
481. **I: Okay, but they still keep on doing it.**
482. **F1:** Yeah, yeah.
483. **I: So, have you ever stopped going to a healthcare provider because they make that assumption, or do you still just go to them anyway?**
484. **F1:** It’s *right* there...
485. **I: So, it’s close and convenient.**
486. **F1**: It’s walking distance.
487. **I: Okay.**
488. **F1:** So that is a bonus. The only reason why I, I go.
489. **I: So, if, if you, if there was a close by option you would probably switch and try that one instead?**
490. **F1:** Yeah, yeah.
491. **I: Okay.**
492. **F1:** It’s right there.
493. **I: Mhm.**
494. **F1:** So…
495. **I: So, it’s close.**
496. **F1:** I walk.
497. **I: Can’t argue with walking distance, right?**
498. **F1:** Right.
499. **I: Mhm, okay. So, um, do you think, um, do you have any thoughts other than what we’ve already talked about the understanding and communication issues? Any other things, um, that, that having Cerebral Palsy have been related to in terms of impacting the quality of healthcare you receive?**
500. **F1:** Well, I think other than, not only Cerebral Palsy. It’s just that other, other people’s disabilities need to be treated with dignity and respect.
501. **I: *Need* to be treated with? So, you’re suggesting that that’s not the case.**
502. **F1:** No.
503. **I: Not being treated with dignity or respect.**
504. **F1**: No. That is not the case because they make the assumption of that. I see, I see whenever I go to other places.
505. **I: Mhm.**
506. **F1:** But whenever I go with my sister to other appointments, uh, ask someone else and you know one, one disability you learn like other disabilities.
507. **I: Mhm.**
508. **F1:** That is all that they see.
509. **I: So, they only see your disability. After, after they learn them, they don’t see anything else.**
510. **F1:** No.
511. **I: Mhm. Do you, have you, do you feel like, you know you said they might know *of* Cerebral Palsy, but they don’t know how it affects people in different ways? I mean, do you ever find, you know we talked about *this* assumption, but do you think that there’s any other assumptions they are making so they like lump you into a category they know about Cerebral Palsy so that it might apply to you or anything like that?**
512. **F1:** Yeah. People, they think not that having Cerebral Palsy do not exempt you from having cancer, having other diseases, diabetes, yeah.
513. **I: It doesn’t exempt you from those other diseases. Right?**
514. **F1:** Right, and it doesn’t exempt you from, um, going to, to school the way everyone does.
515. **I: School or anything else you do, mhm.**
516. **F1:** Right, to go to the store, the grocery.
517. **I: The grocery?**
518. **F1:** Today, they see, and for me, I know I don’t care as long as I get what I need.
519. **I: Right.**
520. **F1:** I don’t care what their assumptions you make about me
521. **I: Right. So, you don’t care. As long as you can do what you want to do.**
522. **F1:** As long as I get what I need from that provider. I don’t leave until I get what I need.
523. **I: So, so you, you are, are more like empowered to force the issue to get what you need -**
524. **F1:** Yeah.
525. **I: -even if you’re not in the first place getting it.**
526. **F1:** Right.
527. **I: Okay.**
528. **F1:** Even if they, if they have certain view. And you assumed that I do not have the mental capacity to make decisions. As long as you are good, you tell me to what it all is.
529. **I: Mhm.**
530. **F1:** I don’t care.
531. **I: So, even if they don’t think that you have the mental capacity, as long as they are helping you, you’re fine with it.**
532. **F1:** Right.
533. **I: Mhm.**
534. **F1**: I, as long as I, I told you, you know that there is the treatment in certain.
535. **I: Right, so as long as you are being treated with dignity and respect, that’s okay.**
536. **F1:** Right.
537. **I: Okay, so, you don’t think there’s been any other incidents where you were treated differently or not receiving all the treatment or in the way you’re supposed to be receiving because of them seeing you as someone with a disability?**
538. **F1:** Yeah.
539. **I: Mhm.**
540. **F1:** But, but that, I think for me, it’s more like if, I do not go back there.
541. **I: You just don’t go back there?**
542. **F1:** No, they are not worth my, my, my self, my, patronizing me. I don’t want to see them again.
543. **I: Right, so you don’t want to be patronized or give them business.**
544. **F1:** Right, right. They are not patronizing me no more.
545. **I: Okay, okay. So, let’s, um, let’s flip the script and talk about like good experiences, like, what, like, what can you tell me about any experiences in the healthcare setting that have been positive?**
546. **F1**: Well, um…the other day, um no not the other day, last year, I went to, I went to Aventura and, I was with my niece, she is not tall.
547. **I: She’s a doctor in Aventura?**
548. **F1:** No, she is short.
549. **I: Oh, she’s short.**
550. **F1:** Yeah.
551. **I: Okay.**
552. **F1:** And, yeah. So, we went, knowing that she let me lead and I think, I think when she let me lead -
553. **I: Mhm.**
554. **F1:** That is a way when I find the best experiences.
555. **I: The best experience is when they let you lead? As in begin?**
556. **F1:** Yeah.
557. **I: Like in the conversation? Share whatever you need to share.**
558. **F1:** Yeah.
559. **I: Okay.**
560. **F1:** And they do not ask, they do not ask (inaudible). That was very good because -
561. **I: Mhm.**
562. **F1:** Because sometimes when I go other places, and they see that you are with someone here. Even if you are doing a, like a, puzzled look.
563. **I: A puzzled look?**
564. **F1:** Yeah, yeah, uh huh, and it, it acts like I am conversing with you.
565. **I: Mhm.**
566. **F1:** You, you wouldn’t pay attention.
567. **I: Alright, so you’re showing the intent with the willingness to pay attention so that they do understand?**
568. **F1**: Right! Right, Right.
569. **I: Mhm, so that makes it a positive experience?**
570. **F1:** Yeah.
571. **I: Mhm.**
572. **F1:** And the dentist office that I go to, I know the doctor is good at paying attention. But the front lady, no.
573. **I: The front office people…so the doctor does, but the front office ladies no.**
574. **F1:** Yeah, she, she, she don’t appreciate that I called and cancelled. But she gives me attitude.
575. **I: Wow, okay.**
576. **F1:** And she, she wants me to bring someone with me to reservation. Yeah.
577. **I: Mhm, okay, so what else would make for a good experience?**
578. **F1:** I think the doctor, the dentist was very good because he paid attention.
579. **I: Mhm.**
580. **F1:** He listened; he let me freak out.
581. **I: (Subtle Laughter) He paid attention, he listened and he let you freak out? Okay.**
582. **F1:** Yeah!
583. **I: Okay.**
584. **F1:** And then I blindfold my eyes.
585. **I: You blindfold yourself and then you, okay, uh huh.**
586. **F1:** Yeah.
587. **I: Is the actual pain a bad thing too?**
588. **F1:** No! Nothing.
589. **I: Or is it just the anticipation? Or the anxiety about what’s about to happen is, is the worst part of it?**
590. **F1:** Yeah, yeah, that’s right! That is why it’s a phobia.
591. **I: Mhm. Right.**
592. **F1:** The pain is nothing.
593. **I: Compared to everything else, right? Mhm. So, so, yeah, what, um, what do you, you know, what else might have, might help the healthcare experience? You mentioned before that the doctors don’t seem to know a whole lot about Cerebral Palsy other than what it is. Is there something that you would want to be different in that, in that sense?**
594. **F1:** Yeah. I think, yeah. I think, I think they should, um, take, take one per person and one, not just one -
595. **I: Mhm.**
596. **F1:** Like answers to one person for Cerebral Palsy and one for intellectual disability, you know?
597. **I: Oh, so like one person per type of disability.**
598. **F1:** Yeah.
599. **I: Okay, so that’s a lot of training.**
600. **F1:** Right!
601. **I: Uh huh, so, you think that would be beneficial if they got disability-specific training while they were still in school?**
602. **F1:** Right!
603. **I: Okay.**
604. **F1:** Right, because then, because when, when I learned that I have Cerebral Palsy, and will always (inaudible) found out when I was 20.
605. **I: Mhm.**
606. **F1:** And I know that it affects *me*, in a different way.
607. **I: Mhm.**
608. **F1:** Before, before, I didn’t know what it was.
609. **I: Right.**
610. **F1:** But I know that I have a disability.
611. **I: Mhm.**
612. **F1:** When I was 20, I learned that I have Cerebral Palsy, I was devastated.
613. **I: Mhm.**
614. **F1:** And I learned then that that’s the way it’s going to be.
615. **I: So. you were devastated when you found out?**
616. **F1:** Yeah.
617. **I: Why, tell me why, tell me more about that, why did you feel devastated?**
618. **F1:** Because it’s finally giving a name.
619. **I: So, they label it and …**
620. **F1:** Yeah.
621. **I: Okay. So, what did it mean to you to have a, a name associated with it?**
622. **F1:** After a while, I, when I read the definition, it makes it sound so bad
623. **I: (Subtle Laughter)**
624. **F1:** Then the perk is, the perk is, I get, I get all of these, these other things. The perk for me having Cerebral Palsy, I, I am attractive.
625. **I: The perk is being attractive?**
626. **F1:** No, no, that’s not the perk. The perk is, is that, I, I don’t, I’m attractive. When I open my mouth, it humbles me.
627. **I: Mhm.**
628. **F1:** It humbles me.
629. **I: Humbles you?**
630. **F1:** Yeah.
631. **I: Okay.**
632. **F1:** Because I am the oldest, the oldest of five girls.
633. **I: Okay of five girls, so you are the older sister of five.**
634. **F1:** Yeah, yeah and if I am. It’s a hard thing.
635. **I: Mhm.**
636. **F1: (Inaudible)** So, so if I was, if I was to be stuck up.
637. **I: Mhm.**
638. **F1:** Yeah.
639. **I: You were stuck up?**
640. **F1:** No! I would have been.
641. **I: You would have been stuck up? So, it’s humbled you in that sense.**
642. **F1:** Yeah. Yeah.
643. **I: Okay.**
644. **F1:** Yeah, yeah. It reminds me of it, to be.
645. **I: It reminds you to be humble.**
646. **F1:** To be human.
647. **I: To be human.**
648. **F1:** Yeah, yeah.
649. **I: Okay, okay. What…why does it do that do you think? Like, why does it humble you or make you feel human?**
650. **F1:** Because, because as I was saying-
651. **I: Mhm.**
652. **F1:** I am pretty, and I got yeah. So, when, when I was working at Pizza Hut,
653. **I: Mhm.**
654. **F1**: People would come in and say, “I do not want the pizza from you.”
655. **I: Okay.**
656. **F1**: And I remember that because of the way that I sound, they would hear me talk and they would say “I don’t want it.”
657. **I: “I don’t want to be served by you because of how you talk.”**
658. **F1:** No, they didn’t say that, they said the r-word.
659. **I: Oh, okay, they used the r-word.**
660. **F1:** No, they didn’t say it, but that’s what they mean.
661. **I: That’s what they were implying?**
662. **F1:** Yeah, yeah.
663. **I: Okay.**
664. **F1:** Yeah.
665. **I: Okay, so they were making that assumption again?**
666. **F1:** Yeah, yeah.
667. **I: Okay. By using that word.**
668. **F1:** Yeah, yeah.
669. **I: Okay. So, that is something that was humbling?**
670. **F1:** Yeah, yeah.
671. **I: Gotcha, okay.**
672. **F1:** It reminds me not to get too big-headed.
673. **I: Don’t get big headed because there, there are those experiences that kind of humbled you.**
674. **F1:** Yeah, yeah.
675. **I: So, it’s more so societal beliefs about disabilities?**
676. **F1:** Yeah, yeah.
677. **I: Those assumptions, right?**
678. **F1:** Yeah.
679. **I: Okay…**
680. **F1:** But it humbles me a lot, I, I, I appreciate having this disability.
681. **I: You have that respect; you appreciate having a disability.**
682. **F1:** Yeah, yeah. Because I see, I see, my cousins and other people, they, I don’t think they recognize other people that aren’t like them. They treat people with, from, other conditions like they are not human, that’s not the answer. I see people, I never see a color; I see people first.
683. **I: People first.**
684. **F1:** Yeah.
685. **I: And you think that they because they don’t have the disabilities or maybe something else that affords them the same opportunities that you’ve had that they don’t have that same ability?**
686. **F1:** Experiences right.
687. **I: Experiences, right. They haven’t been humbled in any other way.**
688. **F1:** Right.
689. **I: Okay, okay, um. So, I wanna, I mean if you have any good or bad experiences let me know, but I wanna kind of move to a different topic area, so, um, with the work we’re doing, we, we know, you know talk about, you know, can we in the healthcare system document disability status so that we can then use it to, to kind of, to use it in evaluation to see are we giving good care to patients with disabilities relative to their peers without disabilities. That’s kind of what we are trying to do.**
690. **F1:** Right.
691. **I: So, the question then is, you know, first do you feel comfortable sharing that information about yourself?**
692. **F1:** *Yeah*.
693. **I: Okay, okay. Then I guess the second question would be if you feel comfortable, you know, what is the, what should you be asked, or what should a healthcare provider ask you , what do you think that they need to know to give you better care?**
694. **F1:** Um, what, what would they ask another person?
695. **I: Yeah, okay. What would it look like?**
696. **F1:** In the beginning, ask the same questions but using different words.
697. **I: So, ask, ask about disability, but in different words.**
698. **F1:** No.
699. **I: Okay.**
700. **F1:** It doesn’t matter–you must learn what the disability is.
701. **I: Learn the actual type of disability?**
702. **F1:** And then, I thought, I thought that would lead to asking the question.
703. **I: Mhm, ask the question.**
704. **F1**: In a different way. The same question that you would asked that the healthcare provider would ask you.
705. **I: Mhm.**
706. **F1:** But, ask in a different way.
707. **I: How do you mean when you say ask in a different way?**
708. **F1:** I mean that, that someone with an intellectual disability.
709. **I: Mhm.**
710. **F1:** You, you understand that they do not communicate, they do not communicate in the same, in the same manner that somebody with Cerebral Palsy would do since we do not have an intellectual disability.
711. **I: So, it has to be, so the question has to be, the question has to be, um, tailored to the level that the individual can comprehend depending on the type of disability?**
712. **F1:** Right.
713. **I: Okay. So, if they have an intellectual, an intellectual, disability, maybe we have to make sure -**
714. **F1:** To break it down.
715. **I: we break it down so that they can understand.**
716. **F1:** Right.
717. **I: Well, let me just show you as an example, this is not necessarily, you know, the, the best way. I’m just giving you a starting point to think about.**
718. **F1:** Right**.**
719. **I: So, these six questions here,**
720. **F1:** I can read them.
721. **I: Are, yeah, I can read them to you as well, either way. These are used, um, for the U.S. census to determine disability status, so they’re not really intended for the healthcare setting, but it’s just kind of, um, to open up conversations. So, in this sense, you know, the six questions are, you know: Are you deaf or have you had serious difficulty hearing? Are you blind or do you have serious difficulty seeing even when wearing glasses? Um, because of a physical, mental or emotional condition, do you have serious difficulty concentrating, remembering or making decisions? Um, do you have serious difficulty walking or climbing stairs? Um, do you have serious difficulty dressing or bathing? Um, because of a physical, mental or emotional condition, do you have difficulty doing errands alone, um, such as visiting a doctor’s office or shopping? So, would you answer yes to any of those?**
722. **F1:** Um, yes, to the stairs.
723. **I: Which one? Number 4?**
724. **F1:** Yes, about coming down, not going up.
725. **I: Okay, so just going down the stairs, not going up. Is that, is that associated with your Cerebral Palsy?**
726. **F1:** Yeah.
727. **I: Okay, so just that? Number 4.**
728. **F1:** Um, yes. But either, either way, I am different.
729. **I: Okay.**
730. **F1:** I my, my cousin.
731. **I: Mhm.**
732. **F1:** She talks when, when STS arrives. She, she, all she does, she explains that I have this condition and I do all the things, the majority of these.
733. **I: You’re doing the majority of these things, right?**
734. **F1:**  I constantly am doing it by myself.
735. **I: Constantly doing it by yourself?**
736. **F1:** Yeah. I compensate.
737. **I: You compensate? Mhm.**
738. **F1:** Because, because, I had to learn a long time ago.
739. **I: Mhm.**
740. **F1:** That I am not gonna be–I am not gonna be–I might have a disability, but I am not disabled.
741. **I: Right. So, it doesn’t speak to your abilities or your capabilities.**
742. **F1:** Right.
743. **I: Mhm.**
744. **F1:** Right. But I try to, I try to do it in a different way.
745. **I: Mhm.**
746. **F1:** I do. When I was learning to drive.
747. **I: Mhm.**
748. **F1:** I just don’t want to do it one, two, three. But I wasn’t able to do that because my limitations, my disability; the driver who taught me. (inaudible 52:13).
749. **I: Mhm.**
750. **F1:** He’s the one, two, three, four, go, yeah, I try to respect what I have just because he’s doing it all day (unclear 52:33).
751. **I: Right.**
752. **F1:** Except for the first one, no the second one.
753. **I: What about the second one?**
754. **F1**: That is deaf. I do not have no limitation like this.
755. **I: Mhm.**
756. **F1:** But I have the understanding to (inaudible 52:43)
757. **I: Number four?**
758. F1: The most important one is that one.
759. **I: Okay, so question. I mean, is there any other things, I mean, with regards to the assumptions and the negative experiences that you’ve had, are there any other questions or ways that they can ask that would give them information that would help them address these problems?**
760. **F1:** Yeah, there are certain things, that you, that, um, read the first one again.
761. **I: Read the first one? Uh, are you deaf or do you have serious difficulty hearing?**
762. **F1:** Don’t ask that.
763. **I: Don’t ask that? Wrong wording?**
764. **F1:** Yeah**.** Wrong wording.
765. **I: Okay, so give me a better wording if you would.**
766. **F1:** Um, learn sign language.
767. **I: So, learn sign language.**
768. **F1:** Yeah.
769. **I: Oh okay, so you need. If you’re communicating with someone that’s not going to work, so it has to be sign language you’re saying?**
770. **F1:** Yeah.
771. **I: Okay.**
772. **F1:** Right, learn to sign, to sign with deaf.
773. **I: Right, so use of sign language would be more appropriate for that one. Right?**
774. **F1:** Right. Number 2?
775. **I: Okay. Are there any other things you can think of?**
776. **F1:** Read number 2.
777. **I: Okay read number 2?**
778. **F1:** We can go through.
779. **I: Yeah, this is exactly what I want. Okay, so number 2. Are you blind or do you have serious difficulty seeing even when wearing glasses?**
780. **F1:** Are you blind? Um, oh, are you blind? No. I would, I would ask the second part first.
781. **I: You would ask the second part but not the first part? Okay. Second part only.**
782. **F1:** No, not only. I would ask, ask the second part first.
783. **I: Ask the second part first?**
784. **F1:** Because, because that is, if they, if they are blind, they will say no, I am legally blind. They, they would tell you.
785. **I: Right, so this would kind of give partial then full? Gotcha.**
786. **F1:** Right, right.
787. **I: Okay. And of course, we would probably need braille or something like that. Right?**
788. **F1:** Right! That one you, that one you do not need braille yet, you need braille not when they are blind, they hear very well.
789. **I: Um, 3^rd^ question then. Um, because of a physical, mental or emotional condition, do you have serious difficulty concentrating, remembering or making decisions?**
790. **F1:** Last. Do you, do you have difficulty?
791. **I: Okay, so the second part really. You wouldn’t ask the first part.**
792. **F1:** Yeah.
793. **I: Okay. Remove beginning,**
794. **F1:** Yeah.
795. **I: Okay. Um, 4, do you have serious difficulty walking or climbing stairs?**
796. **F1:** This is broad. I have coming down.
797. **I: So, you would want to give more specifics about up or down stairs?**
798. **F1:** Yeah.
799. **I: Okay.**
800. **F1:** Or what amount of steps you can go. One flight, two flight, that would give you more specifics.
801. **I: So, number of steps or flights that you are able to do, okay. Anything else with that one? Or do you like that?**
802. **F1:** Do you ask them about limitations, what are your physical limitations?
803. **I: Okay, so what are your physical limitations?**
804. **F1:** Or, what *can* you do? I can do, do the same thing that I tell you not to do.
805. **I: Mhm. Right.**
806. **F1:** (Overlapping chatter, hard to decipher (56:20)) Right, right, what can you do. You can’t do anything physically for the intellectually challenged. What are your physical limitations that sounds wrong.
807. **I: So, that sounds negative you’re saying?**
808. **F1:** No, not negative.
809. **I: Mhm.**
810. **F1:** The person with intellectual disability may not understand about what that means.
811. **I: So, it’s not clear what physical limitations might be in?**
812. **F1**: Right.
813. **I: Okay.**
814. **F1:** Right. Can you jump? Can you stand? Can you move? It’s good for the person with intellectual disability.
815. **I: So, instead of saying what can you do or what can’t you do, giving you specific like can you do this? Can you do that?**
816. **F1:** Right, right. You would ask them to do that with Cerebral Palsy.
817. **I: Mhm.**
818. **F1:** What, and you, you’d have a survey for them.
819. **I: Mhm.**
820. **F1:** I would take a survey of, of what education or background I have because that makes a difference in how you communicate with me.
821. **I: Mhm, okay. Okay, so, question number five then, do you have difficulty dressing or bathing? How do you like that question?**
822. **F1:** Button. Do, can you button? Can you?
823. **I: So, like specific things like buttons, mhm. Anything else you can think of that would make this.**
824. **F1:** Any, coming out the shower, going in.
825. **I: Mhm. Okay. Coming out the shower. And then, the last one, so it has the same beginning as the other ones so I’m thinking you want to get rid of the because of physical, emotional or mental conditions. So, the question would be, do you have difficulty doing errands alone, such as just visiting a doctor’s office or shopping?**
826. **F1:** Right.
827. **I: Is that one okay, or would you change it or add to it in any way?**
828. **F1:** Well, I would customize it too.
829. **I: Right, so you think it, it all should be customized?**
830. **F1:** Yeah.
831. **I: And, when you say customized, how do you mean?**
832. **F1:** Like, so, um, so if you’re talking to someone with Down Syndrome, and you see that they, they’re not, they’re not all, they do not understand the idea of what you’re saying at first.
833. **I: Right, Down Syndrome might not understand what is being said.**
834. **F1:** Right. Certain aspects you may need to reassess at the next visit and explain what you meant. Ask, ask the kind of question that is.
835. **I: Mhm.**
836. **F1:** Do not talk to the people or the caregiver. That is a no-no.
837. **I: So, don’t. Something that says don’t talk to the caregiver kind of thing.**
838. **F1:** Yeah, yeah. Or limit communication.
839. **I: Or limit caregiver communication.**
840. **F1:** Yeah. Who you are going with and they should be, they should be, um, in the conversation because some people they should be a guidelines for the parent to, and they know how many times they are going to talk for you when you can talk for yourself.
841. **I: So, there should be some guidelines about parents intervening when, when you’re an adult and you can take care for yourself.**
842. **F1:** Right, Right! Not, not that there is anything that they can answer all of the questions effectively, it is individual. But you should give them the opportunity to do it. Because maybe, the person does not want to say it in front, in front of the parent.
843. **I: Mhm.**
844. **F1:** You know?
845. **I: Right.**
846. **F1:** They have me sit at school and they forget, they’re not going answer that, and certain people, certain people, they do not know what it is!
847. **I: Mhm, they don’t know, mhm.**
848. **F1:** So, the parent in the room answers those are the types of questions, type of indirect question that are private.
849. **I: Right, are you going to want to answer all those questions when your caregiver is right there, your parent is right there, no privacy.**
850. F1: Right!
851. **I: Okay. So, so kind of falling in line with this idea that you’re talking about, so, if, for maybe your personal benefit would it be helpful to include a question in here about whether or not you need a caregiver–**
852. **F1**: (Laughs)
853. **I: –and that might help to, do you think that would be helpful?**
854. **F1:** No, not for me.
855. **I: Not for you. Okay, so how, can you think of any question that could be asked, um, that would try to get at avoiding that assumption being made about whether or not you can comprehend?**
856. **F1:** The only thing that I would want to let them know is do not make assumptions because I, not every, not everyone who has, who has these, um, these speech impairments, it could’ve happened via stroke. No, do not assume that.
857. **I: Right, it could be a different condition entirely. Or, it doesn’t speak to anything other than what they are hearing and not comprehending.**
858. **F1:** Right.
859. **I: Okay. So, so can you think of, if that’s the takeaway “do not make assumptions.” Is there any way that can be captured in like collecting information like this or, or no?**
860. **F1:** I think, um, there’s a way, um, I do, um, I think you can ask the question. I think it’s good for them to ask the question.
861. **I: Mhm.**
862. **F1:** What is it that you have? Why do you sound like this?
863. **I: Mhm.**
864. **F1**: Look at the person and do not avoid the question.
865. **I: So, ask the question?**
866. **F1:** Yeah. Direct.
867. **I: So, ask what type of disability you have?**
868. **F1:** Yes.
869. **I: Or in this case, why do you speak like that?**
870. **F1:** Right.
871. **I: Because that way you aren’t making assumptions about disability, or stroke, or whatever it is, okay.**
872. **F1**: I think that is the best way.
873. **I: Mhm, okay. Um, so there is, so, so, so that’s the best way to go by.**
874. **F1:** Yeah, for me.
875. **I: So, ask specifically. It’s more in the process of having a conversation maybe the first time you interact with the new patient. Asking collective information and kind of documenting it in the medical record. So, you would prefer that happening in a dialogue versus like, um, like filling out a form?**
876. **F1:** I like it.
877. **I: You feel like an in person conversation.**
878. **F1:** Because that will tell me a lot.
879. **I: You’ll get a sense of whether it’s, it’s -**
880. **F1:** Genuine.
881. **I: Genuine? Okay, versus the form you don’t know because you haven’t interacted with anyone yet.**
882. **F1:** Right.
883. **I: Okay, um, would that be the only way to do it? Is there anything in a form that you think?**
884. **F1:** I think maybe for some, in, in, in a form. But, for *me*.
885. **I: In the form, for you in person?**
886. **F1:** Because I read very slow and write.
887. **I: Because you read and write slow? Okay.**
888. **F1:** Right. Exactly.
889. **I: So, it might be faster this way, and the genuineness you can pick up on.**
890. **F1:** Yeah, right.
891. **I: Okay, okay. And how often should that conversation, should that conversation be had?**
892. **F1:** Whenever.
893. **I: Only once is good enough?**
894. **F1:** No.
895. **I: Oh, okay.**
896. **F1**: Whenever you feel comfortable doing it.
897. **I: Mhm.**
898. **F1**: I don’t mind.
899. **I: Mhm.**
900. **F1:** I don’t mind.
901. **I: Okay, and is there, I guess, is there anything that, um, they should know in terms of what to do or what not to when having that conversation?**
902. **F1:** What not to do is looking down.
903. **I: So, the, beyond, the do not make assumptions.**
904. **F1:** Yeah, do not look down.
905. **I: Do not look down**
906. **F1:** Look at the individual.
907. **I: Make eye contact.**
908. **F1:** Yeah.
909. **I: Okay, so those are the things.**
910. **F1:** Do not look away because you may miss something.
911. **I: Okay. So, don’t look away, so you don’t miss something. Anything about words not to use or certain tone or anything like that that you would add?**
912. **F1:** I would hope that you would, you would respect and use professional language,
913. **I: Okay, but not, but not like medical jargon that’s confusing.**
914. **F1**: Right.
915. **I: Just professional, the dignity, respect aspect.**
916. **F1:** Right, okay.
917. **I: Okay, okay sounds good. Alright. Um, let’s see here. I think that’s the, the gist of all my questions. Um, is there anything else that I missed that you can think of that you, you thought good or bad experiences that you want to add?**
918. **F1:** Um, as long as I get what I need.
919. **I: As long as you get what you need?**
920. **F1:** It doesn’t matter.
921. **I: Okay, do you think that’s true for other people as well, or is that unique for you?**
922. **F1:** No. That’s unique for me.
923. **I: Okay. Cause you’re like forget you, as long as I get what I want, I’m good.**
924. **F1:** Right, as long as you do what I needed you to do.
925. **I: With dignity, respect -**
926. **F1:** Right, right. That’s right. I go there for a bandage with my foot, and if I walk away without that bandage on my foot, I’m not going back.
927. **I: Right, so that’s got to be quality care, if you need the bandage on your foot, if that’s what you get, you’re good, if that’s what you don’t get, problem.**
928. **F1:** Right, right. I need the bandage on my foot. If you have attitude doing it, that’s your problem.
929. **I: So, you can do it with attitude, so long as you do it right.**
930. **F1:** Right, do not, do not intentionally make me.
931. **I: As long as you’re not causing any harm or like missed opportunity.**
932. **F1:** I just think, I just think, they, they want that dignity and respect. My takeaway, my last comment is that you treat individuals as you want to be treated.
933. **I: Yeah! The golden rule, right: do unto others as they will do unto you.**
934. **F1:** Right, you treat them, even when they have limited cognitive ability, you *still* treat them with respect.
935. **I: Right, right.**
936. **F1:** You know, because, because you wouldn’t let nobody treat *you* or if you have a child that way
937. **I: Right, you wouldn’t want you or your child to be treated that way, so you would expect.**
938. **F1:** Right, that is my take-away. If you have a child with limited abilities, whatever it is, physical, cognitive, intellectually, you know, you find a way to, to treat that child or that individual.
939. **I: So, you, you modify or adjust so that you still provide that quality care, okay.**
940. **F1:** Right.
941. **I: Okay, awesome, thank you. I’m going to stop this.**
