# Supplementary material for: Disability disclosure in healthcare settings for individuals with developmental disabilities: A qualitative study of patient and caregiver perspectives
Source: PLoS One. 2025 Aug 7;20(8):e0329328. doi: 10.1371/journal.pone.0329328 (PMC12331114; doi:10.1371/journal.pone.0329328)
Supplement: S1 File — (ZIP) [file pone.0329328.s001.zip › Transcripts/2019.09.10 Interview 11 Transcript.docx]

**Interviewer: Just uh, just for the record, just so the audio recording is on. You've... We've gone through the informed consent and you've agreed to participate to be recorded?**

Person: Yes I do.

**Interviewer: All right. Awesome. So, um, yeah. Let's jump in. So, um, when you think about your son's healthcare experiences, would you say you've got good ones? Bad ones? Both?**

Person: Both.

**Interviewer: Both, okay. So, let's- let's delve into those a little bit. So, I guess let's start with the- the bad. But tell me any specific experiences. What- what made those experiences bad?**
Person: When he get... Got older.

**Interviewer: Okay.**
Person: As... Once he turned the age 18...

**Interviewer: Mm-hmm (affirmative)-**

Person: Well, between 18 and 22 the benefits and the doctors were not available for his disability. Apparently I can... Cannot find... He has Autism.

**Interviewer: Mm-hmm (affirmative)-**
Person: And we cannot find specific neural psychologists- psychologists for Autism after the age 22. I'm having a difficult time.

**Interviewer: Mm-hmm (affirmative)-**
Person: So, I'm just taking the basic psychiatrist, the basic... I haven't even been able to go back to a Neurologist that can just kind of deal with him. The last time we've seen a neurologist was, I believe, at 22 when he graduate Coral Reef Senior High.

**Interviewer: Mm-hmm (affirmative)-**
Person: So, I'm at that point now. Other than that, I have been having positive uh, reactions with the doctors that are working with him.

**Interviewer: Mm-hmm (affirmative)-**

Person: He's with uh, primary Doctor Minkes, so he's not a specialist as that, but he took him over from the pediatrician.

**Interviewer: Mm-hmm (affirmative)-**
Person: Which was um, what's his name? Doctor Geraldi, who specialized in all types of uh, disability.

**Interviewer: Mm-hmm (affirmative)-**
Person: 'Cause he does have... He adopted a lot of dis... Difi... Disabilities in his home, so he really helped me and geared me...

**Interviewer: So, personal experience?**
Person: Personal experience.

**Interviewer: Mm-hmm (affirmative)-**

Person: And he told me, when we diagnosed him at three years old.

**Interviewer: Mm-hmm (affirmative)-**

Person: He said, if he's not Autistic uh, by five um, they will always, you know, redirect him according to what his disability is. But, apparently he was. And from then on, he's... I've had awesome and positive uh, experiences with the doctors, neurologists, Miami Children, even with dental. We started off with uh, Medicaid, then the HMO kicked in with MediPass and then at that time, that's when it kind of got tricky. It was an HMO...

**Interviewer: Mm-hmm (affirmative)-**

Person: A lot of the doctors and the specialists would not accept him because of the HMO. But, I work with Medicaid and said because of his specfictic... Specific diagnosis...

**Interviewer: Mm-hmm (affirmative)-**

Person: Um, a lot of those HMO are not available to him. And some of them are so far away in Broward, it's difficult to just, you know, go back and forth. So, Medicaid allowed me to stay on Medicaid til this day. So, he's open up to all the specialists and all the providers that I needed. The medication, the transportation, dental, hospital.

**Interviewer: Mm-hmm (affirmative)-**

Person: Straight Medicaid til this day, so that's great.

**Interviewer: Okay.**

Person: But the bad part is that he's 32 years old and specific specialists I cannot find for his age.

**Interviewer: Mm-hmm (affirmative)-**

Person: That's where I find my [inaudible 00:03:04].

**Interviewer: So, when you- when you find a need for him, I mean, are you just going without or are you finding someone that is just not what you're... What your ideal is?**
Person: That's what... That's what I am now.

**Interviewer: Okay.**

Person: I found a psychologist... 'Cause my specialist said... My primary told me, "I cannot keep treating him on this particular medication. I was not the initial one that prescribed him. I'm very concerned with the medication that he's on, 'cause he's been on it, I would say uh, let's say seventh grade?

**Interviewer: Mm-hmm (affirmative)-**
Person: And he noticed that he decide... He was developing breasts... It was called gyneco something.

**Interviewer: Mm-hmm (affirmative)- mm-hmm (affirmative)-**

Person: And his breasts... And that's a side effect of Risperdal. Luckily, he was checking his uh, prolactin, 'cause that was what he was concerned of, and apparently the last couple of years, it elevated.

**Interviewer: Mm-hmm (affirmative)-**

Person: So he was, "You need to get him off this medication." So, now I finally found somebody... Psychiatrist, which is not the best, but it's what I could find.

**Interviewer: Mm-hmm (affirmative)-**
Person: He recommended CHI, so we're with Doctor Kevin Ameri. Not very personal, but just, you know, kind of throwing him a different medication. So far this medication is okay.

**Interviewer: Mm-hmm (affirmative)-**
Person: We had him um, checked. You know, there's no um... They were afraid for breast cysts and breast what have you.

**Interviewer: Mm-hmm (affirmative)-**

Person: Luckily, they did a mammogram. Everything is fine. Which that was... That was very dif... Very difficult. Very, very difficult. But, he got through that. So now we're just checking his prolactin level to see if that medication is working for him. It was kinda tough because it just... He just... Whew, it was tough.

**Interviewer: Mm-hmm (affirmative)-**

Person: 'Cause it got him very hyper, very assertive, very aggressive. The program saying he's very aggressive. They changed the dosage, but he can't sleep at night. I finally got him to the point that he can sleep with the melatonin in the evening, and five milligrams of ARI. Can't pronounce the medication, but it's much better. So now, um, the only side effect that I see is that he lost so much weight. A lot of weight. So now we're going back to the doctor...

**Interviewer: Mm-hmm (affirmative)-**
Person: To see if there's any concern, which the primary was happy with because he was pre diabetic. But, since he lost the weight, sugar went down. He's on a specific diet, so that's great.

**Interviewer: Mm-hmm (affirmative)-**
Person: So the- the doctor so far are okay. But, my main concern is the psychiatrist...

**Interviewer: Mm-hmm (affirmative)-**
Person: To really get him into somebody that deals with Autism.

**Interviewer: Right.**
Person: This is just a- a...

**Interviewer: So you mentioned that he's personal. Like what- what is that interaction with your son? I mean, like, how does that go? And is it, you know... Is it...**
Person: It's not really that he tries to get anything out of my son...

**Interviewer: Mm-hmm (affirmative)-**
Person: This is just like, I'm talking for him. Like, he doesn't try to engage...

**Interviewer: Mm-hmm (affirmative)-**
Person: With my son.

**Interviewer: And you think he should be doing that?**
Person: I think so.

**Interviewer: Okay.**

Person: Because I want... When he was younger, the doctor's... The psychiatrist that he used to have sometime would take me out. Say, let me, you know, work with him and...

**Interviewer: One on one?**
Person: One on one.

**Interviewer: Mm-hmm (affirmative)-**
Person: And they would bring me back in.

**Interviewer: Mm-hmm (affirmative)-**

Person: And then um... But this doctor just strictly talks to me. And I try... I try to get my son to talk to him, but you know, I guess he doesn't feel the connection. And my son... Which is a bad thing. Every time somebody asks him a question, he looks at me because I'm so used to answering for him.

**Interviewer: Mm-hmm (affirmative)-**
Person: But, that's what I would like the psychiatrist... Psychiatrist to talk to him and try to get some interaction with him. It's just like, "Okay, we're gonna try this medication. Let's see how it's doing and we're happy? Okay. Come back a few months." And that's it.

**Interviewer: Mm-hmm (affirmative)-**
Person: So um, I wish I could find a doctor that specifies the... With Autism.

**Interviewer: Mm-hmm (affirmative)-**

Person: A lot of them doesn't take Medicaid. A lot of them don't take uh, adults. Always under the age 18, or under the age 22. And he needs that.

**Interviewer: Mm-hmm (affirmative)- mm-hmm (affirmative)-**
Person: Other than that, that's where I'm at right now.

**Interviewer: Right. And you mentioned... You said the mammogram was a challenge. Would you tell me more about like, what made it such a challenge? Or?**
Person: Because he didn't understand w-what was going on.

**Interviewer: Mm-hmm (affirmative)-**
Person: And I think it was a little bit more challenging for me to see 'cause I know what that is about and for a male to have that done, I had to explain it to him ahead of time and he- he's very, very private because he knows this is not normal 'cause he sees his dad and he sees his brother. So, it's not normal for it to be so large.

**Interviewer: Mm-hmm (affirmative)-**

Person: So, to have him explain... To have him take off his shirt in front of the nurse and have to just, you know...

**Interviewer: Mm-hmm (affirmative)-**

Person: He didn't get aggressive, he just didn't understand. He was embarrassed, he didn't want to do it so, it took us a little trial and error. Luckily the nurse was very patient with him. It was a little uncomfortable.

**Interviewer: Mm-hmm (affirmative)-**
Person: 'Cause you know, they have to squeeze down and...

**Interviewer: Mm-hmm (affirmative)-**
Person: But, we got through it and thank lor... Thank the lord nothing came about it. But now that he lost a lot of weight, that has gone down a little, but as woman also...

**Interviewer: Mm-hmm (affirmative)-**

Person: When we lose weight, everything sags. That's where he's at right now. (laughs) Yes.

**Interviewer: Um, so, you said t-the nurse was patient. Did t-the nurse or anyone else in during that process, do anything else to kind of, you know, make the- the experience more pleasant for him?**

Person: Well, because of the doctor's office that I go to, his primary...

**Interviewer: Mm-hmm (affirmative)-**
Person: He has all the, [inaudible 00:08:10] the x-rays and EKG's and mammogram and he's so good that even though he knows Medicaid doesn't pay that much for it, that he's been following Danny so much and he's so concerned about it, he didn't care so much of how much he was gonna get back.

**Interviewer: Mm-hmm (affirmative)-**
Person: He cared more that we need to make sure that Danny is, you know, nothing is happening to him. He took care of him from the pediatrician... I think maybe the age of 25? 23? About 23 he started taking him.

**Interviewer: Mm-hmm (affirmative)-**

Person: And he just followed the medical records of what doctor Geraldi gave him. 'Cause it... I was taking him to doctor Geraldi and here he is. My son is six two, six three. He couldn't fit (laughs) But I said, we're gonna walk in here with pride. I- I don't even care 'cause I feel very comfortable with doctor Geraldi, 'cause he knows him.

**Interviewer: Mm-hmm (affirmative)-**
Person: So, I finally went to Doctor Minkes and he's been treating him very well. He talks to him. He interacts with Daniel. You know, he makes me feel- feel comfortable.

**Interviewer: Mm-hmm (affirmative)-**

Person: So... The nurses fe... Make him feel comfortable. The- the receptionist, "Hi Danny, how you doing?" "Hi Rosie. How are you doing?" That's how he speaks.

**Interviewer: Mm-hmm (affirmative)-**

Person: "I'm doing fine." "Well, you look great! You lost weight!" "Yes. Yes I did." He goes, "Thanks Rosie." (laughs) That's how he speaks. He's very... Very sweet, so with them, awesome. They don't care as much as a give take back of what they're gonna get, they just care more about the care of Danny.

**Interviewer: Mm-hmm (affirmative)-**
Person: So that's the blessing.

**Interviewer: Absolutely.**
Person: His specialist is where I'm having a concern.

**Interviewer: Mm-hmm (affirmative)- So yeah, tell me more about, you know, whether it's- it's pulling from experiences you've had with your... The primary. Or, you know, what you envision when you talk about finding a psychiatrist that focuses on patients with Autism. Like, what- what would make that experience better? What are they doing, other than you know, you just kinda were talking about like, the rapport. The- the back and forth?**
Person: As far as the doctors?

**Interviewer: Mm-hmm (affirmative)-**

Person: Well, like I said, I don't find them engaging. With that particular doctor...

**Interviewer: Mm-hmm (affirmative)-**

Person: I don't find him engaging, but then again, I know that they not... They don't specify...

**Interviewer: Mm-hmm (affirmative)-**
Person: With that disability.

**Interviewer: Right.**

Person: You know, a lot of the psychiatrists that I did go to before, they specialized with that. Therefore, they know how to interact.

**Interviewer: Mm-hmm (affirmative)-**
Person: They know how to get them to respond. They understand.

**Interviewer: Mm-hmm (affirmative)-**
Person: This doctor doesn't have that experience, but that's the only one I had to change... My main concern was to change the medication. And he has.

**Interviewer: Right.**

Person: And so, I have to give that to him for that.

**Interviewer: Mm-hmm (affirmative)-**
Person: But, I think that he needs more. Like, a- a therapy session on an on-going base. Like, maybe once a month or twice a month to help him to communicate on his own.

**Interviewer: Mm-hmm (affirmative)-**
Person: So I can disengage with him.

**Interviewer: Mm-hmm (affirmative)-**
Person: See, that's the ne... Another situation I'm trying to look to a group therapy type situation or one-on-one situation once a week to talk to him.

**Interviewer: Mm-hmm (affirmative)-**

Person: Just like when anybody goes to a psychiatrist. Help him to deal with his differences, 'cause he knows he's different. But, he... He's comfortable in his own skin.

**Interviewer: Mm-hmm (affirmative)-**
Person: And he's... He's only usually just with us, right now.

**Interviewer: Mm-hmm (affirmative)-**

Person: I have family members, but everybody's moved and what have you. Now, it's really just my son and my husband and myself. And my mom, but now she moved to West Palm Beach, so it's...

**Interviewer: Mm-hmm (affirmative)-**
Person: He doesn't really get much of a outdoor, outside engagement except for that ADE, which is with other disabilities. So, it's, you know, I just need like a respite type care. Something that will help him...

**Interviewer: Mm-hmm (affirmative)-**
Person: To be interpersonal with somebody else other than his immediate family.

**Interviewer: Right. Right.**
Person: And I know that before, when he was younger, they had so many things. Now, we're limited.

**Interviewer: Mm-hmm (affirmative)-**

Person: Very limited.

**Interviewer: Mm-hmm (affirmative)- Okay. So, any other things you can think of that would stand out um, that make it a- a quality experience? We talked about the personal interactions and taking the time, being patient. Any other things that uh, you feel they do specifically for Danny because they know that this is the best way to- to approach him, interact with him...**
Person: As far as who? The... Any of the?

**Interviewer: Any of the healthcare providers, yeah.**
Person: Well, like I said, I only have that one, Doctor Minkes. Like, they- they greet him as he walks in. He knows exactly where... What to do. They tell him to urinate in a cup, they said, "Can he do this?" He goes, "Yes I can. Yes I do." And I'm like, you can? Okay, go ahead.

**Interviewer: Mm-hmm (affirmative)-**
Person: So, they really treat him like he doesn't have any difference. That's- that's just so important to me. They treat him as anybody else.

**Interviewer: Mm-hmm (affirmative)-**

Person: They laugh with him and they joke with him and sometimes when they joke, he goes, "You think that's funny?" They go, "Yo, Danny, that's not funny?" (laughs) But, so they treat him like a person.

**Interviewer: Right.**
Person: Not as if he has a difference and that's what I- I want in life for him to... People to treat him as normal as possible.

**Interviewer: Mm-hmm (affirmative)-**

Person: But I understand that people may not understand his difference, but this particular doctor Jules Minkes and his office staff are tremendous. But then again, they also they have a lot of clientele that have different disabilities that are adults. So they know how to engage and how to talk and how to make them feel comfortable.

**Interviewer: Mm-hmm (affirmative)- So you think that- that experience [inaudible 00:13:17] informs them or did they... Did they ever do anything... When you... When you first came in for the first visit, did they ask you certain questions. Did they say, give me information so I know how to better care for Danny?**
Person: Oh yes.

**Interviewer: So, tell me about that.**

Person: A little bit... A little bit of that, his back history is what I've told you and I've told them as far as the um, Doctor Geraldi, which is awesome and Miami Children's Hospital, which engaged him. Did... Did every kind of test possible. Followed up with him without- without a beat. From the time he was diagnosed at three, all the way til 22...

**Interviewer: Mm-hmm (affirmative)-**
Person: When they said I can't take... Miami Children's said I cannot take him any longer. His doctor was Trevor Resnick. He started doing experimental... Not experimental but drugs that the... That would help him with his stimulant, 'cause he had a lot of stimulants and they said that's because of his uh, puberty. And so, they said, okay we need to do something with this. He was getting aggressive and the schools were having a hard time kinda like, you know, containing... Not con... Well, I can say containing him because he was so much bigger than the other children. So when he would get aggressive, you know, his body... He don't want to hurt the other children also.

**Interviewer: Mm-hmm (affirmative)-**

Person: I was a little bit reluctant to putting him on medication so early, but then I don't want him to endanger other children. So, Doctor Resnick really worked with me and we tried this medication that didn't work. That medication didn't work. Then Risperdal. It wasn't 100% approved, but let's try this. A low dosage or miracles. Finally, it was and it went to generic, so it's great. Unfortunately, it was the side affect. So, we're working on that. Um, uh, all the doctors that I've gone, I'm gonna have to say, positive experience. It's just now where I'm having like a little difficulty, but I have very good... If it wasn't for them...

**Interviewer: Mm-hmm (affirmative)-**
Person: Also, not only for him, for myself as a caregiver and Autism at that time was not so profound as now.

**Interviewer: Mm-hmm (affirmative)-**
Person: They, you know, they were very, you know, that's what I say... Had a lot of interest of trying to help. Trying to figure out well, how we're gonna work this out. How can we um, improve Danny's quality of life? And informing me, giving me guidance, places to go for help.

**Interviewer: Mm-hmm (affirmative)-**
Person: I went to uh, the off... They recommended a place at, when he was younger, called Child Find...

**Interviewer: Mm-hmm (affirmative)-**
Person: Which they did this battery of testing, offer schools, and he has excelled. And instead of going to Southaide... I mean, South Ridge, which didn't have an Autistic program, they sent him to Coral Reef and they just opened a brand new wing and he excelled there.

**Interviewer: Mm-hmm (affirmative)-**

Person: And Doctor Geraldi followed through, they would have to do an IEP...

**Interviewer: Mm-hmm (affirmative)-**

Person: And follow up with my doctor and the school. So, all that color... Collaboration and uh, help with the doctors and the school, he... He's doing very well.

**Interviewer: Mm-hmm (affirmative)-**
Person: Very, very well. And even um... He had episodes of bronchitis...

**Interviewer: Mm-hmm (affirmative)-**
Person: And I always would take him to Baptist. That's an awesome experience. It even got to the point that when we got there, they pull up the chart, they knew who he was, no wait time...
**Interviewer: Mm-hmm (affirmative)-**
Person: Brought him right in. That was great. They also recommended uh... Medicaid paid for his dental care...

**Interviewer: Mm-hmm (affirmative)-**
Person: When he was younger and I said how are we gonna do this?

**Interviewer: Mm-hmm (affirmative)-**
Person: I just... I was like, so stressed about that. So, they said that he had quite a few...

**Interviewer: Mm-hmm (affirmative)-**

Person: And they said they're gonna go ahead and sedate him. I said, okay. Sedating him. That was a little uncomfortable. But they said if we sedate them, then we can do everything at one time. So, I said, okay. So how are we gonna do this...

**Interviewer: Mm-hmm (affirmative)-**
Person: So um, but I was thinking about the IV, how I'm gonna hold him down to put the IV to sedate him. But, they had the nurse... They knew exactly what to do. I sat by him. My mom came with me. The nurse relaxed him. He was able to put the- the injection. He went to sleep. I had sat down. They did all his work. Now he... Beautiful teeth. My own... My other kids want... They don't have as nice teeth as my older son because he was so well taken care of.

**Interviewer: Mm-hmm (affirmative)-**

Person: 'Cause the treatment that they did on his mouth was so extensive and so expensive that Medicaid did pay for it.

**Interviewer: Mm-hmm (affirmative)-**

Person: And it was inpatient. Even better, because I kept thinking going to the... It's already fearful for us as an adult...

**Interviewer: Mm-hmm (affirmative)-**
Person: And going there, I said, every time I have to go back with the injection and the drooling. I don't think he's gonna sit still for that. And at that time, he didn't have medication. So...

**Interviewer: Mm-hmm (affirmative)-**

Person: That was an awesome experience. So, Baptist Hospital and Miami Children Hospital took tremendous care of my son.

**Interviewer: Mm-hmm (affirmative)- And ho**w did the- the nurse... You said uh, she relaxed him. How did... How did she do that?

Person: By talking to him. Caressing him. Making... Trying to make him feel calm. Talking to him 'cause at the time, he didn't have very well... Good eye contact.

**Interviewer: Mm-hmm (affirmative**)-

Person: Talking to him, having eye contact, laughing with him, and making him joke... Tickle him. But the most I noticed are caressing him. I think that's more 'cause she was trying to find his vein?

**Interviewer: Mm-hmm (affirmative)-**
Person: So she was caressing him and, "Danny, you're so funny. You're so cute." He was laughing but he didn't talk very much. And so, it calmed him and then somehow or another, I guess... Now I'm thinking of it, that's how she found the vein and that's how she was able to insert it.

**Interviewer: Mm-hmm (affirmative)-**
Person: Cause I got... I guess her technique was to get him comfortable with her, for her to do the job before... By the time he, you know, he- he felt it, but he was already calm enough instead of having to struggle and see the needle and what have you.

**Interviewer: Mm-hmm (affirmative)-**

Person: She showed a good technique. That was...

**Interviewer: And did she ask any question... Or did anyone ask any questions beforehand about whether or not he was receptive to- to touch or anything like that?**
Person: Oh, definitely. Oh yes. You know, what his um, uh... He did... Is he aggressive? Is he... What... Is he very fearful? Is he talkative?

**Interviewer: Mm-hmm (affirmative)-**
Person: I said, well he's... I'm not... I don't... I'm not comfortable with this, but Doctor Geraldi said... Gave... 'Cause we... Let me go back. Um, when they recommended him to do the dental...

**Interviewer: Mm-hmm (affirmative)-**
Person: Doctor Geraldi gave... We have to get information. I had to sign a form for them to get information from Doctor Geraldi...

**Interviewer: Oh.**
Person: So they can get that backup. And then for me, she told... She asked me anything I should know about that I should be concerned about. Is he a biter? Is he aggressive? I said, "No, he's a very sweet, very calm, happy babe. But, I don't know how this is gonna happen. I don't know if he's gonna kick you. I don't know if he's gonna hit you." "But, is he aggressive?" She wants to know. Is he a biter. You know, is he a scratcher. Is he normally like this? I said, "No, he's passive aggressive. Very happy." She goes, "Okay." And then, at the same time, she was looking at him, you know, as she's asking me questions and then took it from there. At first, we did the preliminary and then we scheduled the date. I guess so she can see and interact with him. Kinda looked on his mouth. Open your mouth, see if he responded. Without me asking, he opened his mouth and she looked and then sometime he was looking at what did they put in his mouth. And he didn't put his hand on her or anything, but he was just concerned about what was going in his mouth.

**Interviewer: Mm-hmm (affirmative)-**
Person: They said, do we need to sed... Strap him? That's what they wanted to know. Should we... Do we have to strap him down.

**Interviewer: Mm-hmm (affirmative)-**
Person: And I was like, now they're asking me these questions and like, not... [inaudible 00:20:22] Wow. That was techniques that they used in order to help him with his disability at that age.

**Interviewer: Mm-hmm (affirmative)- So, it seems from- from their interactions that they have uh, maybe some type of training or knowledge of Autism...**
Person: Exactly.

**Interviewer: Rather than, this is what we would do for any patient.**

Person: Exactly. And they had uh, the spec... Uh, because it was at Miami Children, I didn't have to look for a dentist that dealt with children or young adults with uh...

**Interviewer: Mm-hmm (affirmative)-**

Person: Disability.

**Interviewer: Mm-hmm (affirmative)-**
Person: Took him to Miami Children, he took... And they took care of him all the way til 18, 22.

**Interviewer: Mm-hmm (affirmative)-**
Person: Now, that's another thing. Now that he's 32, I have to take him back to the dentist and they just sent me a form so now I'm wondering how that's gonna work out.

**Interviewer: Mm-hmm (affirmative)-**

Person: 'Cause I basically... It's gonna be at CHI. Or they asked me if I could choose from other doctors, so. That's another step I'm gonna have to deal with.

**Interviewer: See, and how do you... How do you find the next person to send him to now that he's kind of aged out?**
Person: Aged out. That's a good question. Well, I'm gonna um, have to call to see if they deal with adults with disability. But, he's pretty good now knowing about uh, shots...

**Interviewer: Mm-hmm (affirmative)-**
Person: Uh- uh, taking blood and IV's because he's not too long ago he was at Baptist 'cause he had a stomach virus or something and so they had to give him IV. He stayed still. He's looking, but he stayed still. They found the vein and they put it in to... Put it in to give him um, fluids. So, let's see. I don't know about the drilling though. So maybe they'll ask me the same questions and since that... I went through it before when he was younger, let me see how they're gonna react and how they're gonna deal with my son at that time. What questions they gonna ask.

**Interviewer: Right.**
Person: To have... Maybe they will give him laughing gas or something and put him out. Because now he's bigger and drilling is a different situation.

**Interviewer: Right.**
Person: I think that I'm gonna have to look at that. But, they just sent me a new information for dental, so I'm gonna go ahead and take him.

**Interviewer: Now, even for drawing blood and the shots now, I mean, do you feel like, you know, he's been through it and understand it as more okay? Or they still need to go through a similar calming...**
Person: No, not at all because it's either done um... He's had it done in the doctor's office. They don't even sit him in the lobby 'cause he- he could go through a lot.

**Interviewer: Mm-hmm (affirmative)-**
Person: But they just like to care of him there.

**Interviewer: Right.**

Person: Doctor Minkes. Um, 'cause now... Now a days, everybody has to go to a lab. The doctor's office don't want to do it. But, for Danny, they do it there.

**Interviewer: Mm-hmm (affirmative)-**

Person: When he goes to Baptist labs... Like I said, not too long ago, no problem. I think that he's so accustomed to it for so long, so many years that he have done it, and they know how... He knows when they tell him to pump his arm or whatever, they know. He knows exactly what to do, but he's watching. And he doesn't jump, he doesn't fidget. He does a good job and they're like, "Wow! Danny, you are really good!" They thinking they have to deal with something. They ask me, "Do I have to strap him?" I said, "No, he's gonna be fine. He knows how to do it." IV's and shots um, uh, uh, blood work, he's fine. He's very comfortable with that. Now, going to the dentist, let's see how that's gonna go.

**Interviewer: Right.**

Person: I don't think it's gonna be bad with this... Well, I don't know. That's a different situation. That is totally different.

**Interviewer: Truly.**

Person: To having to open his mouth and, you know, putting in his... 'Cause that's... Uh, 'cause I don't think he... You know, he didn't remember.

**Interviewer: Mm-hmm (affirmative)-**

Person: I haven't brought him really back since they did it at Miami Children. Being a bad thing.

**Interviewer: Mm-hmm (affirmative)-**
Person: I'm thinking about gosh, I have to do this and try to find a doctor and dentist to deal with Autism or disability. I say, well why can't Miami Children have an extended Miami Children for adults with children with dis... 'Cause they deal with so many different disabilities, you would think they would have something more for adults with... Adults with differences.

**Interviewer: Mm-hmm (affirmative)- Absolutely. Absolutely. Um, so- so, you know, one of the things that we're- we're asking about with [inaudible 00:24:17] is, you know, we know that based on the research that there are help disparities for individuals with disabilities compared to peers without disabilities.**

Person: Mm-hmm (affirmative)-

**Interviewer: Um, and so we're trying to learn how we can address this- this issue. Uh, and one of the uh, starting points is, you know, well, we can't... You know, we can't evaluate what we don't measure. I know when you fill out a patient form, not really anything that asks you about disability status. Have you e-ever encountered any- anyone in any health care setting that asked you specifically about Danny's Autism?**
Person: You know, they do have questions on the form...

**Interviewer: Mm-hmm (affirmative)-**

Person: But see, that's why I always get confused 'cause they... Even at... They'll ask me... It doesn't say disability...

**Interviewer: Mm-hmm (affirmative)-**

Person: It says... It has another word for something... But, I don't know how... Where to put that he has Autism.

**Interviewer: Mm-hmm (affirmative)-**
Person: So, I'm trying to think. I notice I always have that problem. Where do I put... 'Cause it doesn't say disability.

**Interviewer: Do you want to tell them so they know ahead of time? Is that why?**
Person: Yes.

**Interviewer: Okay. But they... But you don't know where...**

Person: Where to put it on the form.

**Interviewer: Okay. Okay**.

Person: Um...

**Interviewer: And I think that's a- a common observation we...**
Person: Yes.

**Interviewer: We're aware of. So, I guess the question is, you know, where... Where does it go and how should it be asked?**
Person: Exact... That's the question.

**Interviewer: Right. So...**
Person: It has different um... Has different questions where you think that it applies, but it doesn't apply. You know how when it says, do you have hypertension? Hyper this and then what? And then it says other? So, I don't know if that's the section? Should I put Autism? 'Cause that's not really a sickness.

**Interviewer: Not like a chronic condition**.

Person: Exactly. So I said, do I put it here or do I... So I don't put it anywhere until when I guess when they see the doctor and the doctor makes their notation.

**Interviewer: So that's the way it gets in. You have to communicate it to them.**
Person: Exactly.

**Interviewer: Okay. So- so I do, I mean, that's the question we're trying to answer. Um, we don't have the answer. We're hoping to learn from you.**
Person: Mm-hmm (affirmative)-

**Interviewer: Um, but we do have at least is a starting point.**
Person: Okay.

**Interviewer: So- so this right here is not intended for the healthcare setting. These six questions are um, used by the US Census to identify individuals that say that they have a disability of some sort.**Person: Okay.

**Interviewer: So, I just want to kind of read through them for you, just to get your- your thoughts on would any of these capture Danny? And is there something else missing that you would want them to know.**

Person: Okay.

**Interviewer: So, are you deaf or hard... Uh, have serious difficulty hearing? Uh, are you blind or do you have difficulty seeing even when wearing glasses? Um, because of either a physical, mental, emotional condition, do you have serious difficulty concentrating, remembering, or making decisions? Uh, do you have serious difficulty walking or climbing stairs? Do you have difficulty dressing or bathing? And because of these same conditions, do you have difficulty doing errands alone, such as visiting a doctor's office or shopping? So, just at first glance, what are you thoughts? Are- are those capturing anything or?**Person: Yeah, number three.

**Interviewer: Okay.**
Person: Because of physical, mental, or emotional conditions, do you have serious difficulty concentrating, remembering... This applies to him, but not everything applies to him.

**Interviewer: Right.**

Person: Because he has very good memory. Difficulty concentrating sometimes to stay on task.

**Interviewer: Mm-hmm (affirmative)-**

Person: Making decisions. Those are the two.

**Interviewer: Mm-hmm (affirmative)-**
Person: So, I guess that would apply to him.

**Interviewer: Okay.**
Person: So, it's not that all of it applies, but this one would apply to him. But, with remembering, he's great. Making decision, on his own...

**Interviewer: Mm-hmm (affirmative)-**

Person: He probably would need the extra boost. He knows right from wrong.

**Interviewer: Yeah**.

Person: But, for him to make a decision, he always likes to have...

**Interviewer: Confirmation from you kind of?**

Person: Yes. And that's- that's where I need that extra help.

**Interviewer: Mm-hmm (affirmative)-**

Person: But that- that's helpful. [inaudible 00:28:06] Yes. That definitely...

**Interviewer: Where? Number six?**
Person: That's 100%.

**Interviewer: Okay.**
Person: Yeah.

**Interviewer: So... Yeah?**
Person: Now, I don't know if it's because of myself and not releasing him so much that he has the ability to do that, but right now, no. No. He would always have to have somebody. He may be able to go by himself to an aisle, but for him to go by himself like, if he had STS and they drop him off to the market to go and come back, his situation is he just would go focus straight on that and may not be... Don't- don't pay attention to his surroundings. If somebody's in front of him, if someone's behind, he may bump into him. Like, a couple times, I've had that situation...

**Interviewer: Mm-hmm (affirmative)-**
Person: That um... 'Cause he always goes to the market with me, he knows where things at. So, if I tell him, "Oh Danny, go put this back" or if I don't want to buy something, he just takes it and he doesn't want me to leave it there.

**Interviewer: Mm-hmm (affirmative)-**
Person: He wants to go put it back. But then he hightails and go and then he'll bump into people, "Excuse me sir, excuse me" but he'll bump into people. But, since he's so big and he doesn't look like he has a difference... One time I had a situation that somebody said, "Hey dude, whatever" and I'm like, "I'm sorry" you know? "I'm sorry sir." And then he goes, "I'm sorry sir." Then he realized he was a little bit different. So, doing things on his own...

**Interviewer: Mm-hmm (affirmative)-**
Person: I don't think he has the capibil... Capability to do that.

**Interviewer: Right.**

Person: So, that number six is good and number three.

**Interviewer: So, I guess that's the question. I mean, do you think these types of questions are helpful...**

Person: Yes. Very helpful.

**Interviewer: As far as getting information to... 'Cause we're thinking about, you know, we could simply ask what type of disability, and that's an option too to consider. So, whether you like that or not, but the looking toward the future of how this information is helpful, obviously for evaluation purposes, but also for um, identifying what accommodations, if any, need to be provided in the healthcare setting. So, in thinking about what Danny might need, for example, in the scenario you gave me where like, the nurse did the calming and had time to get the needle in...**
Person: Mm-hmm (affirmative)-

**Interviewer: What other... What- what information would you want to either, you know, share verbally or check off on a- on a list that you would want them to know ahead of time**?

Person: Well, so far the experiences that I've had...

**Interviewer: Mm-hmm (affirmative)-**

Person: For that... How you're asking me...

**Interviewer: Mm-hmm (affirmative)-**

Person: They've told me to put any kind of inf... Anything that they need to know ahead of time. Like, when we had that situation for the dental, I had uh, uh, a me... Uh, appointment with them ahead of time so they could see him, observe him, and they tell any additional information. They have that on the form or they asked me and they put it down prior to scheduling his appointment for the dental. This is very helpful because then you could also add other...

**Interviewer: Mm-hmm (affirmative)-**
Person: 'cause then, like I said, there's two that doesn't apply to him. I could make that notation. They could ask that question and then I could put [crosstalk 00:31:02]

**Interviewer: Add another additional detail.**

Person: Yes. And for the same thing for number six. That's more helpful because then I don't have to worry about do I have to put down Autism? Am I labeling... Maybe that's something they could put on their notes instead of having to put it on that initial application. This is much more appropriate 'cause at least it gives me an avenue instead of, does he have [inaudible 00:31:23] and then you just have other. Or would I put other.

**Interviewer: Right, 'cause you want to distinguish it from an illness.**
Person: Exactly. It's not an illness, it's a con... It's his disability.

**Interviewer: Mm-hmm (affirmative)-**

Person: And- and that allows me to to put down... This applies to him... This- this concerns his characteristic, but not everything.

**Interviewer: Right.**
Person: That's how I feel.

**Interviewer: Mm-hmm (affirmative)- Okay. And...**
Person: Both of those.

**Interviewer: Anything else that is not captured in those questions that you would want to share**?

Person: No, this really pinpointed it perfectly.

**Interviewer: Okay.**

Person: Perfectly because for my experience, meeting a lot of uh, developmental uh, people...

**Interviewer: Mm-hmm (affirmative)-**

Person: It... This kind of more or less is like, like, most majorly that's what they're dealing with. Some of those high functioning, they can go.

**Interviewer: Mm-hmm (affirmative)-**

Person: Like, I know some of his classmates that can do this. They can go on the STS. The STS waits for them, they go and come back. Some of them go on the bus, I'm like wow. But, I noticed a difference. I could tell.

**Interviewer: Mm-hmm (affirmative)-**
Person: But I'm saying, wow, that's so great. And then I'm... Then I feel bad do I... Did I help... Hold Danny back? He coulda been like this? Coulda... I can't say coulda, shoulda, go back. I did the best and I'm doing the best...

**Interviewer: Mm-hmm (affirmative)-**
Person: But, this gives us, you know... Uh, knocks off so much label him. And not so much saying, oh he has this disability, has... Just to kind of give a guideline to what's his situation and what's his disability and to go forward to best help him. And then the doctors can ask the questions and they, you know, put their notation after those questions asked and after I put down... But this would be very, very beneficial.

**Interviewer: And do you think that that captures um, other concerns that you know, you're asked about, you know, is he gonna be aggressive? Do I need to tie him down? Do you think that those- those types of things fall into any of those questions?**

Person: Yes.

**Interviewer: Which one in particular do you think?**
Person: The number six.

**Interviewer: Okay. So, kinda doing things on your- on your own? Okay. So, that would be could I have a- a procedure done to me on my own or...**

Person: Exactly.

**Interviewer: Do I need [crosstalk 00:33:30]? So, you would check that mood. So, would you see this as something you would check on a piece of paper? Or do you prefer that to be something that's discussed in conversation? How do you see this information being collected?**
Person: Hmm. I guess it would be better on one-on-one. Only because... Unless if they put others and they have to list... I prefer doing it one-on-one.

**Interviewer: I mean, that would mean you don't have to figure out...**

Person: Yes.

**Interviewer: What to write down.**
Person: Exactly.

**Interviewer: Okay. Okay.**
Person: Because if... If they... If I... If they gave me a series of these questions... Like, suppose I was just this application, I would check off three and six.

**Interviewer: Mm-hmm (affirmative)-**

Person: And then from there on, they could ask me. Or, they could say any additional information or something. Other that you want to add and maybe I could put it down and then they can elaborate. Maybe they could do that. That could be...

**Interviewer: Mm-hmm (affirmative)-**
Person: There's not... Like I said, uh, everything is applies to him, but most of it applies to him.

**Interviewer: So, do you think these types of questions are enough? Or do you think there should also something that asks specific of what type of disability?**
Person: Yes. What type. I- I think so.

**Interviewer: To ask specifically?**
Person: Ask specifically.

**Interviewer: Okay.**
Person: At the get go, so they all know ahead of time what they're dealing with.

**Interviewer: Mm-hmm (affirmative)-**

Person: Yeah, so I- I think so.
**Interviewer: Is there ever any concern about sharing that information from the standpoint of being concerned that you might be treat... Or Danny might be treated different because of his having Autism?**
Person: In this... In the... In the... As far as going to the doctors? And the hospital?

**Interviewer: Any healthcare. Mm-hmm (affirmative)-**

Person: No. I think... I think that's beneficial.

**Interviewer: Okay.**

Person: 'Cause I think that will help them better treat him instead of just trying to figure out and trying to treat him and they don't really know?

**Interviewer: Mm-hmm (affirmative)-**
Person: If they know from the get go, they can refer me to somebody that- that... A colleague or someone that's better informed with Autism or whatever the disability is. No, I don't think... I think I'd prefer to put the information up front.

**Interviewer: Mm-hmm (affirmative)-**

Person: So they can at least at the get go. Don't... Not when I go there, make an... Then figure out oh no, I can't help him.

**Interviewer: Sure.**
Person: If they... Maybe they have the first consultation. They have the information. They see Daniel. And they move from there.

**Interviewer: So no... So no concerns about assumptions being made about someone with- with a disability or specifically, Autism? Because you've had kind of positive experiences? Or?**
Person: Because I have the positive experiences. I think it's great from the get go. Now that I'm having difficulties, um, I definitely want it to be specified that he has Autism then because I don't want him to... Sometime we think, oh he's fine. He looks normal. He talks. He does things by himself. He does... He comes and goes to the program. He loves to open the door, lock the door, not to go out the side... I mean, makes his own food. He makes his own bed. He fix his room. Uses the bathroom. Hygienically, he's fi... Hygienically... I don' know if that's the right word. His personal hygiene (laughs). Personal hygiene is- is great. You know, he does all... A lot, oh wow, he's good, he's fine. And then they'll pass him on.

**Interviewer: Mm-hmm (affirmative)-**

Person: But, maybe specify he's in the Autism spectrum would help um... It's s-s-such a wide... It's such a wide spectrum and there's high functioning, low functioning, you know, in between. Thinking kind of better... And there's a guiding tool for them to help them. Guide them to the right type of doctor to help them.

**Interviewer: Right. Okay. And um, you've mentioned before, you know, when they give um, positive feedback, you know, hey, you're doing great, you know, do you ever interpret that as anything other than encouragement? Uh, maybe like uh, um, a misconceptions about, you know, whether or not Danny's capable of doing anything like that? Or what... What are your thoughts about the- the intentions of- of those comments?**
Person: Like I said, I'm limited to the doctors.

**Interviewer: Sure. Mm-hmm (affirmative)-**
Person: So, so far those... Positive. Because they see the progression.

**Interviewer: Mm-hmm (affirmative)-**

Person: P-P-Progress. Talk about progress from what they advise him to do and when he comes back, he's improved.
 **Interviewer: So, it's based on that improvement more than anything else?**

Person: Exactly.

**Interviewer: Okay.**

Person: So, when they advise me, and they advise him, you know, and they come back, it's positive, so I say okay. We're doing the right thing.

**Interviewer: Mm-hmm (affirmative)**-

Person: So, it's a positive thing, I think. I haven't had any negative, so- so far...

**Interviewer: Mm-hmm (affirmative)-**
Person: This case that I have now, but prior to that... I think it's uh... Like, I haven't had like a... Like a... They brush me off type thing.

**Interviewer: Mm-hmm (affirmative)-**

Person: Like, it's not as if, okay, they gave me... Told me to do this and then just let me go.

**Interviewer: Mm-hmm (affirmative)-**

Person: And then they... They don't see a difference if I mention I see something that hasn't... That's not good or I see that he's too hyper or what have you, they think about it. They look at it and they move on. But, the positive um... The positive remarks that they give him...

**Interviewer: Mm-hmm (affirmative)-**

Person: I think are words of encouragement for him and for myself.

**Interviewer: Right.**

Person: 'Cause they engage with him so well.
 **Interviewer: Mm-hmm (affirmative)-**

Person: And they know him so well. So, and he feels... When I see my son feel comfortable with them, that's positive.

**Interviewer: Mm-hmm (affirmative)-**

Person: So, I don't... I haven't had an experience where they're just brushing him off until I'm saying recently now.

**Interviewer: Right. Right.**

Person: This just started, I would say, last year November?
 **Interviewer: Mm-hmm (affirmative)-**

Person: So, this is kind of fresh.

**Interviewer: Right. And would you say it's also... You know we've... We've kinda heard the phrase personal expertise being used to kinda express like the knowledge you have about how Danny likes to be cared for and what he needs and that type of stuff. Do you feel like that is welcome and your listened to and afforded time to share that? Or- or do you have any different experiences?**

Person: Like, I have... I've always had positive experience prior to now. That's why I think maybe I'm being a little harsh, because I don't see the same...

**Interviewer: Mm-hmm (affirmative)-**

Person: I don't see the same interaction with this particular doctor that I've had with all prior doctors.

**Interviewer: Right.**

Person: At this one doctor... Actually there was one doctor I had, but he was the best of the best, but... Doctor Tuchman. And then that's when Autism just started coming about.

**Interviewer: Mm-hmm (affirmative)-**

Person: And so... But, he was not so personal. He was just like a really clinical type doctor. Okay, Danny sit here. And this and this... He was really like, into what's going on with this child. He was so good that Dan Marino took him. And I said, "How the heck did he take... I finally found a doctor of this condition." 'Cause Doctor Geraldi said, "Go this doctor, he's the best. He's the one that's doing all the research who's a neurologist. Go to him." And he was doing... You know, [inaudible 00:40:07] he just wasn't as personal, but then Dan Marino found him. They opened up a Dan Marino thing in Broward and he was running that Autism... 'Cause you know Dan Marino has a child with Autism.

**Interviewer: Mm-hmm (affirmative)-**

Person: And then he referred me to "I have another partner who's just as good. Don't worry." I said, "Doctor Tuchman, you're leaving?" He said, "Yes, I have to go, but go... I have this doctor." I'm just so blessed because that doctor was much more personable. He was just into Danny, you know, talking to him. And he really was into... Just like doctor Tuchman, but just different personalities.

**Interviewer: Right.**

Person: Doctor Tuchman was more clinical and more this asking the questions and Doctor Resnick was more into him. Asking me the question, but interacted with Danny also.

**Interviewer: Right.**

Person: That was a [inaudible 00:40:50]. I forgot about that, I guess.

**Interviewer: So, tell me that it seems like there are two aspects that are important. The, you know, some understanding of- of Autism and what that means for a patient, but also just the... You know, maybe I'll say, good beside manner...**

Person: That's the word. Mm-hmm (affirmative)-

**Interviewer: Or- or- or just that person... That personable interaction. So, um, which, you know... If you had to like, talk about how important those are, you know, are they both the sa... Equal important? I mean, what are your thoughts about those two dynamics?**

Person: It is important to find a doctor that is... That knows about whatever your condition may be. I understand that part. And when you find it, it's very difficult to find. You want to hold on to it. But as uncomfortable it is for the caregiver, myself, or the parent, it's uncomfortable for the child, you also want to feel comfortable. 'Cause you don't understand what they're talking about really. You try to understand and try to find the best way... Now you're making me tear up.

**Interviewer: Oh no, I'm sorry.**

Person: That's okay. Trying to find the best way, but then you need that comfort. You need to feel like, okay. I understand what he's talking about. My son is feeling comfortable, it's not like okay here, touch your knee or you know, it's not... It's more a little bit personal. That bedside manner is- is very important to me.

**Interviewer: Mm-hmm (affirmative)-**

Person: It's very... 'Cause if I am uncomfortable, my child's gonna see that I'm uncomfortable and then he's gonna feel uncomfortable.
 **Interviewer: Mm-hmm (affirmative)-**

Person: Oh god.

**Interviewer: Oh. Well, I'm gonna... I'm gonna cut it there, unless you have any other thoughts that uh, I- I... You want to share that I need to know about?**

Person: I think I shared a lot. This was a... I'm just blessed that I'm able to... That you guys called me to do this because...
