# Supplementary material for: Disability disclosure in healthcare settings for individuals with developmental disabilities: A qualitative study of patient and caregiver perspectives
Source: PLoS One. 2025 Aug 7;20(8):e0329328. doi: 10.1371/journal.pone.0329328 (PMC12331114; doi:10.1371/journal.pone.0329328)
Supplement: S1 File — (ZIP) [file pone.0329328.s001.zip › Transcripts/2019.09.11 Interview 03 Transcript.docx]

**I: Interviewer**  **F:** Female Key Informant

**I: Alright, and I will ask just for the record. We’ve gone through informed consent and you are, uh are okay to proceed to be recorded?**

**F:** I am.

**I: Alrighty. Uhm, so let me ask one thing that I didn’t get on the eligibility screening. How old is [NAME]?**

**F:** He’s fourteen.

**I: Fourteen okay.**

**F:** He’ll be 15 soon and scary! I guess every year is scary. (laughs)

**I: He’s a teen! Each of the teens.**

**F**: I remember when he was turning ten, I was like ‘oh my god double digits. What am I going to do?’ They fly by.

**I: Mhmm.**

**F:** We are already at 14.

**I: Mhmm. So, so tell me uhm, when you think about all of his health experiences, health care, doctors, whatever, nurses, all of the above, hospital outpatient, would you say you got good experiences, bad experiences, both?**

**F:** Uhm, I think and I think I probably mentioned this a little bit, uhm at the uhm meeting, but I think I try to make them the best possible because I try to be proactive. By you know, calling, I want to know exactly the length of the appointment, I try to make the best of like, when there is less of a crowd, even if he has to miss school, you know I’ll ask which days are you know, easier for them to be able to give him a little bit more attention. Uhm…. And if there is a procedure, I try to find out details so that I can also explain to him and give him some information. So he is not just going in blindly, you know. Uhm, so I guess in that sense they haven’t been terrible but for example if we have to do blood draws, that’s an issue. Because I mean, even as I explain it, they’re just going to sit him down, tie his arm down, and they really try to you know, you’re just the next person. They’re not really about telling you a story, especially at a lab.

**I: Mhmm.**

**F:** I, I feel that is more of the environment. It’s just, next arm, next arm, next arm. And the situation is so it’s been a little bit dicey. But, like I said as far as doctor’s offices I try to be, I try to get ahead, I guess.

**I: Proactive, mhmm.**

**F:** Yes, so they haven’t been terrible. But I did have like an outstanding experience with a dermatology, uhm, center. And the doctor that was uhm tending to [NAME] and the nurse, like all of them, uhm, he’d have to have a procedure done that we didn’t know about, because she just decided there that it would be uhm, a good idea to do a biopsy, so there was no preparation on our end, and they still, they gave us a few minutes to tell him about it, they spoke to him. On his level. You know? And not even, this is the first time, like even seen him so I was… impressed. And uhm, they just went with his flow basically which was to me it was, I was... impressed. I was really impressed with their, you know, with their work. So uhm, and they didn’t know prior, you know, other than the receptionist, I did, you know tell her a little bit about his condition but,

**I: So you, you don’t, you didn’t even, you weren’t even certain they were aware he had autism?**

**F: Right, I don’t know if the doctor was aware, because I try to tell whoever is making the appointment, but I don’t know how that is relayed or not.**

**I: Right.**

**F:** So I mean it may have been or not, but we had a great experience that as above and beyond, even from his pediatrician office.

**I: So when you talk about being proactive, what uhm, what do you share, what type of information do you think they need to know with regards with [NAME] having autism?**

**F:** Well so I start just saying that he has autism. And then wait (laughs).

**I: Mhmm**

**F:** And see if they are familiar. You know, uhm, because usually if they are a little bit aware they’ll ask a little bit of questions. But if not then I just keep giving them, so I offer the condition and then I’ll ask, I’ll tell them he has a hard time waiting, uhm, if it’s a crowded room, you know he gets a little bit antsy. Uhm, he won’t try to sit there you know for thirty forty minutes to wait for the appointment. Uhm, if it’s a little bit easier once we are inside like our own...

**I: Exam room?**

**F:** Exam room, uhm because then you know, he gets up and about or whatever. Uhm, but yeah I, I try to ask uhm, what the setting looks like especially if I haven’t been there before. And uhm, how long the appointment after, you know, once he goes inside, how long does that usually take?

**I: Mhmm**

**F:** Uhm, what procedures are offered there… uhm, you know so if its, you know his pediatrician’s office, I know they don’t do labs for example, but if he’s up for a vaccine or something like that, you know I want to make sure that I know what the length of waiting is, uhm to get things done. Uhm… even little things like if he has to be changed into a gown.

**I: Mhmm.**

**F:** You know because there’s things he doesn’t like to be touched in certain places.

**I: Mhmm.**

**F:** Or at least you have to warn him that you’re going to be doing, you know, some assessment.

**I: Mhmm.**

**F:** He doesn’t like to be in a supine position

**I: Mhmm.**

**F:** So, uhm, like at the dentist, there’s a certain degree that once you pass that, he starts trying to sit up, so that could be dangerous you know.

**I: Right.**

**F:** So little things like that.

**I: Mhmm.**

**F:** That Ill, like at the dentist I’ll explain certain things that the pediatrician.

**I: Mhmm.**

**F:** You know depending on the doctor I guess, you try to ask the questions

**I: So do you feel that whenever you share this they’re very receptive to this information or put it to use?**

F: Uhm, receptive in the sense they’re like ‘ah ah’ or like ‘I’m writing things down’ kinda thing. Put it to use? I’m gunna say no.

**I: Okay.**

**F:** Like at the pediatricians they know. I mean he, from the time he was diagnosed actually, I changed pediatricians right after that. Uhm, but he’s been going to this one since he was about three and a half. And uhm, I still have to every time I make an appointment, listen you know the situation is this..

**I: Just to, kinda like a reminder? Mhmm.**

**F:** What day is better? And, but it doesn’t change because we’ve sat in the waiting room for 30, 40 minutes.

**I: So they’re not, they don’t, not necessarily trying to accommodate or able to accommodate?**

**F:** Right. Or maybe they can’t. To be fair.

**I: Mhmm.**

**F:** So ya, not, not all the time.

**I: And are there any instances where, I don’t know if this applies since you say you’re proactive, where there, they don’t know this type of information and they try to get it from you, whether in a paper just or in conversation?**

**F:** No.

**I: Okay. Is that because you kind of beat them to the punch or they just don’t do it?**

**F:** Maybe? But I feel like I am always the one, to you know, offering, or having to state or I guess I state...

**I: Or I guess, going back to the how they respond, do they kind of engage you in conversation to ask more understand better the information you’re sharing with them?**

**F:** Not really.

**I: Okay.**

**F:** You know if I said, he has, he has a hard time waiting or whatever, the most I’ve gotten is “okay well” because then I say, that’s why I need either the first appointment or the last appointment of the day, so that, you know that even if he is waiting it’s not a crowded room. Then they’ll just be like ‘well we have 9:30 available.’ You know what I mean, that kind of thing, so.

**I: And do you tend to kinda of say it not only to the front desk but everyone you come in contact with?**

**F:** Yes.

**I: do you get a sense that they… heard it from.**

**F:** And I think it’s because I’m proactive too.

**I: Right.**

**F:** But uhm, yeah because I’m not sure how they, like I said, how they relay.

**I: Right. Do you ever get a sense that someone’s like ‘oh that sounds familiar because so and so already told me’ or anything?**

**F:** Uhm, not really.

**I: Not really? Okay.**

**F:** No not that I can remember.

**I: Mhmm. Mhmm.**

**F:** And I try to remember those things because I’m like “oh that’s a good doctor” or “that’s a good nurse” you know because you always need a reference.

**I: Right. Sure. And you, you mentioned changing doctors right after the diagnosis. Was that intentional or it just happened?**

**F:** SO uhm we... he went to a 15, I believe it was 15-month checkup and uhm, the pediatrician assessed him. He was a very good pediatrician. He assessed him and he, uhm, handed to me a prescription paper that said, ‘PDD’ on it. I had no idea what any of this was.

**I: Mhmm.**

**F:** I was not, uh, aware of what autism was, or what PDD meant or anything. And, he asked me a few questions, you know, how many words [NAME] had, and things like that. Uhm, and now I know I didn’t know at that moment that he was assessing his tracking, you know with eye contact, joint attention, that kind of thing. I didn’t know what he was doing. But he gave me the prescription and I basically, I was upset.

**I: Mhmm.**

**F:** You know I wasn’t explained what was happening. What he was thinking. And I’m, after, uhm, I realized he just wasn’t 100% sure he wasn’t diagnosing, him, he was, you know he was recommending him-

**I: You don’t want to say anything until he knew, maybe kind of thing?**

**F:** I’m not sure, I’m guessing.

**I: Okay.**

**F:** Uhm, so I went on to, he uhm, the pediatrician we moved to was before his partner. So I knew him already. So it wasn’t like I was going to someone completely new.

**I: Mhmm.**

**F:** Yea I did leave because I was upset.

**I: Right. So did, were you aware of the partner having like greater knowledge about autism or something like that? Or you just wanted to kind of make the move?**

**F:** No. **I was just upset at the old doctor.**

**I: Right okay.**

**F: and I did write him a letter after because I felt bad. (Laughed). I think it was just my mom reaction, like ‘no, what did you see in him” you know?**

**I: right**

**F: but now, I don’t think that he has a greater awareness, uhm like I said even for our regular visits, I just feel like I have to remind them, you know, uhm, but I, its someone that I already knew so I felt comfortable with him, as seeing eyes.**

**I: And when you, when you see interactions when you go in for appointments does it seem like the staff as whole has a decent understand of autism and what that means for giving care?**

**F:**  Uhm not really. He has the original pediatrician we went to. Uhm, has a new partner.

**I: Mhmm.**

**F:** And is a female doctor which doesn’t make a difference doesn’t matter, but uhm she seems to ask more questions. So now she is the one seeing [NAME]. The last couple of years she’s the one that has been following [NAME]. And she does uhm, ill mention things and then she will ask follow up questions. Like she seems at least more interested.

**I: Mhmm.**

**F:** In what’s going on and what he’s doing.

**I: And so do you like that, asking questions?**

**F:** I do like that; it makes me more comfortable of course

**I: And when you say you have to remind them, what are the interactions as far as them getting it right, let’s say interacting with [NAME] and also with you?**

**F:** Right, for example, we walk in to the appointment aside from the waiting room, like once we are called back, it’s probably just one of the techs or one of the nurses just getting his weight. For example, and so what I do, because you don’t always want to be obvious, you know at least for me, that’s the way I see it. So I’ll try, she’s talking to him like normal. Or like, I shouldn’t say normal, but you know she’s just saying it matter of factly, ‘hey, you know, we need to get your weight get on the scale, ‘take of your shoes’ you know, and I’m like, “[NAME]”. So I go near him and I speak a little slower. And I say “[NAME] the same thing she said, but just so, that they’ll know that they’ll notice. You know so I try to be subtle like that. I don’t want her also to feel bad, because maybe she doesn’t know

**I: Right.**

**F:** You know, or whoever it is.

**I: Or did you ever see them maybe observe that and maybe follow your lead going forward or?**

**F:** And then she does.

**I: So. And do you think in light of that example, do you have to present for the appointment, or could you stay in the waiting room?**

**F:** Uh no. I feel like I, I feel like I have to be.

**I: Okay. Okay.**

**F:** yeah

**I: And do you ever find, I mean do they tend to focus their attention on [NAME] when they are communicating or asking questions, or do they tend to involve you as mom?**
**F:** Uhm, initially, they’ll try to talk to just me, once I’ve disclosed something. Uhm, but, I want to also say that new female doctor, we don’t go like every month to the doctor, so she maybe sees him once a year. And maybe two if something else is going on. So I understand that too, so, it, once we are in the exam room and I you know, remind her, she does address him a lot. And I, and I like that.

**I: Right.**

**F:** She’ll say ‘mommy’ you know whatever, and then she’ll say, nope, you know she actually speaks too him.

**I: Mhm.**

**F:** Which makes a difference too.

**I: Mhmm.**

**F:** For him. As well.

**I: So that’s more so her than the other doctors?**

**F:** Yes. I’ve noticed it.

**I: Okay. So do you think it’s her inquisitiveness more than anything or?**

**F:** I think so, uhm, also and I could be wrong but I think it’s also a generational thing. She’s a younger doctor than the original pediatrician that we used to see so uhm, that may be a factor.

**I: Okay good to know okay. Uhm, are there any other things you can think of like sand out as you know, problematic iterations, or hoe you were treated or how he was treated during any type of procedures or any type of healthcare interactions?**

**F:** Uhm, uhm problematic, I don’t, I mean, I think just in the sense of all the legwork that has to be done before hand, and even at like an appointment if they’re not aware. Or if I don’t step in and do the subtleness.

**I: Right if you take your proactiveness out of the equation what would the experience be like type of thing.**

**F:** Right, and I haven’t really tried to do that. You know because-

**I: Because you don’t want to know (laughs).**

**F:** Well, and also I feel like, I think, I think for any of us not only for [NAME], when you wonder, when you go to the doctors, when you wanna get out of there. So I haven’t really tried to just let it be and then have something happen than have to back track. Especially if [NAME], you know has a tantrum or something like that. You know it’s just a negative for everybody involved. Including him and I always try to make the doctor visits positive.

**I: Mhmm.**

**F:** And so, to the point he likes doctors a little bit too much (Laughs) and I’m the opposite. He’s always like “Mommy my throat hurts, I need to go to the doctor, I need medicine.” And I’m like oh my gosh [NAME], yo have no idea how much work that is to take you. But yeah I think that’s the result of us constantly stepping in and yo know, I had, I don’t do it so much anymore, but when he was younger I had a little summary of like this is who I am, you know. I like video games, whatever he was doing in the moment, that I would bring with me because I felt like, you know they always ask me and I understand they can’t understand him a lot of questions but it’s always like they don’t know him. You know?

**I: They don’t, do they, do they try to get to know him?**

**F:** It’s rare. It’s rare.

**I: Mhmm and you think that’s because its-**

**F:** Aloof maybe, that he’s quiet.

**I: Okay so they’ve never really tried to, you know I guess if they don’t even know anything about autism, do they try like they would for another patient and then realize they’re not getting it right or do they not even try at all because they found out he has autism?**

**F:** I think I’ve only seen that with female doctors the most. Uhm, that kind of interaction, where she is in between mommy questions, she will ask him “Have you seen this movie?” you know and obviously even if he doesn’t answer. I will say “No we haven’t” And she will say “Oh but you’ll like it” or she will go back and make another comment to him. But specifically about the autism, I think it’s just whatever I offer is what-

**I: What they go with?**
**F:** What they go with.

**I: now on the flip side on the terms of, have you had any really bad experiences that might have caused some issues that come to mind that you want to talk about**?

**F:** That was just, that was just my perspective of it, was the initial pediatrician assessment. But uhm, in high and sight, it was not, you know, I don’t think it was a, I just…

**I: Well going back to that, what, what, how should it have gone to make you as comfortable and not unnecessarily worried, given that the process of-**

**F:** Right and I’ve gone back and forth about that. Because I have thought about it and im like, ‘what if he would have said’

**I: Like would he have freaked out? Like what, or what would be the ideal, like what should he have said or how he should have handled it?

F:** I don’t know. I don’t know. I have thought back like I said I felt bad about thinking ‘he was so mean!’ because that’s not fair. What if he would have said autism and I would have gone on my phone and gone crazy on him or something. I don’t know. I, I, like I said, in high and sight it was fine. It was just like my motherly, you know, ‘there’s nothing wrong what are you doing’.

**I: Sure sure. So because you, you’ve never really had any negative experiences its more about your proactiveness and preparing and also explaining. So no, no instances where someone accidentally tried to touch [NAME] or he was uncomfortable with, or you know trying to stick a needle before preparing and then it being like that didn’t happen because of your proactiveness would you say?**

**F:** I think so, there was one time not that I think about it when he was turning seven, you know how they start losing teeth, well his tooth that was supposed to come loose never came loose and his permanent tooth was coming in. So now he had two layers of the tooth. And I was like oh that’s not okay so I had to take him and this age I was dreading always doctors and even more dentists so. When I called, and I went ahead and I called, I’m sorry, to make the appointment, they said okay ya come in we will look at him. And I didn’t disclose what I usually disclose about him. Uhm, the waiting was minimal I remember, uhm, everybody in the office was nice, but once the doctor came in, uhm, she is nice but then she nope, we need to remove the tooth, you know right away because then that, the permanent tooth will move in, will slide, whatever. And we just went with her whatever she was suggesting.

**I: Mhmm.**

**F:** And all of a sudden it was like five nurses in the room. When including me and dad. At the time. Oh my god. That was, that was actually a terrible experience. And they just came, it was like a team of people, that came in with like a (inaudible) and they basically they wrapped him up, no questions, no ‘we’re gunna do this’ he needs to be, this is what’s gunna happen, and everybody just came in.

**I: with no explanation or?**

**F:** No. No no. My husband at the time, [NAME]s father was livid. And he actually stepped out of the room because he was crying any everything. [NAME], was screaming and crying and kicking and I’m there trying to hold his head while, you know there’s six people and one lady with two hands in his mouth. I guess that was a bad experience. And yeah, they, they did not…

**I: This was probably in addition to you trying ot prepare them about how you need to prepare [NAME]. And they still-**

**F:** You know, (inaudible). You know little schpeel that I always give.

**I: But it fell on deaf ears?**

**F:** But I guess, I guess the doctor, once she was, maybe she did know or she didn’t, I guess she didn’t because of the (inaudible).\

**I: That was trying to restrain or?**

**F:** Yeah, because I’ve seen other little ones and they weren’t or anything. But maybe she felt he needed, because of his circumstance?

**I: And no one asked about whether he needed to be restrained or?**

**F:** No, she asked us if we were okay with having the tooth removed?

**I: The procedure.**

**F:** Well, ya, we will see how we can do it. And that’s when she, all of a sudden she, took out the room and when she came back it was like five people in there.

**I: And do you think that five people, obviously you’re not there for every procedure for this, but did it seem like five people was their standard practice? Or they did that because they were concerned about restraints?**

**F: No, it was because, I guess his condition or they, I, I have no idea.**

**I: Mhmm.**

**F:** Maybe they weren’t expecting what actually happened.

**I: Mhmm. So they didn’t really ask you whether needed to be restrained or whether you were okay with him being restrained and the process to explain to [NAME]?**
**F:** No and I did write reviews about that.

**I: Mhmm. Did you write that directly to them or-**

**F:** Yes. Directly to them, and I believe it was Yelp or something

**I: And did they respond to that feedback?**

**F:** No. No.

**I: And have you returned to them?**

**F:** No, I try to warn other people. Other people say they do love the place and that they do accommodate to special needs. But I guess it depends on, I guess, all the children are different so. I don’t ever say don’t go there, but I try to say this was my experience and just so you know, you know.

**I: So did, did, did they, did you, did he get through the procedure?**
**F:** Oh they did what they have to do.

**I: And how was [NAME] after that?**
**F:** Oh it was bad. Even like little like blood vessels that burst around his eye. And that stayed. That remains to be every time he gets a little bit upset you will see he turns purple.

**I: Mhmm so, so if we could repeat that situation but in the right way, how, how would it have gone down?**

**F:** Well for sure explaining that there would be ten people in the room it would be one. Uhm, the part of the (Inaudible) I don’t think I would have been opposed to it regardless. But I think the way they, they literally before I knew it he was (inaudible) the lady, I’m sorry, the doctor was in his mouth. And so, I think you needed to give him a heads up, I mean. Because I’ve, I’ve seen with other, not necessarily the dentist, uhm, but I have seen with other you know, appointments and procedures that I can tell him, you know a little bit of what’s in his own, words. I can explain to him you know a little bit of what could happen or, you know that he needs to you know, just relax as much as you know, he could. But yeah little bit of uhm, more of an explanation.

**I: Mhm. And do you think it’s, it’s like the you as the caregivers uhm responsibility to play that role in making sure that the patient in this case your son is, is relaxed or do you think that your kind of filling that role because no one else is or is it the responsibility of the health care provider? What are your thoughts about how that dynamic, given that you know what you know about your son best, better than anyone but there also the health care provider?**

**F:** Well I always feel like it’s an obligation of mine. Uh, and I guess I do expect that if I go in with some information to help you, that you are able to help me with you know at least following my lead right? So even if you’re clueless, you could at least like I said, follow my lead or even ask questions to you know. Uhm, because they you know obviously out of respect for them I, don’t, I don’t tell you you don’t know what you’re doing ever because I’m the one coming to you. So the doctors know their field and respect that always. But what they don’t know is [NAME]. And so that’s, I always try to give them like a glimpse of what he is. So that he can treat him the best way possible. Best, at least that’s my rule. But yeah I don’t really feel like it’s my obligation. Id not think anybody is going to know. And they can’t ask him the questions to get to know him.

**I: They don’t, you said they can’t, they can’t ask them you’re saying?**

**F:** Right, well in the sense they can ask but he probably won’t be able to respond.

**I: Okay, uhm-**

**F:** To the questions.

**I: And is that because they’re not asking in the way they need to be asked or he just wouldn’t be able to?**

**F:** Some things just, he wouldn’t understand.

**I: Okay. So, so going back to you know I know you said you’ve had to remind people on occasion of what you’ve already told them. So when you do share, specifically after the front desk and you’re interacting with a nurse or a doctor or a PA, or what have you. Do you find them receptive? How, how do they receive the information? Do they seem receptive about it at all?**

**F:** Yeah I think so for the most part. I’ve never had anyone say to me oh just sit down don’t worry about it. And I may, been asked where he goes to school or if he’s in some kind of therapy. Uhm, and I usually get oh he’s doing really well. Uhm-

**I: They say that to you?**

**F:** Yeah

**I: Okay.**

**F:** So I guess, I guess that’s receptive.

**I: Okay so, receptive would also maybe whether they’re asking follow up questions. Like you said the one younger doctor or applying the information you give them. That type of which you said maybe less so.**

**F:** Yeah.

**I: Mhmm. Okay. And when they do say he’s doing really well, what are your thoughts on maybe the intention of those statements or any assumption behind it. Do you think it’s a good nature well intended comment?**

**F:** Oh yeah of course.

**I: Do you ever, ever think there’s any assumption underlying it about whether or not they think that [NAME] is capable of this or that? Or what are your thoughts when they give those?**
**F:** In general I think it’s just a well-intended, you know, because he does you know for the most part sit, uh, you know, uhm. He just looks typical. You know and he-

**I: Typical as in what sense?**
**F:** In the sense he can sit. You know next to me when the doctor is speaking to me. He may, he makes an utterance, or some vocalizations. Of some sort. But for the most part he just you know, he’s respectful in the sense he’s not trying to slap people and walking around or uhm, I think, in the general sense he doesn’t have uh, a lot of the stereotypical uhm, autism behaviors. Uhm, just because uh I’m constantly giving him like subtle cues. Uhm so he, he has uhm, very little flapping or clapping or uhm, things of that nature. I mean if you leave him to his own for a couple of hours in his room, you’ll see some of that but for the most part if he’s sitting here next to me, and he’s enjoying some breakfast or something or we are just talking for a few minutes, you will maybe think (inaudible) unless he’s trying to engage in conversation maybe. So I think that’s where that may come from. You know those comments, uhm, and yeah they may very well think you know, he, well he certainly behaves well, at least in those moments. He can certainly I’m sure he does well in school. You know that type of thought. You know.

**I: And uhm, thinking about not just the health care providers themselves and those interactions, the physical environment, is there anything about the physical environment that is relevant here** **that may be helpful or, or, or hinder interactions? When you go into the health care setting?**

**F:** Uhm-

**I: Whether it be lights, noises, anything like that?**

**F:** I don’t think so, uhm, he, [NAME] has his own little things that he will stim on, but I mean, it, a lot of most doctors’ offices cater to children and adults and, so there’s little things that for me I would rather not have in there that’s not, not I mean, not a problem.

**I: Just out of curiosity, what would be those things that you would rather not have in there?**
 **F:** All those little toddler toys that spin. He had, he had a very big stim when he was young for spinning objects. Uhm, and he would just, it, it was uh, rapid spiral downward. Uhm, where he would just, have outbursts all the time.

**I: When playing with these toys?**

**F:** Yes, for a long period of time.

**I: Okay.**

**F:** So, uhm, but like I said, if, if that little box is stimming stuff is on one side of the room-

**I: Then you’re fine?**

**F:** Then, then I can tell him you know we are not playing with that right now. We are here to see the doctor or whatever his ailment is. You know. And for the most part its fine. It’s fine. But I think other than that, I guess it’s just the outs, the space.

**I: The number, so depending on the space so if it’s a small space (inaudible).**

**F:** But the actual settings I mean; I think-

**I: Nourishes. Okay. Uhm, so why don’t I kind of transition to you know the work we are doing (inaudible) what we talked about before. We are talking about if we are trying to dig through the health care experience for all types of, people of all types of disabilities, what things that we start off with is well we don’t even know who has what because we don’t traditionally routinely ask. So there’s the question of you know, should we ask? Like is that an appropriate thing to ask? And if so, what** specifically? So what are your thoughts about should we ask and what should we ask?

**F:** I was thinking about it, and I was thinking, is it the same as, a HIPAA violation you know, are you, are you allowed to ask? You know. Like, what is your gender over the phone, uhm, or even on an application you know, that’s for me , personally, I don’t have an issue if somebody would just outright ask, does he have a, a mental disability, does he have autism, like go down a chart list, for me that’s not an issue. And I think that actually would help.

**I: Mhmm.**

**F:** Uhm, like I said, I don’t know lawfully what is-

**I: Maybe not over the phone but it may be over an online portal or something that would allow for the confidential-**

**F:** I think any way I would be open to disclose.

**I: Okay. So you definitely would want to say what type of disabilities. Is there, well, let me assure you. This is a starting point not a suggestion. This right here is six question s that are used by the US census to ask about disability status. So not really intended for the health care setting.**

**F:** Right right.

**I: So keeping that in mind, as a starting point, this is how they, they’re not asking what type of disability, they just what to know what type of difficulties someone has. So the first one being deafness. Then, uhm, blind, or serious difficulty seeing when wearing glasses. So I would guess those are no for [NAME]. Uhm, because they lump physical mental emotional conditions, do you have serious difficulty concentrating, remembering or making decisions.**

**F:** Yes.

**I: That would be a yes for him? Okay. How about do you have serous difficulty walking or climbing stairs? No. Do you have serious difficulty dressing or bathing? And then lastly, because physical mental or emotional condition, do you have difficulty doing errands alone, that would be like visiting a doctor shopping?**

**F:** Yes.

**I: That would be a yes, even, even if he wasn’t a minor or, or because he’s a minor?**

**F:** Even if he wasn’t.

**I: Okay. So, so tell me what you think about these questions. Are we asking the right types of things, is the working appropriate? What are your thoughts?**

**F:** Uhm, I think in general it covers a lot. Uhm, I think the physical mental emotional is kind of clumped.

**I: Okay.**

**F:** A little bit.

**I: And it should not be or its okay that it is?**

**F:** Uhm, well I guess, I guess it would be fine if you could detail, what you know-

**I: So like a follow up for additional information.**

F: Right so if its physical, what exactly. Because if they need a wheelchair or ramp or they cannot get out of their wheelchair, I don know. Something more specific. The mental as well.

**I: Mhmm.**

**F:** Because I guess that’s where [NAME], I guess, that’s would be the slot that [NAME] would fall under. Uhm, but also would have to be detailed. Like what does that mean? You know because for him its difficulty concentrating, focusing, uhm, like going back to the example of the pediatricians, if I made the appointment and I just let him go into the appointment, he could maybe wander the offices, go into other exam rooms. Just, because he is curious, but if you don’t know that, you know what I mean so. I guess more specific uhm, question about like each because I’m assuming, physical there could be a million-

**I: Mhmm. So there has to be some follow up to capture specifically-**

**F:** I, I think that would be the best thing.

**I: IS there anything thinking about going through as you know you said, your spheel, what you tell people, what of all those things you would want to share, are, are those captured here and the detail, or is there something else we should be asking.**

**F:** Well I think, like you said this is not necessarily, uhm, you know for health care. But because we are talking about health care appointments and things like that I think the nature of asking how long usually the appointments are, what the usual wait time or the average wait time is. Uhm, offering if there is a disability or, you know some type of physical uhm, issue uhm, offering early appointment or a certain day that they would open it to you know-

**I: So you’re kind of saying like tying these to accommodation’s they could provide. So, so there has to be something on that end to. So it’s not just collecting information its doing something to acknowledge you’re applying this knowledge in a way that helps them.**

**F:** Yes. I think that I would find that beneficial.

**I: Mhmm. So you said, or first or last appointment, anything else how and you said, information about how long an appointment is how long do I have to wait in the waiting room vs. in the exam room?**

**F:** Yes, uhm maybe also uhm, if, it is for example like a dentist appointment and you already knowing that you’re going in for a certain procedure, maybe l like the type written, a few steps of what it is that they’re going to do. Uhm, then we can, we can then relate to our loved one in sort of a social story, kind of way so that gives them al little bit of a heads up. And also for us, you know.

**I: So that’s a great example. So in that sense that explaining to reduce anxiety. Where, where do you see that captured anywhere? Or would that be something else we would want to ask separately?**
**F:** I, like I said, I think because its clumped into physical mental-

**I: So you think that would be captured in number three because it would be associated with any** **issues with concentration, remembering of making decisions?**

**F:** Yeah. Definitely.

**I: Okay that would be emotional or the mental. For that question?**

**F:** Yeah.

**I: Okay.**

**F:** And also because its asking here, making decisions, uhm, also to be on the front and they should know if the person needs a caregiver or a guardian-

**I: Present with them.**

**F:** Present to go through the appointment with them. And make a decision should a diagnosis be, needed or, you know. I think that’s also-

**I: And also what about you said [NAME] doesn’t like to be touched in certain places or certain ways, do you see that being captured in any particular questions. Do you think that’s kind of missing or?**
**F:** Yeah I think tis kind of clumped. So I think for me if I saw this I would write a whole paragraph down here and say the mental applies and why. And so that also includes sensitivity to tough, and I would really explain (inaudible) things like that. That I think that would be necessary, for the dental-

**I: That specific setting and making them-**

**F:** Medical, medical setting.

**I: Mhmm, uhm so, so they uh, yeah.**

**F:** Definitely, follow up.

**I: So a lot, so do you feel like we should be asked, in, in a way like this where it’s like a yes or no and provide details or do you feel that. Like you said you’re writing a paragraph and that wouldn’t work, or what’s the best way to ask this. Is it like this? Is it just in a conversation so they can write it down?**

**F:** No, I think it should be both.

**I: It should be both? Okay.**

**F:** I think yea. I think that if we have to answer this and if you’re saying yes, you can’t move on to the next question until you explain.

**I: Give a little- and then when you say both you mean?**

**F:** Because then there’s no point. If you say yes, you’re not really offering information.

**I: Useful detail that they needed-**

**F:** You cannot move on, you need to say why. And uhm, and then of course if they’re speaking to somebody over the phone also to be able to explain it.

**I: Mhmm. Right so this could be, then they use this to have a conversation to make sure they have the detail. So, what that means for and maybe a conversation about what they’re going to do in light what you share with them?**

**F:** Exactly. This could be like a set of tools. Like they already know, this person is anxious, this person is you know, doesn’t like to lie (inaudible). So you know we are going to assess your belly, the pediatrician, for example that we have, no no no, you can stay seated in your chair, he doesn’t like to take off your shirt. Uhm, we have gotten to that point now, but before she would be okay with just pulling it up. You know and she could assess from there.

**I: And he’s okay with that and she can do what she needs to do that way.**
**F:** Right and because there was communication. That was the important thing because if she just went in there, take off everything, put on the gown, that was not going to happen. And she was not going to be able to assess him. And he was not going to be better.

**I: Mhmm.**

**F:** You know, so. The communication and the you know, cooperation on both ends, because like I said, we have to be respectful that the doctors uhm are doing their job. You know and are trying to help us.

**I: Right but they also need to be-**

**F:** They also need tools.

**I: Mhmm. So you’re saying this is kind of like the way of, so they have their tools and this is the way of telling which tools are needed today.**

**F:** Just like the doctor needs to know, you know if your blood coagulates a certain way, you have a certain condition. If you don’t let him assess your lungs, he doesn’t know you have some upper respiratory or whatever, so you know, you need to be able to get to the patient.

**I: And so we are collecting that type of information right now but not necessarily the right way. Have you been asked anything like this in any kind of form or anything like that?**

**F:** No.

**I: It’s all just what you voluntarily share.**

**F:** Yeah. Mostly it’s just medical questions. Uhm, medical history, uhm I think there uhm, I did see on, on the form something of uhm, is there anything that you would like to add or any comments or something and that’s where I usually will make my rant-

**I: That’s where you put whatever.**

**F:** But not specifically.

**I: Right. Okay.**

**F:** So I always see that as just, they needed to finish the paper and just you know make it look complete. But I don’t know how far that goes you know being related-

**I: Right do they actually put it into-**

**F:** Right do they see that as an important thing or she actually made a comment. And lets file it. Uhm, but if it’s an actual part of the medical intake-

**I: Like the medical record.**

**F:** Right, it’s very important.

**I: Okay and how often should it be assessed, these types of questions. Is once enough should it be done on a regular basis because things change?**

**F:** Yes, I think, I think every uhm, maybe uhm every year or every six months. Because like I said I don’t think any of us go to a doctor every week. But they do change just like we do

**I: So if you see them once a year, definitely each visit. Just to kind of uhm, like you would at other things. Is your blood pressure still or what not?**

**F:** Exactly.

**I: Okay. Okay. I think those are. Those are all my questions, unless you have any other last thoughts that you think I need to know about.**

**F:** Uhm, no, I really appreciate you doing this, because I think-
