# Supplementary material for: Disability disclosure in healthcare settings for individuals with developmental disabilities: A qualitative study of patient and caregiver perspectives
Source: PLoS One. 2025 Aug 7;20(8):e0329328. doi: 10.1371/journal.pone.0329328 (PMC12331114; doi:10.1371/journal.pone.0329328)
Supplement: S1 File — (ZIP) [file pone.0329328.s001.zip › Transcripts/2019.09.13 Interview 09 Transcript.docx]

**I: Interviewer**

M: Male key Informant

F: Female key Informant

**I: Just for the record now that we're recording I want to just acknowledge that we went over the informed consent And you agreed to participate and to be recorded. Is that correct?**

M: Absolutely!

**I: Alright thank you very much! All right so you have been to the doctor and other Healthcare settings yes?**

M: Yeah

**I: So tell me–What settings have you–so you’ve gone to the doctor Have you ever been to the hospital, or the dentist, or anything else like that?**

M: Yeah I'm pretty sure that there are sometimes when I visited the dentist.

**I: Okay okay so in thinking about all those times that you went to the doctor or the dentist or any of those experiences, um–would you say that you've had any bad experiences?**

M: Um...No not exactly

**I: Okay so tell me when you say not exactly, what does that mean?**

M: Oh, as in not even once!

**I: Okay, not even once! Okay, okay. So you'd say your experiences have been good?**

M: Yes indeed!

**I: Okay so can you tell me a little bit about, um, like what makes them good? Like what makes it a good experience for you?**

M: The dentist do the best they can to help improve my teeth.

**I: Mhm, okay. So they, they, care to do a good job would you say then?**

M: Mhm.

**I: And can you Think about when you interact with the dentist or the Doctor...what about that interaction makes it a good experience for you?**

M: [Mumbles]

**I: Want me to be a little bit more specific? Or...go ahead..I’m sorry to interrupt you**

M: That’s okay. I tend to, or sometimes I'll ask the dentist, I want to ask the dentist if there some way to help make my teeth more even–I mean look how crooked and uneven they are

**I: So you ask them if they can help with that you're saying?**

M: Mhm.

**I: How do they respond...what do they say to you?**

M: They told me that they are expensive beyond my family's capabilities/

**I: That's what they tell you?**

M: Mhm.

**I: Okay and how does that make you feel?**

M: Bummed out.

**I: Bummed out?**

M: Mhm.

**I: Okay. But you said that it's usually a good experience–Should I say it in a nice way or?**

M: Yup. They did.

**I: Okay. So what else about interacting with the doctor or the dentist makes it a good experience for you would you say?**

M: Oh! Oh yeah there's some pretty good waiting rooms there!

**I: Waiting rooms are good? What makes a waiting room good? This is good to know.**

M: They're pretty nice seats sometimes there'll be these little tv's on the walls with gaming consoles.

**I: Mhm. So it’s–uh–Fun things to do while you wait.**

M: Yeah

**I: Mhm. Is that both of the dentist and the doctor or just one?**

M: Both.

**I: Both? Okay. So so tell me when you–when you talk to the doctor, is it a he or a she that you see?**

M: He.

**I: He. Okay. When he when he meets you–take me through an appointment–I don't need to know any details about your health–but what is that experience like–So you get there and you check in–What does that first interaction when you check-in feel like?**

M: Oh like oh like–I try to calm down and not get too anxious

**I: Okay so tell me about that–so does going to the doctor make you anxious?**

M: No not really–no not really

**I: Okay, Not really? So not usually anxious or...**

M: Not usually anxious.

**I: Okay. And you mentioned needing to calm down or is there any time that you're not calm about going to the doctor?**

M: Okay yes. Yea yes There were sometimes when an appointment would take would feel like an eternity.

**I: Oh yeah? Like waiting for an appointment or like the actual appointment?**

M: No! Like the actual appointment.

**I: Okay. So what would you say makes them so long? Were they actually long or do they just feel long?**

M: Both.

**I: Okay so tell me more about that... Yeah.**

M: I would say that The doctor would have to tell me to do an eye check test, and then they would ask me to do a hearing test.

**I: So they would kind of add things to the to-do list that you didn't know about initially?**

M: Mhm.

**I: Okay. That's what would make it long?**

M: Mhm.

**I: Okay. So When you do those things, you said they were good experiences overall, how do they treat you, how do they interact or respond to you, that makes it a good experience? Like how do you like to be treated by them that makes it a good experience?**

M: [Contemplates] Oh like They loosen up and relax...And I want my worries to be alleviated.

**I: You want your voice to be alleviated?**

M: Worries!

**I: Worries, excuse me...worries. So they do a good job in kind of, addressing any worries you have?**

M: Yeah. Excuse me.

**I: So, how do they learn about your worries? Do you tell them?**

M: Yeah.

**I: Do they ask you?**

M: Yeah.

**I: Are you more likely, do you tell them instead of them asking you?**

M: Yeah.

**I: Okay and do you feel comfortable sharing about your worries or anything else?**

M: Yeah.

**I: Mhm. What makes you feel comfortable with him–Is it because you know that they're a doctor or is it because how they treat you?**

M: Yeah it's because I know that they are doctors.

**I: Okay so you trust doctors because they know a lot?**

M: Mhm.

**I: Okay. Is there anything about how they treat you that makes you comfortable with him?**

M: Yeah.

**I: Okay. Tell me, tell me A little bit about that. What do they do to make you feel comfortable? You said they have a good waiting room, so that's a good start?**

M: And also oh they would suggest me and [PARENT] some useful advice

**I: Okay. So they give you based on what you tell them, they give you some useful advice about things to do to take care of yourself?**

M: Yep. Absolutely.

**I: And do you always go with [PARENT], or do you usually see the doctor alone?**

M: Always with [PARENT].

**I: Okay. So tell me when you go to the doctor and [PARENT] is there, does the doctor always talk to you directly, or does the doctor talk to [PARENT]?**

M: Me directly.

**I: Okay and what do you say–some other examples that we've heard in the past about what make people's experience good, I'm going to give you some examples and see if this is something they do for you. So they talk to you directly, we know that, do they make good eye contact?**

M: Yup.

**I: And would you say that they treat you with respect?**

M: Yes.

**I: And how do they do that. What do they do that feels respectful?**

M: [long pause] Oh, they tell me whether I've been on a proper diet or not and tell me how my health is.

**I: Mhm. Okay. And when you tell them your worries or anything you want them to know, would you say that they listen to you?**

M: Mhm.

**I: Yeah?**

M: Yep

**I: Okay And do you think that they try to understand and learn more about you?**

M: Yes.

**I: Okay. And also do would you say they also get to know you on a personal level...like things that you like and things that you do besides just your health?**

M: Indeed.

**I: Okay. So like you talk to them about jamming? [laughs] Okay. Um...Another thing that comes up in terms of a good experience is, you know, If you ever have to have something done, like a shot, or some other procedure–have you ever had to get a shot, like your flu shot?**

M: No. I deny the shots.

**I: So you’ve never ever gotten shots, or just recently?**

M: Never.

**I: Never?**

M: [[PARENT] whispers: you did get shots] Oh no, actually yeah. Blood shots.

**I: Blood drawn, or maybe you had some blood taken to be tested?**

M: Yeah, I did have a few shots. Yea I did have some blood taken before.

**I: So how do you feel about that? Are you okay with that? Or does that make you nervous?**

M: Yeah that makes me nervous [chuckles]

**I: Okay. So do they do anything to calm you when you're feeling nervous or tell you what to expect before you go through something like that?**

M: Yeah. They would tell me to calm down, the needle wasn't all that severe, and that they would clean it with a nice wipe, and place a bandaid on it.

**I: Okay. So they kind of told you what to expect, that it wouldn't be too bad, that they would keep it nice and clean, and put on a Band-Aid after? Okay so did that help calm you?**

M: Yeah.

**I: So they said it in a nice way that made you more relaxed and willing to do it?**

M: Yeah.

**I: Okay. And did they ever talk to you about having autism?**

M: Yes.

**I: So tell me about that. What do they talk to you–or do they ask you anything?**

M: No.

**I: Yea or no?**

M: No

**I: No? So if they don't ask you, how does the topic come up?**

M: Oh no. It's more like my [PARENT] would tell them I have autism.

**I: Okay. So [PARENT] would tell them when? Before the appointment, during the appointment?**

M: During!

**I: Okay. And what does [PARENT] say?**

M: I am diagnosed with autism.

**I: Okay. Anything else or just that?**

M: Well, well, she should would also tell them about the side effects of my autism and how I become–excuse me–excuse me yet again–And when I became diagnosed with it

**I: Okay. And when you say she talks about the side effects, What are those side effects?**

M: Oh yeah! That's a good question. Well she would tell me that my autism would cause me to take me longer to certain skills, like how to speak, takes longer than most other people.

**I:Mhm. Okay. Anything else, or is that the main thing?**

M: [long pause]

**I: Take your time, you're not in a hurry, or let me know if I need to say it in a different way, if I'm going too fast, or if I'm not making sense, let me know.**

M: Oh no, you are making sense. I will pretty much act like a loony oddball.

**I: A loony oddball? [laughs]**

M: Yeah.

**I: How’s that? What does that mean? [laughs]**

M: Well it’s [inaudible]

**I: So sometime you panic, am I hearing you right?**

M: Yeah.

**I: Okay, so what–what makes you panic?**

M: [long pause]

**I: And it doesn’t have to be about just anything. Is there anything about the healthcare setting, or the doctor, or dentist that makes you panic?**

M: Yeah–the blood shots. And also, another thing that makes me panic is that sometimes I would doubt myself.

**I: Doubt yourself?**

M: Mhm.

**I: How so? What would you doubt yourself about?**

M: I would feel like I would fail a quiz or an exam or an essay.

**I: So that makes you feel panicked sometimes? Anything else about the healthcare setting–interacting with doctors or nurses–so anything like that? You mentioned the blood draw, the shots–anything else? Or just that?**

M: No, that it.

**I: Okay. So overall good experiences, that’s nice to hear.**

M: Yup.

**I: So just to kind of sum up what you were telling me, so the doctor talks directly to you, makes good eye contact, explains things so that you feel more calm if it’s something that make you panic or anxious, um, and they do know about your, um, being diagnosed with Autism because [PARENT] tells them.**

M: Excuse me.

**I: No problem, no problem. So, so why do you think [PARENT] tells them about your diagnosis?**

M: [Yawns] Yeah that’s a pretty good question. Uh that pretty much due to how, how I had a vaccinated once back in 2001, and my parents didn’t know any better back then, and yeah, and then about at least a decade after that, they would tell me, and really not to take any more shots like that ever again. Or also they would find out about some Autism related events that they would take me there in hopes of [inaudible] doctors and scientists find a proper cure for Autism.

**I: Okay. So [PARENT] wants to kind of tell the history. So, so, um, you think that it’s linked to the vaccine that you had in 2001?**

M: [long pause]

**I: Is that what you, just to make sure I am understanding you correctly, so you’re saying that it was the vaccine in 2001 that caused the Autism?**

M: Yeah.

**I: So [PARENT] wants to let them know so they also can update her on possible cures?**

M: Mhm.

**I: Okay. Do you think [PARENT] at all wants them to know because they might need that information for providing you with good healthcare or no?**

M: Pretty much.

**I: Mhm, okay. So if um If you went to the doctor, and maybe you have two different people, one with autism, one who doesn't have autism, what do you think, how do you think care should be different for someone who has autism? Do you think it should be different?**

M: Yeah, there... Yeah they, yeah there should be therapist for, for autistic people and as well as [inaudible] advice.

**I: Okay. So therapist and advice?**

M: Yeah and, and oh and doctors as well

**I: And doctors, right? Do you think that they need to provide you care any different than anyone else because you are diagnosed with autism?**

M: No.

**I: So do you think that the care should be exactly the same?**

M:Yeah.

**I: Okay. Okay. Do you, when [PARENT] tells them that you were diagnosed with autism, does it seem like the doctor and the dentist and everyone you talk to, do they know what that is?**

M: Yes of course.

**I: Yeah? How did I, how did they show you that they know what it is?**

M: Well they, they explain to me what it is

**I: Okay. They seem like they know what they're talking about? And would you say that they treat you any differently once they learn that about you?**

M: Yes, Indeed.

**I: Yes? How so? How would you say?**

M: I mean, no, they wouldn’t.

**I: No?**

M: No. Oh yeah, they would, they would tell me that, tell me that, tell me that, Autistic or not, you can do achievement. Anything else?

**I: Is that what they tell you? That you can achieve Just like anyone else?**

M: yes indeed

**I: Okay okay. Um, let's see, so is there anything else that the doctor might ask you ? So they don’t ask you any questions once [PARENT] tells them you are diagnosed with Autism?**

M: No

**I: No follow up questions about how you like to be cared for at all?**

M: No.

**I: Okay, okay. But they do a good job because they already know a lot about it you would say?**

M: Yes indeed.

**I: Okay, so one of the things we are working on in this project is doing a better job of documenting disability. So you know like when you go to the doctor and you fill out a patient form , they ask you your name, they ask you your address, they ask you your age, your race, your marital status even–all that stuff. Right. But they don’t ever ask about disability status–usually not. Have you ever been asked that on a paper, or any paperwork at the doctor’s office or the dentist’s office?**

M: No.

**I: Okay. And how do you feel about that? Like would it be okay to ask you that?**

M: It sure would.

**I: Okay, Okay. So that’s one of the things we want to know. But we also want to know if I was going to ask you, how should I ask you? Should I just ask you “do you have a disability?’, or should I ask you...**

M: Yeah you can ask me if I have a disability.

I: Okay. Should I ask you what type of disability do you have?

M: Yup.

**I: Okay. And then what, what would you expect me, as if I’m your doctor, what would you expect me to do with that information?**

M: oh, you could tell me that I shouldn’t let my Autism hold me back.

**I: Okay, I like that, I like that. Anything else that you would want me to do with that information?**

M: That, that, I shouldn’t let anyone else underestimate me.

**I: Okay, okay, should I do anything with that information that would change how I provide care to you? Like is there anything different or extra I should do for you? [long pause] So let me give you an example...**

M: No. Not really.

**I: Not really? Okay, so let me give you an example and see if that is still your answer. So, sometimes people with different types of disabilities, um, have unique needs, uh, for example, some people have limited mobility so they might have trouble walking and use a wheelchair, or a cane, um, sometimes people are really sensitive to light or noise, and so we might do something to change you know, make the light less intense, or noises softer...anything like that that you can think of that I should do differently to make sure that you have the best experience if you were my patient?**

M: Nope.

**I: No?**

M: No.

**I: Okay. So just the same care as everyone else plus those two encouragements of “you can do anything you want”, “nothing holds you back”**

M: Yup.

**I: Okay, okay. So what are your thoughts. So, so, we asked whether you have a disability, we asked what type you have, um, do you think it would be helpful...maybe not for you because you said that your care should be the same–thinking about one of those other people that may have one of those unique needs, like maybe the wheelchair or something like that, do you think it would be helpful if we ask them what challenges they have in terms of whether or not they can walk?**

M: Indeed.

**I: Okay, can you think of any specific questions that we should ask them to kind of get more information about what we could help them with, with regards to their disability?**

M: No.

**I: No? Okay, no problem. Can I show you some examples and get your thoughts?**

M: Yeah.

**I**: **Okay, so these questions here are from the US census that we use to determine how many people live in the US, how many of those people have a disability, alright, so this isn’t meant for the healthcare world, but we can use it as a starting point. So, I’ll go through them and you tell me what the answer is for you maybe–we’ll do it that way.**

M: Alright.

**I: Are you deaf or do you have serious difficulty hearing?**

M: Nope.

**I**: **Are you blind or do you have serious difficulty seeing?**

M: Well I am nearsighted.

**I: As am I–I think I’m near and farsighted these days. Okay, but overall, the answer is no there? You can still see with your glasses?**

M: Yeah.

**I:** **Okay. Umm Because of a physical, mental, or emotional condition, do you have serious difficulty concentrating, remembering or making decisions?**

M: [long pause] Yeah sometimes I get–wish I could remember the things of my old town from my [inaudible] shake-a-leg days.

**I: Oh, Shake-a-leg? Okay, Okay. So that would be a yes to that, not all of that mostly just the remembering? Not the concentrating or decision making.**

M: Okay yeah. Sometimes I struggle to brainstorm with something [inaudible]

**I: Okay. How about–do you have serious difficulty walking or climbing stairs?**

M: Nope.

**I: Okay. Do you have difficulty dressing or bathing yourself?**

M: No!

**I: Okay, and then the last one is, because of physical, mental, or emotional condition, do you have difficulty doing errands alone such as visiting the doctor or shopping?**

M: Um, no. My [PARENT] has been training me to go shopping at [grocery store] alone.

**I: Okay, so you do that stuff all by yourself?**

M: Yeah I’ve been doing that.

**I: Okay, so no to all of them except for this third one about possibly remembering some stuff from the past?**

M: Yep. And also concentrating.

**I: And concentrating a little bit. Thank you. So, okay. How do you feel about those questions?**

M: Um, yeah I’m glad I got those off my chest!

**I: Okay, okay. So the wording is okay, there’s nothing that you would think would be that sounds bad or offensive or anything like that?**

M: Indeed.

**I: Okay, Okay, now let’s say you-you were actually at the doctor and they asked you these questions and you said yes to that one about remembering and concentrating, what should I do with that information, if I’m your doctor? So you tell me that you might have challenges sometimes remembering and concentrating; what can I do to help you with that?**

M: Yeah. You can tell me to deep breathing?

**I: Okay does deep breathing help you with remembering and concentrating?**

M: Yes.

**I: Okay. Anything else I can tell you to do as well?**

M: Well I could-I could use a squeezer on my hands.

**I: Mhm, like a stress ball?**

M: Yep.

**I: Okay Do you like those, ‘cus I have some, I’ll give you one.**

M: Of course I do!

**I: Okay okay, um, so you’re talking about things like deep breathing and squishy balls that to me kind of are used for stress, are you stressed at the doctor?**

M: No, not really.

**I: No you just like those things that helps you with concentrating and remembering.**

M: Indeed I do.

**I: Is there anything else I could do? For example, if I’m getting older and I have difficulty remembering sometimes, I like to write things down. Is that something I should tell you to do to help you remember things by writing things down?**

M: Yes of course, on notepads!

**I: On a notepad okay. What could I do, what else could I do to help you with concentrating? Or should I do, for example, for example, let me give you another idea, umm if I’m not sure that you’re able to concentrate, and I wanna make sure that you know the information I give you, maybe I need you to change your diet, or get some more exercise or something like that, and I want to make sure I know that you’re concentrating, should I ask you to repeat back what I said so you show me that you understood it?**

M: Yea!

**I: Would that be a good idea?**

M: That sure would!

**I:** **Okay. Do your doctors do any of those things? Do they ask you to show them that you understand what they’re saying?**

M: Of course.

**I: They do?**

M: I mean no.

**I: [PARENT]’s shaking her head no. Okay. And do they ask you to write anything down, or do you just because you want to write anything down when they tell you things to do to be healthy?**

M: No

**I: No?**

M: I just remember them.

**I: You just remember them? [laughs]. Okay, okay, so are there any other ideas that you can think of that I should try if I was your doctor to help you with concentrating. We said the ask you to repeat that.**

M: Nope no more.

**I: No that’s it? Okay okay. And [PARENT] you can chime in too if you feel the need.**

F: Yeah. I don’t know–I could add a lot of things. I don’t know if you wanna pause maybe here, I can see that he’s a little–already–maybe like five minute break?

**I: Yeah absolutely, take a rest**

F: You wanna take a break?

M: Yeah. How much longer will this take?

**I: Not, not much longer unless [PARENT] wants to add some of her thoughts.**

M: Okay you can talk if you want

F: Really?

M: Yeah.

F: Okay, well as for the way the interview goes, you are trying to improve the services of the doctors right? So, I haven’t any idea if I am able to interrupt because I think you want his thoughts, but my son basically he doesn’t know how to say bad things about that unfortunately happen right, he sometimes we worry me and my husband that he live in this wonderful world that maybe he is not aware of what is going on, and as beautiful as it is, his innocence sometimes, you know like, we tried to prepare him, but it’s hard, we’re doing the best we can so with the doctors, yes, there’s a lot of need for improvement you know. Like, just remembering him having chronic ear infection, he has a tube placed in ENT doctor which specializes with children, right? Very famous doctor, I’m not gonna say the name, but I–we, not my husband, we went together when my son was about 3 year old, I left that office crying! Just by the way he addressed that was before my son was diagnosed, so we ask young parents, we didn’t know that’s our first child, we didn’t know but the way he addressed–I still remember after all these years how he look at him and address him because my son wouldn’t stay still, didn’t follow instruction, you know, was making sounds, like loud, and just now when I know… actually this doctor with all his rudeness, not being understanding of patient, and just remembering the last thing he say “you should take him [inaudible]”, actually he was one of the person that kind of made me do it faster, sooner, because the whole experience was really like traumatic, like and unpleasant, just thinking of this now. How someone in the medical field dealing with the children, seeing a lot, because right now I can spot the child next to me, I don’t have to know the [PARENT], I can see that the adults, that there is something my God, but that doesn’t mean that that person, you know. We are different but we all beautiful, but how the medical professional can be that unsensitive, I still to this day cannot bare with that.

**I: So that’s still continuing to be your experience with his care?**

M: Yes. Well that was the initial experience with the ENT pediatrician right?

**I: Do you think he–the ENT was like that because, uh, he didn’t know about the diagnosis because he wasn’t diagnosed at that time?**

F: Of course he wasn’t diagnosed, I didn’t know, he just look at the chart, and first he look at us that we are like, maybe we have a spoiled child, but then at the end he said we should take him for some type of evaluation, he was just unpatient, and there is another way to talk to parents, you know? Like–you know

**I: So you don’t think it has–**

F: First of all what I understand now that doesn’t matter if you work with, uh, let’s say neurologists are more likely to meet various disabilities or specific–but what I do believe doctors lack in is this awareness [inaudible] to read–they should prepare them in the medical school as autism is on the rise and other disabilities–developmental, we have high rates of schizophrenia, all kinds of mental problems–I feel like it’s a crisis. So all the medical professionals are able to read some signs and treat them with dignity. The question you ask, that would be ideal if the patient could be addressed, looking in the, many times I try even this interview give my son freedom, thats, I don’t want to speak for him all the time, that’s the best way I can prepare him.

**I: And do doctors respect that?**

F: I don’t feel–for example, doctors visit, st some point when he become teenager we schedule with his pediatrician, we go together yeah, but at some points in the exam I leave the room and they are one on one, doctors yes because I choose the holistic doctor, and that I even worked there at some point for two years in the front desk, so maybe he knows me in the personal [inaudible] its kind of like he ask two, three questions. I usually doctor visit, you know that would be ideal when they get to know you to have doctors but it’s never, they always behind, there’s always rush, they just have to go through certain point, so there’s not really much time unless I guess you have a lot of resources, go to private doctor, pay a lot of money, or have them come to your home, and the public system is not much on the personal level.

**I: So can you say it’s more–if they had more time do you think it would become more personal or do you think it’s just a lack of those skills?**

F: It could be probably both. Now I know that they have developmental charts includes certain question, and obviously it change from when my son was little to now because I was knocking on doors to his pediatrician for long time, “doctor my son doesn’t talk”, like uh it’s two, three years old. I mean he loves his vocabulary, he regress, no [PARENT]my you don’t worry you have three languages, he’s fine, the boys talk–so basically iI would say he delay my diagnosis at least a year. So...

**I:** **And what do you think the cause of that–so he was that lack of knowledge about autism and diagnosis options?**

F: Definitely couldn’t see the sign even though me as a young [PARENT], didn’t know much, but have a feeling, so the only person who kind of pushed me to start searching and asking was his, uhh, daycare teacher because fortunately she was a [PARENT] of a child with little boy with disabilities, so she noticed that my son instead of playing with kids, he’s always moving through some pages by himself under the table and she noticed those things of course lack of language, so she, with the beautiful nice way, she said just something that [inaudible]–so first thing you’re gonna do is you ask the pediatrician, but when the pediatrician just going “no, no he’s fine, what are you talking”–we are a three language family, my husband speaks spanish, I speak polish, and we speak English too, so...we thought yeah maybe try–but still it’s, uh, I’ve been around kids, I see that my son is a little different. So I omit the pediatrician, I just went step ahead, I went to [name of hospital] asking for evaluation they say “yeah, we will do that gladly, we just need referral” so at that point I just demanded from the doctor, regardless of what he thought for that, and that’s where we get this diagnosis.

**I: Do you feel after that diagnosis that interactions with the doctor changes in a good or bad way?**

F: [mumbles] Well, I can write a whole book about that you know, the journey, and I’m really here because I hope that things will change. I’m not on the same page like my son, he’s like saying rainbow and everything is fine.

**I: So is he just being nice, or does he not see it?**

F: He might not see the thing I see, we don’t go to doctors very often, he’s very healthy, we like, he mentioned, I don’t know if you get it, like as soon as I get all my research and facts down, I dedicate conscious hours of researching and observing and talking to other parents, we stop vaccinating when my second daughter is vaccine free, and because that’s actually we do believe that’s why I inform him, because sometimes they bring the buzz here, everybody flu shot, we do inform our kids, do not ever take any flu shot, do not ever refuse those things, they know it’s bad, it’s toxic, I observe as a [PARENT] even though the DCF says that’s not the cause of it, I’m a [PARENT], I know what happened to my child, I know that the regression, I seen it on my eyes what happened after a series of shots, and that he received in one day from being able to speak ten word, cus he was [inaudible], it was after that when he developed terrible fever, we had to run to [hospital], like almost seizure like, so...when we wanna talk about changing approach for person disability maybe it’s not your study, I wish–we have to go to the roots first, have the doctors be real doctors, not I walk away from medical field because I see so much corruption, so much just for money, for profit, the more drugs, the more vaccines, the more bonus, if I’m the doctor I’m doing all the papers, I know what I’m talking about, I’m not making that up! We host the pharmacuetical lunches with the doctor just being busy with his stuff, not even listening about the new drugs for ADHD or Autism, I’m a secretary, I was asking question, the doctor not really–he just ask where he sign. I witnessed it. So, maybe you know, an idea were there somebody else doing a study on this are the doctors they really doing more help than harm, you know? Let’s stop that. Let’s stop injuring our kids. These vaccines injuring, I don’t know about other developmental problems because they vaccine can cause, there should be information given to parents who could all avoid, not everybody’s curious because I save my second child from being injured–let’s call it how it is, but I’m doing the best I can now, it’s basically helping him throughout his life, the therapies and this, and this, but in the ideal world before we get to services how they treat our kids, our adult kids, teenager with development–let’s make all the doctors properly inform the young parents, now they don’t even offer you the vaccine insert–they don’t even know what’s inside the vaccine–they’re not informed because in medical school they just memorize the schedule of the vaccine, nobody even question what is inside the, why what are the side effects, you know. So that’s where I would like to–the–the basic where it is what I see for 20 years dealing with the medical field, addressing because of the lack of time and the medical professions, I direct them to my son, because, you know how he is–why I told you to take a break, he yawns although he sleep like 10 hours, he takes his time, you know so the doctor prefer, you know–”let me ask [PARENT]”

**I: So you think that they’re going to you not because they don’t think he understands but because they’re trying to get you out faster?**

F: Faster.

**I: So they’re not necessarily making an assumption about him and his capabilities?**

F: But that also too because it is an assumption because my son–he might, you know, he have difficulty, his hands sometimes, or eyes, you know, he appears like he’s totally lost sometimes. Or stare, or ask random questions. When you, if I send you the first essay on his ENC English II, when you read, I keep asking “Please, did you take it from–where did you get it?”. What he’s capable of, what he’s writing essay about time, how he understands, I was really impressed. I say “No mama, [inaudible]”, and I do believe that even for me as a [PARENT], it was like–like I have to be reminded, and I’m impressed, and of course somebody who doesn’t know my son, they just keep, and that’s one of the fact that we need to change in the medical profession.

**I: So they don’t–um–like ask any follow up questions of him about, you know, knowing that he has that diagnosis, do they ask any follow ups about how they should provide care any differently or anything that they need to keep in mind?**

F: No!

**I: And are there things that you share with the doctor about things they should know, like he mentioned that you mention he was diagnosed with autism, he mentioned there are these side effects?**

F: The most, um, what we can talk about, his pediatician we see once a year for a check up, right? So it’s a routine question about the diet, then how he’s doing in school , all of that is fine, then the physical exam, and like I mentioned the doctor kind of knows me so he do little talk, so he would tell him about a certain age let’s say when he reached 17, he was talking about, you know, because it’s a routine, no smoking, no drugs, you now, so just do a reminder, so I think they doing–if I feel like, for example, I have issue about sexuality, right, like–any time I told my husband ‘It’s time, you have to talk to him” since he was like 14, 15, about masturbation, about teens, right? Like–I don’t want to talk about my son, so I’m like maybe I need help to reach out to–maybe that’s the last thing he wanna talk with the parent–I’m very open, I say “You can talk to me about anything, there’s no subject”–sometimes maybe I don’t wanna hear things that you’ve done or wanna do, but believe me even if I upset, I wanna know, and I be happy if you share with me, you know. My [PARENT] wasn’t like that, so you know, I search [inaudible] and it didn’t turn out much beneficial right? But he say “Okay”, so he shares some, but I know he be very reserved.

**I: So you think he’s more likely to share with the doctor when you are not in the room?**

F: Yes. Yes. He been under care of like, uh, it’s a mental health department because I seek out, like in the past, more than a year ago, like, somebody like a counselor, like ideally it would be like male figure, like so he’s going, he took a little break because they change profession but he used to see someone like for an hour, like a counselor, just to talk, they ask him how you college, how to deal–that’s why he knows to breath, to stress ball, you know, without any medications, you know. Like just how to…So this been I’ve been very happy about those services, like those are young professional , I think they are they only work with people with no insurance, because we didn’t have insurance back then, so that’s how they start, and they kind of, like I guess psychology students during their last year or something, some kind of, so they relatively young, they have a lot of enthusiasm, I see only positive things with them. Like they just, there for ***him***, right. But I guess your research is more of the medical stuff, the dentist, we’re not there yet we’re not there, I’m not sure about that. The clinic that they created specific for adults with autism, there’s something like that by [name of hospital]–we never went there, because even though my son is 20, his pediatrician will see him until 22. So, like we don’t wanna change before then. Probably when we go to the clinic when the profession see just the adults–it’s all different right? But it shouldn’t be like that.

**I:** **Mhm. So overall you would say that they don’t have enough time to do a good personal interaction, but even if they did they might still not have that, and they don’t know enough about Autism? Is that a good summary?**

F: Yes.

**I: Okay. And what are your thoughts–I mean you obviously heard me read these questions–would you like me to read them again? I don’t know if you were paying attention at that time.**

F: If I understand correctly, you would like to use those kind of questions for you records?

**I: Well it’s one–the question is should we be asking about disability? And if so...**

F: Yeah, maybe it should be like let’s say like in college campus there’s access center where he received the letter through hand it to professor in the beginning, where the disability is not stated, but by receiving this letter, the professor knows that he has some type of disability, doesn’t matter what, and by law they not supposed to put what is it, although [son] is very open, he talks about it, he writes and he shares, but maybe some people don’t wanna share it, so maybe the option–my idea, I’m not sure if it’s correct, maybe option do you wanna share if you have a disability? An option. If you wanna put it, because some people might not wanna share that they going through mental problems, or I just don’t know.

**I: So of having an option for–**

F: Option–yeah!

**I: So I decline to answer the question, or something like that.**

F: Mhm, yes.

**I: So let’s talk about the people that would answer the question. What do you think that they would want to share, and you can think about specifically for [son] or just in general, what should the doctor know? Should they know the type of disability or know more than that?**

F: Especially with Autism, I would say that it is a huge umbrella, there are kids, adults that are non-verbal, there are..my son is considered high functioning, he is able to study college, I guess like several question that without being offensive, help the healthcare provider what level of disability or function he has. What I understand we have not gone through the procedure, but I know when you reach 28, 21 you go with your parents through this process that if you not able to function, I guess you having your power of attorney turn to parents, and they are the one making decision as far as healthcare and anything everything had to be done with them. So I’m at this point that you know obviously my son is stable, I don’t wanna strip my son from any rights, voting, he was voting last election, and I encourage him to be curious, and as far as medical decision, I would consider with the lawyer if, what kind of like, I don’t wanna also strip him with that, but if there is a place that the parent can have some kind of input, like I haven’t gone through this process yet, so I just wanna find out. So maybe those questionnaires would be something that help to determine the level of functioning and the way to figure it out is to just, you know, ask the–

**I: You think it’s better to just ask and have a conversation versus having something on paper that needs to be–**

F: The general should be–the general question if you have any disability, or you wanna share with what type optional, yes. The general information I think it should be–

**I: And that’s kind of like a starting point for the conversation?**

F: Yes. And then there’s–yes exactly. And then you have your medical record, maybe it follows you so the dentist know, it’s some kind of alerts so maybe that will give like oh I can be patient more, give you extra time, or use when you talking to this type of patient you can use, um, you know maybe some kinds of, um, how do you call this, assisting...

**I: Assistive technologies?**

F: Yes. Assistive technology because even though I know personally adults teenagers that are nonverbal, they can really communicate very well and using this, usually with the help of parents, because I know two cases. I know one case that is the boy is 20 something nonverbal at all, and the [PARENT] is just, you know, go and get it, anything possible, she will get it and try, and, I know another person that is a girl that is probably not 18 yet, maybe 17, the [PARENT] just have her, it’s my neighbors, so in the beginning I tried to help give her resource, or go, they giving iPads for kids who are nonverbal, I tried to encourage her to do stuff, but I see she gave up, I don’t know the reason she have her just at home, and the girl is injuring herself, biting herself, nonverbal, just...and...nothing is done for her, no therapies. It’s like breaking my heart because with all this technology available, it’s–that’s why I think that the doctors should be informed because we have different type of parents, we have parents who either don’t know, or are lazy, or just let it be, let’s send them to the institution right? And we have parents, I consider to be one of these parent, that I find out the resources, my son is always doing something, and always pushing, always, you know have this attitude, you can do it, and yes, like he mentioned, participating in all kinds of events, organizing autism thing, walk, surfing, everything. And even though college campus just umm–spreading awareness because as much as we talk about that, it’s still a big lack in even in access center and the professors too. We do. So that’s idea for the doctors be able to to have more information like a general training that, to be able to recognize the signals in the young childrens, and then once they are able to recognize certain symptoms to be sensitive, know the-have a list of resources that could be given to the parent, then once they deal already with the person, address them as much as they can address them really, you know, because of this lack of knowledge probably that they have when they look at my son, they just address me, right? Because they see my son maybe, like, you know maybe staring at the sky or something, let’s say at the dentists, so they just go straight ahead right. And not necessary now at this point I go and first thing I say is my son has–no not really. I go with the flow, it should be able to determine right? And it used to be when he was younger as a boy to kind of like–uh–give them more patient like help them, how can me as a parent be of service, maybe he doesn't like to be touched in the head, blood works, maybe they add initial help, or sing a song, or like, you know, create sticker, or distraction, or like right now

**I: None of that’s happening..**

F: I don’t use this We walk in to his office and he has autism-treat my son differently. I actually want him to be, you know. I believe that all of the healthcare professionals regardless of disability-they should be caring, respectful–and yes. If they should treat my son any different? No. He should be treated the same unless he requires certain accommodation, or then yes otherwise, no, please treat him the same way. And that’s all I guess.

**I: Okay. So I mean yea those are all my questions. I know we’re also I want to be respectful of your time so I’m gonna turn this off right there–**
