# Supplementary material for: Disability disclosure in healthcare settings for individuals with developmental disabilities: A qualitative study of patient and caregiver perspectives
Source: PLoS One. 2025 Aug 7;20(8):e0329328. doi: 10.1371/journal.pone.0329328 (PMC12331114; doi:10.1371/journal.pone.0329328)
Supplement: S1 File — (ZIP) [file pone.0329328.s001.zip › Transcripts/2019.09.13 Interview 10 Transcript.docx]

I: Interviewer P: Participant

**I: And just for the record umm we’ve gone through the informed consent and you’ve agreed to participate to be recorded?**

P: I have and this is [Participant Name].

**I: Thank you. Alright. So, you know, in talking about healthcare experiences we kind of want to get a sense of what experiences have been good, if any, what experiences have been bad, if any. So, I’ll start off asking have you had any bad experience in one or more healthcare settings?**

P: I don’t know if I would necessarily describe it as bad, like it wasn’t torturous or didn’t, didn’t scar me umm irreparably. BUT, I will say that looking back at things now umm as a 45 year old when you look back at things that happened to you when you were younger, and especially now I kind of work a little bit in the healthcare arena but I do more advocacy stuff so I’m kind of a little bit more familiar about how things SHOULD be rather than how they actually are. One of the things that I remember very early on umm I’m a twin like I told you before, and umm a very umm interesting experience happened to us. When throughout your early childhood when you’re diagnosed with cerebral palsy, which is what my disability is, umm you have a series of doctors’ visits for various things. And umm I remember going to a number of doctor’s visits both here in Miami like early, like maybe nine, ten, eleven years old, and even before in Jamaica and we actually lived in Cuba as well. We lived there for three years umm right after diagnosis. And I remember always feeling like we were not a part of the conversation. That things were just told. Explanations were always given to my mother who was our primary care because my parents umm divorced when we were what? About five? Ummm that’s another disability; that’s another outgrowth of disability, I believe, although our father will tell you that that wasn’t the reason, but looking back now it’s pretty clear. Umm and I remember I remember always the doctors, like I said, explaining stuff to my mother umm telling her, for instance, we had to have abductor and hamstring surgery. And umm just explaining to her all of those processes, but no one save one doctor and I’ll mention him and it actually was at [hospital]. Umm his name was Dr. [doctor name]. Umm ever no one ever had the conversation on a level that young children could understand. That that said, we were not, if I do say so myself, unsophisticated young children, because we’ve been exposed to a lot. So, we naturally had questions. But those questions were never addressed in a way that would make us less anxious, make us less concerned. As a matter of fact, it created a whole lot of umm talk after. And I look back at it now on what it must have been like for my mother because she kind of had to play this intermediary role because she was a this parent getting all this information of all this stuff that was new to her and when she had us she was in her mid-20’s, 24, 25, and we were diagnosed at like about a year and a half. So, she was from from the point of diagnosis to what I call the point of stabilization which is when we go to school and start doing stuff, it was like I would imagine going to disability university for her. Because nobody, especially circa 1970 something into the 80’s, we were not at a time where we are right now where, you know, you have the [doctor’s name] and you have all these people who are experts at laying it out and, you know, I I remember when I taught the LEND program, one of the one of the module’s we had was on delivering uh diagnostic news. [Name] taught that that module and umm, you know, for her, I was I was not old enough to remember what it was like when the news was delivered, but I- I knew that there was a lot of sadness associated with the disability and it it’s interesting because yesterday we were at a bioethics thing and there was a parent there that was a part of the panel. And one of the things I thought and one of the questions I asked is, “How much has a teen kind of worked with this parent to not make the disability the most devastating thing possible?” Because I reflect on my time and for my mother and father, particularly from my father, you find out in your twenties that you’re going to have twins and the babies are here and they’re seemingly normal and they’re- and they’re twin boys and you’re a dad and you’re in your twenties. So, you feel pretty good, right? And then all of a sudden, the disability thing raises it’s head. You knew that there were complications at first because we were born prematurely, but I mean there’s not a lot of education about what that meant. And and we developed pretty typically throughout our throughout our early development. My mother says we talked and did everything, very surprisingly I talked early. But we did everything early, it was just when it was time to walk and sit up and do those things, developmentally we weren’t there. And she was told any number of things from we’d be severely disabled never to be educatable, to you might as well just write them off, to uhhhh everything in between. And I imagine what that might have been like for a twenty-something year old couple to deal with that. And my mother umm had my sister when she was relatively young, she was 17. So, she had not only these two babies, but she had an older child. So it was just a lot going on. And and uhh my father was my mother’s second marriage. So she was a rebellious child and she married her high school sweetheart and it was a whole thing. But then they got divorced and then she met my father and then ultimately we were produced. I say all that to say: in your twenties, that’s a lot going on. And add against that the backdrop of being in a country that’s economically disadvantaged. We were umm middle-class, upper middle class whatever you call. (phone rings) See this face?

**I: (laughs) Oh yea. You need to take that?**

P: Let me just tell him that..

**I: Ok.**

P: (puts phone down) It’s weird. I tell my brother all the time I love him. He freaks out about that. (laughs)

**I: (laughs)**

P: I’m like yea! You’re you’re like my, you know, my second my half so I have to. If I don’t love you, I don’t love myself. Anyway. So add all that again to the backdrop of the economic picture in Jamaica for the unknowns. Right? We were umm middle class umm but we had no resources about knowing any of the stuff about disabilities. So it was a very uncertain time I would imagine for my parents but back to the question about me. All I remember especially when we came here because we had been in Jamaica and Jamaica had very limited resources so my mother being- I’m giving you all this background to you so you understand kind of the table- my mother had decided that there were no resources in Jamaica so she’s going to have to find a way to fix her children. So she uh got a job in the Jamaican embassy in Cuba because at that time working in the U.S. was not even- it was not what she wanted to do, it just kind of happened. But but she was very active politically and everybody knew, she knew all these elected officials and everybody knew that these children had issues. So she got herself a job in the embassy as an attaché. My mother spoke no Spanish. But she was just determined that this- she had heard good things about socialized medicine and this was kind of what we’re going to do. So uhh she said to my father- they weren’t divorced-, she said to him “please come” and whatever. He wasn’t really interested. So uhh she got the job in Cuba, we lived there for three years, and every day while she went to work, my aunt who was one of- my mother has six sisters and a brother and we’re a very close Jamaican family so her thing was okay we’re going to fix the children so my aunt went with her. So while my mother worked, my aunt would go to therapy or be at home and whatever. I must say one of the best things in my life is that that particular act, they were worked outside the home. Because she was a child of the fifties and for her the whole model was find a husband, get domesticated, get married. So I tell you all that to say after we moved back to Jamaica when her assignment ended, this was mid- early eighties, parents got divorced. Then we emigrated here in the ’84, three years later four years later. And when we came, it was like we were exposed to this whole buffet of all this stuff related to fixing the children. So it was biofeedback therapy, it was occupational therapy, it was physical therapy, it was all of that every day. And my mother got blessed and she would work pretty much all day and by this time my grandfather had come into the picture, so he was the one that took us to school and went to after school activities and did all that stuff. So what that did was umm during the whole exposure to all of the stuff, biofeedback therapy I don’t know if you’re familiar with it?

**I: I’m- I’ve heard of it yea.**

P: Dr. [doctor’s name] was one of the first people in the country to do biofeedback therapy. And this is how I know my mother was hard core. She knew nothing about it. She wrote him a letter literally a hand-written letter and mailed it to him and then we were accepted into the program. She didn’t know whether it would succeed, fail, whatever, she just knew that she had seen something. I think she saw it on channel 10 or read it in the Herald and it was…so I I tell you all that to say we had a lot of exposure to a lot of stuff, a kind of buffet of medical settings, but I will tell you very early on and I would say up until about 1987, and I would have been about 13 at the time, everything was done to us and for us, but never including us. Even though we were always present at the doctor’s appointments. We always- my mother always encouraged us to interact with the doctors. It was almost as if you had to prove you were smart enough to be in the room in a way. Nobody ever said that, but it was like, you know, you’re kind on display so you’re kind of, you know. And and and looking back at it I, I realize that it was a fair amount of pressure with that. You know? And my brother my brother is the- we did a umm he’s strong certified which is the career umm axis testing of personality types- and in every way every axis, we’re completely different. I’m an extrovert, he’s an introvert. Umm I am very I’m very spur of the moment, he’s very analytical. It’s crazy. But what I noticed during that process is, where as I would pipe up, my brother would pipe up but he wouldn’t pipe up quite as much. But he’d be kind of urged on by me so by virtue of me speaking, it would be- it would give him the impetus to want to speak as well. So in those settings like I said, we were always just the object to be moved around.

**I: And do you see that as a reflection of them seeing you as as minors or or do you think it had anything to do with having a disability?**

P: It was all of it. I don’t think it was one thing or the other. I think there was not a lot of emotional intelligence about what it means to interact with a person with disabilities. Umm you have to remember basically on the timeline of disability civil rights and it all- I know it sounds like I’m thinking myself so important in the timeline but- have to think about this way, the view of disability and what that meant even in the medical community was completely different to what it is in 2019. Right? So this whole idea of nothing about us without us, this whole idea of me being self-actionized and being myself and speaking up for myself, that was something that just did not exist. So it was that and it was also children are seen and not heard. I mean that was, you know. And when I think about those things and I know have uh young nephews, I can’t imagine ever being like that to a to a little- because I consider children like little adults. You don’t have to burden them with all the details of everything but they’re capable of getting so much and if you are a role model and if you’re a parent then you know what bits of information to give. So...so did I answer your question?

**I: Most certainly. No yea, so yea, I mean so you definitely talked about the fixing us mentality and the the just kind of you to us and for us but not you know with us.**

P: I wanted to say one thing. I mentioned Dr. [doctor’s name]. This is a function of my mother again being the awesome dynamic. I I really think what she did was phenomenal because she had not one, but two. But not only that, she just taught us to really speak up but when we were thirteen, we had to have hamstring surgery. Both of us. So we’re both in full-body casts, right? And so Dr. [doctor’s name] who worked at [hospital] for years in pediatrics and they had us (inaudible). He died a few years ago. I looked him up the other day because I was curious about what- because I would’ve like to see him. I think he died in 2009. Umm he- when we went pre-surgery, he explained everything not only to her but to us. And though our physical therapist was in the room because it was part of a whole team approach, before team approaches were like the-

**I: thing.**

P: thing. He made sure that we understood everything. He made us understand that there were risks associated with the surgery, he made us understand that he would- I was I was concerned like why do I need to have this surgery if I’m not going to walk because that’s the other thing. And he said well, you know, it has to do with your spasticity and your muscles being very tight and you want to give yourself the best. And he explained it in a really understandable way to a thirteen-year-old and that made the whole surgery process so much better cause we were in pretty much a body cast all the way up to our waist. Because he had done the the releases but then you had to stay pretty much rigid and we were out of school for like three weeks or whatever because we couldn’t go to school. But the fact that he explained it and the fact that we were doing it together made it more digestible. The other person that was really good at it- umm including us in the conversation- was Dr. [doctor’s name]. [Doctor’s name] was in addition to doing biofeedback, he was a psychologist. And my brother had a lot of issues of depression heading into early adolescence. Because I was the more extroverted twin; he was the more introverted twin. But beyond that, he has a lot more physical limitations than I do. And in your adolescence early adolescence is where you start to branch off. We were twins so everything we did together. But I was Student Government nerd and I was mister wanting to do this and do that, and my brother was always kinda not that person. And not that it was my fault but it was just the dynamics of twinning. But one of the great things that happened from us getting to know [doctor] is he saw the sadness in my brother. And he had several sessions and to this day my brother and I talk about everything. Till this day my brother won’t tell me all the details of their conversations. And this is like thirty years ago. But whatever he said to him, whatever they discussed in those sessions, made him realize that, you know, it wasn’t all sadness, it was going to be, you know, a challenge, but it wasn’t going to be the end of end of life as you know it. You’re going to have a life in other words. And and I mentioned that in the context of, again nobody had really had an in-depth conversation with us. There was always an acknowledgment when we were in the room “Hello young man, how are you?” that kind of thing. But never “how do you feel? What are you thinking?” all of those things. So, my mother kind of had to play the intermediary role. Like we discussed stuff before and then we’d go and have the doctor’s appointment. We- they wouldn’t really talk to us and then she would on the way driving home, she would kinda explain everything. And I remember us having even some fights in the car because, you know, her mode at the time was fix the children. So everything was fix the children and and we just got frustrated with that. And also and I feel horrible because for her at that time, I mean she didn’t know anything different and she was not she was not trying to exclude us either from the conversation but that’s how we felt.

**I: Mhm, okay. So what other things can you umm think of when you reflect back and it could be more contemporary rather than, you know, when you’re in your childhood.**

P: Oh okay.

**I: I mean all of it is game, but, you know, you said it’s different now. So what-how how is it different now?**

P: It’s different now because a) I have been around a lot of doctors. My sister now is a doctor. My brother-in-law that just died last year was a doctor. Umm so going to the doctor and and and functioning in that environment, I don’t think doctors realize how intimidating they are. And especially for young children because typically when we went to the doctor it was always about “oh we have to have surgery” or “we have to do this or do that” so it was always a little intimidating. Now, as an adult, I am fully- I fully own my experiences. In the presence of doctors, I know exactly what I want to say. As a matter of fact, in my family I’m kind of the designated go-to-the-doctor-with-person. Because as my as my mother and her generation have aged, I’ve always encouraged everybody to kind of go in teams. Particularly when it’s like big news. Like umm my mother had a stent put in. And so I- one of the things that I always do is go with her to the cardiologist. So, if I’m working, I’ll take the afternoon off or whatever just simply because I always think that you understand more in teams and then you can discuss it after and decompress. And when they’re talking they’re they’re just giving you information. They’re not really, most of the time, really making sure that you understand all the implications. So, if you have it in teams and you have somebody that you trust, I always recommend it. But umm so I totally own the experience that- I have my questions. Dr. [doctor’s name] was my mother’s cardiologist. Whenever I don’t go with her, he always asks where’s [participant] today because he’s so accustomed to me taking out my iphone and going okay I want to know this this this this this, and what medication can we do for, you know. So it it’s a completely different experience and I own it as a result of a) age, but b) realizing that ultimately I’m the one in charge of whatever is going to happen. It’s not it’s kind of the reverse of the feeling that everything was done to you. Taking on a shift makes you say “okay this may be done with me but it won’t be done to me.” Does that make sense?

**I: Yea yea.**

P: Okay

**I: So so so tell me so when you when you first make a connection with a healthcare provider, think about when you when you first, you know, went to an appointment for the first time with a given healthcare provider, I mean do you do you go out of your way ahead of time to let them know about your disability? Or how does that conversation come up? What is discussed?**

P: It kind of depends.

**I: Okay.**

P: I’ll tell you a five second, maybe a minute story. In my previous life, right out of college I was a reporter. I worked for the Sun Sentinel. My job initially out of college was doing what we call editorial assistant. What that meant was I called- every morning I’d go in to get all the faxes- faxes existed back then. Then we’d call the police department and we’d do checks. We’d say what’s the follow-up on this story that they faxed us about or anything good going on or etcetera etcetera etcetera. So and from that evolved the reporting thing which was ultimately what I wanted to do. But at 22 you did get whatever- you take whatever job you get. So I was on the phone with this police officer one day and I was getting all the information and then they sent me out on a story. So I went and I went to the press conference and I raised my hand and all that stuff and I asked a question. And and I saw this weird look on the police officer’s face like I recognize that voice. So I went up after and I introduced myself because this is this is the function of of newspaper journalism. You don’t always meet the people that you’re talk- you know, you could report with people for a long time, never meet them. And he was totally in shock when I introduced myself. And the wheelchair and the disability and all of it and what was really cool about it was it was horrible, but it was cool. He was so unsophisticated about like being totally like blown away that this person that he had known for six months on the phone that he thought was this able-bodied person was this dude in a wheelchair. So I tell you all that to say, sometimes I don’t always introduce my disability because I kind of feel like particularly in the medical realm, they should be okay enough to be alright with the fact that I use a wheelchair. If I go and the office is not accessible, then that’s a different conversation. And I’ve actually had people say to me, you know, we’re at a family doctor that we used to go to down the street who when without powered wheelchairs was always very like oh my god it’s so inconvenient like all of this stuff, how are you going to weigh all this stuff. And then I ultimately stopped going to her when I was an adult because I said if her practice is to do medicine and I’m her patient, I’m not saying she needs to fix it right away, but if there’s no plan to fix it, then my money and my healthcare is not important to her. So...

**I: Right.**

P: Did I answer your question?

**I: Mhm yea yea. I want to delve a little bit more into that. So so.. so you said sometime it depends on the context**

P: It depends on the context

**I: as far as what you want to share, but**

P: And also what I’m going to the doctor for

**I: Sure.**

P: Like I have a hematologist

**I: Mhm.**

P: that I go to that’s part of [healthcare system] now. And I go to him because I had a pulmonary embolism about seven years ago. Umm didn’t know that it was coming, it was kind of out of nowhere. It was one New Year's Eve, I think New Year’s Eve 2007. I just felt very ill. Didn’t know what was wrong. And umm I had been working really hard umm but I just felt very winded, very tired. Um, ironically, everybody except for my one aunt had gone to Jamaica for that Christmas and New Year period. And I remember talking to my brother because my brother and I talk every day like teenage girls when we’re  when we’re not around each other. And usually we’re like kind of the last people that talk to each other. And and I said I feel like crap. So ultimately I called that doctor and umm I said I don’t feel good. And she said well you know it's the holiday, the emergency room is packed right now, maybe you just need to get some rest which was absolutely the wrong thing to tell me. This is one of the other reasons why I no longer see her. And ultimately I I called 911 and they came and they said, they told me oh nothing just, you know, you’re not having a heart attack, my heart was racing. And I said okay and my aunt stayed in bed with me which is very unusual because she’s also not a very touchy feely person. And she said-  they left- and she said “Chew this aspirin” . And I got up, got dressed and I was supposed to go to work and I called in sick and I said I’m going to drive myself to the hospital because I just didn’t intuitively feel right. Ultimately it was diagnosed that it was a clot that had not only traveled from my leg, it was in my lung and it shut down the lung and it was on its way elsewhere, so I’m very lucky. So I say all that to say that it was pretty much stable for years and years, but a year ago I noticed swelling in my leg and it freaked me out again. I was like oh my god another clot. So I started seeing Dr. [doctor’s name] at [healthcare system]. It’s not far from here it’s not downtown it’s like the Kendal. And and so I had to kind of announce my disability beforehand because it was directly relevant that I was that I- why I was coming to them because of the swelling of my leg because I had a sedentary lifestyle. So in those cases, I definitely announce it.

**I: Sure.**

P: That make sense?

**I: Yea yea. So so I guess the question is, you know, in those contexts where you feel like it’s it’s relevant and you want to share ummm how how do you share or or, you know, what information do you think they need to know specifically.**

P: Well ummm I find it funny, okay, because on a lot of the intake forms they also give  medication and they ask you a whole bunch of stuff. And particularly for those that I do not disclose it to in advance, I fill out the forms and they and I I there’s always this kind of awkward experience where you go in to- before you see the doctor you see the practitioner who takes your blood pressure and does all that and goes over your chart. And then for your given, and there’s always this moment where they want to say so what’s the deal with the chair. And some do it more elegantly than others. Others just say okay you have all of these things and they never address the chair and they leave that to the doctor which is kind of catcher’s catch can. Umm but if I see awkwardness, I’m somebody that just throws it out there and I go oh by the way, I know you’re wondering, so I use the wheelchair because I have cerebral palsy. And, but uh my Cerebral Palsy has not limited me from the duh duh duhuhuhuhuh. Because sometimes it’s easier to, if I’m not in a sadistic mood (laughs) just lay it out there, but sometimes it’s good to watch them squirm a little bit. Because honestly, I find it fascinating that a lot of these folks are trained to deal with people across the healthcare spectrum and yet they’re uncomfortable talking about the big ass elephant in the room. That’s amazing to me.

**I: Is it because while they’re trained across a lot of different aspects, they lack this training? Or what do you see it as being?**

P: I think they lack the training. I also think that, you know, it’s a product of integration, right. I remember when I when I worked in the newsroom, my internship in college, senior year UM. I worked at channel 10. I complained, unlike all the other interns that were interested in TV and wanted to go out with reporters, a) I drove but I always knew it would be an obstacle to go out with the reporters to do whatever, so I was more interested in the nerdy writing part of it. And so, I someone saw that I was kind of like a uh associate producer doing my internship that they actually offered me a job before the internship ended. I ended up not taking it because it would’ve been overnight and then I would’ve dropped out of college and then my mother would have killed me cause then you know... So, whatever, we’re letting that go. But I’m writing and the producer says to me, get a reporter's notebook and I want you to do this interview. I go to get the reporter’s notebook and it is in the cabin way high, higher than I could reach. I come back, I sit down and I’m writing on a little scrap of paper. [Producer’s name] who’s the producer says to me why didn’t you get the notebook? I said I couldn’t reach the notebooks. Som he said, “fuck that’s horrible.” So, he went and he took the notebooks down and he and he put them where I could reach and he sent out a little memo from now on we’re supposed to keep it where everybody could reach cause we want to be accessible. I tell you that story to tell you- to illustrate a larger point which is once you’re exposed to it, it’s not a disability issue anymore. It is an [Participant] issue. You like [Participant], [Participant] is a part of your environment, you want to make sure that he feels included not excluded. So, it’s not a weird thing. Likewise, umm with the doctors, once they get to know me, then all of a sudden, I’m not this clinical person in a wheelchair, I’m a patient of theirs who, you know, aspires to do this, or does that or whatever and it becomes real. So, a lot of times, what happens is, I suspect that a) a lot of these doctors have not been exposed to people with disabilities in integrated settings which means classrooms, school dances, all of the above, right, cause that doesn’t really happen typically. It’s happening more and more now but it didn’t as I grew up. So that changes. Because once you get to know somebody then it’s not the disability anymore, it’s a me thing.

**I: Right.**

P: Does that make sense?

**I: Mhm.**

P: And so and it’s interesting cause my brother-in-law who passed away last year was umm very educated, he was a Rhodes scholar and very involved in academia as well as medicine. So he was a PhD and MD. But I’ve I asked him repeatedly, particularly when I started working at [center], I said what were you guys taught in medical school? And I mean he did medical school not only in Jamaica, but he did it in a [college] in Oxford and he did and he did different- he did his residency somewhere else. And my uncle who was a resident under [doctor] who now works in umm Nashville, I said “[uncle] what did you do? What did you learn about disability?” And the overwhelming answer is “not a lot”. They learned about the diagnosis of, they learned about the clinical piece of, so they could recognize CP or they could recognize autism or they could- but not the whole picture. Piece of it. Which is fascinating to me that I that I’m a little part of trying to make that more real because that is that is a piece that has been missing for such a long time. So.

**I: So, so do you think umm their knowledge of you having a disability negatively impacts the healthcare that you receive in any way?**

P: Um, no, one thing I will say is that for a long time I didn’t weigh, it's uh it’s one of the questions on every on every medical intake form, I weigh it, it's one of the demographic questions for a long time I didn’t weigh. When I was in DC I still had the same issues I have now which are I have upper GI issues as a result of the blood thinners, what the blood thinners did that happened with the PE is I had a couple of small esophageal ulcers which the blood thinners caused to perforate, and so I I take umm reflux medication instead of - But I remember going to Dr.[Doctor’s Name] in uh at [Institution] and I remember them asking me what’s your weight and I deliberately said “gee I don’t know, where can I go weigh? I walked I rolled the entire [institution] campus trying to find an accessible place to weigh. Fast forward to a “year ago I mentioned this to Dr.[Doctor’s name] who is now my um hematologist and he says :of course there is a way for us for you to get weighed, just go in that room and then go on the chair scale and then whatever,” and I was so excited I swear I hugged him, I’m like do you know how many years it’s been since I, I mean I had an idea of my weight but I mean but it’s such a fundamental thing and to just realize that you have to go through all that just to get what you need is amazing. So, but, eh and and he said you know cause we had a discussion because I actually took a picture on the scale and Shelly and I do this thing with medical students every beginning of like March where where we’ll we’ll talk about health disparities and all this stuff and there’s a there I took a picture they included it in the powerpoint just to show that this has been a challenge cause I tell the story and and and it's amazing to them that there’s this and they’re so fundamental that people just don't think about because again it's not experiences that they have, you know?

**I: Right, right. So umm would you say that umm you know we talked about if them knowing you have a disability impacts your health in anyway. So umm, have you encountered any any negative experiences with regards to like assumptions being made about you? or?**

P: Always, Always, Always

**I : So tell me more about that**

P: a) there there always assumptions a) that you, let’s put it this way, because of lack of exposure to peoples’ disabilities the assumptions are always that you are this clinical being that functions that is literally confined to a wheelchair that’s why when I read stories about people who are confined to wheelchairs I always write the reporter and I said you know there’s a better way to say this and style book and all that stuff but the reason why I say that is with that assumption of confinement what it means is this idea that you are no more than sum of your parts, than a wheelchair, so you don’t have a social life, so you don’t have a sex life, so you don’t, you don’t you don’t and it’s so untrue and and and it is more for me it’s easier to kind of prove that the other thing because at this point at 45 years old what do I have to prove to anyone, right? So whatever assumptions they have that’s on them I will tell you that because I am somebody that says it out there I will often just disclose things that may be surprising and it sounds like I'm putting them through a test and I’m really not but the goal always is for them to realize that I’m a full human being with good bad and indifferent and all the flavors in between accounting being a full human being. That makes sense?

**I: Absolutely, yea definitely. Mhm. So in terms of those assumptions do they ever do they ever say anything that kind of, I mean well sounds like obviously you’re an extrovert and it sounds like you take the time to kind of educate them. Do you, do you do that kind of in response to to maybe the less than ideal experience or do you ever do it proactively to kind of prevent any type of experience?**

P: Well, you know, it it depends. They’re some- okay, here again is another assumption. You’re a person with a disability so therefore you don’t work, you don’t, you know, your life is very restricted. When I when I go to my doctor and I’m explaining that I need compression stockings because I sit 18 hours a day and because I have a very active lifestyle and I don’t understand how the pulmonary embolism thing happened to me because even though I don’t walk around, I transfer 20 times a day. I get, you know. When I say 20 times a day I’m not exaggerating. I go bed to chair, then I go in the bathroom brush my teeth duhuhuh, then I go from the chair onto the shower bench, then I take a shower and I get out of the shower bench and get back into the chair and I go to chair back to the bed to get dressed. That’s just the beginning. So that’s like about six or seven times. Then my job typically all of the jobs that I’ve ever had- my brother is a very institutional guy, he works at [college], he’ll be there till he retires, that’s him- that never appealed to me. One of the reasons I wanted to be a reporter even 20 years ago when I graduated was you can do it anywhere. And remember this is before a laptop. So you would call it in and you weren’t restricted to the the hermetically sealed environment of an office. I don’t mind an office but it’s not, you know. So when you’re explaining to your doctor all of the things that goes into your day being a productive day, there’s often as they look at you like “you do all that?” Still. Now, it happens less and less the more the doctors know you. Umm and like now I’m in a phase in my life where for the last year and a half I’ve been working on forming my own consulting business. So the insurance’s ask for my provider name. Because I have cerebral palsy I’m eligible for Medicare. I don’t have Medicaid because I earn too much money. But when you go in and you present that Medicare part, the assumption is then that you are just like every other person with a disability. You wake up, you watch Barry Springer, somebody comes up so you get dressed.. and there’s nothing wrong with that picture, right? And and one of the things that’s really cool as you mature is, as I’ve matured anyway, is now I don’t feel like I have anything to prove. Make whatever assumptions you want to make cause that ignorance is on you, not on me. I can’t own that ignorance anymore and it’s burdensome to always try to put in to people’s misconceptions because who has the time for that.

I: Right. But do you feel like it ever has had a negative impact on your healthcare in sense of, for example, you mentioned certain assumptions like, you know, how we don’t have sex or something. So do you ever feel like they don’t ask you certain questions that are medically relevant toward that?

P: Yea. Yea. And again I’m the person that puts it on the glass. Yea. Yea and I I I sprinkle those things in that I think they should know. Umm it’s very interesting because I have learned to to not let what other peoples’ assumptions are prevent me from getting what I need from that person.

**I: So you push any- like fill in the gaps if you will.**

P: So if if I if I see that they’re not asking me something that’s directly relevant and it would help me to get from point a to point b in their understanding and get better medical care, you kinda you kinda have the responsibility to try to jump the hurdle. Now my brother looks at it different. He’s somebody that says why should I have to, you know, prove whatever to anybody and I I get what he’s saying but for me, I want the path of least resistance. I want to get what I need whether that’s medication that I need for my blood thinners or whatever. If I don’t let you understand that I do sit eighteen hours a day and- then you’re not going to be the person to give me the best medication. Back to the doctor I was telling you about that that that that ummm that we’ve severed ties because her office is very small. One of the straws that bumped the camel’s back, right after I had my PE umm you have, once you’re discharged from the hospital one of the protocols is you have to do something called taking your INR levels. It’s how how umm cause in the beginning it’s trial and error to see how much blood thinner they’ll put you on.

**I: Okay.**

P: She didn’t call me back. So I got the medication for the three day period. Cause what they do is they- three to five days you’re on the blood thinner then they do blood work to see how you did. I- scared to death, right? Because this is deadly crap. (laughs)

**I: (laughs)**

P: So I called her out after we did the blood work and they didn’t call me back for two days like Friday, they didn’t call me back till Tuesday. Between Friday and Tuesday I had so much anxiety because it was- and I’m typically not an anxious person but this is a new diagnosis, the doctor was very clear on discharge that I needed to do this at least for a month until we got the numbers right and then we could decide on medication. So the fact they didn’t call me back really annoyed me and I just said “later.” And and and and I say that to say sometimes our medical needs as people with disabilities- I can’t talk about everybody, let me talk about me. Sometimes your medical needs are not taken as seriously as somebody else.

**I: Okay.**

P: And I kinda eluded to it a little bit in the in the PE story. I had called that same doctor, explain my symptoms, and she said oh today of the holiday weekend just relax. Then the first responders came. I was concerned I was having a heart attack. They did the EKG. Said no heart attack. I said but my heart is racing. They left. I decided to take matters into my own hands and go to [hospital]. Now, any other person who has to interact with first responders, who fairs they’re having a heart attack, who all of this stuff 9 times out of 10 they would say to them go to the hospital, go to the ER, and even if it’s nothing at least you go. I don’t want to say it’s directly related to the fact that I’m a person with a disability. However, there’s nothing else that I can point to. Because I was very emphatic about what my symptoms were and I I wasn’t alone, so my aunt was with me so heard me. Cause sometimes you think you say stuff and you don’t emphasize it as much as you think you do, but it was clear that I had, right? So I don’t know what else to attribute it to other than to say disability was the only thing that made it, you know, that made the reaction less urgent. As a matter of fact, umm one of the projects cause my my company is called “Nothing About Us Without Us” it’s from the disability mantra and what happened was I worked for a nonprofit for a few years and then again the whole grant thing happened and I’m like okay we’re defunded. And I’m like okay, do I go look at another job at a nonprofit? Do I go look at a newspaper? What do I do? I don’t know. So, my brother in his infinite wisdom said “you’ve always wanted to do exactly what you’re doing, why not do it for yourself?” And “I’m like, you think so?” And he’s like “yea, we’ll do it together.” So he’s my like silent partner who who helps backroll the websites and all the stuff that we need to do. But but one of the projects that Nothing About Us is trying to take on is how first responders respond to people with disabilities. And that goes that goes fire, police, that goes everybody. Because again, domestic violence for example is a big issue in the disability community. Right? Because of anger, misplaced rage- a lot of clinical stuff that I can bore you with. But the bottom line is, you don’t want it to be dismissed because oh it’s two people with a disability just having an argument, how bad can it get? And likewise in healthcare, you don’t want ever to say oh I’m having something and they go ohhh, you know. I’ll give you one more quick example. My doctor in Maryland, I’ve known him for about six months. Went in for a physical. They did the bloodwork. And my hemoglobin was low so she called me and she said it was a- their ARNP called me- said “Mr. [Particpant] your hemoglobin is about 6. Aren’t you feeling very tired?” I said “yea, I’m feeling kind of tired. But I’m a single guy in a wheelchair in a city by myself who does travel training, it would be a miracle if I weren’t tired.” She says “no ummm I think it has- I would actually like you to be admitted to the hospital.” And just then “oh.” Really, Felicity Huffman just got 14 days in jail for college admissions, whatever. So I’m a news junkie, it’s occupational hazard.

**I: (laughs)**

P: But umm one of the things that was interesting was umm even the way that was delivered to me, oh I want you to be- aren’t you tired I want you to go to be admitted to the hospital. It was not like an oo la la bright red flashing emergency, it was just like a casual conversation. Now my sister, her practice is in hematology. So that’s really what when I hang up with the doctor I’m like what does this mean, call me back! And ultimately what I got my sister she said oh you don’t need, you shouldn’t be transfused, you should be infused because if you’re transfused it was all this medical stuff and I did it. But had I not had that person to lean on, I would’ve gone okay. She just casually called and said do I feel tried. It wasn’t an emphatic thing. Now, I’ve known other people who’ve been called and the doctor said “oh you feel- I’m very worried about this I want you to go to [hospital] right away.” And you do. Because its emphasized- the approach is different the urgency is different.

**I: Okay. And, you know, you said that, you know, you correct or provide additional information. So do you find that healthcare providers respect and listen to what I’ll call your personal expertise?**

P: Umm yes and no. Umm I had a friend who had lupus. One of my best friends in the world. And she said you may- she always used to tell her doctors, you may have PhDs or MDs or whatever but I have a PhD in me. So I understand what this disease is doing and all that stuff. Likewise, I have a PhD in me. What that means is I get what the implications are of things, that doesn’t mean I’m not open to listening to medical professionals because they are experts, but I will tell you that it depends how you approach them as to whether they listen to you or not. What I mean is, it depends on the doctors mindset too. There are doctors that come in and want to have a conversation with you. There are other doctors who have five minutes after the ARNP has done everything and they’re “well Mr. [Participant] you have this and we have decided that, you know, this is a closer treatment thank you very nice to meet you we’ll see you in six months.” There are other doctors who come in and want to engage you and have the conversation like whatever. Those doctors are more open to the explanation and whatever.

**I: So they get some more balance at how they uniquely engage with any patient versus their knowledge of disability for example.**

P: Yea, yea, yea. Yea and I mean, by nature- and I could say this because I know many of them and many of them are in my family, doctors are not always very open people to learn from people who they believe are below their social status. What I mean by that is- you’re a little guy in a wheel chair. I’ve been a doctor for twenty years. I know more because I have PhD, MD, I went to medical school and I have a residency, I have an undergraduate degree. You’re this guy who has CP. I don’t know how educated you are, I don’t know- and all of these assumptions again. And and and and so BUT if it’s a peer-to-peer conversation, then I believe it happens on a whole new- on a different level. Does that make sense?

**I: Mhm. And do you find that that is at all different on a generational level as far as receptiveness to information?**

P: Yea, yea, yea. I think again it goes back to the timeline of disability, right. So doctors that are older, 60s 70s and beyond, even some in my generation who were not exposed to disability who disability is not a natural part of the human. They just kind of- the way they view their role as medical practitioner is more authority figure that’s going to do- that’s going to

**I: do to you**

P: distill knowledge from on high to the little people. As opposed to somebody who is willing to have the conversation and engage.

**I: Okay.**

P: Makes sense?

**I: Yea, yea. So umm one of the things that obviously- obviously you’re well versed in knowing that there are a lot of health disparities with regards to patients with and without disabilities and that’s kind of one of the areas that we’re looking at right now. So, part of the conversation is, you know, how do we make improvements if we aren’t even ummm documenting disability status. So, you know, one of the questions I have is what are your thoughts, should we be documenting somewhere in the electronic medical record or elsewhere, should we be asking you do you have a disability?**

P: I kind of feel that that is a question that needs to be addressed but doesn’t need to be put on a form, and I’ll tell you why.

**I: Mhm.**

P: Because the answer to that question goes beyond a line on a form. It should be about engaging the patient, the person with the disability, to understand yea I have spastic CP, this is what spastic CP means for being in my circumstances. If I’m even able to articulate it, right. For some people, they will see the question and it’ll be a turn off. And and and that and then that will assume a whole bunch of things- because the other thing is, when you put something in a medical record it kind of follows you forever. Right? So if you could have that as a soft question meaning a face to face question, an interactive question then yea it needs to be part of the record. But I don’t know that it needs to happen in the intake as to what you’re diagnosed with.

**I: So not with like name, age, address, all that stuff?**

P: Right. Yea, demographic information. Or or it should be maybe it should be a check box like yes I have a disability and then it should say something like this will be discussed further on the adoptive. So that the information is captured so that somebody knows that that conversation needs to happen. But not all the details of the disability because it may turn off the patient or it may cause the doctor to have all sorts of assumptions. Just be vague so that we know that maybe because the conversation takes more time for scheduling purposes I think that’s important to have that. Some way of knowing okay this is going to take a few more minutes.

**I: Right, okay. And so yea I think that’s part of the conversation, deciding what is the most appropriate way and the most useful way to ask this question and how. So you’re saying more in the face-to-face than a document.**

P: Yea.

**I: So so let me show you ummm this is not meant for the healthcare setting. These questions actually come from ummm the U.S. Census. And this is how the U.S. Census assesses disability. Arguably good or not, but wanted to get your thoughts. So, you know, I’ll read them to you but- Are you deaf or do you have serious difficulty hearing? Are you blind or do you have serious difficult seeing even when wearing glasses? Because of physical, mental, emotional condition do you have serious difficulty concentrating, remembering, or making decisions? Do you have serious difficulty walking or climbing stairs? Do you have difficulty dressing or bathing? And then because of same conditions, do you have difficulty doing errands alone such as visiting a doctor or shopping? So generally speaking, how do you- how do you feel about these questions? The wording, the content, you know, are we capturing the right things if we were going to ask about...?**

P: I like the questions on one level. And on another level I think they don’t work.

**I: Okay.**

P: Okay. I like the questions because they’re very specific. In other words, you don’t want to say are you deaf. It asks you do you have difficulty hearing. And it kind of drills down. Are you blind? It doesn’t ask you that. It asks you do you have difficulty with seeing or using glasses. It drills down. So I like that aspect of it. What I don’t like and what I think would be- I think disability is a very tricky area. For a lot of people even people who are profoundly disabled- that’s if you have CP or whatever because all your life you’ve grown with it- but I know many people that are aging. That definitionally they would be a person with a disability. But you ask them about their disability and they go I’m not disabled, I have difficulty walking. Now they advocated once ago well then you’re disabled then. But the reality is it doesn’t register like that. Right? It just doesn’t.

**I: So a question like do you have difficulty walking or climbing stairs would be- would capture them better than asking do you have a disability, you’re saying?**

P: Yes, yes.

**I: Okay.**

P: And so I like I like the way it drills down but I also know that if you ask the more generic question, “do you have a disability?” I think you’d get me to answer to me yes but you’d get that person in that wheelchair who says no I don’t I only have issues with my knee and I can’t go out at night because I really can’t see to drive at night, but I’m not blind, because there’s still stigma associated with disability.

**I: Sure and they don’t want to be associated with that because of the stigma-**

P: Mhm. Yup.

**I: Okay. So so, you know, we’re thinking about- yes we’re thinking about what are the best things- so this is like I said it’s intended for the healthcare setting but it’s another idea in terms of asking specifically do you have a disability, yes no or I decline to answer and trying to get a more of a functional way of identifying okay what difficulties or challenges do we need to know about so that we correct or just modify our care in a way that supports you better.**

P: Right.

**I: So in that respect, do you- do you think that we’re capturing things that you would want them to know?**

P: I I think that it would it is capturing things that that that are definitely important and you’d want them to know. The question becomes how do you put these questions in a healthcare setting so that doctor’s don’t feel condescended to or that patients don’t feel like their put off by them.

**I: So that’s my question to you (laughs).**

P: And I don’t know the answer. Umm I know intuitively that I’m not put off by these things but I know that somebody who is, again, in their 60s who is always seeing themselves as able-bodied, for them- for you to ask them these questions they’d look around like are you talking to me? I’m not that person, you know.

**I: Mhm.**

P: Umm I I I like the idea of the functional in the question rather than just the generic are you x y z.

**I: Yes or no…mhm.**

P: Because because so often it’s not a yes or no question. It’s yes I use glasses but I only use them at night or I only use them when in the sunlight or whatever. And and and you may say that, you know, it really doesn’t make a difference but to that person in their in their mind it does. And as much as we’re dealing with people of many different shapes and and and and flavors of unicorn-ness umm it’s important, you know, for them. And also I think that disability for most people particularly acquired disabilities, it’s a slow realization. One day they may say oh I’m not disabled, I only have difficulty walking. But then one day when they go to Macy’s and getting through the front door is the biggest challenge they’ve ever had, they’re like shit I’m disabled. And it’s that it’s a moment. And one of the things that I that I would kind of caution against is of course doctors are in the business of delivering diagnosis but to some people the shock of realizing oh my god they’re seeing me as that disabled person may prevent from them curing other (inaudible) that may be directly (inaudible). Does that make sense?

**I: Absolutely, yea. Mhm. So so if I- let’s just say if I was your healthcare provider and I asked you these questions and you answered yes or no for them. Would we be done with our conversation or would- it there anything else that you would want me to know because it was specifically relevant to to the your care?**

P: Umm what I would like at the end of whatever many questions is asking the person is there anything else they’d like to share. Because THIS kind of puts them in a box but THAT opens up a conversation.

**I: And would you maybe even do that per each question you said yes to so that way detail specific to can be provided?**

P: Yea. Yea. Yea. Because you have difficulty hearing. I don’t think I have difficulty hearing but there was that one time at the concert, right. At that Christina Aguilera concert and she like blew out my ear and I- woah I guess I do. And then and then that squares the conversation. And it’s a softer it’s a softer conversation, it’s a softer way to get the information that you need that’s relevant but that doesn’t put the patient off.

**I: Right. So this would be kind of a starting point and then the dialogue, the softer dialogue would capture any nuance, the other additional things that were missed…**

P: and I kind of feel that the training not only has to happen with the MDs, but it has to happen with the support staff as well. When I say that I mean the ARNPs, the greeters, and then the receptionist, all of those people because those- the way those people interact with the person is the- it’s sets a tone for the entire conversation.

**I: Mhm. Right.**

P: Make sense?

**I: Absolutely.**

P: and and often you have more interactions with the support staff than you do with the actual physician.

**I: True.**

P: I think the other group of people hat definitely need to have this conversation is the nurses. Right? Because if you make it to the hospital setting, your interaction with even a huge medical team of doctors will be very minimal. Your interaction with your nurse that is there on a 12-hour shift is going to be so much more. And I think I think still in the nursing profession there’s still a lot of paternalistic views about people with disabilities. I remember after my PE umm it was one of the rare occasions that I was in the hospital by myself because I guess like we’re we’re a Jamaican family if one person is in the hospital, we’re all there for better or for worse. Nobody was here everybody was away. And I remember wanting to a take a shower- bit of a germaphobe, I was in a new environment whatever. And I remember the nurse was just very like how are you going to do that, like like very little understanding of what that meant. And also that hospital wasn’t super accessible, right. The hospital itself was but the room was not. And that wasn’t even a consideration. Now I had a male nurse after who said listen dude you just got a clot, chill. And then he got me transferred to another room that was bigger and whatever, and he’s like we’re going to do the shower but first you have to chill for a little bit, but I realize that it’s a concern. So, you know, and and just by that interaction- made all the difference with how I perceived it as a patient. It just had to do with our relatability to each other too. You know, and there are lots of cultural aspects of it as well umm because the big elephant in the room has to do with- how non-Americans and people outside of the American acculturation process view disability. Right?

**I: Okay. Tell me about that. Yea.**

P: Umm well disability generally, I can say this as a Jamaican, is seen as less than. Right? You’re less than capable, you’re less than fill-in-the-blank. So culturally, the idea of interacting with you in a real and meaningful way- not that important. Right? You’re a poor thing. And I think that not only translates to the small island where I was born, but it translates to a lot of other cultures and societies that view disability from a more paternalistic lens.

**I: So would you say American culture is similar to that or..**

P: No..

**I: What would you compare it to?**

P: American culture is a lot more open, generally.

**I: Relative to?**

P: Relative to- I’m not saying there are not imperfections, but I’m saying based on other cultures I’d say they’re more open. But I mention the cultural aspect because a lot of the people that you interact with in the hospital setting, the CNAs, the CPs, all those people, sometimes they’re not from the acculturated American school of thought.

**I: So there’s more diversity in…?**

P: So there’s more diversity and more- and so the prism that they view disability through could be influenced by cultural factors. Which I’m not I’m not saying that to be xenophobic or anything, I’m just saying it because it’s real.

**I: Right, it’s just another lens.**

P: A lens that- so what what I think needs to happen is those people also need to be brought in. I think the whole healthcare system as a whole needs to be brought on board the disability train.

**I: Mhm, right. So you mentioned- yea you mentioned the whole healthcare system so let me also ask you about have you had any experiences good or bad with regards to coordination of care? So, you know, if you have a team of people working with you, do they…?**

P: No, no because typically the way this works for me is by the time we get to the to the phase of the process where there’s a team involved i.e. surgery, hospitalization, or whatever, my anchor is usually the general practitioner or the specialist that knows me so by virtue of knowing me, those assumptions involved pretty much disappeared. So getting what I need from them is not as big a challenge.

**I: So they do a sufficient job of communicating whatever needs to be communicated because of your established relationship?**

P: Right, right. Now, if I had to do it cold then I would have to go through all of the processes that we discussed earlier in our conversation, but generally you don’t have to do that.

**I: Mhm. Okay. So yea those are all my questions. I know I want to be mindful of the time because we’re we’re a little over an hour so, but any other thoughts that you think I need to know that I missed out on or any other experiences that you want to share?**

P: The other thing that I would share is I really hope that this presents an opportunity for people with disabilities to get INVOLVED in the process. In other words, one of the beautiful things about the [health system] is that they involve civilians like me. And this is not in any way soliciting a job for myself, but this is simply saying by having us involved in the process, the conversation becomes so much richer. And I understand that not everybody has the desire, the cognitive ability, or many of the things that other people have, but I would encourage there to be kind of a be-on-the-lookout-for, when I say be-on-the-lookout-for rather than recruitment of people with varying disabilities, not only wheelchairs. Because disability is so much more than that. It’s about people with hearing difficulty, it’s about people with autism- even people on the autism spectrum who I understand experience all these kind of conceptions and misconceptions about are able to participate on some level in the discussion about their care. Because it’s super important that it, you know, I named by company Nothing About Us Without Us for a reason. Because so often, we’re just not in the conversation. And and it’s all well and good to depend on our caregivers appearance or teachers, etc. etc. etc. But ultimately, this person will stand on their own. And so you need to create a welcoming environment where that person feels comfortable to open up and share about their disability as much as you would if you went to the gynecologist office and they were talking to you about your pap smear. I mention that because it’s a touchy subject, but both things are touchy subjects. Women are now comfortable more or less depending on who your physician is to have that conversation. So we need to create an environment where people with disabilities are able to feel comfortable to have that conversation because with disability often comes a lot of fear and a lot of anxiety because we’re still a people and not too long ago we were institutionalized because we were different. And so often people are afraid to disclose because if I say too much am I going to make myself too vulnerable, which then is going to create a whole bunch of problems for us. So if we make the cocoon more welcoming and less fear of oh my god baker acting or something bad happening to me then I think it’s a beneficial environment for everybody concerned. Certainly the patient, but also for the practitioners because the only way we learn about each other is by having real dialogue.

**I: Okay, perfect. Thank you.**

P: Does that make sense?

**I: Makes perfect sense. Thank you.**

P: Thank you for your time.
