# Supplementary material for: Disability disclosure in healthcare settings for individuals with developmental disabilities: A qualitative study of patient and caregiver perspectives
Source: PLoS One. 2025 Aug 7;20(8):e0329328. doi: 10.1371/journal.pone.0329328 (PMC12331114; doi:10.1371/journal.pone.0329328)
Supplement: S1 File — (ZIP) [file pone.0329328.s001.zip › Transcripts/2019.09.16 Interview 12 Transcript.docx]

**I: Interviewer F: Female Key Informant**

1. **I: Alright so normally when I start I just for the record, we have informed consent, you’ve agreed to participate and be recorded. Is that correct?**
2. **F:** Yes, correct.
3. **I: Okay thank you. So–alright. So, we know, want to learn a little bit about, uhm, about [NAME]’s health care experiences. So would you say overall, have they been good, have they been bad, have they been both?**
4. **F:** We luckily have had pretty good experiences, I can’t complain about uhm, about his health care. As far as like his uhm, going to the doctor’s things being covered, not having to go through referrals a lot of times and, and stuff like that. Uhm, the only think that we did go through, part where they, uhm, didn’t want to pay for his uhm, his therapy, behavioral therapy. But that gotten resolved and so far I think we are doing alright.
5. **I: Okay, okay. So you said overall good experiences, great. So–so let’s–let’s, let’s kind of get a better sense of what, what makes them good experiences. Like what happens that you think okay this is a quality experience.**
6. **F:** Well not having to, to jump through hoops, to, to get, you know, appointments as far as like getting approval for appointments. And, ‘cus a lot of times the parents have to call and the referrals not active and if the referrals not active sometimes the insurance doesn’t want to cover it. And, thankfully we have never had that problem. He goes to the pediatrician and he has to see a thyroid specialists and he has to see a cardiologist once a year. And, so far we’ve been lucky not have to go through any hoops and stuff for that
7. **I: Mhmm. Okay so beyond the uhm, insurance aspects and the access to care, when, when you go to a health care appointment, do you always accompany?**
8. **F:** Yes.
9. **I: Yes, okay. And–and tell me a little but about the interactions from–from registration to seeing the nurse or the doctor all those interactions tell me what makes the quality experience for you.**
10. **F:** Well his pediatricians’ office he’s been with the same pediatrician since birth so they know him, and what’s good is [NAME] has a good character so everybody knows us wherever we go pretty much’s everybody’s friendly. The uhm, you know check in, you know I mean information change mom, no, no information okay, hey bye [NAME] how are you, you know. And then we wait usually the wait is not that bad if you know ***when*** to go. That’s a whole thing to, you know, try to go in between right after lunch or early, early in the morning. And uhm, otherwise it does get very crowded and you do wait a long time. So we’ve learned kind of by the ins and outs of being able to not you know, not have to wait so long. And uhm, once we get to see the doctor and you know get put in a room, they weigh him, they measure him, and so forth, take his temperature, and he while we are there, it’s for a well visit or because he is sick, and uhm, we never, you know, have a problem. Doctor comes in and, the doctors spends his time with us and he’s very thorough usually with him. And uhm, then if, he you know we have to wait for testing you know come and take him to the testing. Although [NAME] doesn’t like to be poked and prodded. So that can be kind of a challenge but he’s got a little bit better. You know, so uhm, but it, its uhm, we’ve had good experiences with the cardiologist as long as you make your appointment. Yeah we might have to wait a little bit but they’re, they’re nice they’re polite, you know–I, I can’t, say I’ve had a rude experience. With any of his doctors. I did have uhm, once uhm, or twice we actually and the same infectious disease doctor when he was at (location) he was there one time for four and a half weeks. He was on antibiotics for suspicious of endocarditis, which is an infection in the bowels of the heart. And he, his cardiologist he’s said since birth said no, I don’t trust, I would rather you do the treatments, stay in the hospital and rounds of antibiotics on IV, because if not, mom, its, you know, valve replacement surgery and you don’t want to have to put him through that. And the infectious disease doctor said well I’m not sure yet but I really doubt he does. I really don’t think he needs this. My kid, I’m not going to take the chance, you know–and she kept, and when we were there the last time also, uhm, for, where we had to see her was for uhm, he had uhm E. Coli. And uhm, she’s, she can be little bit rude. And she’s a just, just I guess her character. She’s got a strong character. But she’s not, I mean, she doesn’t have a good bedside manner. But everyone else that we've dealt with, the doctors the nurses have all been super nice with him. Even (location) when he had to have some teeth pulled, and uhm he had some teeth pulled out–I wanna say when he was about six or seven and he was there for his adenoids and his tonsils should (inaudible) everybody was super nice super attentive to him. So, he’s had–we’ve had luckily like I said, good experiences, can’t say I’ve really had a bad experience with health care yet.
11. I**: Okay great. So maybe those instances with the teeth pulling or maybe getting blood drawn or shots like that, what uhm, what uhm is the process that they use that kind of make it a pleasant experience. Can you think of in those more challenging situations?**
12. **F:** Well, they, we try to draw his attention to something else. Uhm, we’ve, uhm, have people hold him down, uhm and screaming and cry but you know thieve had to do it, but lately he’s gotten older so you know, me talking to him, and coaxing him and the nurses also talking to him and telling him everything they’re going to do, and so forth, then, before we wouldn’t really tell him, we just tried to get it done, because, I mean he’s strong, he would push his way out of there if he could. But, they did very pleasant as far as telling [NAME], we are going to do this or we have to do this because it’s going to make you feel better and you need to have it. And you know for you to get better, and he scream and cry and so forth, but we get through it like he’d have to have a couple nurses holding him down and he sometimes would try to kick his way through them. You know, but they all get their composure and are very nice about it. And they are pretty good, pretty good about getting his veins pretty much right away. So that was a good thing. Uhm, but uhm, they, you know they always help explain to him, or tell him maybe they’ll give him something a toy. or something to play with or to keep. I know (location) we always come home with, when we stayed overnight, bags–bags of toys like it was Christmas. And I mean big toys not even little dollar store things. I’m talking big trucks, remote controls, but they were always you know really nice with him. And uhm, (location) same.
13. **I: Mhmm. And uhm, you know some, some parents say that they go out of their way to kind of prepare for and ensure the best experience possible. Is there anything you do ahead of appointments to kind of, make sure it’s the best possible experience?**
14. **F:** Well as far as if we have to go to the hospital or something like that, when it’s something of the emergency type of thing I try to, you know, talk to him, on our way there, and explain to him what is probably going to happen what they’re probably going to have to do. And then I’ll try to talk to the nurse outside of the room or something. Usually mom will come with me, lately she hasn’t because, she’s gonna be 85 years old so it’s getting its, not good for her health to be there because usually he gets sick every time she goes. So when I’ve been the last two times I’ve taken him myself and so, but, uhm, I try, I try to talk to them and he might not like this. He doesn’t like Band-Aids he doesn’t like you to put a Band-Aid on them, he doesn’t like tape with IV’s. And, you know, they’ll tell him, unfortunately we have to put the, the sticky stuff, the tape on because it needs to hold the IV in because you don’t want it to come out. So if it comes out we have to stick you again, but they’re all, they, they, they’ve all been super accommodating and super nice to him. And to me as well. And uhm, you know, me, they also talking to him. I’ve always told them since he was a baby, you know before he could talk everything I was doing or everything that was going to happen. So he was, he grew knowing, you know, pretty much what was going to happens, so. But uhm, and since, thankfully everything’s gone very smoothly pretty much. Except for the time that he had to get stitches because of the dog bite in his mouth. But yeah a dog tore out, he went to go kiss a rat terrier, I was talking to my neighbor and he had a rat terrier. And [NAME] went, I saw him going down to go kiss the dog, and is said [NAME] no, I saw the dog and the dog just jumped up and bit his face. His face always bleeds more, and I was–I didn’t even know where it was bleeding form, he was just screaming. But he actually came out of there with all these little dots all over him because he had broken, I guess they had broken little vessels and, and you know and he just, screaming and screaming and screaming because they had to give him stitches and I mean no matter how, you know anybody talked to him that day or, and how nice they were just wasn’t happening for [NAME].
15. **I: Yeah no calming him down after that experience.**
16. **F:** No, I mean once, once it was over we were okay. It was fine. Once it was done and over with and he was off the table and everything, and he was okay. But as long as they were sewing him, even if he couldn’t feel it, he still knew something was going on, and they held him down and the nurse had to hold him down and he didn’t like that. And I was singing to him talking to him, everything that I possibly do to calm him, but its not happening that day.
17. **I: Mhm. And so you talked about things you do and tell [NAME] to prepare him. Is there anything you do ahead of time, uhm, as far as interacting with the healthcare staff?**
18. **F:** No, I haven’t had to thankfully. I haven’t had to call in advance and say okay [NAME] needs this, this, and that. Thankfully I haven’t had to do that because I guess the relationship I have with [NAME] and I explain to him and I try to make things easy on my sider for him. So I haven’t had to you know, do anything. I mean, maybe the time that he got the teeth pulled and the, and the uhm, and the tonsils out when we did the pre-op they asked me questions about you know uhm, is there certain things that make him uncomfortable, and things that he doesn’t like. And I explained he doesn’t like an IV and he doesn’t like this. They were good about giving him a little bit of groggy just before that in order to calm him down just before that. It worked fine.
19. **I: And so when you share that information, you feel like they listen to you, they respect your opinion, and they apply it?**
20. **F:** Yes, yes they did. They did. Two times he s had to have surgery, well. And they were very, very good about it.
21. **I: Uhm, so one of the things uhm, uhm, parents will say is that they, they like to say ahead of time, my child has this or that disability and that means this or that for their care. You kind of mentioned few things about not liking needles or what not but-**
22. **F:** Whenever, I’m asked or, or I, you know sometime volunteer when I’m making an appointment, I do tell them that he does have down syndrome. And, uhm, you know, I, we have, basically we have found out through trial and error, from his cardiologist that he doesn’t like to have his (inaudible) put on. And so, they already know that and so when we get there we try to make a game out of it so that we can try to get it on him. Hmm, his uhm, you know as far as like me letting them know he has down syndrome he doesn’t like to be poked, uhm, getting an IV is hard, if he tends to need that or, that, that’s going to happen. Uhm, pretty much he’s, he’s, he’s, an easy kid, I mean as far as getting along with people and, and people liking him I mean his own character draws him to, you know, for the people just to, be good with him, to begin with. I really can’t complain. There’s, nothing except for that one, one infectious disease doctor that really isn’t, they gave me, a, like a bad taste you know, on those two occasions... but mom, can I ask her if she?
23. **I: Sure.**
24. **F:** Mom, can you recall, any, any kind of negative experiences that [NAME] had in the doctor or in the hospital that, that, that caused us to, him not to be comfortable or, to feel, like how, how they interacted?
25. **Grandma:** No never.
26. **F:** Everybody’s been so, lucky, and so blessed that he, that we have had really good care for him. And I know it’s hard in that sense, it’s hard to believe in some respect because a lot of people go through a lot of problems and I know because I go through from my friends hearing about [name] mom, what he goes through and what she goes through with the girls sometimes and getting appointments and jumping through hoops for this and that and another. It’s hard. But luckily we have been lucky. And he’s, he’s a pretty healthy kid. So we don’t need extra, you know. Stuff. A lot of extra stuff, health care.
27. **I: Right so not a lot of extra accommodations or anything. So do you feel like you need to mention down syndrome at all, or do you feel like that’s not something they need to know for any particular reason?**
28. **F:**  Uhm, you know, I’ll, I’ll mention it when I make the appointment just so that they know, and you know, I don’t know if, I need to or not but I always do. But they’re aware in case they need to do anything special on their end or any kind of testing or anything like that. Uhm, but uhm, not for any other reasons.
29. **I: Mhm, and do you feel like, well, I would say first when you give them the information do they react in any particular way?**
30. **I: Okay.**

(inaudible)

1. **F:** Yeah, his, his, his, behavioral therapists really uh good with him, Sam, is-
2. **I: And you say that some of the other people, that you, that you go to that you interact with have, don’t have the same experience. They have some issues. Do you have any sense of why, you said you were lucky, any reason why?**
3. **F: I think maybe the insurance and I think maybe, I mean, I’m a single mom, he has full Medicaid, I don’t pay for his health insurance. I know a lot of people that have private insurances they’re limited to a certain number of–of-of visits a year, a certain number this year and then you know, when they have to get referrals and stuff they can’t. I’m, I’m, like I said, we’ve been lucky that we have had, I’ve haven’t had to jump through hoops just to get care for him. And where I’ve seen other parents have to do that and you know, yes, we wait for appointments sometimes because we, the doctors busy and they have appointments are way out there. Yeah that’s normal. I mean, you know, thankfully. Like I said he’s healthy. pretty health. He was born with two holes in his heart. One was closed and the other is the slightest bit open. And, and it’s not a concern as far as the doctor is concerned. They–no surgery anticipated for him to be needed and you know, I’m lucky.**
4. I: Mhmm.
5. **F: Just worried about his weight and him in the future needed, you know additional medical things because he does, he doesn’t like to eat healthy. That’s the biggest fight here. Is, we will, we will make something, I, won’t even eat dinner now because I can’t enjoy my food because he doesn’t want to eat this he doesn’t want to eat that, I don’t like this I don’t like that, well that’s what’s for dinner now and we aren’t going to change the dinner for you.**
6. **G:** The evil eye.
7. **F:** Yeah, the evil eye is given!
8. **I: So when you do mention down syndrome, do the nurses the doctors, do they seem like they know what that means.**
9. **F:** Yeah they seem comfortable and knowledgeable–I mean. I took him to the eye doctor and the eye doctor I was worried they, you know, they wouldn’t measure him right or feel comfortable because a lot of the time they’ll do glasses and they don’t fit the bridge of the nose right because, because of the way the bridge of the nose is. But the doctor was very good with him, and he, seemed to know, and know how to, how to, hot to treat him. You know.
10. **I: And no, no assumptions of no capabilities or anything like that?**
11. **F:** No. No. Like I said, he himself, you know shows, and, the way he carries, you know, interacts with people, he shows you that he’s high functioning which is great so that he doesn’t–he can pretty much speak for himself.
12. **I: So, when people interact with him, they already get a sense, even if they have initial thoughts they already see based on how he interacts-**
13. **F:** No, he’s very, he can speak well, he can thankfully, he can, he can interact with you, he can know what you’re saying when you talk to him. He’s not in another cloud somewhere. Which unfortunately there’s many kids that are. And you know, it’s hard and I’m, I, don’t know how some of their parents do it. I’m very blessed that [NAME] is high functioning as he is. And I started him, and I started him with therapy when he was 8 weeks old. And even that, went wonderful. I mean everything I just, from, from, from when he was born we lined up his therapies and stuff, we started therapies, he went to the uhm (location), and they were fantastic with him. They, he just learned so much, you now and was able to, the muscle toning, everything, was able to be strengthen, they, hes too strong now! (laughs). So, he’s, he’s very strong and I thank god he’s, he’s, able to you know, do almost everything that pretty much any other kid can do. He plays sports, and uhm, he’s been, got two, two medals in the Special Olympics this past–in the summer games. He won a gold and silver.
14. **I: Oh in what specific sport?**
15. **F:** In running. In running track and field and, and uhm, and, throwing for distance.
16. **I: Excellent.**
17. **F:** Yeah so...and so now he’s going to play football with the dolphins, doing uh, uh captains training, uhm, event that they get to sport Sundays. And uhm, September, one October, one November then November 22^nd^ is the last one and it’s , uhm, kind of like a captain leadership type of program and he was chosen from his school as one of the children to go take part in it.
18. **I: Okay excellent.**
19. **F:** I’m proud of him.
20. **I: Yeah as you should be. Going back to the infectious disease doctor that you said was a bad experience. You mentioned bad bedside manner. Can you tell me a little more about–about what just didn’t work in that situation?**
21. **F:** She was very cold, when I, when I would talk to her, it seemed like she wasn’t even paying attention to what I would say to her. And she was going, she says well I, I just don’t think he needs it, I think you’re dong this, for, for, for as a waste of time. And I’m like, the end result if he winds up, I just could you tell me 100% that he doesn’t have it? She goes I can’t tell you 100% what tomorrows weather is going to be or anything else is like in life. Come on. I mean, like I’m are talking about my child here. Not talking about uh, an animal you know, and even that, I, I, I wouldn’t want them to tell me that about her. You know? And uhm, she’s like, she says, I just very doubt, I’m very doubtful that he has it. I said you can be as doubtful as you want. I said his cardiologist that knows him since he’s one, who was the first doctor to see him and you know after the Pediatrician in the NICU, seems to think otherwise and I trust.–and he came highly recommended to, a lot of other moms I spoken to and talked to have used (Doctors name) and they, you know, he’s a fantastic doctor. And, uhm, he, thought it necessary and I, I just, you know. Said, no I’m going to go ahead and, and do it and she rolled her eyes as if I was not, I was doing the wrong thing. And Yeah he suffered for the, with the antibiotics because it made his, his, his, uh, get the runs from it, he had so many antibiotic and strong antibiotic that it was, I mean his poor butt it was bleeding. And I literally had to sit there, the nurses were so great, they were like mom this is what we need to do. They ordered extra magic spaces they use in the hospital. They said just blow the oxygen right on his butt. I’m going to bring you a tube for it and you just sit there for it, and it will clear it up. That’s the best thing for hit. And it did, it was wonderful. But, you know, the doctor would come and she would say Oh I see you’re still here. I’m like, yeah I am.
22. **I: And do you think she would be like with any patient? Or do you think, you know having a disability played any role in the connection?**
23. **F:** I, I don’t know, I don’t know, I honestly don’t know. I just really, didn’t, she just left me a bad, a very bad taste in my, and I, and I told his pediatrician and, you know, the other uh nurses in the floor, she’s got a really bad, bad bedside manner. And they say oh she can be a little bit, you know, rude and I said yeah but that’s not right you’re dealing with children you know. If you’re working, and they go well she works she’s the infectious disease doctors for all the patients that are hospitalized. Maybe she’s not working with kids that much and when she has to maybe it’s not her forte. She just doesn’t like working with kids. Maybe kids with special needs even bother her more. I don’t know–I just, but-
24. **I: And did you say anything directly to her or did you just mention it to other people?**
25. **F:** No. I just mentioned it to the head nurse and the other doctors. I didn’t want, you know, I was afraid maybe his care in the hospital would you know, be, uhm, what’s the word I’m looking for?
26. **I: Negatively impacted? Or? Mhmm.**
27. **F:** Yeah and there’s another word- compromised. You know, if I, I, you know if anything I would have said to her, point blank this is my child, his doctor says I should do it, and I’m going to listen to him and I’m sorry.
28. **I: Mhmm. Okay. Well one of the things we are working on right now, is, we, we know generally speaking, individuals with disabilities get inferior health care quality or have lower satisfactions with the quality of care that they do receive. And so we are trying to kind of, figure a way how to tackle that and first thing is, we don’t even assess disability status like we would, asking people, their age or their marital status or their race ethnicity all those things. So, so one question I have is, do you think that, that is a good idea, appropriate to ask about disability status?**
29. **F:** Uhm [long pause] I think that I really honestly think they should be treated like anybody else, like any other patient. They’re, they’re, they’re a human being put on this earth the same as you and I, they shouldn’t have any, any cut backs on anything or anything on how they’re treated or the care that they’re given and no they don’t deserve this care because they’re not going to do this in life or not going to do that in life. No, no, that to me is horrible. I don’t think it should matter if they have a disability or not. They should have the same care, and, you know, and maybe, you know, they did, they shouldn’t have the right just to, to basically to act on what the issue is. I mean it helps to know so that you know how to handle the child or the, or the adult any kind of, any person that may have special needs and the needs that they may require, but as far as to, to cut back on their care because of, of, of a disability-
30. **I: So would you, would you your concern because asking would may lead to that? Or, or?**
31. **F:** It could. It could.
32. **I: But you also say that it could be useful in connecting it to additional-**
33. **F:** Yeah its kind of like a catch 22 because you know, it may be necessary for uhm, say a person that's autistic and non-verbal that they know about it so that they know you know, more how to handle it know how to treat whatever they’re there for. Supposed to a persona that can speak and, and tell you what’s wrong with them. You know, I know they will probably take a care taker but maybe that care taker goes out of the room for a minute or something. Are they going to be a parent that has to work and cannot stay in the hospital? I thank god I’ve been able to stay with I’m 24 hours a day. Either myself or my mother have been in the hospital he has never been left alone in the hospital. But I’ve seen children by themselves in the hospital. And it just freaks me out for that child, because then I’m like I think about it and maybe it’s not because the parent doesn’t care, maybe the parent has other kids and if they take off the day and they don’t go to work they’re going to lose their job. You know I’m lucky where the places that I’ve worked have been very accommodating as far as he goes. As far as my son, being I stay in the hospital. Maybe I wouldn’t get paid for it, but I still was able to get out without losing my job. And I didn’t work for a long time after he was born to stay at home, I mean we were lucky that my mother and money saved away and stuff and that we were able to do that and yeah, that, but it came in handy. But looking back at it. I wouldn’t change it for a thing.
34. **I: Sure.**
35. **F:** I mean I thank the world, I mean time with him, is why he is the way he is today. Because I was able to give him the therapies he needed, I was able to take him there, I was able to spend the time with him, my mother spent time with him, you know we have always just been hands on with him contently so it’s been good.

(side conversation inaudible).

1. **F:** So-
2. **I: So, so, it sounds like you, you have reservations about how that information would be used, but you would be more interested if we were going to ask something not asking specific what type of disability, but also what also what additional assistance would you need?**
3. **F:** Would you need, mhmm.
4. **I: Okay, and what, what format would you think would be ideal in that situation. Should it be something that you fill out when doing your intake form or is that something that should be a conversation when you get in to finally see the health care provider?**
5. **F:** Maybe uhm, the initial paper work your filling out, maybe if, sometimes the doctor doesn’t even read thought hat. Sometimes there’s some busy days. You get in there and the doctors like you know, you’re here, what’s going on, is what they’ll ask. So, it’s, like I said, it goes another way, because can put it on the form but if the doctor does have time to read the form, then that doctors not going to know that this person may need a special kind of assistance. Or they should need some assistance uh, writing, uh, or filling out a form or, discussing uhm, what’s wrong with them, or something because they won’t necessarily have to have a special needs some people just are either very shy or, or people who are depressed that maybe don’t like to talk to anybody, you know these are your average people that are walking in the street.
6. **I: So general needs that could kind of apply to anyone..**
7. **F:** General that can apply to anyone yes.
8. **I: So, obviously I want to get your thoughts but I do have kind of a starting point for conversation if you like, so these questions are not for health care, these questions come from the US Census to establish disability status based on these six questions. So, I will read through them and I just want to kind of get your thoughts about what you think, whether or not they capture anything you would want to tell a health care provider. So first honest being about difficulty hearing or seeing. Uhm, then looking at uh, do you have serious difficulty concentrating, remembering or making decisions, any difficulty walking or climbing the stairs. Any difficulty dressing or bathing on your own. And then any difficulty doing errands on their own, that would be visiting a doctor shopping, anything like that. So do those capture anything you would want to share about [NAME] with a health care provider?**
9. **F:** (Pause). With the concentrating maybe, or the, uhm, difficulty concentrating, remembering making decisions, making decisions because he wouldn’t be able to, make uh, life changing decisions. On his own, yet. And that’s also because he’s still a child and you know, because he’s, he’s below his age level as far, you know, his, social behavior and how he, how he and his mind assess things. There’s time you can have a conversation with him, and it will be like talking to a normal fourteen-year-old. And then he will do something five minutes later that’s the behavior of a two-year-old. And uhm, uhm, things like uhm, needing uhm, to, and then, hmm. So uhm, this would be to find out, uhm, if they need any, any assistance going to the doctor. Uhm, and uhm-
10. **I: Yeah so are there any other things you would want- like for example earlier in our conversation yo mentioned wanting to let them know he’s not a fan of needles or a fan of drawing blood, something of that sort. So anything beyond that, that you would want them to included that you want them to know?**
11. **F:** Just that he doesn’t like to be poked, he doesn’t like sticky Band-Aids, he doesn’t like anything sticky that sticks to his, to his body that uhm, that you know, it can be done, but it needs to be done-
12. **I: Maybe touch, something specific to those cases?**
13. **F:** (inaudible) don’t like to be touched at all. And it is hard for them. So, uhm, something along the lines of maybe uh, I wouldn’t even know how to word it honestly. Uhm, just some kind of a sensory issue maybe.
14. **I: Mhmm. Sure. So beyond, if we kind of lump sensory in, it could be hearing vision, something about touch.**
15. **F:** Right.
16. **I: Okay. Anything else that kind of stands out? Or does that kind of capture everything?**
17. **F:** Uhm, (pauses). Uhm, no I think this would capture pretty much everything I think except you know adding maybe a sensory issue or something like that to, (whispers).
18. **I: Mhmm. Anything about, any, any, social interaction issues, and this could be based on your experience not with just [NAME] but any other person that you’ve interacted with, do you feel like there’s anything that a health care provider would want to know about how he interacts socially or do you think that would be captured in what you talked about before with making decision?**
19. **F:** I think pretty much it would be under making decisions, or uhm. It depends, what, what health care provider you’re going to, who you’re going to see. If they really needed to know that like I, I, maybe would need to volunteer that to, to the eye doctor. But I need to let the neurologist maybe know that if I was going to a neurologist or you know, even his regular PCP as far as you know, as, his developmental, uhm, uhm, and social behavior was.
20. **I: Mhmm okay. So much need to be specific on depending on, its, its, all, its you’re on a need to know basis kind of thing.**
21. **F:** Exactly. Exactly. I mean, its, its, sad to that you, you know go, certain places and its try, your just your care depends because some places if you have a special, a person with special needs you lower down on the totem pole than another person because they may feel that you don’t need it so to speak so much as the other person. You know I haven’t experienced it but I’ve heard of other parents who have experiences things like that and it’s just-
22. **I: What do you think is behind that belief? Like where that belief comes from? So you’re saying they’re not seen as equal and need less health care or?**
23. **F:** Uh…I would think they, would need more services but, some uh, as far as my conversations with other parents they’re not seen that way and it may be just what society is, has put out there. You know? I mean now thankfully; people would have special needs or have some kind of a a disability. It’s become more average, more out there that there’s so many people out there that have special needs out there that it’s become more acceptable now. Now, thank god, back twenty years, back, it wouldn’t have been like, as easy as it is now, I mean people were, were frowned for you having a child that has special needs. I mean I, my, my boss, asked me why I would do that myself.
24. **I: Mhmm.**
25. **F:** Why would you have a child. Why would you do that to yourself. You’re young, you can have another kid. No, no. first of all I was very upset with that. Second of all even if I didn’t, I still would have. I said, who knows not to say your child doesn’t get sick, I hope it never happens but what if you’re child gets into an accident tomorrow? And what are you going to do? It’s not your kid no more? Throw him somewhere?
26. **I: So you think things have changed for the better?**
27. **F:** Things have changed for the better. I think how society accepts-
28. **I: We are not the full way there but-**
29. **F:** Not the full way there, but a lot better than they were. They used to be considered retarded. That word is not acceptable to be used. Unfortunately, I’ve run across people who know not to say it to him, but just meaning stupid in general or, moronic. Or whatever. And, don’t use that word and I’m like really? Especially in front of me you’re going to do that? I’m sorry you know I don’t mean it that way. Then don’t say it at all. Why do you have to say that word? It’s just how people re. but society has been a lot more accepting. I see a lot more jobs with people for special needs I see, (inaudible- dog barking) more models with special needs and stuff opposed to, opposed to before where there were, it was not, you know? Before there would, you wouldn’t see that in a magazine or you wouldn’t, you wouldn’t walk into a store and maybe Publix, more so, because they, they’ve pretty much have been accepting you know, giving jobs to, people with special needs for a long time, but, even more so now you’ll see it another stores too. Still not completely there, but it’s getting there. It’s getting there. So hopefully with health care, you know, it’s also getting there. I mean like I said, I’ve been very lucky and very blessed that the doctors who we have chosen to see and wo have been available to us have been very good and, and when I have taken him to the hospital I have not gotten you know, less care because he has special needs or anything so. I, I’ve been very lucky but I have heard parents complain a lot also.
30. **I: And, and the uh, health care providers that you use now, did, you just get lucky off the bat? Or did you go to specific ones because they were recommended by anyone in particular?**
31. **F:** Uhm, starting out with his pediatrician, it was a friend of mine’s pediatrician and I just knew he was very good and I have asked him and I’ve called and I’ve spoken to him and I made an appointment to go see him before [NAME] was born and he told me he’s had a few children with down syndrome and that he did know and his colleagues were very knowledgeable to deal with down syndrome and other disabilities. That uhm, that I could feel confident that he would care for him well. Uhm, there here was another doctor uhm at the time and its very famous (doctor) uhm who has even taken care of, opened up a home for children with down syndrome and stuff but I felt he was up there in years, and uhm, he would retire, and I would be stuck looking for another pediatrician.
32. **I: Mhmm.**
33. **F:** And so we went with (doctor) and I’ve not had a bad experience yet. Has he grown and gotten busier? Yes. So the wait times maybe a little bit more like I said, I know how to play with the schedules so (laughs).
34. **I: Mhmm early.**
35. **F:** So I pretty much, we pretty much get in and out but uh, he has gotten, gotten bigger and I’m glad. I mean he has enough staff to, to be able to, uhm, take care of it. But uhm, what you would have to wait normally if you went in or made an appointment for just the middle of the day or you know just decide to go in at 10 in the morning, you better make it at 8 o clock in the morning and be one of the first patients or go in right after lunch and get there a little bit early or before. You know, other people come in. You learn the ropes here and there.
36. **I: Of course. Uhm, just to follow up on this, so, you know, I showed you these few questions but we also said the initial intent you like the idea of kind of asking for the sake of kind of connecting people to additional resources that they might need, right? So, so do you think those additional questions should also be included in writing or that is something that is discussed based on the answers to these questions when you interact with the health care provider? What do you think about that?**
37. **F:** I would just, based on, on these questions and being, uhm, and maybe if there were some things here that they saw that maybe okay and this one person needs it or assistance in certain areas, let’s have them maybe fill out another form, you know and answer some more questions fill out another form that has more in depth to what they really might need.
38. I: Some thins specifically for people that answer yes to any of these that gives the detail.
39. **F:** Right.
40. **I: Okay, okay. And how often should be asked these questions. Is that like every appointment or?**
41. **F:** No I don’t think it needs to be every appointment if you’re seeing the same, the same uhm, doctor, I think that maybe you know, once every, six months to a year if it, maybe the person, you know, there’s a change that the caretaker or the patient themselves if they are able to can let them know of a change.
42. **I: So only a change to update me on.**
43. **F:** Yeah update information.
44. **I: Okay and od you think it’s their responsibility to ask these questions or do you think its your responsibilities as a caregiver?**
45. **F:** No I think they should ask. I think they should ask. I mean, you know, I don’t, I mean, I don’t see anything wrong with asking.
46. **I: Mhmm.**
47. **F:** The questions I mean uhm maybe you know your average person might be like oh I can do this I can do that, why are you asking me all these questions, they might be offended a little bit. But you know, I know where I would say to that person you know hey you might be in a predicament one day where you actually may need a little bit more assistance because you’re getting older or something has changed in your life. So it’s something that may benefit you as well.
48. **I: mhmm sure. Okay well those, those are all my questions. Any other thoughts that you have for me or you got it all out?**
49. **F:** Really? No (laughs).
50. **I: Okay.**
