# Supplementary material for: Disability disclosure in healthcare settings for individuals with developmental disabilities: A qualitative study of patient and caregiver perspectives
Source: PLoS One. 2025 Aug 7;20(8):e0329328. doi: 10.1371/journal.pone.0329328 (PMC12331114; doi:10.1371/journal.pone.0329328)
Supplement: S1 File — (ZIP) [file pone.0329328.s001.zip › Transcripts/2019.10.25 Interview 14 Transcript.docx]

1. **I: I like to start the recording just by saying, just confirming that we went over the consent and you agreed to participate and be recorded?**
2. F: Yes.
3. **I: So just to start off, you know, when thinking about your-your son’s healthcare experiences would you say that he’s had good experiences, bad experiences, both? Where do you…**
4. F: [Long pase] Both.
5. **I: Both? Okay. So-so I‘d love to get in-in some detail about what made each of those experiences good and bad–kinda get some sense. So I guess let's start with the bad. So what-what about the experiences, like what were the experiences? Who was he interacting with and what made his experiences bad?**
6. F: For him, um...when he was 8 years old, he was doing–he can ride bike, he’s very functional. Uh–He was doing wheelies [inaudible], so he falls...he breaks his thumb, we take him to [Local Hospital Name] they said that there’s nothing wrong with him. So that wasn’t a good experience for him because all night he got swollen, he got [inaudible] so I took him to the hand center where he had to undergo surgery and they had to put, um, like, one of those...something to get the-the bone together.
7. **I: So maybe like a pin or like a metal…?**
8. F: *Two pins*. They had to put two pins and I didn’t like that [Local hospital]–he was a little swollen so I’m giving them the benefit of...maybe the x-ray wasn’t too clear for them but they said “No just put some ice in there, he’ll be fine tomorrow”. He’s always saying that they didn’t take care of his hand at [Hospital name]. When [son] was about 3 months old, we went to [Hospital name], he had a tiny, tiny scratch by the mouth, on the side of his mouth, and we put him in the water. The next day I read...something started. And I could see it going up and up and up. I took him to the doctor and he said he has to go to the hospital, they have to um, we have to put an IV. This is called cellulitis. And I go, “I thought Cellulitis was something else altogether” and he goes, “No. We don’t want it to go beyond his eyes.” But the thing kept going, I mean, if you could just...
9. **I: Like an infection?**
10. F: Yeah. Umm. He also remembers that they had a lot of trouble getting his veins to put the little IV. So finally they did, but it was not a good experience for him.
11. **I: Okay**
12. F: I thought they should have brought someone in with a little more experience.
13. **I: What type of experience?**
14. F: Experience on explaining a 3 year old down syndrome child, uh, what they were gonna do.
15. **I: So talk him through what to expect you’re saying?**
16. F: Yes. Yes. In a language that he can understand. He’s very-he’s pretty functional, and I have to do it myself. I said [name] I showed him a mirror, and I says “look what you have here on the side”, this is the same as this, and he looked at it and I go “See”, and he goes “No”, and I says “We need for them both to be the same, we don’t need the red, and the only way we can get the red out is to give you some medication, and the best way is through you vein here–see this vein here?”. All of that I wanted a professional to help me with, he gave us a hard time–but finally, he stayed three days in the hospital and it went away.
17. **I: Mhm.**
18. F: But he was, he was three.
19. **I: Right. So you’re saying that they didn't, uh, they didn’t take the time to explain it at his level–given that he was three and he has down syndrome–even though...**
20. F: No.
21. **I: So do you think that was just a lack of–of knowledge on their part–or, or, they didn’t take the time? If you had to–if you have a sense at all, what do you think was behind why they didn’t approach it in that way?**
22. F: My experience as a supervisor, I worked at a mental health center for many years as a supervisor–I think training.
23. **I: Mhm. So lack of training**
24. F: Having the–when you’re gonna train the people to work with children, tell them they’re not all typical children, they’re all not the same–you might get somebody that might not understand. And he is one who would have understood–I’m wondering if they would have had some child that was on the lower level intellectually than him, I mean, they screen they have to, so I think now there is a little bit more awareness on this type of thing but we’re talking about 28 years ago–okay, I think there’s more awareness now, it’s more upfront, down syndrome and autistic children and that kind of stuff, but those two experiences were not nice
25. **I: So do you feel that–when you say “lack of training”, do you think it’s lack of training specific to different types of disabilities more so than a general, how to interact with patients?**
26. F: Yes–exactly, yeah.
27. **I: Okay. So going back to the–the first example, what uh, you know, so you think it was just the x-ray and the inflammation wasn’t clear, wasn’t really anything specifically related to having a disability or anything like that?**
28. F: I don’t know. No. I don’t think so.
29. **I: Okay**
30. F: No I don’t think so–maybe, you know, you always have in the back of your head, and I don’t wanna say the “chip on your shoulder”, but maybe they thought “this kid with down syndrome riding bike and doing wheelies...uh..maybe he just fell in the park, it’s nothing”
31. **I: Okay so you think maybe discounting it because–**
32. F: I’m maybe judging
33. **I: Sure–but it’s a thought in your mind?**
34. F: It was a thought it my mind the next day, I said “wait a minute” (laughs)
35. **I: That is it was kind of dismissive given that information?**
36. F: Yeah, maybe, I don’t know.
37. **I: And do you think it would be dismissive in the sense of, you know, judging based on knowing he has down syndrome, or maybe judging in the sense that “Oh a caregiver of a child with down syndrome might be more cautious or sensitive in seeking help”? Any thoughts about which one–or, or...**
38. F: No, just maybe that, um, maybe he just fell, maybe–I thought these people don’t believe that he could ride a bike at eight years old
39. **I: So some of the assumptions about capabilities**
40. F: Yeah, yeah.
41. **I: Okay. So in thinking about other things–other interactions that may not have been ideal, not necessarily full on bad, are there any other things that come up in terms of–it could be how they communicate, whether there’s assumptions, they physical environment or equipment being appropriate, procedures, anything that comes to mind?**
42. F: No, and I’ve been thinking about it a little bit, I am–I was very lucky to have a good pediatrician for him–well, his first pediatrician was fired on the first day–on the day that he was born. He had been my pediatrician–my oldest daughter was 15, I have four before him from my first marriage, then I have him, and then I have a daughter after him, 20/22 months. Um, he had been the pediatrician since my daughter was born and she was 15 at the time, so he comes in the room the next day after I had him, and I had no idea that he had down syndrome throughout the pregnancy. My alpha fetoprotein was very high, but nobody did anything about it, not even telling me get a [inaudible] in the delivery room. He was born very quick–I saw it in his eyes, I knew he had down syndrome, and the pediatrician comes in and says “We’re gonna do some tests, I’m still not sure, his ear are not the height that they are supposed to be, it’s okay, I don’t see anything in his heart, I cannot detect anything at this time, uhm, but you have to realize that he’s not gonna walk for a long time, he might not even talk. You have a child that is mentally retarded”. I had a child a few hour ago, and I sat on the bed–they had these phones on the nightstand, these old phones on the nightstand, I grabbed the phone, and I said to him “If you think that chromosome is gonna scare me you don’t know *me* yet in 15 years–you get out of here because I’m gonna hurt you”. I ran out–I ran to the nursery, and I says “You can’t see my child anymore. *No!*”
43. **I: So you did the firing?**
44. F: Mhm. So yeah, I called the doctor that delivered and they recommended a doctor that was his doctor until he was 18, and then I transferred him to my PCP–very good doctor, he talks to him nicely, he understands, uhm, he does the down syndrome walk, so this is a very sensitive doctor, and he’s a young guy–and when I say young I mean he’s in his 40s–to me that’s young and uhm, he had a couple of problems that he needed specialists so he said “I’m gonna send him to my friend”–the way he talks to him, the way he treats him, my son adores him, so that sense–and he hasn’t had any hospitalization, thank God since then, but we had to go to the emergency room one time, we waited forever but, uhm, that’s when they discovered that he had a heart problem when he was 25 years old, which is something that I think should have been discovered before–uhm, this was about seven years ago, and it's not a problem that's embarrassing in any way, but his veins and his arteries are on the wrong side, and his vena cava is missing or something, there's something in the plumbing, but they didn't discover it until he was, no he must have been about 22, he's 32 now–31. Uhm, so they discovered that because they did an ultrasound of his stomach because he was having stomach pains and this, that and the other–so–
45. **I: That's how they found out?**
46. F: Yeah. They said–ah–let's take an x-ray–and they said "ahh" so cardiologist came and he says "I need to see him, I need to do an echo"
47. **I: So it wasn't something that would have been picked up in a routine medical appointment, there had to be imaging?**
48. F: No. In my opinion, he should have been tested for heart troubles, which is very common with Down Syndrome kids, uhm, his pediatrician did follow him with calcium because he's what do you call, his fontanelle?
49. **I**: Yeah.
50. F: It was open until he was like about a year old.
51. **I**: Okay.
52. F: Lack of calcium so he put a new calcium in there, but uhm, not his heart but, and then he wanted to do special olympics, he needed to have x-rays of his neck, and he does have a plantoaxial instability, which means they're not all–like this one is like this–but it seems like it's something new...
53. **I: Something that has recently developed new?**
54. F: Yeah because he had his x-rays for special olympics about 8 or 9 years ago, and he came out okay, and now, so we want to a neurosurgeon, wonderful guy, and the doctor also explained to us, "look I'm gonna send you to a doctor, there's been a little problem with your x-rays that we want to know–make sure that you can play, he cannot do uhm, contact sports"
55. **I: Too–too risky?**
56. F: Yeah. Too risky. He can do swimming, which he does, he can do basketball as long as he use dribbles, like that, not contact.
57. **I: No defense?**
58. F: No, not at all. And, uhm, he can do bowling [inaudible] so, he can do all that, and he goes with his companion to the basketball at the park by himself, a little bit and, he's good, but uhm, what else?
59. **I: So...so...you talked about him having a heart problem identified around 22, and thinking that should have been checked earlier, given the, the, know that that can be a problem with people with down syndrome?**
60. F: Yes. It should have been checked earlier because the–when I took him to the cardiologist he said "This could have presented a problem". Some of his organs–because his plumbing is twisted, it's on the wrong side–the the right is on the left side, and the left–some of his organs could be also, in a different place, especially his appendix. You take him to the hospital with pain on his left side, and he said it happened in Colorado, and one of his professors had the story where this child had the same problem, with the pain on the left side, and the pain on the left side and it was a ruptured appendix, he died. Why? Because his appendix was not the other side. And I said "these–nothing's happened, thank God everything is good, but we need to do uhm, better they did an abdominal scan to see that everything was in place, that–
61. **I: So that they know where–what is-**
62. F: Yes. Where everything was. And he did tell me it should have been done before–it should have been done before because you never know.
63. **I: And do you have any thoughts about why it wasn't been done before? Like do you think it's lack of knowledge about down syndrome and the risk?**
64. F: I think he was such healthy kid, and maybe another side, I don't know, and me, it's my first down syndrome child, so I maybe denial–I didn't wanna know? Or maybe cus I have friends that have their children surgeries down syndrome kids that have had surgery about a month or three months, my good friend has a daughter with down syndrome and I was in the hospital, I went to see the cardiologist with her and they said "I'm gonna put it in the machine, I have to do everything in 50 minutes, then I'm gonna get her heart and I'm gonna put it in here", and I was like [inaudible], and uhm, I know a lot of kids, his good friend has a scar in his chest, but since nobody said anything they said "No, he's okay, his heart is okay, the echo is okay" they didn't wanna go further, I don't know...we were lucky [inaudible]. He goes to the cardiologist every year, every two years, and every three years they do a an echo and it's good. Everything is good.
65. **I: Okay. So I wanna go back to the doctor that you fired after the birth if you don't mind. So, so, obviously he was giving you information that he probably thought you wanted to know or needed to know, how could he have or should he have handled that information sharing differently so that it would have been a positive experience that didn't get him fired?**
66. F: He probably has three [inaudible] 21, he probably has down syndrome, and tell me what the other doctor said, and I said "Now what". He said three words: school, school, school. That's the first thing, and he could have told me "I know you've raised four kids, and you're not new to this" though I was, uhm, but uhm, let's see what we can do, I'm gonna tell the nurse to find you a support group which there wasn't, I started the Down Syndrome Association in 1995 in Miami. Okay, so let's see if, uhm, we can find, uhm, a support group, or maybe you gonna have a little bit of problem with his feeding but maybe we can help you, or, something like that, uhm, maybe we can get some information from New York, he should have known better, his daughter had–his granddaughter had down syndrome.
67. **I: Oh wow. Okay.**
68. F: And he was raising her because when it was time to take her home, mom said "I'm gonna go to California, you do with her whatever you want", he was raising her. So I don't know.
69. **I: Do you think he was using his personal experience and saying this is what you can expect because this is what I've seen or experienced maybe?**
70. F: I don't know, but I didn't like it. At all.
71. **I: So it sounds like, if I had to kind of summarize the difference that you're suggesting, it's not telling you what's going to happen in the most negative terms, just kind of saying "Let's see how it goes, here are some options and resources"**
72. F: Yes! Give me some hope–I just had a baby, he was born at 4 o'clock in the afternoon, he comes at 8 o'clock in the morning, I mean 15 hours later he's telling me all these things about *my* child, I know, and then I went into the most unbelievable denial of all my life, I have never, never encountered such denial, uhm, I have a masters in Psychology and I know all these cells were effected, I mean I went to school, I mean I have my little education there to work with, and I said "it's gonna be a miracle, it's gonna be okay in a couple months when the results come back".
73. **I: Like nothing's gonna show up**
74. F: One day I'm gonna wake up, and I'm gonna look at him, and I'm gonna go "Whoops! It's gone!" And I didn't tell my husband, I didn't tell anybody, for two months.
75. **I: That was maybe in the hope that things would shake out differently?**
76. F: Yes I would just have him next to me, I would play the piano, I would do things, but then, a friend of mine came to see me, and she told me, "Are you sure?", and I says "No I'm not", so she calls this other friend, and I didn't wanna talk to her–her son, mine, is gonna be okay. When I get the results from Mayo Clinic, with the all the chromosomes and there's three on the 21.
77. **I: And it was real.**
78. F: It was real, it was bad, I went through the five stages, and there was I bargained with God, I did everything I had to do, and it was bad, but then I just, I went to the uhm, [inaudible], the [local child development center] and, that's how it all started for him to, I mean, he's a model.
79. **I: I was gonna say that, it looks like a very glamorous shot!**
80. F: Yeah. For him to do everything he's doing, I'm taking this to my friend and I'm [inaudible], uhm, he's really good, and that's when everything started to work for him, but the first two months–and then these other doctor, pediatrician, I told him, and he says "you know, it was good that you did that, you took time for you heal from giving birth, you got your strength back, everything's working good", so.
81. **I: So yeah, so when they recommended the, the new doctor that you said is, is great, why did they recommend this doctor? Does this doctor have experience with down syndrome or, or training?**
82. F: No. I think it was, my–my, obstetrician thought very highly of him, yeah.
83. **I: Just in, what respect? Did anything stand out in particular?**
84. F: That he was a good doctor, he worked with families a lot, and he was very caring, respectful with everything, and yes, yes he was good.
85. **I: Okay. So more of the, good bedside manner?**
86. F: Yes, definitely.
87. **I: So would you say that, you know healthcare providers, doctors, nurses, whoever, need more t5raining about disability or do you think that it's sensitivity training? Or what do you think they need to kind of help, like that first doctor who got fired, what does that doctor need?**
88. F: He needs all of that! All of the above! He needs sensitivity–they need sensitivity training, they need to know, and they need to be aware that the parents have a child who has a disability and they want him to be treated like he's any other kid in the emergency room or anywhere else, and now that he's older, explaining to him, okay, and asking him, and making him comfortable with either a procedure, even, even people when they take out his blood at the lab, which they do at the doctor's office, even explaining to him the little details makes him feel more at ease you know. We go to an ophthalmologist whose been my ophthalmologist forever too, and he's also good with him. He's very good with him.
89. **I: So good in the sense of explaining...**
90. F: Explaining, talking to him, even though I have, when he was 18 I went support to get the guardianship, and medical decisions and that kind of stuff for him, I do, but still I like for them to ask him, and sometimes they ask him, "Now we're gonna dilate your eyes and it's gonna be a little uncomfortable, are you okay with it?", and he will look at me, and I said "yeah, it's okay", so he wants me to give the okay sometimes because he know about the guardianship and everything so...
91. **I: And do you usually accompany him to his appointments?**
92. F: Yes. Yes, definitely.
93. **I: And have you found, you're telling me that these are good experiences with the people that you work with, do you find that in your presence they direct their attention at him, or to you more often?**
94. F: I think at me more often, but at him too.
95. **I: But in an appropriate, like in a comfortable, do you feel like they're not neglecting him, that they're including him in his healthcare?**
96. F: They're including him, yes.
97. **I: Okay.**
98. F: They better, because if not I'm getting out of there.
99. **I: Okay!**
100. F: The dentist too, well actually the dentist he goes by himself, because I have a fear of dentists, and I don't want him to. I can stay outside, and I say "Okay, it's time to do your cleaning", and he goes inside and they tell him, and he's there by himself. So, that's a good one too.
101. **I: And being the guardian do you–have you ever done anything proactively ahead of an appointment to kind of prepare someone new perhaps about "Hey, my son is coming into see you, just so you now he has down syndrome, and that means that he might need this or that done differently".**
102. F: The dentist I did. The dentist–
103. **I: So tell me about that.**
104. F: Well I called at night and they asked me, and uhm, when I gave them the birth date, and they asked for insurance and I said simply Medicare, and the lady goes "Okay. Explain that to me again. He's 29 years old, he has Medicare?" I said yes, he piggybacks on me because he has a disability. "Oh okay, he has down syndrome, he doesn't like the dentist but he's not afraid of going to the dentist", so we talked a little bit, and when I went to the first time the appointment for the doctor that takes insurance, I introduced them, I said this is [son's name] "Oh Hi [son] how are you?", so they would see him, and they'd be aware, and they would know that was okay. But I did that with the dentist.
105. **I: So you haven't felt the need to do otherwise for any doctors or?**
106. F: I'm gonna, after my lunch today I'm gonna go to physical therapy, that he was recommended by the doctor to strengthen his muscles in the shoulder and the neck, and I'm gonna tell them. I'm gonna, you know, so that they know.
107. **I: What are your expectations when you tell them that like what are you, why do you want them to know or what do you hope for them to get out of knowing?**
108. F: Sensitivity so that they know, like they're not like, "Oh my God, he has Down syndrome, what are we gonna do with him now?". So that they know, and they say okay, whose gonna do the evaluation, and I'm gonna tell that person he has down syndrome, and you're gonna ask him a lot of questions, does it hurt? Does it hurt? And sometimes he goes–do you want me to be with you? I'm gonna go talk with them now [inaudible]
109. **I: Do you have a sense of uhm, let me ask, when you tell them that he has down syndrome, do they react in any specific way? You know, facial features, emotions, or do they ask any follow up questions about how they may continue their care after knowing that information?**
110. F: The, the, dentist asked "Can he talk?", and I said "More than you and I"–and I says "Yes he can". She asked me "I don't know what these people are gonna ask you, maybe they'll ask you the same thing, can he talk?
111. **I: Or understand, or maybe they'r trying to gauge level of function?**
112. F: Yeah, probably.
113. **I: And do you feel like once they know that information that they treat him differently in any way?**
114. F: No. No. So far, Down Syndrome has evolved and it has changed so much in the last 30 years.
115. **I: As far as our understanding of it?**
116. F: Yes, yes and acceptance. Still kids look at him places, you know, and I'm like, but not as much with the professionals as it used to be.
117. **I: So, you know, some of the work that we're doing is trying to improve care for individuals with disabilities–we know from the literature that there's this disparity where they're less satisfied, more health issues as result not getting the care that they need and all that, so it begs the question of where do we begin to fix this problem that we know exists? And we talked about, well you can't fix something that you aren't measuring, so we'r talking about, would it be appropriate to ask about someone's disability status whether it be in the electronic medical record, or in a patient registration intake form, so what are your thoughts? Would it be acceptable, to you, to ask that in some fashion when you go to an appointment?**
118. F: Like you've had this problem and this problem and then you say see yes or no? Kind of like you having a disability? I would say so.
119. **I: Okay so you're comfortable, you have no concerns about asking that question?**
120. F: No because when I filled out papers for him, and they ask me the information and they ask other, and I mark it and I put down syndrome. It's not an illness, but it's a condition that needs to be worked with.
121. **I: So it definitely should not be asked in a grouping of illnesses because it is not an illness, so**
122. F: No. It's not.
123. **I: And should that be all there is or should there be follow up based on what you put down? So I guess the question is if we're using that information to improve healthcare experience, what do we do with that information? Should we make certain assumptions about that disability or should we ask you more information about what specifically we should do to tailor our care for you?**
124. F: I think I'm getting what you're asking me...like if a nurse gets a paper and it says that the individual has down syndrome, that nurse should know what to do, in a way, maybe ask–
125. **I: Should be familiar with down syndrome enough to know what they might do differently or what concerns or considerations?**
126. F: Just be aware that they could be, that there's, I don't wanna say what they use for autistic in the spectrum, there's kids that act like my son, some others are more afraid, you know, be aware of that, not that this child is different but that there is a condition that needs to be taken into consideration when you are gonna do a procedure.
127. **I: Right, and there might be some accommodations that are provided?**
128. F: Definitely, definitely. Most definitely.
129. **I: So they should be based on training or their experience, should be aware of what kind of accommodations might be needed?**
130. F: Uh-huh, definitely yes.
131. **I: So I do wanna show you one thing as an example, so these are questions from the US census that are used to access disability status, so they're not really intended for the medical world, but just as a starting point to whether you like these questions, so, so here we have, and I'll read through them, so a question about deafness or hard of hearing, vision or difficult seeing, uhm, this one is asking about issues or serious difficulty concentrating, remembering, or making decisions, uhm, difficulty with mobility, walking or climbing stairs, uh, having difficulty dressing or bathing oneself, and then conditions that would lead to difficulty with doing things on your own, running errands, going to the doctor, going grocery shopping...so do you feel that questions like this would be helpful in getting more in depth?**
132. F: I don't like some of those.
133. **I: Yeah, talk to me, tell me what you like and what you don't like.**
134. F: I would take *that* off.
135. **I: Take off the are you deaf?**
136. F: Are you blind? Are you deaf? Are you blind? No! Do you have serious difficulty hearing or do you have difficulty hearing?
137. **I: So just keep it at that. Tell me why you want to omit that, what are your thoughts?**
138. F: I would feel, I don't wanna say the word, should I?
139. **I: For, just so I–I correctly understand you-**
140. F: Do you have difficulties hearing? Okay. If the person has difficulty hearing is obviously gonna have a hearing aid.
141. **I: Or would you use different language so are you unable to hear would be better than saying are you deaf are you saying?**
142. F: Yes. Do you have difficulty hearing? Or, can you tell me about the hearing aide? Why are you wearing a hearing aid? That would be a lot softer. This is like..
143. **I: So soften the language?**
144. F: Yes definitely. Do you have difficulty concentrating? Here too. Now do you have–why does this say serious? Do you have difficulty walking or climbing the stairs. "Serious" is like.
145. **I: You have to decide how you define "serious", so you're saying that's too subjective?**
146. F: Yeah. Do you have difficulty dressing or bathing? That's good. And six is good. See five and six are different. But this, especially the first two..
147. **I: Okay, okay. So if we soften the language..**
148. F: If they had to ask my son, and he would have difficult–hearing difficulty because I would take him to uh hearing task, I wanted to ask him uh, what kind of difficulty or what kind of problems do you have with hearing.
149. **I: Okay.**
150. F: So one day he comes and he says "Mom, I can't smell", I put my perfume in the morning and I can't smell it, and I couldn't smell the shampoo. So I went and I got [inaudible] and I said close your eyes "What's this [son]", [Son]!", and I says "Let me call the doctor and have an appointment, so the next two mornings after that, he's sitting on the sofa, his back is to the kitchen, and the granddaughter wants rice for lunch, she calls me the night before "I want rice for lunch tomorrow I don't wanna eat school lunch", "Okay I'll make you some rice", I get this little pan, I put rice, water, salt, and whatever and I put it to cook. My husband calls me in the room, I'm talking to my husband and I'm used to doing rice, I'm 72 years old, the rice is cooking, there's no more water, the thing starts burning, the kitchen is full of smoke, and my son is there and he cannot smell the smoke. And I go "uh-uh" , now we're in a problem. So I called the doctor, we had and appointment, he checked him, and he says "[son] I'm gonna send you to a doctor that has your same name, I went to school with him", "What's his name?", he goes "[Name]?". "Yes. I'm gonna send. So you go see him, over by [inaudible]", and he says "Tell me what's going on", "I can't smell", "What do you mean you can't smell", and so he had to do, he was outpatient, he had some membranes or something there and he had to clean it up and he did and now he can smell, but the way he talked to him, I loved it, he was not do you have difficulty smelling or seeing is difficult, tell me what's–
151. **I: Or you tell me what's going on?**
152. F: Yeah. What's going on? That's why you're here. He says, because my doctors says that I have to see you because I can't smell. Some of them would approach different, like "So your mom says you can't smell? Alright, okay" [inaudible] or something like that. And he told him "I'm gonna tell mom to do a test, she's gonna blindfold you and she's gonna, you're gonna try three different things and she's gonna give you to taste", so I gave him soy sauce, sugar, and I don't remember what's the other one I did. "Close your eyes, she's gonna put a little bit and you're gonna tell her what it is". And he did, because he said if you cannot smell or taste then we need to go a little bit further. But he could do it. But he told him what I was gonna do so I was like [inaudible], but here, make it a little softer.
153. **I: Same for two and three a little bit?**
154. F: Yeah.
155. **I: So, so would you still recommend asking something more in depth or would you rather you know, when you see someone wrote down syndrome on a patient form, would you rather you say "I see that you wrote down down syndrome, tell me more, tell me what that means, should I be doing anything differently in terms of how I provide care to you? Or would you prefer questions more specific–do you prefer the more specific questions?**
156. F: More specific questions.
157. **I: Okay, okay. And how often should these questions be asked? Every time someone comes in or only the first time?**
158. F: Well, if I ask you do you have any difficulty hearing? [Inaudible], then I could say is your hearing better?
159. **I: So only if something has changed you follow up**
160. F: Yes for followup, but not the same question.
161. **I: Okay, okay, alright, do you find, you know, when you have had longterm interactions with all of the doctors that you'v had that you like, do you find that you need to continuously remind or explain anything about [son] having down syndrome or they're good, they have it in their record…**
162. F: They're good. They're good. They know him and its good.
163. **I: Okay. In that sense, can you think of anything you wanna share in terms of things that haven't gone right or haven't gone well, or thing's that you're like, you know, this is how it should be. These are the best practices in my book and this is what I would recommend healthcare providers do.**
164. F: My alphafetoprotein was very high, and nobody did anything about it. They should at least have told me have a neonatologist in the delivery room. Nothing. Granted, I was 41 years old, but still, that would have put a red flag on that alphafetoprotein high. And when it comes back and it's really high and I ask the doctor "What does that mean?". "No, it's a little hight", but it wasn't a little high, it was a lot high.
165. **I: So they should have know–or that is a known kind of screening factor?**
166. F: Yes. A red light, let's have a neonatologist in the delivery room. When my daughter was born there was one because I called them. And uh, that's one thing, and what was the question?
167. **I: Any, any, if we were trying to fix issues, what are your best practice recommendations, or what would you suggest needs to change so that we give better care overall for anyone with a disability?**
168. F: I guess just being sensitive to the person, and not trying to make that person feel different. You know, like "Okay we're gonna do this to you because–we're gonna try to make you feel comfortable because you might not feel good about this or, we're gonna do a CT scan, or we're gonna try to make you feel good", if that's what you tell everybody, tell the person with down syndrome, do not make any distinctions, and um, when they're a little older I think this should be addressed as to, alright, so you can't smell, well we're gonna see what we can do about it, and like my doctor said I'm gonna take you to the doctors for an ENT he's a specialist, and you know, instead of telling me let's send him to an ENT because we need to take a look at this.
169. **I: So explain and be very clear?**
170. F: Yeah, and also if they, they also have to be aware of body language
171. **I: Okay tell me about that, body language.**
172. F: They have to be aware of like, when–
173. **I: Their body language?**
174. F: Oh yeah, definitely. If their eyes water because they're scared, or if they're cold, if their hands are cold, if they're kind of distant from the doctor, or they're kind of pulling into the mother…
175. **I: Oh so you're saying [Son's name], or the patient's body language?**
176. F: Yes, the patient's body language just feel a little bit for it because they're telling him we're gonna do this and that and all of the sudden they kind of like wanna cry but they don't because they're big and they're not supposed to in front of the doctor you need to know that.
177. **I: Pick up on those cues.**
178. F: Yes. Oh yes. Those are cues that you need to pick up on all of that.
179. **I: Are there any uhm, cues, their own personal, the healthcare providers cues or body language that you think need to be different or do you think that's fine?**
180. F: Yeah if they have good bedside manners they know, you know, like to "Hey, [son's name]", and give me five, and that kind of stuff.
181. **I: So building–doing the relationship building.**
182. F: Yes very important.
183. **I: Well thank you, those are all my questions!**
184. F: That's it?
185. **I: Yeah!**
