# Supplementary material for: Disability disclosure in healthcare settings for individuals with developmental disabilities: A qualitative study of patient and caregiver perspectives
Source: PLoS One. 2025 Aug 7;20(8):e0329328. doi: 10.1371/journal.pone.0329328 (PMC12331114; doi:10.1371/journal.pone.0329328)
Supplement: S1 File — (ZIP) [file pone.0329328.s001.zip › Transcripts/2019.12.12 Interview 15 Trancript.docx]

1. **I: Alright, so we’re good to go there. So, so you told me over the phone that you’re a caregiver, can you tell me a little bit more about that?**
2. F: Yes. My daughter, um she's 17 now, single parent for the bulk of her life since she was 18 months, uhm so all responsibilities have been on me as far as her care from day one, um it has not been an easy journey because we know with time things have changed. But early on in her life um a lot of things weren't available that may be available now. So, I kinda feel like we've been in the loophole not really getting the attention that we should've gotten early on that really could've made a difference.
3. **I: Um...And what condition or conditions that she have?**
4. F: Found out later on visually impaired and is intellectual–is cognitive. I don't know if it was due to the birth or if now that she's getting older and we just weren't given a formal diagnosis that time has went on and it has progressed to this state. So, I'm still kind of trying to find the way is who, what, when, where, why and how. Yeah.
5. **I: Right. So, okay, so you kind of said that you haven't had the best experiences– maybe it's different now, but tell me about that. What hasn't gone...**
6. F: To me it was birth trauma.
7. **I: Okay.**
8. F: So, from there, not knowing, not getting the answers–you ask questions, but you weren't getting the answers. Um...So, you kind of just let time go by and you wait and you see, then you start asking more questions. It's like it was a hands-off thing. Nobody wanted to really touch it per se. I left one stage, she was born in Nashville. I left there and moved to Miami. We're back here–this is where I grew up. But coming back here, it was almost like we moved to, I don't know, a foreign country because (illegible) want birth records? I got them what I had–or what I could get, but still nothing. Nobody formally diagnosed her. It wasn't until third grade that the school system decided to do what they call, I guess this uhmmm what do you call it? It's an apt- test–IQ test. So, based on that they said she has developmental delay.
9. **I: Was that the first kind of quote unquote diagnosis that you got?**
10. F: Yeah, I felt sideways kind of cause I felt like how can you tell somebody’s IQ when they really haven’t been given a fair chance. And you guys are not doctors by far, you're educators. So, I just feel like from there we started the whole gamut of trying to figure it out–with placements and schooling–they kind of just get nowhere. They just do a little bit to keep it quiet. So that's kind of what we've been going through that. And then I've been the parent that always keeps appointments. So, it's when I go to the appointment and it's something new or some kind of new something I'm asking doc, why? Why is this? And everybody just said, same thing. Oh, don't worry, don't worry. She'll get, she'll grow out of it as she'll get older.
11. **I: So they didn't think that it was a permanent thing. They just kind of, they just kind of come down the road and said “Well Oh–**
12. F: When my mom's there [00:03:33 overlap] My mom said early on something's not right. Then my mom passed away from cancer. So that was part of the reason why I moved back here and um just nothing, nothing just everybody–even now I feel like if I just touched it, they just stayed away from it and they just kind of fell up under the school and we've been from day one to the pediatrician and two groups of pediatricians, the same group, but nobody did. Later they started to give me referrals to like speech, OT, the things then lead to physical therapy.
13. **I: When did that start? She's 17 now, so how long before they they gave your referrals or...** .
14. F: By five she was getting–supposed to be getting some OT, I want to say about five but more so maybe by the time she got in second grade it was two sessions a week, 30 minutes. When you're talking about not just her, this is through the school as well. Nothing on the outside. Uhm, speech. She got some speech. Same thing between second grade-ish, first, first to third grade, but after a while, either because of insurance or something, you only allowed so many visits with private insurance through their employer and I think as I maxed that out, then it was letting me and then they felt like um she’s making progress–there’s a difference between I learned late on speech and language is two different things, so I don't know how what they determine the need for both because she could articulate so to speak because of that. She didn't get it either way. Then I tried to revisit it later and because of age, a lot of people kind of just said, “well she talks okay”, but she doesn't, she has a speech impediment but nothing. So, every year maybe every couple of years would go by. I would try to re-approach it going back to the same place because that's what I was familiar with, but really no direction from any, I guess trusted physicians per se.
15. **I: And that assistance that you did get, did they freely recommend it or did you have to kind of push to get that support? For OT?**
16. F: I think I had to push; nothing has been freely recommended. Right. Then now maybe I say with time I can see maybe some areas or some places are getting better because more people are speaking out I should say. But initially, no.
17. **I: Right. Do you feel like you're not getting the assistance that you need because they are not familiar with what her conditions are or what do you, what do you think is behind them not being more helpful?**
18. F: I wish I knew. I’m baffled myself by what is it? Um...Is it that complex that nobody's really per se touched it or is it because of time and they kind of just throw the towel in because of the age group? But my angle has always been it's never too late. It's never too late and the mind is powerful– the brain is a powerful thing. We just have to figure out: who, what, when, where and go from there. Um...Whatever strategies you have. I just say with anybody you can learn, you can learn. So, I don't know. I say with science and technology, medicine is always improving. I just still feel like there's a chance, there's a chance. Even in school, even at doctor's offices, but like I said, now that she's a teenager and people kind of just feel like they’re set in stone. But you're not–you’re still young to me. You’re 17 years old but still young.
19. **I: When um when she goes to the doctor, do you go with her?**
20. F: Oh yeah.
21. **I: Okay. And so, can you talk a little bit about what that experience is like? I mean how they interact with her or how they treat her, how they treat you?**
22. F: Early on, I don't think they understood cause I don't know if sometimes doctors hands are tied and they don't want to put labels on children. So maybe they saw different things from the pediatrician standpoint. But once again, they still they still led me to believe like maybe she'll grow out of it, or they said if you want a referral to here, we can give you a referral. But nobody really said “I think you should take her there”. You know, they kind of like left it up to you to decide what's best for your child. But I'm saying you’re the doctor, you're the expert. You're supposed to know, I would think more than me, but they didn't have, I never saw that happen and I was shocked. But what do you do? You just keep hoping and praying I guess that the light comes from some different angle and I'm always one for reading a lot of books. So, I think that's what helped me get through a lot was just reading. And I always say informational knowledge is power. So, if I can put together this and say, well maybe if I tried this or if I asked this, cause sometimes as parents you don't know what to ask. You don't know. You can give situations or symptoms but if you don't know, then you don't know. So, I found myself kind of in that.
23. **I: Just kind of on you to educate yourself or ask the right questions and get more information.**
24. F: ‘Cus if you don't ask, nobody says. Even with her vision, she was born, uhm, cross eyed–uhm, slightly lazy eyed. There was a phenomenal doctor out of New York. She since has gone back as I was told, but she right away at two, two years old, they did the eye surgery. So, it wasn't 100%, but it corrected the vision to a great degree. Um...It was just hard for me then after that to keep continuity of care of, I don't know if it's due to the insurance, we had the same insurance, but I don't know, it just kind of gave me like it was a cure all thing, it was done, but it really wasn't because now that she's older, we're still seeing the same doctor from maybe third, fourth grade, and the eyes made a different turn. She doesn't have the cross eyes anymore, but it was almost like they were still trying to figure out what's going on. Now she has a degenerative eye disease now, so I'm thinking, wow, they couldn't have caught this earlier? It didn't tell me if they need to do the surgery again for the call side. It would be in a decade, so decades. That would've been 12 but when I visited it at 12, they said, “Oh no, her eyes are good, good.” But now they told me that she has this innovation that she could wake up one day and be blind. So that for me was shocking. So forgive me if I…[voice becomes shakey]
25. **I: No, no, no, please. Sorry to be bringing up old memories or feelings.**
26. F: [pause to recollect emotions] So, it's, I don't know, I'm still trying to figure this thing out. How, how people cannot provide services for children. They're innocent. They have no voice. And if you the parent don't know, that's why have so many kids falling through the loop. Because we know that early intervention is the key, the key. And you have to, as well as the physician or anybody, you have to educate the people that’s in their circle. So, I'm thinking now that so many people spent so much time with her–It wasn’t educated. They rely on the parents to give a lot of feedback and I can only tell them what's happening at home, but that may not be the case at school. And I know that labels are hard because once you get a label it pretty much sticks. So, it was like a catch 22; your hands are tied.
27. **I: Right. They're trying to hold back a label, but at the same time they need to be giving you information.**
28. F: Yeah. Yeah. Cause if you don't get some piece of it, how can you fix, or what, which way do you go? So, I feel like for me, that’s, it's just been all over the map. I can only approach it from a spiritual point saying that God don't make mistakes and everybody has a purpose.
29. **I: So, is she is she able to make any medical on on her behalf or do you make those for her?**
30. F: I make them.
31. **I: Okay. And how would you say she's treated by her doctor or other healthcare providers?**
32. F: Pediatrician wise, I think the female doctor does a pretty good job of trying to include her, asking her for feedback. And she’s been so dependent on me, I guess per se. I'm truly trying to teach her to talk for herself more. And you answer, they're talking to you, you answer the questions cause they’re speaking to you about you, children learn what they live. We as a family have tried to say we won't limit her. We don't, we didn't see the disability. We saw the ability; so, she doesn't know or she didn't act like her thing was, I'm just like you. I’m just like me. I don't have whatever, but you do. So as she gets older we are trained now or people are saying you have to talk more about her disability, but I'm still saying no, we got to talk about her ability not the disability and I feel like because of that they don't really include her in lot of things.
33. **I: They go straight to you rather than trying with her first you’re saying? [00:13:05 overlap].**
34. F: Yeah, yeah, yeah. Her eye specialist, they don't, they don't even, everything is to me and I'm saying you should be asking her cause it’s her eyes-
35. **I: When you take that opportunity to kind of encourage her and in front of them, hopefully them seeing that as well, do they kind of try to redirect their attention and then give her the direct attention?**
36. F: Yeah, yeah, yeah.
37. **I: Okay...And is it, do they kind of get the message?**
38. F: More so now. Now within the last year, I want to say, she’s 16, I'm starting to see more of that, but before everything was really–only maybe once a psychologist was visiting the doctor for whatever reason. I guess they started targeting the teenagers as far as drugs, alcohol, sex and all that. So, they asked me to step out, but I felt kinda, I don't know. So, I stepped down just to see how it would go. And then I think whatever questions they asked her, cause I was like in my house is really no–my teenagers, they’re pretty open with me. There's really no secrets. We talk all the time. So, they are the type of kids they want mom around. They don't want me to not be there. So they immediately call me back in and then I think they asked me one or two of the questions that they ask her with that kind of shit me because I was like, if you had read her files, she has an intellectual disability, she’s cognitively degrade, so I need to be here. But I slipped out just because she was the physician in charge and I don't know what the goal was.
39. **I: It was supposed to be like privacy questions or something.**
40. F: Yeah, I don't even think she understood to what degree the lady she did because sometimes even with our special needs children they understand a lot more than we give them credit for and they may be slow in one area, most of the time it’s academics, but the other areas latch up with any peer side by side. So I said, as they often say, children say the darndest things and they will surprise you. So I didn't ask what she said, what her response was, but I'm sure.
41. **I: And do you find when you, when you are a present and they are interacting directly with her, that they use language or speak to her in a way that that she can understand and interact or do you feel like they have difficulty kind of meeting her on her level or what are your thoughts there?**
42. F: Uhm...Both, both. It just depends. It's both. The pediatrician, she's pretty good. She's a female, so she's pretty good. The ophthalmologist, I'll tell him to bill both meals.
43. **I: Do you think there's a gender difference there?**
44. F: Yeah. Yeah.
45. **I: Tell me more about your thoughts on that.**
46. F: They are, I don't know, you call it old school insensitive or that’s just their persona, but
47. **I: Is it more than just gender. Would you say? Is there an age thing too if you’re talking about old school? [00:16:08 overlap]**
48. F: Yeah, yeah. Yeah.
49. **I: Okay. So he is older than the female physician?**
50. F: Yeah, yeah.
51. **I: Okay**
52. F: Much older. And they've been around practicing a while. The female–she's probably used to dealing with teens, and...she’s been at that practice quite a while. I don't know how long before we got there, but she's pretty good about getting them to talk about whatever it is. If they have questions and she talks to her and me. But um...Let’s see, the speech pathologists? She was much too young. They would talk to me. Occupational therapy, I can't really say because occupational therapy, she was getting supposedly at school by the time they rounded up her and all the other children keeping the 30 minutes, you're talking about 10 or 15 minutes and if my daughter goes a whole year she can't tell me the name of the person. That says a lot right there. Kind of a time was spent and I never got any feedback on paperwork, especially during middle school nothing and probably after third grade not much as far as them interacting with me or her, so I don’t know what to say other than it being on paper that it took place.
53. **I: Right. It's more like checking a box than actually a quality experience interaction, okay.**
54. F: Because as far as I was concerned, I wanted all those therapies to continue on indefinitely. And I said at least give her the, I think my plan insurance is um 15 visits per discipline per calendar year, which I said is pretty good I thought. It's better than nothing. So, but that's not what happened. It's hard trying to find somebody now that she's older that'll take them, cause they really cater now I think probably to newborn to um-
55. **I: -very young.**
56. F: -yeah. But I think if those therapies are ongoing the progression would have been greater. I think even with the early intervention, you can't just start something at, whatever the age is, birth to two or birth to five and think that it’s a fix, because these kids they need constant, you know? I think if we have more medical professionals pushing, then you might see a change. It can't just be teachers saying that.
57. **I: And healthcare providers too may need to be stepping in. But you're saying, they're not really pushing for it. It's more about you asking about it, okay. Can you think to–you mentioned that you said that the doctor in New York was really, really good and the pediatrician is pretty good. I mean already. Great. Is there anything that they do that stands out that you'd like to share that like this was, this is what makes it a positive experience for you and for her?**
58. F: As far as I can remember maybe because she was younger, I just felt like that doctor stood out because she took action, you know, she saw something and said, you need to do this and it's what I'm going to do, a plan of action, and we did it. And then she even had a projection. She said, and if something should transpire between here and here, then this is what we'll do. But right now we're looking at the 10 year span. We'll revisit this and we'll go. And you can appreciate that. Not just nobody saying anything or doing anything. Um...Like I said, I think because of either my plan or she just moved for whatever personal reasons, her job or whatever, life took her back there. But when I questioned her, um I said, is she still here? Is she still around? I want to continue to see her. And they said no, she moved, she got her own practice or she left the practice and then somebody later said I think she had gone back to New York, I thought, wow, here we are back to ground zero. We have to find someone that's gonna be offer continuity with her care number one. Will pick up where she left off. But I just think she, they, I, I'm hearing now that New York city is a big city, but they’re on boards as far as care for children and adults, teens or whatever they’re just– I've met some people and they've said the same thing. They were just visiting here and they was telling me they was thinking about going back because the board member here and the other members there, but their children are here. They’re kind of like fishing. And they were saying they left a place where children were first. So they think about going back. So that lingered in my mind saying, wow, here we are, my daughter's much older now and I don't know what it is. We're just behind times or I don't know, this was certain care of certain people. I don't like that either. Mind me, you gotta be a certain status for you to know, or to get information or for services. I've always had insurance. Good for me I guess you could say. But even with that, that still didn't give me a leg up on somebody who didn't have any insurance. I don't feel like it did. It's one thing not to have insurance and you're just left hanging.
59. **I: So, there’s access issues.**
60. F: Yeah, you apply for whatever those insurances are for the healthy kid care or whatever those bases on Medicare. That might help too. Cause I used to get that question a lot: do I have Medicare or whether it's called Medicaid and I would always say no, I have private insurance. So, I pay it to myself. I started to tell myself that maybe that was a barrier for me because I had the private insurance, which I thought was fairly good through my employer and very costly. So, I'm thinking why am I paying something that nobody wants to take? Wow. So, I've never been able to answer that cause I still have the same plan and years later, I still have the same plan. And...
61. **I: And no one's ever talked to you in the healthcare setting about eligibility for other other programs or anything that would have increased access to resources.**
62. F: No only Medicare, they only mention if I had it.
63. **I: Only really asked you if you have it so they can document, okay.**
64. F: But nobody ever mentioned and I said to myself, wow, it's not, it's a disadvantage to *me* for working and having insurance than it is for somebody who doesn't work and doesn’t have insurance. I started trying to say, wow, what is it? Why am I? Whatever.
65. **I: Right. So you said when you talked about switching over to a new healthcare provider, so when you go to an undue healthcare provider or a new eye doctor, whoever it might be in the healthcare setting, do you tell them ahead of time any information about your daughter and you feel the need to or what are your thoughts there?**
66. F: I'm in between all that.
67. **I: Okay.**
68. F: Because over time I felt like...it can be helpful and it can be hurtful because I want them to see for themselves and tell me and we go from there. Cause we sometimes when you give too much information then they don’t... they just go off with somebody else says or does, not come up with their own study and say this is what we should do, I understand you've had X, Y, and Z, but I read over your note doc. So, I'm in the middle with that. And I think it's, I don't know if it's better for adults and worse for kids or vice versa, I’m still torn with that because I felt like I shared some information with someone or something else with her and they did nothing. They did nothing.
69. **I: They didn't kind of adjust or accommodate in any way.**
70. F: Yeah. So, I'm in the middle with that. You can, it can be against you or for you. I think if you go in sometime and scope, then you could see what happens from there.
71. **I: Right. So, you're saying the downsides can be they take that information and they do nothing with it or they take that information and they prejudge. They don't, you know, rethink or update according to their interactions. So, what would be the potential upside then?**
72. F: Upside is that, okay, they can say, wow, here's a child that has X, Y, and Z. We've never seen them do this, so we should probably do this, or let's try this. She is similar to another patient that we had, but this might work or that may not work, you know, rather than do nothing cause like no two people of course are alike and they're not responding to the same care. I don't know. That's something that I guess overall, has to be worked on, because even the same thing in the classrooms and the school– I just think more people have to be educated, more people have to be hands on and I don't know. This has gotta be [00:24:59 overlap] Yeah. Especially if you're going to be in the, in the field, you're going to be in healthcare, you're going to be involved with a diverse group of people. You got to study, you got to know this, this, this and no two likes are alike.
73. **I: Absolutely, so given that there seems to be pros and cons of that, would you air on in any direction or just you're still undecided?**
74. F: As she’s getting older, I want to say maybe I should, ‘cus I don’t feel like we've been formally diagnosed with anything. I can only tell them my experiences per se. So, as she's getting older, I'm saying maybe I should just say this so that we can maybe get this or we could be over here in this group of people or should I just stay in the middle and kind of just wander around, [laughs] I don't know. I don't know.
75. **I: Well one of the things that we're talking about, you know, cause there's a lot of literature that talks about disparities or patients with disability relative to patients without. And a lot of that is thought to be attributed to what you're talking about. Lack of training or whatnot. So, you know to fix this problem, one of the things we're exploring is, you know, well you can't fix something you don't understand or you don't measure and if you’ve ever filled out a patient intake form or or medical record, they don't typically ask you about disability status or if you have any accommodations.**
76. F: Exactly. Now it should say that. What I started to do on a lot of her forms that I fill out for her, because usually per calendar year, a lot of offices are on point with updating the face sheet, I guess you could call it, so I started in bold somewhere on there putting disabled child or intellectual disability or cognitive delay or developmental delay. I started kind of putting that somewhere bold because I felt like there was insensitivity. They don't because sometimes you could scan a person and then not look. Everything is not physical. These doctors offices, they are not for some staff standpoint, so I started putting it on there so they all know mom has to be there and she may or may not understand. Think of it. If they had a a clue, they'll know that I found that it didn't always work in every place.
77. **I: Writing down information didn't always work, okay, whether they ignored it or whether they still had insensitivities.**
78. F: Or they have chance or look to [overlap], yeah, yeah
79. **I: Okay. Okay. So, tell me, can you give me any specific examples of that of that insensitivity?**
80. F: It’s hard, it’s hard.
81. **I: Cause I know we talked already about them speaking directly to you and not directly to her. So that's usually one example we hear, so is there anything else that...**
82. F: Well there were times when we go to the eye doctor and you see so many people for the workup, I call it, before they actually see the ophthalmologist or the optometrists and people have to be mindful of what they're saying because she's not deaf, and I'm not deaf. Um...Little kids, all of them, no matter what the diagnosis is, can be, I call them little hoppers. They all over the place just because that's just what kids do. Um...And if they could factor in some other motor gross or some other disability, it may be exasperated. So, with that being said, as a commission, you can't put labels on children. You can't paint them out to be your terror. But I overheard someone saying, so when I got into the physician, I said your staff need to watch what they say because, and he was like, and he agrees. He says because what they said to me about this patient and this patient, even my daughter in another patient and he's like, I don't see the same thing. I say that's what we have to be careful with labels because of what we say. You don't know what kind of day the person was having or what they ate or whatever. I said so, I don't know and we kind of just left on neutral grounds but I had to tell myself, did you want to come back and give it a second try? Or do you want to just find somewhere else? Because they were so insensitive. They'd already given her a label and we are bare for services. You already have given her the label. It's like you know.
83. **I: And what specifically was the label just so I know?**
84. F: They said that she's ADHD. Okay. Yeah cause they said she wasn't sitting still or something of that nature.
85. **I: So. you think they were using it not necessarily in a clinical sense. Just kind of making commentary or making assumptions about her?**
86. F: Yeah and they say see with your eyes, and then you say, wow, first I've heard that.
87. **I: So, at least the doctor did acknowledge that that was probably in poor taste?**
88. F: That was probably the first time. The second time I was somewhere and the doctor said the same thing because the school was missing, she was color blind. Oh, she might be, she's hyper, you know what I’m saying...but she’s a kid, you want kids to, they come to school to learn yes, but they are children, they have to be taught, they have to be reinforced. Teaching starts at home, but like at the school, you guys are the rules. I think we as adults, we all know when the cat's away then the mouse will play. So, kids are no different.
89. **I: Right.**
90. F: And that's when I kind of felt like, okay, now here we go. We want to put a negative attachment to a child and that’s how children become scarred or can be scarred. So, I said, if you say that, let's take her to the doctor. I'm not a doctor, you aren’t either, we'll get to the bottom of it. So, I did. I took her to the neurologist. The neurologist said what people don't know, in my office when you come here before you come to see me in the back, usually I have already observed you out front for them to go on. So, I love that about him. And he said, I don't see any of that. So, I don't know what they saw and what she did different from here. But if she was truly ADHD, she wouldn’t have been sitting there with the game, she missed, she would’ve been jumping around here too. Cause I said, well thank you doc. I don't know what else to say. I took the information back that he got in writing because they had to rule that out because they said that they want to put a harness on her. She’s not sitting down on the bus. She's not doing this, she’s not doing that. So, we’ll clear that up.
91. **I: Mhm...So, in instances like that when you know their perception is that she has something that she doesn't have because she, she's moving around or not. Or- do you ever feel like you have suggestions for them because maybe they're not doing something that she needs to needs to do, get her to sit or whatever they're asking you to do it?**
92. F: That time I feel like I was cut off for lack of a better word, could have the umbilical cord. I thought they didn't want to hear what I had to say.
93. **I: So, you didn't feel like it was falling on deaf ears so you didn't bother?**
94. F: Yeah, yeah. I just knew that at home things that I need to do, cause once again I would pull out books. I said okay, she's not doing this so let me see if I can do it at home to try and do this. And I started, I think it was another, I don't know if it was another advocate cause at that time, she was, the younger she was, I found myself going to a lot of those advocacy needs or parent to parent type meetings. My kid was young and a lot of the parents were there had kids that were teenagers. So, for me it was this information and knowledge and it made me feel better. So, I would just go and sit and I'd say, I'm gonna try that. And if it worked it worked but if it didn't, I’d say, Oh Lord, I don’t know what I'm going to do, but I was just listening to them because I felt like they'd already been in in my shoes and I started this, this tiny thing, set the timer, and it works, set the timer it was your time to do this, it’s your time to do this. And it worked for me. So I felt the more idea that and the more she understand that when you're at school you move around, but you only can move around when the teacher says you can move, but you can move around, when is time and this and that. Because I already knew that there was a cognitive delay, but nobody, at at that time had said anything.
95. **I: Official for you.**
96. F: Yeah. They just wanted to keep the label that she was ADHD. With children, if you don't figure it out early, then they get pushed to the side. There's no learning taking place when they're vegetables- their minds are like sponges, if you don’t soak the sponge, everything that everybody else is learning at a typical age, she’s not. So, the gap is growing. That's why I feel like when she got to third grade, she was nowhere on third grade. She had no idea what I was doing at home with her, she kept digressing. It was to the point where I put her in a one-on-one school and when they told me, every little bit gave me hope. It's not me, it's not them, but we got to figure it out and when I took her out of school cus I ran out of money, I was back to square one and even now they tell me that she needs a lot of one-on-one and I keep saying it's because of gap. Nobody’s taken the time to understand every child, it's not a one fit for all. You can't teach them all the same. You got to provide the right resources the right sources, all of that. Everything that they asked me for, any kind of medical documentation I provided that, and I'm saying because I provide that, why aren't things being done. You ask me for this, I go get it. You said she was color blind? I said I don't know. I take her to the doctor- is she color blind Doc? By far... No, it was more prevalent in boys. Okay Doc, I need something in writing and they would give it to me. So I don't know. I just feel like I've been doing and doing and doing and doing. It got so bad to where she is 17 now listen, she was 13 going on 14, I started to question the whole birth process again cause I never got the answers. I thought everything was connected to that delivery and I consulted a lawyer. Um I didn't get anywhere with it because I didn't know how it goes with it. So, I kind of just got emotional and I let it drop, because there is an attorney still was telling me they didn't handle that kind of case or call this person, that person. I just got overwhelmed and I kind of just said forget it. Forget it. Forget it. I don't know. I don't know, I’ll just put it back in God’s hands. I started looking for answers to this cause I was running into so many roadblocks on how to fix it, how to make it better. So, I don't know. I'm back there now saying okay, here we are. She's getting ready to transition into young adult, she’s leaving high school, and some of these medical gaps, they're not closed. But what do you do? Do you just... let it go- or what? But overall, she's been healthy. She's healthy. That's a good thing. She's healthy and I don’t know, I just wish that more people would just give everybody a fair chance. Fair chance. Whatever fair means just give them a fair chance, no matter what gender, color, creed, none of that, just let me treat this person as a person. If it hopefully doesn't mean giving them a label that I don't know cause I guess labels can be rude. They can be bad. I don’t know.
97. **I: So, would you say overall her quality of care was lesser than someone exactly like her without her conditions? Or what are your thoughts?**
98. F: I feel it was less.
99. **I: Because they weren't able to give you more information than then use that information to link you to other support resources.**
100. F: Mhm. Yeah...Everything was always based off of. It's like I'm just searching, searching, searching. It may be because I don't know, I can articulate, they feel like I don’t need, but I'm saying I need help, I need help. I don’t know. I'm still trying to figure this out. But-
101. **I: And you think they got that sense from you all through this through the questions and everything you kind of directed at them.**
102. F: They feel like, oh she's on the board, she knows this mom knows, no this mom doesn't know. I'm still trying to figure it out. I’m still searching...
103. **I: Or did they take it from the standpoint of, oh you seem well read about it and so you have all the answers and I don't need to tell you anything more.**
104. F: [Laughs] Like no that’s not the case, I don't know nothing. I’m trying to figure it out. As I said I spent countless hours up on the computer reading or trying to type in something and see what it says. It's like, oh okay, maybe this, maybe that?
105. **I: Something new to try. Right. So, so I know you said you're on the fence, you're not exactly sure whether or not to to give the information or not and that's part of what we want to hear from you. But, um, in the event that you were going to share information, I guess the question would be um what exactly, what do you want to share? So you said on the papers you have written disabled child or intellectual disability, up is that the route that you find is most useful?**
106. F: As far as dental, I started saying that because I wanted them to do more, what's the word? Cause you don’t want them to feel sorry for you.
107. **I: I think you want someone to be empathetic or more sensitive you were saying-**
108. F: Yeah, so that was the first place that I started was dental by putting it on there cause at one point I thought they were like, insensitive because even people who don't have disabilities when they start hearing drilling sounds or they get fearful. So, I was trying to give them a heads up like, hey, she’s not that child. You gotta be a little patient or whatever. I could say without I guess making it a label per se. You just have to be *mindful* that everybody's different.
109. **I: Right. So, so that's what you've done in the past. So, I guess I'll, I'll give you a few examples of, of ways that can be asked and get your opinion on what you think is most useful, uh so one thing would be, what disabilities do you have? And that’s that's more specific. I don't know that it gets at specific needs that someone might have because you've already expressed that you know, everyone, even if you had the same condition, everyone is different. Everyone is unique in that way. Um, so, another route is asking specifically what needs do you have or what accommodations should we be providing for you? So that's another way to go about it. And then another one, and this is actually from the US census, it's not really, it was never really designed for healthcare, but we're just showing it as an example so these are questions that ask about specific things about, you know, are you deaf or do you have serious difficulty hearing? Um are you blind or have serious difficulty seeing? Um because of any physical, mental or emotional condition do you have any serious difficulties concentrating, remembering or making decisions?**
110. F: That would be a perfect one. [laughs]
111. **I: Okay. So that one you would say it's to, you're saying, okay, do you have serious difficulty walking or climbing stairs, difficulty dressing or bathing or any difficulties doing errands on your own?**
112. F: Only time you see those questions is when you are looking for services.
113. **I: Okay, okay.**
114. F: Can you see things like that, but in a doctor's office, you don't wanna see that.
115. **I: So, tell me in a, in a doctor's office or eye specialists, you know, which of those questions or what types of questions should, would you want to be asked for fo helping you?**
116. F: Those, those sound good.
117. **I: So you like more specific versus that, just that kind of a, tell me what accommodations.**
118. F: The questions they usually ask are do you have uh heart disease? Do you smoke?
119. **I: Right, they ask about medical conditions more so than accommodations or, okay. So you actually like the wording on these, these you feel comfortable with?**
120. F: Yeah cause then they’re accommodating or they’re making modifications too.
121. **I: Okay, and then what would you expect them? So, you said yes to number three about difficulty concentrating, remembering or making decisions. So, based on that, what would you expect them, how would you want them to follow up if you were asked this and you said yes?**
122. F: So, then that that can be like a prompt from them. Okay, maybe they can ask me a different way, you know or with some adults been on the IQ, you gonna make it in a child-like way. You wouldn't be using uh physician terminology, which a lot of people who don't have disabilities don't understand.
123. **I: So, get rid of the medical jargon. You know, talk at their level, maybe talk slower for some individuals. Okay. Maybe ask whether they're making decisions or whether the caregivers making decisions, things like that. Okay. And so that, would that make you feel more comfortable about being asked to just share that information?**
124. F: Oh yeah. Yeah. And usually in the doctor setting, I don't really like, I had a problem per se. Like I said, you only feel bias when you feel like it might hinder them doing their own thing. Instead of going off -
125. **I: Right. Like preconceived notions, or -**
126. F: Yeah, yeah. Especially as they got older, I mean younger, not so much I guess, but when they start getting older, I don't know if that’s the time when you need to be more specific...I guess it just depends on the specialist or the setting. Those questions might work for pediatric, I don’t know if those same questions would work for OB/GYN or whatever, so it just depends on...but it's a start.
127. **I: Right. So, they might need to be tailored depending on the setting and what services are being offered. Right. Okay. Yeah, that's a good point. Mhm. Okay. So would you think of any other things that you would want to be asked to that better capture anything that you would share or have shared with with the doctor?**
128. F: Hmmm... Not really. Most of the time you see, I don't know how much time there is between the doctor and say the nurse or the nurse practitioners. It just depends, it depends. Surgery wise you’d probably have to ask these questions before she sees the doctor, most of the time she sees the nurse when somebody else is doing the work up, whether it be lab work, so they may not need to be informed on *everything*. But even with them there has to be some sensitivity, some empathy on everything so *they* know what they’re working with as well. I think as a society you're just coming across that all the way across. We have to know the truth. People, even people sitting here, you know these people that are serving them, you know they may not understand. I know other places are making it more user friendly for lack of a better word. But that's stuff’s gotta be the same way. Because of the visual impairment, sometimes I wonder too, she won’t be able to read and won’t be able to see the information, and I’m telling the school's system that because she never came to me. They ignored us. They ignored us for the bulk of her education, fighting for large print books from the time they told me she had a degenerative eye condition cause they kept saying she sees well, she can see, she sees well. It's another doctor. So, it's things like that and they have all the documentation from the physician and have everything in the file. I don't know how to fix that.
129. **I: Right. So, in that instance for number two, the vision difficulties, you'd want them to respond in a way, say for example, larger print materials. If there was like you know health education information being shared about eat healthy or whatever have you rather than maybe a fine print that others might've gotten or maybe even you're asking about assistance walking just so so you don't you know have any bump into anything or anything like that with that, stuff like that. What about your role as a caregiver, do you, would you, are you comfortable with you know a healthcare provider expecting you to play a very active role in being there and assisting and giving input or, or do you think that they need to play a larger role? Where do you draw that line between your role as a caregiver and their role as the healthcare provider?**
130. F: Oh. That’s a tough one. [laughs] Wow. Oh boy. I don't know. I don't know cause that's where I go on both. That's like what I do. Maybe because I don't know. I spend the bulk of the time with her and maybe I know most of them, but...I don’t know.
131. **I: S o, in that sense you think that you should play a role because you know her better than others?**
132. F: Probably yes, but as she gets older, that's the scary part because I don't know how much more she’s gonna want me to be around.
133. **I: So, you want her to have her independence and be able to just do things on her own. That's the other side of it.**
134. F: At least be able to advocate on their own put it that way. Maybe she can't do it on her own. At least having them understand advocacy or trust that she's on familiar ground, like now she's seeing the same pediatrician forever so she knows where we go, we want to see dr. so and so, or we go to this doctor and we go to see the eye doctor, we're going to see this doctor. So, as she rose into a young adult, hopefully she has that same rapport you know and can feel good about speaking whatever the condition may be, yeah. she will continue on with her care. If I'm around, of course I'll be making sure, but if not, then that's one of my biggest fears is that whoever has to help out with her because of the IQ, that they feel on board with her, her health care and all the extras that come along with.
135. **I: Right. But do you feel currently that the healthcare providers that you've interacted with, do they ever kind of use you as a crutch because they don't know what they should know or-**
136. F: Yeah, yeah.
137. **I: So, it's more of that versus like including you in her care you feel like. Okay, okay. Well, do you have, those are all my questions. So, any other thoughts that I missed that you want to share?**
138. F: I don't know if you would call, this it’s an agency, it's called American disabilities something or they changed the name, so I'm not sure the name without having the card in front of me, but I feel like it’s that type of agency–they place too much on the caregiver. They don't offer the support that we need as caregivers to make it better, especially for those of us that work. So, I'm wondering like did you just hit the nail on the head. Like what what is their role? How does it tie into the caregiver and the care taker? I get you can say, I don't know. It's like is everything's supposed to be just me, me, me. Or are they supposed to be able to, I don't know the relationship between the two because the agencies tend to put it all on you. I feel, I don't know, maybe cause they're bombarded and overloaded. I don't know the answer to that one. And then with doctors it's gotta be, information has to be out there and available to all of us. It has to be, it can't be a certain group of people or certain income. It just has to be available to all of us. And who could better advocate for the child and the parent when they're young. Maybe the system has to work both sides. Cause there are some parents that for whatever reason, they don’t advocate for their children. And I feel like it’s off the record that you become the, you're the advocate, but I don’t wanna feel looked upon. And this is like you become the nuisance or the bad person. I don't want to be that person. I just want to know and just want to be involved and help make it better for everybody.
139. **I: So, would you say nuisance in what sense are you seeing or you feel like you're seen that way?**
140. F: Cause if you ask too much questions or you've called too much, then.
141. **I: Okay. So, like you're bothering them with too much, too many questions? Okay okay.**
142. F: Well they want to know why, they want to know why and what I seem like when I asked for medical records from different places, they ask you why? Because they're mine. They're my daughters. That's why. Yeah. So, I don't know. We've got to change that. You should be able to get your records without conditions. I think any of us.
143. **I: Yeah, without the why.**
144. F: Yeah. Yeah. Yeah. I don't know. It’s just room for improvement. I don't want to say one class is better or you know, it’s just room for improvement and things have to change. Everybody should have to change.
145. **I: Right. Okay, thank you and I'm gonna stop this.**
