# Supplementary material for: Disability disclosure in healthcare settings for individuals with developmental disabilities: A qualitative study of patient and caregiver perspectives
Source: PLoS One. 2025 Aug 7;20(8):e0329328. doi: 10.1371/journal.pone.0329328 (PMC12331114; doi:10.1371/journal.pone.0329328)
Supplement: S1 File — (ZIP) [file pone.0329328.s001.zip › Transcripts/2019.12.19 Interview 16 Transcript.docx]

1. I: Interviewer F: Female Caregiver
2. **I: All right so just for the record, you’ve signed informed consent and you don’t have any questions right?**
3. F: yeah.
4. **I: Okay, so, so, tell me about – I wanna understand your health care experiences that you’ve had so far, so in general, do you have good experiences, bad experiences, both to talk about?**
5. F: I would say both. It’s a lot of running around, paperwork, overwhelming at first. Like I wish there was just one place (laughs) where you just did everything, where you just kind of did everything. But it doesn’t work like that. Umm, I started to notice signs of autism and he was 18 months and I said let me give him till he’s two, you know give him some time. He turned two on October 24th and on November 10th I was at a psychologist because, you know, he didn’t get better.
6. **I: So rather recently?**
7. F: Really recent. Uhm, the psychologist diagnosed him, and then it was hard to get an appointment with the neurologist at the [office name] because they didn’t have anything until April, May, and then I called around and most of them were like that. And I was able to get an appointment in two days because I woke up at 6 AM every time and uhm called every 15 minutes–somebody canceled and I got in. So then the neurologist diagnosed him right away, and uhm, so I got the appointment with [Dr. name] and then he diagnosed him, and uhm, you know, they gave me a folder with a ton of information which was really overwhelming. Umm...Then I went to another doctor in [name], which is another mental health place, and they also gave me the diagnosis, the referrals–you know, basically just a bunch of referrals. So, it was hard, like I had to quit my job to sort of just call everywhere and set up appointments–it’s really a full time job to start. So, it wasn’t really a bad experience per se, because I realized that there were a lot of resources out there, but you have to, but it’s really a full time job. It’s very difficult to–you know and as a single mom, so now it’s just like really difficult because I just have to live off–but it’s worth it, I figured I figured two-three years that I dedicate for him since it’s so early for him. He’s gonna start school not behind, and then after that I think he’ll just have a steady schedule with his therapies and stuff like that.
8. **I: Right, right, so you said that at 18 months you started noticing signs, how do you know what to look for or or what might be something like that?**
9. F: I worked with Autistic children in college for two years, I volunteered because I have a degree in psychology, so I kinda knew more or less what the signs were, um he sort of stopped doing a lot of things at 18 months, like everything was progressing perfectly he smiled still, he was an early walker, he would say “Mom”, he would engage, and at 18 months everything kind of went away, and then he stopped saying “hello”, “goodbye”, he stopped you know–you would call him, he wouldn’t look at you, um a lot of issues with the eating, pocketing, um he started just pacing back and forth a lot, um clicking of the mouth, flapping his hands, just a couple different things, certain things, and I started to say okay, something’s off. And when he turned two, I started to really notice when I brought him around my friends kids, and I noticed a huge difference–you know they could sit at a table already, they could you know if you ask them if they could drink this from a cup they could do it, or they seem more like present and that they understood certain things that he just, you know, went right over his head. So, I decided then that I needed to be proactive and do some sort of early intervention for him.
10. **I: Right. And how did you know where to take him, like how did you decide who the right person to take him was?**
11. F: Oh man, that was crazy! So I put him in a daycare for a month you know thinking okay he just needs to socialize he just needs to be around other kids, he’s with me too much–it was a bad experience, they were having him in the corner all day, so he wasn’t even there three weeks so I took him out because I started to catch on, and then I went crazy calling the best academies in Miami and Broward and I called this place called um [name], which was super expensive, way out of my budget, but I heard that they were like the best, right? And, I spoke to the director and I said, you know, I have a feeling that my son might be autistic, and she had like over 20 years of experience and she told me the best place you can take him is [name], that is the best place you can, so I googled [name]–[child sings in background] he likes to sing a lot, (laughs) he’s a good singer. So I googled [name] and I called them every 15 minutes for two days straight, they hated my guts, but I got the appointment in two days, so it worked. And you know after that it was just like, I got all of the flyers, I started calling places, googling, like everything just kind of snowballed. Like I’d go to one place and then they would tell me about other things like that, um and I signed him up for PCIT, at [name] so we do that on Monday’s, and um [name] the therapist there has been really helpful too, telling me, guiding me, [center name] has also been great, cause they tell you kind of what you could do, all your options, but you still have to like do it yourself–like the calendars packed, most of my days are like wake up at like 6 AM, and I have to take all the food to, you know cause he’s on routine, and I don’t get home until 5 or 6, so it really is a full time job driving around to all of the appointments, getting him registered, evaluations, pretty overwhelming at first.
12. **I: So, obviously you had this expert at the school kind of tell you where to go or where, so those are areas where they kind of focus on this specific you know you know condition or similar to it, so would you say those have been positive experiences in terms of how you’ve been treated or how he’s been treated?**
13. F: Yeah, the [name] doctor has been really great, everyone’s been really informative, but I don’t think that–like I feel like I had access to all of that because of my background and you know, I’m proactive, but I know that there’s like in my area there’s a lot, for example, I live in Hialeah, the doctor that’s know here, the neurologist, she likes to I’ve heard she likes to prescribe a lot of medication, it’s not like the same quality, and they’re not as, like I went to her too for a second opinion and I hated it, like it wasn’t very, like they didn’t guide you, they weren’t very helpful, um so I was lucky enough to get that appointment at [name] but I kind of feel bad for anyone else who’s maybe a little more ignorant to the chronic illness, you know, like-
14. **I: because they might not connect to those resources you’re thinking?**
15. F: And sometimes people don’t have time to do all that research you know, and all that stuff, finances, you know work, everything so, I can see how it would be really difficult for someone. I mean I also–I’m a single mom, I don’t have any help, but I know where to find help, you know, help, I know that I could apply for social security and you know there are a ton of ways to make a little cash in the side to get by. But I know that a lot of people who don’t have that community and a full time job.
16. **I: It would be hard to swing both?**
17. F: It would be hard to really dedicate the time, and they need a lot of time and attention, and a lot of exposure, and they need a lot of, obviously, patience. It would be nice if something existed where it was, like, you had someone who just kind of did all that
18. **I: facilitated the process for you? Or with you.**
19. F: Yeah. Because life has to go on, realistically speaking life goes on, you have bills to pay, you know?
20. **I: So, you said they’re really helpful at the places you’ve been to, but they gave you a lot of information that was overwhelming, so did they kind of walk you through that, or answer your questions, how was that experience?**
21. F: They don’t really walk you through it they just kind of tell you this is a program that this this and this call this, so it’s kind of like that, that’s kind of how it works. And you have to do it yourself, or like with [name] they were like sign up for [name], get a care coordinator, they’ll do it for you, and that actually, [name] has been the one experience where he really took his time and like explained every program for me. Umm. He asked me about my situation, he’s like so tell me, you’re a single mom, what do you do? And then he kind of, I felt tailored a little bit more to like our lifestyle. He said from what you’re saying, this is what you might need, and he really explained everything. He really made himself available, he said call me with any questions, you know, obviously I still have to call the places and make the appointments myself, but he kind of just streamlined it a little bit better.
22. **I: So more so [org name] versus something in the health care setting was the most helpful?**
23. F: Yeah, [org name] was more helpful for sure. But then healthcare all over the place. And then insurance comes into play, and some people don’t take insurances and then it’s very expensive benefits, I mean services.
24. **I: have you taken him to any health care providers that don’t you know deal directly with autism or any conditions like that?**
25. F: I would say maybe just his PCP but even that is in [name] and [name] is that mental health organization um so but not really, I’ve been really good about taking him, like I took him to a psychologist first, and the neurologist, then I went to that PCP because I wanted just extra documentation so that the insurance would approve his therapies, so I haven’t really had experiences taking him somewhere that didn’t understand what Autism is. Most people do and I find that very helpful, or the places I’ve taken him. They tell me, you know, which is how I see it, I think he just views the world a little bit differently, I don’t really think his diagnosis like defines him, and most of them seem to be onboard with that idea, and I like that.
26. **I: So you said that it’s helpful, tell me how they make it helpful for you?**
27. F: Because they don’t have, they don’t you know, when he was diagnosed, a lot of family members are kind of ignorant and they have misconceptions, friends, it’s been a little bit isolating in that sense cause I just wanna focus on him, but they seem to get it. There was no stigma attached, they seem to understand, you know, they’re very hopeful, like he’s gonna be fine, he seems very high functioning, Autism is not you know mental retardation or something, like most people sometimes equate that, you know, they seem to understand how he views the world, which I know how he views the world because I’m with him all the time. So yeah.
28. **I: So I know he’s young but do you find that they interact directly with him or more so with you when you’re at an appointment or they’re providing care?**
29. F: It’s a combination of both. Most people really try to interact with him, most healthcare professionals, but obviously he’s so little they have to go off what I say, you know they ask me a ton of questions.
30. **I: and for the PCP that would be the one that maybe doesn’t have that direct experience, has it been noticeably different because of that lack of experience or lack of focus? What are your thoughts?**
31. F: She was actually surprisingly really great. Most PCPs, well his PCP before sucked because I had been telling him since he was 18 months, um okay so that was a bad experience, I had been telling him since 18 months about my concerns and a lot of PCPs and I talked to like other moms and a lot of PCPs just dismiss it, they’re very dismissive, they’re very like no he’ll grow out of it, or let it go, it’s almost like they’re scared of autism, where I feel like there should be more preventative work out there, they should definitely at the first signs, instead of just assuming, no he’ll grow out of it, how about well let’s take him to a neurologist or a psychologist let’s do thorough evaluations let make sure this is not like that he’s gonna grow out of it you know. Um. But I guess it’s just so hard when they’re little, it’s hard to tell, I get that part of it, but his PCP, until I showed up with the psychological, cause I took him to the psychologist first, and then I got that appointment at [name] in two days, so I had to show up at his PCP in the morning and say, you need to give me the referral for the neurologist cause it took me forever to get this appointment, and until I went and I did that and I showed him an actually psychological assessment, he wouldn’t give me the referral. So you know after he was diagnosed a couple, like two weeks later, I changed him to a different insurance. He used to have [insurance name] and I changed him to [insurance name] and with [new insurance] I was able to get services at [name of mental health org] which was so much better because his PCP really seemed to, this PCP really seemed to understand, she asked me a ton of questions, kind of like very similar to the neurologist questions and she was really helpful like she gave me all–I told her look, I have all these referrals, but the more paperwork I have the insurance is more likely to approve the hours. She’s like don’t worry, she everything, all the paperwork–
32. **I: So questions to to help with diagnosing or what kind of questions?**
33. F: She asked me what questions–uh yeah questions about his behavior like like you know when did you start noticing, what is he do that makes you think he has Autism, so it was nice that she didn’t just go off of whatever the neurologist said and just because I had the paperwork that’s it, she really sat down and asked me questions, and then, she said don’t worry I’ll give you all the referrals all over again, I’ll give you a new diagnosis letter, I know that you know the more information that you have the better, um so yeah she was pretty helpful in that sense.
34. **I: and you said before with PCP it was the first one that was not as good of an experience, you know he might be scared of it, if you have any thoughts kind of, what might they specifically be scared of?**
35. F: Uhm I mean that’s as his PCP since he was a a newborn, not that he was scared but he was very like, no no no he doesn’t have autism, you know he has to let it, to grow out of it, and I just had a hunch, you know mothers instinct that somethings off, I noticed him with other kids, and he was just always so dismissive, and he was like no that’s not what it is, you gotta give him time, you gotta give him time. So not that he seemed scared but I think a lot from talking to other moms most PCPs are like that and I think that you know, I guess they don’t want to put like a label, it’s more like labeling that’s the issue which I think is really dumb because the label doesn’t bother me, it might bother other people but not me, you know, I just want my son to get better and if the label is going it help him get better...because at the end of the day without the diagnosis he won’t have access to the resources, so…
36. **I: So you feel with your background and your experience in this area, you’re not afraid of labels but others might be because they don’t know as much?**
37. F: Yeah, the stigma, I mean I have my own Chronic illness, I have too, me too, I was diagnosed when I was very young, so I have, I felt the stigma even now, but you know obviously I don’t care for it, I don’t mind, I I know that it’s just other people’s perceptions that are ignorant sometimes, um so yeah form what I’ve gathered from talking to other moms a lot *a lot* of PCPs are very dismissive when moms bring up their concerns with these signs and everything, they’re very much like oh–
38. **I: Especially it’s the early on not wanting to label or is it more than that would you say from what you’ve heard?**
39. F: Yeah. I think it’s the labeling, they don’t wanna label, they don’t wanna have like–it’s almost like they don’t wanna, they don’t wanna label the child and then the child starts talking or whatever it’s like they don’t want that responsibility or something–that’s like what I feel that it is, but I don’t know if that’s exactly what it is, that’s what I’ve gathered from talking to other moms.
40. **I: Do you think any part of it might be like they don’t know how to identify it, like they don’t have have the experience that you had to identify the signs or?**
41. F: It could be, it could be, but I would think that with how you know all the cases popping up with children with autism in recent years that they would be a little bit more trained when it comes to these issues because they’re the PCP, you know you can’t go anywhere without their referrals, that’s kind of where it starts. If I If I had not been proactive and I hadn’t taken him to a psychologist first, which a psychologist thank god you don’t need a referral from the PCP, but the psychologist the psychologist diagnosis doesn’t weigh anything it’s just an assessment, you gotta get it from your neurologist, so you have to go through–
42. **I: Convince this person to convince this person?**
43. F: Exactly, pretty much, pretty much, so that’s kind of how it was, like let me go to the psychologist to then show the PCP so then the PCP will give me a neurologist appointment. It’s a lot you know. Imagine I wasn’t working at that time because I quit my job, but imagine someone who was working full time. It would take them months to get all of that sorted so then after what, when the baby’s like two and a half almost three then they can start therapy, that’s what I have a problems with. I was able to do it, he was diagnosed on November 14th, I already had all the three evaluations at the center, so he’s most likely gonna start therapy early January, but that’s because I don’t work, and I have the time to be that proactive and I’ve gone everywhere, and I’ve called, and I’ve made the appointments and the evaluations and this and that, but if I was working full time, I know that he wouldn’t have started therapy until three really. That’s how long. And also I was really proactive in the sense that, before I took him to the neurologist, I went and I showed up at [hospital name] and I did a hearing test and an eye exam because it’s just like here in the healthcare system they ask you for, before you do this we need to make an appointment for this, and this, like everything is specialized it’s not like you can go to one doctor that does everything, so that also delays the therapy process.
44. **I: Because you have to hear from all those people before you get the–**
45. F: So I did that before I went to the neurologist, I showed up, I said he needs a hearing exam today, and um they hated me at a lot of places, but you gotta do what you gotta do!
46. **I: Do you think based in that you as a caregiver are treated differently in any way, because you know quote on quote you’re “hated” for the follow up?**
47. F: sometimes they get annoyed because I’m really persistent about everything, I’m his advocate so I’m just like, I don’t really care [laughs].
48. **I: Do you feel like they ever kinda dismiss you or discount you for what you have to think or say?**
49. F: They try, but I have a lot of information because of my background so it’s kind of hard to dismiss me because I just keep trying.
50. **I: So you feel like someone without your background might be dismissed?**
51. F: They would have a hard time, they would have a hard time. I would think so, and I know moms that do.
52. **I: Even with these settings where these are the people that are best suited to provide care and know about autism?**
53. F: Yeah. Because I think they’re overwhelmed, they’re over- sometimes it’s a lot or they’re overworked sometimes, you know I’m not the only mom who has an autistic child whose desperate to get him need, I’m sure all moms are like that, it’s a big diagnosis, um so I think that’s what it is, but you know, I- sometimes I would go, for example, when I try to make a neurologist appointment, they’re like oh we’re not gonna give you the appointment until May, either way you got to get a hearing exam, and I’m like I already have that. Then they’re like you have that? But no you need–no I already have that, I’m telling you, (laughs) you know it’s very they think sometimes if you don’t know–
54. **I: It’s easy to just kick the can down the road cause they’re like oh if I just kind of put the ball in their court then they’re out of my hair?**
55. F: It’s like procrastination, like you gotta do–they just give you all this information to pass it on and just be like okay I told you, but it’s not really helpful you know...very few people actually like take the time to explain certain things so…
56. **I: So one of the reasons why we’re doing this work and asking you these questions and getting your thoughts is you know there’s lots of research and literature that says there’s health disparities people with and without disabilities, and they kind of attribute that to how they’re treated in the healthcare setting. Um, so to address that issue, one of the things that we’re doing is just like document disability status, like how are we going to fix the issue if we’re not even measuring it? So the question first is how do you feel about someone asking whether or not he has a disability? Are you are you OK with that, do you have any concerns about that?**
57. F: I’m OK with it personally, like I don’t have a concern, um if they’re asking for the purpose of helping I definitely don’t have a concern, if they’re asking for the purpose of you know discrimination or you know stigma, then I definitely have a concern. But most of the time when they asked me if he has a disability it’s to provide extra resources so that has been a good experience so far.
58. **I: Okay.**
59. F: But yet again, the doctors in the places that I’ve gone to are geared towards kids that have autism, so I guess that’s like that’s their job. I don’t know if I were to go to PCPs or like other places that I might have the same experience. I can tell you that in the daycare that he went to, it was a terrible experience, they were very ignorant to children with autism, and you know the daycare system here I think should be extremely, way more regulated than it already is, and I’ve had a really bad experiences. He was in two day cares, the first one I thought oh maybe he just doesn’t like it and it was horrible. Daycare professionals will literally lie to you and tell you that everything‘s OK to keep taking your money. Which is why I had to quit my job because now you know I don’t trust anyone. Now, I really need to make sure that he’s in a special-needs school, that they have the training, that they have the patience, and most daycare centers don’t have the time or the patience, they lie to you, and I didn’t realize until I started popping up at different times unannounced. And that is an issue, that is a big issue. Because even Neurotypicals, I don’t think they’re getting developmentally what they need, and these kids are spending hours in daycare is because we all work, you know in order to make it and survive you have to work a ton of hours, so I just don’t understand I guess how the little amount, you have to have barely any education to work in a daycare, you get certified, but that’s all it is. You are super ignorant. A lot of them are super ignorant to autism, when I told them look I’m taking him out because I took him to a neurologist and he’s been diagnosed, they’re like well he only needs a little bit of therapy, he’s just a little aggressive, that’s a lie, and I would ask them every day how was he, did he socialize, did he poop, did he eat, did he sleep? Everything was always, he’s always happy, but I knew that he wasn’t always happy because he’s my son, so that really rubbed me the wrong way. And I told the owner, you told me that he was always happy because you wanted to take my money every week, and you knew that if I- you told me the truth, he wasn’t going to stay here, he needed to go to a special-needs school, or he needed to go to therapy center. So that’s a really big problem, that’s a really big problem.
60. **I: Right, right.**
61. F: A really big problem. I think even bigger than the healthcare system. The healthcare system, you know if you’re persistent enough they’ll help you, I don’t think they really discriminate children with disabilities–if anything they’re more helpful when they know you have a disability, but with daycares, think about it, that’s kind of where it starts, how else would you know if you’re at work eight hours a day?
62. **I: Right, to see signs or what not.**
63. F: That’s when you really see, that’s where it starts, when they’re socializing with other children. I noticed because I would take him to the park two, three times a week, I had taken him to story-time at the library, so I started to catch on, but no, that’s where it starts in daycare, that was a terrible experience, terrible. They will literally tell you what you want to hear so they can take your money, just like with the food, I would send him with food every day and it would come home empty but I knew that he gives me a hard time to eat, and I’m his mom. So again one day I popped in, oh he didn’t eat today, and it kept happening, so so I caught on. But imagine a person who doesn’t have that flexibility at work, who can’t just pop in because they might lose their job the next day. So that makes me feel bad for people.
64. **I: No that’s...Obviously we’re not dealing in that area, but absolutely. So you said in general you’re comfortable being asked that question if it’s intended for helping. Um, so I guess the next question will be, what does that question look like? You know, it could be do you have a disability yes or no? Another suggestion would be, we have here, this is from the the US census and it wasn’t created with the intention of using it for healthcare but just as an uh opportunity for discussion. So these are more specific questions about different types of disabilities, so first talking about death or difficulty hearing, blindness or issues with seeing, difficulty concentrating, remembering, making decisions, so on and so forth. So when you see these questions, do you, what do you think would be the best way to ask these questions that would be helpful to provide quality care?**
65. F: This is regarding healthcare right? Not when they ask you for jobs?
66. **I: Right, right, obviously this was designed for the census, but we’re thinking about what would this look like in the healthcare setting?**
67. F: I think these questions are a little it depends if they’re trying to do an assessment these questions are definitely very limited, but I have the experience when they do do assessments for him, they are very thorough, and they ask you for specific behaviors about the condition, uhm, I don’t think these questions are bad per se, like if they’re asking these questions I think it’s to help you, but you know a lot of these, when you fill them out in doctors offices, I don’t get the sense that anyone even reads them.
68. **I: Is reading them. So not just taking information but acting on it?**
69. F: I think it’s a legality for them in most cases because in the doctor’s offices you usually have the first time these huge packets that I don’t know how long it takes to fill out, but then they see you and it’s like a five minute visit. So you’re not, I don’t think they’re really, it’s all about pushing out patients really, so that is an experience that I’ve had, and even with the neurologist, it they took their time and they did ask me a lot of questions and it was like about I don’t know a 30 minute appointment inside with the doctor, but to be honest with you I expected for it to be longer, more thorough, for them to observe my child a lot more, and a lot of times I feel like well, you gave him this diagnosis just based on what I’m telling you, based on a small interaction with him, so I don’t know, I feel like it should be a little bit more thorough, like it should be a couple hours of an evaluation, really watching him in different settings, it’s a pretty serious diagnosis, uhm and there’s just so much in the spectrum. And when you get the diagnosis, it leaves you with more questions than answers, because they don’t even tell you like where he is on the spectrum, it takes a while for that, and then oh my god to get an assessment like that, they’re expensive, most insurances don’t cover it, and those are the thorough assessments, and I just think it should all be packaged in *one* healthcare provider, you should get out of there really knowing, even for me with *my* mental illness, like I still am looking for answers, like I haven’t had a thorough assessment done, ever, and I’ve asked for one, you know, I’ve never had like a physician or a psychiatrist that’s really taken the time to find like my right combination of meds and it’s been hard even for me as an adult, so it could definitely use some improvement in that area.
70. **I: Right, right. So, so let’s say someone, your son, hypothetically, receives a diagnosis, going to a setting, a healthcare provider that doesn’t have that direct focus of care, you’re just going to a PCP or something like that, you know, what would you want to share with them about your son, or what would you want them to ask you so that they’re providing the best quality care? What specific things do they need to know?**
71. F: Instead of asking, does he have a disability?, I want them to ask specifically thoroughly. Like behaviors, do thorough evaluations, and take their time, actually reading it, and the questions that you did answer yes for, asking more about them, which none of them do that, none of them, never encountered a PCP that does that.
72. **I: And what would you, what would the format look like? Would that be you know one initial question or some initial questions that kind of put up the flag to say hey follow up with me to ask me additional questions and would that be in writing, in person, speaking to them? What do you think that should look like?**
73. F: I think speaking is always best.
74. **I: Across the board, even for the initial question of of what accommodations you might need?**
75. F: No, I think obviously you first you should fill out a form, and then follow up with verbally speaking to you about what you answered yes to or no to or whatever, however the questions framed.
76. **I: So that initial form, what would you like that to say?**
77. F: Uhm…
78. **I: Would you like questions like this, or something more general, or something you know different than—**
79. F: I would like questions that you know—obviously PCPs have a variety of ages, I think they should have ideally different forms for each patient’s age range because you know, developmentally, and then ask you developmental questions, so like how was is child socializing, how is your child eating, are they a picky eater, all the different signs… Do they walk on their tippy toes? All the different things. And I mean autism isn’t the only disorder, there’s a ton, but there has to be a form that they can formulate that can pinpoint red flags in different areas, and you can always add comments and concerns, and you know they can talk to you about it, but you know, must PCPs won’t do that. They won’t do that. It’s all about his weight, his height, the shots, do you have any concerns? I have this concern—oh no it’s okay. Then they leave.
80. **I: So they just kind of dismiss the concern with “oh they’ll grow out of it” or something like that?**
81. F: Yeah. They completely dismiss it all the time, and most of the time they don’t even give you a treatment plan, like they don’t tell you, maybe you can give him certain vitamins to help or...you know one big thing for me is that diet–how important your diet affects literally every part of you as a person and no PCP ever questions the diet of the child or like helps you with baby (inaudible) or how to start, no one, no one. And then even the neurologist.
82. **I: Why do you think that is? Do you think it has to do with their level of training and understanding of diet relative to ASD?**
83. F: Yeah, I think it has to do with their level of training, or they don’t do as much. I guess I don’t know, I’ve done a lot of research and I have him on a really low carb, low sugar diet, and I’ve seen improvement, and I only started to do that like three weeks ago, and I have seen improvement. The other day he said “shoe” in Spanish for the very first time, I had never heard him say that before. So, you know obviously there is some truth to the research out there and you’d think most PCPs would know about it but they don’t or most doctors, and they don’t, it’s really just in your hands to do the research.
84. **I: So when you say they’ve they’ve been dismissive that also includes you sharing the outside information with them and they kind of—**
85. F: Yeah! And they are very also like they will fight you on it.
86. **I: How so? Like challenge the validity of what you’re saying? Or?**
87. F: Or they’ll make a face. Like I tell them I don’t wanna give him no carbs or sugar, and they’re like oh you can eat carbs, and I said well I disagree, I’ve seen improvement. You’d think that they’d be more interested like really? You’ve seen improvement? Tell me more about that. You know, because I’m telling you I’ve seen an improvement big time. Big time.
88. **I: So it’s more of how they come to you, not only tone, but perspective as far as like openness to consider—**
89. F: Other options. Yeah. I guess cause they’re the physicians so they’re very just focused on what their training is or what they know, uh but I know that there has to be other ways you know? Sometimes parenting is a lot of trial and error, so, that’s kind of how I try to do it with him. And I started to just, when he was diagnosed I really buckled down on his diet, whereas before I was way more flexible, just because that’s all I knew, that’s how I grew up. But I had to really change what I knew and my mindset by research, and then I started to also implement like uh supplements, like I give him Omega-3, iron pills in the morning, and I’ve just seen a big improvement in him. Huge. But again, you know, I don’t work, I have the time to do all that. Maybe someone else went somewhere and they gave them like a thorough treatment plan, like look, diet and supplements and routine and sleep and also with the therapy and this and that, it’s like a complimentary of things for them to get better. It’s not just sticking them in a therapy center and that’s it. That’s not the fix, that’s not like gonna fix everything. It’s a very I think complex diagnosis.
90. **I: Right. So, so, one route is what we’ve been talking about and being asked certain questions in person or in forms, another area that people uh have tried is also what we call a health passport where you’re given a packet or you download it from their website that asks you all these questions and it’s kind of like your little information guide that you fill out and you share with the the healthcare provider. Is that something you would be willing to fill out and share as kind of a record of information that they need?**
91. F: I didn’t know that that existed at all.
92. **I: It does but obviously not consistently across settings, it, it, some areas use it.**
93. F: That’s cool, but I don’t think they would read it [laughs].
94. **I: Okay, so it goes back to whether or not they would actually use it?**
95. F: Yeah I could bring everything but I don’t think they would take the time to read it. A lot of the times like I show them, like the PCP at [center name] I showed her the diagnostic letter, the referrals, the assessment by the psychologist, and she just started talking to me and went off what I told her, she didn’t take the time to read them. And a lot of people will do that so…
96. **I: Mhm, so do you have any thoughts or recommendations about what it would take to get them to read that or pay attention to that information?**
97. F: Maybe like an assistant? Like if they’re that busy, have someone to really look at the paperwork, I don’t think they do, I think most people just want to have the paperwork on file for legal purpose and insurance purposes. No one’s taking the time to read it. And then a lot of the diagnosis doesn’t have behaviors attached to that child, you know cause there’s a spectrum, everyone’s different. It just has a diagnosis.
98. **I: Right so what you’re saying is that’s not as helpful because it doesn’t help you pinpoint what the need might be?**
99. F: Yeah, like I know that there is specific criteria in the DSM, to meet like a diagnosis, but not everyone has every criteria on there, so you’re kind of over explaining yourself everywhere you go whereas maybe if you just had like one thorough report, that’s it.
100. **I: Standardized?**
101. F: Standardized. Or - Well maybe not standardized because it would have to be tailored to each patient, but you know, not the way that they do it where they just give you what is it? The F04, I don’t even know and that’s it’s, Autistic disorder. And then that’s about it.
102. **I: Well those are all my questions, unless you have any other thoughts about, you know, you’re experience, but no that was super helpful, thank you.**
103. F: You’re welcome, hope it helps!
