# Supplementary material for: Disability disclosure in healthcare settings for individuals with developmental disabilities: A qualitative study of patient and caregiver perspectives
Source: PLoS One. 2025 Aug 7;20(8):e0329328. doi: 10.1371/journal.pone.0329328 (PMC12331114; doi:10.1371/journal.pone.0329328)
Supplement: S1 File — (ZIP) [file pone.0329328.s001.zip › Transcripts/2019.12.20 Interview 17 Transcript.docx]

1. I: Interviewer M: Male Key Informant
2. **I: Alright so I’ve got the tape recorder on and just for the record we went over the informed consent and you are consenting to to participate, is that correct?**
3. M: Mm, yes.
4. **I: Alright. So so as I said before, we want to know about your health care experiences. So in general, would you say you’ve had good experiences, bad experiences or both?**
5. M: Hmm...you mean in the United States, right?
6. **I: Yes.**
7. M: Good experiences.
8. **I: Okay, okay. So, nothing bad you need to talk about, just good?**
9. M: Mm.. right.
10. **I: Okay, so so you say you’ve got good experiences, so tell me what specifically makes you say that they are good experiences? Tell me about that.**
11. M: Mm... I just think umm cause I’m from [COUNTRY] so umm comparing with the doctors in [COUNTRY], uh the doctors here are way more caring, I think.
12. **I: Okay.**
13. M: Probably because number of patients is not that high so umm so each doctor can spend a lot of time with me. They didn’t only care about my um physical conditions, they also cared about my um mental conditions.
14. **I: Mhm. Mhm. Okay. And when you say ‘they cared also’ it sounds a little bit like in comparison to [COUNTRY] that they have more time to spend with you, but they’re also more caring. So how do they demonstrate caring to you?**
15. M: Mm like um sometimes I had a lot of additional questions and they were able to answer them very patiently. Umm and like when I was umm looking for a place where I could umm diagnose my um developmental disability even though it wasn’t their responsibility, they also provided some help to me.
16. **I: Okay. So referrals help in that sense**.
17. M: Yeah. Yes.
18. **I: Okay. So so you hadn’t been diagnosed with autism when you came to the U.S.? Is that is that what I’m understanding?**
19. M: Right. I umm got diagnosed umm like um just this year.
20. **I: Okay. And how, did … is that something that you were seeking out ... you had some thoughts** **and were seeking out a diagnosis? Or did someone bring it to your attention, how did that work?**
21. M: I just had some thoughts and umm I was pretty sure that I had that condition.
22. I: **Mhm. Okay. So so how did that go down? You went to a doctor and you mentioned these thoughts and they gave the referral, is that how?**
23. M: Well I actually first went to the counseling center and then umm they didn’t do that so they just told me some of the other resources and umm at the same time since um I went to a doctor for some other reasons as well that’s why I asked the doctor, I thought I needed a referral but actually I didn’t need a referral. But that doctor still umm cared a lot about me and umm helped me find some organizations and after that when I went to that doctor again, um he was still kind of concerned about my condition and he just um kept asking me the progress that I made in the diagnosis.
24. **I: Mhm. So showing caring by following up and showing interest?**
25. M: Yes.
26. **I: Okay. Okay. So have you seen any other healthcare providers since you’ve been diagnosed, or at all here in the U.S.?**
27. M: Mmm…not yet.
28. **I: Okay, so just the one?**
29. M: Yea but I mean.. oh you mean like regarding physical conditions?
30. **I: Or any any at all. Any at all.**
31. M: Mmm oh yea! I actually saw one after that,
32. **I: Okay.**
33. M: Umm...It was just like a couple of months ago.
34. **I: Mhm.**
35. M: And the experience was similar.
36. **I: So caring? Showing interest?**
37. M: Yes.
38. **I: Okay. Okay. And so tell me, you know, when you go to a health care provider now, this other one perhaps, you know, do you feel the need to disclose having autism to them or do you feel like it’s not really important for them to know?**
39. M: Umm not really important for them to know.
40. **I: Mhm.**
41. M: But actually that doctor was at the same hospital which is our student health center, so um she could also see the record and so she actually knew that I had autism because the previous doctor already took a note in the system.
42. **I: Okay so she brought it up because she saw it in the note?**
43. M: Yea. So she was also following up.
44. **I: Mhm. And what type of follow up was she doing specifically?**
45. M: She just mentioned all these previous conditions that I had, I mean that uh the previous doctor took a note of in the system and then she just asked me regarding each of these conditions one by one.
46. **I: Mhm. Okay. And what information did you share with her about it when she she mentioned autism?**
47. M: Umm. I didn’t share about anything um I just told her that umm it was already taken care of.
48. **I: Okay. Okay. And she was fine with that? She just wanted to make sure that you had received whatever help that you were seeking out.**
49. M: Right.
50. **I: Okay. Okay. And do you feel like umm having autism means that you’re treating differently in any way than another patient at all?**
51. M: Mmm, no.
52. **I: Not at all? Okay.**
53. M: No.
54. **I: And would you want to be treated differently in any way, given any additional assistance or anything like that?**
55. M: Uh I think for healthcare services I don’t need to
56. **I: Mhm. Mhm. You think that’s true for you as well as other people with autism?**
57. M: Um no. maybe only for me cause for some other people who have autism, maybe they need some additional services.
58. **I: Mhm. Mhm. So there’s no specific accommodations you’d say you would require to to receive quality healthcare?**
59. M: Right.
60. **I: Okay. Alright. Umm so can you think of any other things that when you’re at the doctor that they do for, and it could be the doctor, it could be the nurse, it could be anyone that you interact with, anything that they do that you know makes it a quality experience for you?**
61. M: Mmmmm… umm actually I think umm yes. It was just similar like umm I think the overall experience was just umm with a lot of patience and care.
62. **I: Mhm. And time as well?**
63. M: Yea, and time.
64. **I: Okay. Okay, so you know one of the things that we we talk about umm when we look at the literature, we see disparities between patients with disabilities and patients without, in the healthcare setting. The quality of healthcare that they get. So we’re interested in kind of addressing that and one of the first starting points that we’re looking at is while you can’t really address an issue that you’re not measuring, or documenting, uh so the question would be would it make sense to ask patients to disclose their disability status? So, what are your thoughts about that? Would you like to be? Would you feel comfortable being asked about whether or not you have a disability or not?**
65. M: Mmm, yea I’d feel comfortable. So you mean at first they don’t know anything, they just ask if I have a disability, right?
66. **I: Yea, I mean it could be as simple as that.**
67. M: Yea, that’s fine. But and umm probably I can choose to disclose or not to disclose.
68. **I: Right. So it would be yes, no, or prefer not to share kind of a thing.**
69. M: Yea because it depends on whether my physical condition is related to that.
70. **I: Right so really you wouldn’t want to answer unless you think it’s directly involved in your care?**
71. M: Right.
72. **I: Okay. So so let’s say, I mean, you already kind of suggested that you don’t really need anything different or any special accommodations, but thinking more generally to others that might have autism or another developmental disability, what other questions do you think someone might need to be asked, or to share information about themselves that would give them better quality of care?**
73. M: Hmm...You mean for someone with a disability or?
74. **I: Mhm.**
75. M: So after they know they have a disability, right?
76. **I: Mhm.**
77. M: Okay, so I think they can ask them specifically what types of accommodation they need.
78. **I: Mhm. Would that be an open-ended question like that or would you see like specific detailed questions?**
79. M: I think umm like an open-ended question is fine cause if they really need some additional things they can tell the doctor.
80. **I: Okay. Okay. So open-ended would be good. Let me also give you an example. This is a list of questions that comes from the U.S. Census, so it’s not really designed for the healthcare setting, but just to kind of give you an idea. So here’s six questions that get at different types of disability. So what do you think about these? Obviously we said open-ended would be good, but what do you what do you think? Do these come out… you find these helpful in any way? [male reads questions himself]**
81. M: I think, like for example, for the first one umm like if the person is deaf then umm they are not being able to hear the doctor, I mean if they use some other methods to help and hear the doctor then it’s kind of fine because that’s why I don’t think this type of questions is that crucial.
82. **I: Mhm.**
83. M: And same for the blindness. Umm like cause if they need to see anything, if they cannot see clearly, umm an open-ended is also enough for them to um explain.
84. **I: Document that same information. Mhm.**
85. M: Um, and like the other ones…Um I think for the other ones umm if they really have this type of difficulties while they are um being serviced umm like if these difficulties really affect them then uh they can mention that to the healthcare provider.
86. **I: Mhm. Mhm. So it would more be open-ended and then… so that would be open-ended on a form it looks like but then you would followed up by discussing in person? Is that what you’re thinking?**
87. M: Right. Because otherwise umm sometimes some of these difficulties are not related to the services they receive on that day, and I just think it’s a little unnecessary to umm get all the details at the beginning.
88. I**: Mhm. Mhm. Alright. And do you feel, do you ever have any concern whether for yourself or someone else about sharing the fact that you would have autism in the sense that it might be, you know, it might result in you being treated differently in any way?**
89. M: Are you only talking about healthcare providers?
90. **I: Mhm.**
91. M: Um...Yea, I have had this concern. But, um so far I haven’t seen um anyone who treats these people differently.
92. **I: Mhm. Okay. Okay. Let’s see what else…So let’s say you were in a situation where a healthcare provider was aware of you having autism and treated you differently because of it. How would you respond in that situation?**
93. M: I think it depends on how they treat me differently. Like umm um is it a very bad experience like…um so you mean like they treat me um worse than treating others?
94. **I: That would be one possibility, right. Mhm.**
95. M: Yes so I think if it has a umm if it has a bad effect, probably. If it really affects me, I can just um talk to their supervisors.
96. **I: Okay. So kind of wait till the experience is over but then talk to the supervisor? Or would you stop the experience short?**
97. M: Oh, if its serious enough I can also stop it.
98. **I: Mhm. Okay, okay. So you feel comfortable doing that?**
99. M: Right. But some other people may not feel comfortable that’s why like if they like if some of them have some verbal issues, probably um someone can come with them to ensure a quality service.
100. **I: Mhm. And so let’s go back for just a moment to, you know, when you first went to the doctor and kind of shared your thoughts about possibly having autism and and where’d you go to get an assessment, how would you expect your healthcare provider to respond to that information?**
101. M: Mmm because at that time I already knew that healthcare provider was pretty nice and caring, so I just expected some um kind help from him.
102. **I: Mhm. Okay. What would be, you know, a quote-on-quote bad reaction?**
103. M: Mmm no I didn’t expect a bad reaction.
104. **I: Mhm. Can you imagine what, because you mentioned you know, or am I just asking these questions about healthcare versus outside, so do you have any thoughts about how people outside the healthcare system would treat you if they knew you had autism?**
105. M: Yes.
106. **I: Tell me about that.**
107. M: Um...Like people may judge me because they think um these people are different from other people. So like when I express my thoughts they may think um this person’s thoughts are less reliable or umm or maybe umm are umm less meaningful.
108. **I: Mhm. Okay. So you would see it in a more likely to be a negative reaction than a positive reaction you think?**
109. M: Umm yea, I mean if it’s just um to random people, but if I disclose it to um um some people that are very reliable or some people that I definitely trust, maybe there are positive reactions.
110. **I: Right, right. And the healthcare providers that you have interacted with, they had a good reaction and have given you positive experience – would you say that um they had a good awareness of what autism is?**
111. M: Yes. I think so.
112. **I: Okay. How did they how did they show that they, kind of, had a sense of what it was?**
113. M: That’s because at first I asked them if they were familiar with it and they just clearly responded to me stating that they were very familiar with it.
114. **I: Mhm. Did they ask you any like specific follow-up questions about maybe certain signs of-**
115. M: Yes, they did.
116. **I: Okay. So that was another way they kind of showed their understanding of of the condition?**
117. M: Yes.
118. **I: Okay.**
119. M: But that was only one doctor, so I’m not sure about the other ones.
120. **I: Right, of course. Of course. So do you think that, you know, the good response was because of their awareness of autism or just good bedside manner, both? What are your general thoughts?**
121. M: Umm I think um it’s both. Because they have to be familiar with it otherwise if they don’t know what it is, they may not be able to uh respond properly and in a professional way.
122. **I: Mhm. Okay. Well I think those are, I mean, those were all of my my questions, really. I mean because you had good experiences and sometimes people like to talk a little bit about not so good experiences which takes a little bit of time. But any other thoughts that you have for me about your healthcare experiences or things you would hope you experience when you go get healthcare?**
123. M: Umm actually I do have a couple more thoughts. Umm... Or is that not as I mentioned when I went to see one of the doctors at the student health center - (phone rings) sorry –
124. **I: No problem**
125. M: He took a note in the system and then he um – so the next time I wasn’t able to book an appointment with him so umm I was in an appointment with another doctor and she could also see all the records so like u I wasn’t notified of that beforehand that’s why I was a little surprised because I didn’t know that *all* the doctors can see all the records in the system.
126. **I: When you say you’re surprised is that a good surprise, a not good surprise?**
127. M: Actually, at first umm a little um bad surprise, um but I was still okay with that.
128. **I: Would you say you’re okay with it because of how she responded positively?**
129. M: Yes. And also um I’m pretty sure these doctors will obey their rules.
130. **I: So you think umm if we’re asking people to disclose their disability status, we should make it clear that other healthcare providers can see their records as well?**
131. M: Yeah. Yes.
132. **I: Okay. Because that would be – cause, you know, like you said, you’re okay with it because of how you were treated, but if it was something different, there might be some concerns attached to that?**
133. M: Yes.
134. **I: If they used that information in a less than good way?**
135. M: Yes.
136. **I: Okay. Okay. Any other thoughts then? That was that was a good one to hear, yea.**
137. M: And also umm…oh by the way, the other thought is not regarding my disability because at first you were asking me about any um bad experiences, umm I think I kind of have um another concern like umm I see that when I go to different healthcare providers like when they do some tests umm their umm like uh safety methods are a little different. For example, when I do X-rays, some of them use uh more protections, some of them use less protection. And I think like I just don’t know why they have different levels of safety methods.
138. **I: You’re saying that’s separate from anything – it’s unrelated to disability?**
139. M: Right.
140. **I: The different safety – is that on-campus versus off campus, or is that all..?**
141. M: Yes.
142. **I: Okay, yeah.What are your thoughts about why that might be?**
143. M: Mmm maybe it’s because different healthcare providers have different regulations regarding that.
144. **I: Mhm.**
145. M: Yes, so I just – I’m just wondering why they don’t um unify these regulations.
146. **I: Mhm. Yea wondering if one might be not meeting the regulations and one is or one is or they both are and one is just more… protective**
147. M: Maybe. Yea.
148. **I: Mhm. I’m not sure myself, but that’s an important question. Absolutely. So any other thoughts specifically related to your disability in healthcare?**
149. M: Umm no.
150. **I: Okay. Okay. Well in that case, I’ll pause here.**
