# Supplementary material for: Disability disclosure in healthcare settings for individuals with developmental disabilities: A qualitative study of patient and caregiver perspectives
Source: PLoS One. 2025 Aug 7;20(8):e0329328. doi: 10.1371/journal.pone.0329328 (PMC12331114; doi:10.1371/journal.pone.0329328)
Supplement: S1 File — (ZIP) [file pone.0329328.s001.zip › Transcripts/2020.01.07 Interview 18 Transcript.docx]

***2020.01.07 Interview 18.mp3***

| SPEAKER1 | 00:00 | All right , so I got the tape recorder going , and so let me just ask you first off , what you seem to know . You and your son have had good or bad health care exchanges . Both . |
| --- | --- | --- |
| SPEAKER2 | 00:12 | Would you say you should , even though [spouse] worked for a [University] that doesn't cover many of the young people that epicentres here about human resources at the [University] help [spouse] for the approval of some centers that are not affected by the I don't know how they do , but it is more about health care and hands where has hasn't been able to make they talk with me . Not this , he said . Is that specific to health care needs ? |
| SPEAKER3 | 00:50 | Yes , because [healthcare system] doesn't cover any of your species . You no . To your speech pathology . OK , let's think for each picture . |
| SPEAKER4 | 01:03 | It's so so you said good . Tell me what specifically makes your health care experiences good . |
| SPEAKER5 | 01:13 | Well , at least twenty five dollar copayment used to pass for all , but now we are able to pay for this kind of things for other families for every session . Twenty five dollars for it already is part of a good experience for us . At the beginning , [spouse] , like I had to spend to save someone new . But now we we know how to spend that money . But for other families , maybe they can pay that much for us . Good . Because in nurse to [city] help because we know we are not . We are in and would never spent . They're receiving education and we are not being paid usually like that when we go to an all out of what know . |
| SPEAKER6 | 02:08 | So , so there's that part of it . Tell me about what makes when you actually visit and the actual experience , what makes it a good experience for you in the hospital , either in the hospital , your regular primary care doctor , the neurologist , anyone , they work they work on something , especially for kids in the hospital in the waiting time . |
| SPEAKER5 | 02:29 | We go to all units in [city] because we go through to refer to to the [University] for the pediatrician . We go to , you know , to [city] . Gando , OK , because we had a good a good doctor . But before for us it was because the doctors diagnose the patient . |
| SPEAKER7 | 02:55 | Didn't I know I my son on time , even though we would let him close like [doctor] , if you saw signs and you mentioned to the doctor and several times I mean , that's why it's time for us . I notice that he was different . He was one year old , but he was on the same way . He was three , three years and eight months . He looked kind of late . He was in the early step program because a friend of ours recommended us . But it was . And she's working for the [University] . But why do you think that was ? |
| SPEAKER6 | 03:31 | That they didn't diagnose sooner ? Because they are not aware of the size of kitchen , so they're not aware of the signs . So when you pointed out things that you thought were signs , how did they respond to , you know , you have to work looking like that ? |
| SPEAKER8 | 03:46 | She was like , no , I didn't know my son was in the hospital . But at the same time , I knew that he was a typical child . |
| SPEAKER5 | 03:54 | But I think that we for more . But she was a bit she was you know , maybe they were like focus on the low functioning kids , but they are like my son that mean moderate . And I'm going to give that up . Are in the spectrum of association . |
| SPEAKER6 | 04:13 | No , the clues . So you think that they didn't they weren't aware of the signs and symptoms does work . And you also sounds like you're also saying that maybe they they wanted to kind of encourage you to work with him so that he wasn't there . When , you know , you need to ditch you , you need to show your side more about the process of the development that he like a normal thing . |
| SPEAKER9 | 04:38 | And even though when we we saw that , we told him that , no , he's not playing with other kids in school . She didn't know what to do in my home . I think you should go to the neurologist . I said , could you if you want . |
| SPEAKER10 | 04:51 | And she didn't any we have she didn't recommend that anything . We have to look for ourself . She didn't know anything she could do that could be she didn't know any . |
| SPEAKER11 | 05:01 | So if you asked for a referral , you don't think she could have provided it and see that that was on the south side ? |
| SPEAKER9 | 05:08 | Well , I don't know . Maybe she's more aware now , but he was two doctors , one doctor , [University] Hospital . And I was a doctor and she and they didn't know what else to do , what we did with either . But she was like but I was sure that that my soul was a no , it was not normal . |
| SPEAKER12 | 05:36 | But she was telling me , no , you have to work it . You have to show him the consequences , you know . So that was frustrating . And I had this experience in hospital . But I do have to prepare this . And I went to our for my position and then my son was completely normal . He doesn't have anything that maybe he has ADHD , but I have to wait until he gets five year old to get diagnoses or ADHD . If I was happy because I was there . Two doctors , three doctors . My son doesn't have anything on you , but this , if you me , there was no he's not playing with other kids . And I thought that because he was my my first song . He's an experience . But the teacher said , no , that's not wrong . But I wasn't sure . Nobody told me that he doesn't call me anything because he was assured of a short attention span but did not know his words . |
| SPEAKER13 | 06:40 | So I always figured that maybe we could work with him earlier and they might have let me help out , OK ? I don't know . |
| SPEAKER6 | 06:50 | But it was frustrating to see that you were telling me what to do and say , no , they didn't value your opinion or your your input when you're sharing with Observer . |
| SPEAKER9 | 07:04 | They didn't know about autism symptoms to doctors and nurses . I mean , one doctor in my area . |
| SPEAKER11 | 07:11 | Would you say that they didn't take your information because they didn't think they knew or they didn't care what you knew ? They wouldn't know . They just don't know . So not not enough training about autism specifically . So how did they treat those those pediatricians ? How did they treat your son ? Just like any other child ? |
| SPEAKER14 | 07:30 | Did they interact with him in any different way or know that people are going to be wanting to know what he's doing as he was ? How old at that time to one ? |
| SPEAKER15 | 07:41 | He was the one I moved here when he was one in six months . And I don't think he was even because he was very excited he was fighting for her . But now that they have stopped . But they were like he didn't know how to control . And I didn't when we went to [activity] classes , I noticed that he was the only child who was doing that to her and my son , [spouse] . Look to us . Oh , gosh , you know , you don't have anything to was like [spouse] against me . I know she's different . |
| SPEAKER16 | 08:17 | So . So you realize something was different by observing other kids , or did you do anything else to , like , learn more about later this my other kids and we're talking to something like that . |
| SPEAKER17 | 08:30 | I know , but I was really upset about that . She was the only choice we got . Right . She knew something was different because other kids weren't doing the same thing . OK , so . So you found other pediatricians that were able to diagnose . So how did you find . You said you had to do that on your own . So tell me about that process . |
| SPEAKER18 | 08:48 | Oh , because , well , you know , I mean , they have a lot of peer pressure and sometimes they assign you a roundup . Every week we get I yes . For someone different and they assign me because I see the morally and and I think she's the one who knows more . So you always you got assigned to someone who knew more . OK , so I always make an appointment with her . When is the check checkup for both of my kids ? |
| SPEAKER19 | 09:17 | Because they said something like because I don't know any other doctor , but they are not check up and they will think about my children never get sick , so I only go once a year . |
| SPEAKER17 | 09:31 | So . So what's what's different ? You say she knows . I mean , what , what does she do that that shows you that she knows more and can work with you because she recommends it . So she gets on the clinical about what what she's doing . |
| SPEAKER18 | 09:45 | But see , the other thing that I don't want to get me on this is before they like the additional doctor that she recommended . Go to go , go . Sure . He must be a fishing industry man , you know , because he's a bad patient . He's not a neurologist , but he knows about the Medici's because it's because we've done so . |
| SPEAKER15 | 10:08 | We've made an appointment with him , but we feel a lot of paper work myself . Yes , a lot . But because doctors recommend and go to the doctor , what's the name of the doctor ? Something , go to the doctor and he knows more . Maybe he can do more than the neurologist . I say no , no . I don't think he knows more than I know . |
| SPEAKER20 | 10:35 | But we went to the doctor , but we filled up a lot of paper . We wait like three months for an appointment . And he said no because he's married hyper . I recommend you to see doctor it for me . |
| SPEAKER12 | 10:48 | And she was kind to see whether it was they OK , you have so many things . What medicine do you want to try ? |
| SPEAKER16 | 10:57 | It was like asking you what kind of OK , so you didn't like that . You thought that that because I was getting I know I'm not quite right . So you wanted her to tell you not to her to ask you what you think you should try did . What do you want to try now . Did she just ask that cold or did she give you some options to consider ? No . So we say , OK , we can try conserve that way because we are suffering , because we see a pediatrician as well , or she's appeared to be showing her work with the movement center . |
| SPEAKER12 | 11:32 | So it was horrible to make a lot of people walk away from appointment than when we were waiting for someone else . And the only thing that we gave was a question . What makes it so ? She recommended concern about the . So when we went to the pharmacy , they say , no , we don't have the usual , doesn't cover the brand . So to get the information to the mail , when they contacted the secretary , the secretary was , OK , we're going to go to the are going we have to go to the neurology . If she knows and she's very accessible . She has . She answered the emails that she never gave us . E-mail is all all the same . But the regular for the secretary and the secretary doesn't answer the question . So it's not accessible . And she didn't know we were going to go to the meeting . We were talking to them . And there has been so many things for the first , you know , but the neurologist was the judgment because a friend of us is [name] . But she was in training and she saw that he was good diagnosing children in this picture . So when we went there with my son was three years . He has not in my son . He was very hard on me because he was like , give me a good life sentence . |
| SPEAKER16 | 13:03 | It was hard to hear the diagnosis because we worked very hard , even though you kind of suspected it . No one had . No . I mean , anybody told you that was the first time I did suspect that I thought he was dead for me because my son back then was more interactive , was less hyper . He was more OK . So you you had some suspicions that something was going on , but you didn't think it was autism , that he was alone with my kids and besides yourself has autism , and that's it . |
| SPEAKER12 | 13:33 | We can never have no cure , have anything to do . You can treat me . But it was horrible . |
| SPEAKER21 | 13:41 | And then I didn't know what she was doing , so I didn't want to give you medicine . But he went and he was four . We went , oh , he said he decided to do . He said that all the missing are equal . So just go with the medicine that is covered by the insurance . So she gave him for Kaleem . But I don't do that . I must know what horrible we like steaming all the time . So we were afraid that things didn't work . I'm going to give you the information . And he gave us pay for their refills . We gave him the stance on that , but he didn't give us back . So we said , no , we're not going to do that doctor anymore . |
| SPEAKER12 | 14:24 | We went to the chief of neurology , same [University] , the chief peer pressure new . I'm going to give you that . That makes you the same statement I you , buddy . But I don't want to try to lose you . I want to do it , does it not ? He like me . I can't . I'm the responsible mother and was alone again because [spouse] worked a lot and I went I told my [spouse] said we should file a complaint because I was really hard because I don't really want to even have that mail on you because you said he didn't want to use that . He's only four years old . But he said I hated my father . I say that that makes me so strong . So you explain why you didn't want him to take the medicine and he still was upset that that was you , you know , so it was horrible . [University] training doctor . But doctor told me everything I talked about . The two profession is doctor . |
| SPEAKER22 | 15:27 | I don't remember the name of the doctors . So we because a friend recommended us , we went to doctor . So let's go say she's she's crying . She understood . |
| SPEAKER11 | 15:38 | When I want to give me the when I want to stop , she really she listens and talk to you about how you feel and what you want to do . And she listens . |
| SPEAKER23 | 15:49 | She has a song to speak to me . She knows what she understands . Do you think he understands ? More so it might be personal experience with a child with autism rather than professional training . You may . I have it . Do you have any sense of those that have been helpful to you , whether they had some type of professional training where they know about autism ? |
| SPEAKER22 | 16:05 | Yeah , she she she she has a good system . The . I actually have to work because you have to have you to so you can get appointments , early appointments , but she also goes to the she's much better than the nurse practitioner . |
| SPEAKER24 | 16:24 | Like , I know you got to give me that , but we have to go slow . |
| SPEAKER16 | 16:28 | So you think she does specifically with patients that how about autism or for anyone who she's with ? Anyone . OK , and you mentioned early appointments . |
| SPEAKER22 | 16:36 | Are those specifically for patients with autism or just anyone know for anyone but the when you have when you are already a patient , if you follow her because she answered the question . But we her we have tried we are not built so many stimulants , but they didn't work . So maybe she doesn't do anything to keep on making it worse . Like the side effects you get from Hungary . He is sometimes aggressive for the side effects are worse than any . But we tried one one . We was Quinlivan and he was Kalki down . But by the same time he was very quiet and that may that was so , you know , I think it was last year . |
| SPEAKER18 | 17:23 | We started a meeting in July or something like that July . |
| SPEAKER10 | 17:27 | But in November , October , they had the laboratory [name] . That's more like our there were not that many thing anymore in the United States because the were in [country] and [country] were following with the measles . So while we were waiting for another meeting , the teacher told me that your son is talking , but you said , look at me . |
| SPEAKER24 | 17:55 | It was a well , I didn't know that . Please don't give him anything . I prefer he hyper but interactive than why they can interact with him so that the medicine wasn't allowing . |
| SPEAKER22 | 18:07 | Yes , please don't give me . So we created the last year to live with me , but he was in this in and out some school . |
| SPEAKER10 | 18:15 | The teacher was good , but no , it was hard for him because I really hope so . It was difficult . But she preferred like that because the main thing was keeping him his own war more time . But we finished the school . I noticed he knew everything there was to do now is so anxious . So we we went to Dr. [NAME] before we for for nothing because she didn't kill us . So we returned to the colleges . She recommended that another nun still underneath Schapira because she said , I notice that the students are not good for peace in this picture . |
| SPEAKER22 | 18:53 | It's worth keeping the peace in their favor sometimes to do it . And so now she's with an experiment to see whether he's better . |
| SPEAKER25 | 19:02 | He's still like with amazing . He's more quiet , but it's better for him because when he does not win amazing things , especially the school , I forget because he was very used to I forgot to give him the amazing and everybody knows all day he's more unsure if he's hyper , he's not doing anything . He's going to eat it . He looks at relation with the lazy . He's more calm . He stopped talking , but he's more calm . And it may seem like you was going with a really low dose . The doctor said that for for his weight he should be forty milligrams , but he was twenty five . She's not pushing us to go higher . |
| SPEAKER26 | 19:49 | OK , so she sounds like she's listening to your input and what you want , what you observe and what's working , what's now working . She respected fine . Is there anything else that you would add that she does , whether it be stuff like that or how she interacts with your son that you would say are good health care ? |
| SPEAKER25 | 20:07 | Seems like she she interacts more with them , but with me , because I think that the much look at him , she's not hyper and she always has more . She's not pushing us forward . Go to another blaming . |
| SPEAKER26 | 20:23 | She just wait for the body to come back . And do you feel like she gives you information that could be useful to you proactively or only when you ask for information like referrals or other things that you can do for my friends . And so she doesn't try to interact with your son at all or she tries to talk about , you know , the amount of time is very short time and to gain and is there anything they do , either the doctor or the Yankees or the office as a whole that they do to to better accommodate you and your son because he has autism , whether it be the way that they deliver treatment or scheduling of a . Appointments or anything you can think of like that ? |
| SPEAKER25 | 21:13 | No , I think they are doing the same . OK , but I try to go with the once I remember they used to keep me before God . |
| SPEAKER10 | 21:23 | So they call a lot patient before me . And my son was without me . He was very happy . And they they asked for for him to say , I'm sorry , were you not rolling ? We were confused , but now usually with way that much . |
| SPEAKER22 | 21:37 | OK , so it's never a really long way to make it an issue or anything where you want to it through the waiting is for everything . What they don't respect you , you respect everybody waits . |
| SPEAKER23 | 21:50 | It doesn't matter coming to you in any way . Is that something that you wish they did ? |
| SPEAKER10 | 21:55 | Yes . And you know , have we fight so we Phakisa respect the waiting without doing anything that has nothing to do with the type of stimulation . You mean for my daughter ? She's not even if but gets really bored . But we still going there because we like we like adults are good for the treatment . But she's good from where we were . When I really worry about something , I wait for her appointment with her . |
| SPEAKER26 | 22:24 | So I don't know if this would be something that you've experienced or something hypothetical . But if you're going to a new doctor of some sort and they weren't aware of your son having autism , how would you feel the need to to share that information with them before the appointment or know I should do it to record ? |
| SPEAKER20 | 22:44 | So that way , I don't know . I'm away that much . I , I went to another doctor . Patricia is believing I know about Jackson , that a friend recommended me and she's good . |
| SPEAKER27 | 22:57 | She's on her own party . She's also good because she , she tries , she's trying to another alternative to give me some pictures and she leaves things and she's really good . But we stop going there because it's so far away for me . She's the only one . So she's on vacation . So she's OK . But anyway , so I prefer to go to Canada because there are always my my kids are six times better go there , that's why . But I like her because you don't wait that much . She's very good with the kids . This going sees the great . So when you say she's go with the kids and you say kind what . What else is she . But she will be fine . She's like me her . We're going to give you what Mickey said . So she put the Mickey Mouse computer so she can talk with me while during the days she does like that's productive . |
| SPEAKER28 | 23:55 | You don't ask her to try to entertain you or your keys . So she go home because she's like that . |
| SPEAKER27 | 24:04 | Like she tries to interact like she's funny , she's with the kids . So she looks more like serious . She said she doesn't think he's a good guy , but like , I don't really try to play , you know , make it playful . But the waiting game is horrible . [spouse] all the time . So you have to complain because he said those . |
| SPEAKER15 | 24:29 | That is horrible because they make you wait in the waiting room and then the nurse and then your child all the way through . |
| SPEAKER14 | 24:38 | And then you have to wait in a small room . Right . |
| SPEAKER29 | 24:41 | You go to the doctor , you wait sometimes like forty minutes without doing anything with no . What I mean , that's more crazy . The window of your life is horrible . |
| SPEAKER24 | 24:53 | So just to prepare for that , you know , made up for Newsweek and [spouse] is not going to be horrible for me because I'm going to be alone with the kids . Sometimes I had a nanny so she can do things for [name] and that couldn't live with them because for me it's hard to manage both my two kids and one person . Yeah , that could be and thing the doctor , it comes to the the residence and then I go to go out so quickly and then if you have to see what you need to do . So a vaccine , you got to wait for the vaccine . So it's not lock step , it's like a lot of downtime . Everything that we use for all the kids were the good things . And they never I they don't care because they know my son has a little kid I come to . Sometimes I have to go to the nurse . Sometimes I left . There's so many times I left because I went to do much for the vaccines , usually everything . |
| SPEAKER23 | 25:50 | So they say you really like you said , you haven't really had any accommodations because you brought that attention to him . Having autism is up to you to do that . Because [spouse] knows the system . He knows . You should be faster . You don't have to wait that much longer . Have you seen the behind the scenes ? Me ? |
| SPEAKER20 | 26:16 | It just like a lack of respect for not only for the of the Great War , but keep in perspective , because they don't do anything for all the babies and mother and baby like you have to do anything you have to do to schedule a whole day to go to the food all morning , a whole afternoon , because they think that you have to wait for the lack of respect is more about the wasting of your time and not getting you in and out quickly , more so than how they interact with you . |
| SPEAKER6 | 26:47 | Or would you say there's any other type of lack of respect that you experience before the break ? |
| SPEAKER30 | 26:51 | Because we go and they respond to it in is OK , but getting in is the problem the way everybody people . I finished the vaccination with my kids and I don't even vaccinate for the flu because they don't need it . They never get flu . And I don't know what to do , see it next to the vaccines , make my son war , they they do this over time . Is that something you've observed or the doctors have observed people that usually does . And believe me , about parties with kids in the house and being in my in my son's case because he was a preemie , like he was more worried about autism is not just for one causes many causes , but for my son that he was with me , baby . He was really small . He was he was only three pounds . He was going to was 34 weeks . But I knew that he was his mom because since I was in the 28 weeks and my daughter showed me that my phone wasn't growing , they didn't know why I was . So I lost it . And they never knew why , because I was healthy . I was thinking healthy dialogue . So that happened with me . But nobody told me that you have to take care of your son because maybe he was more of a get out . I told me that we treat me like a normal kid , but I have pictures I looked at , so I looked at my son was interacting with or three months old with other babies , but he was nine . He stopped looking at other women . He was interacting with adults with no babies . So something happened between the three and nine months . She was good . And I told you when he was the doctor say that her son doesn't have anything , which was much more interesting . He was like talking more about this and think you regret now . |
| SPEAKER12 | 28:55 | He let you know that that if my son was doing that , what he's doing now with me and has a note is that he was autism right away , that he wasn't doing that before . |
| SPEAKER31 | 29:11 | So he was like , oh , no , no , no , right away . What about the stories of other families that he's usually in the vaccine , the MMR ? He was then when they were eight years old , 18 months , 18 months , they were you know , they were on a quote unquote , normal track . |
| SPEAKER6 | 29:34 | They got the vaccine . They are . And then they saw kind of a regression . |
| SPEAKER19 | 29:38 | And they have a friend for somebody who has two kids on the spectrum . But she did the vaccine . She's vaccinated in the day , the youngest one . |
| SPEAKER4 | 29:48 | But this operation is basically but since he saw , you know , so they they still are getting them , but they're spreading them out . And you said that your son react badly to the flu vaccine . But no , we're not giving the vaccine . |
| SPEAKER19 | 30:04 | But for example , my daughter is six now . Last year , I have to give in the MMR and I want to work into their lives , but I don't want to because I see a lot of people look , you don't give them the vaccine . They cannot be a patient if you're not . I was looking , you know , so I . |
| SPEAKER4 | 30:30 | I felt I was too . I separate , but I gave him the vaccines to my daughter . She got me a lot for the vaccine , if you will , because I have a friend who studied biology and she told me that the problem with the vaccines is that they give them the liquid that they would do to keep the vaccines is very bad . But people who cost money , they have received a better vaccine that they they don't put that that kind of hard to say to give people more money or people who know receive a better vaccine , that the people that they know , OK , and all the kids get older , some will get cancer or anything . But kids like our small or weak , like my son , they can get out to more susceptible . And also the other thing , it's not only like boxing , the whole thing , like my son , I have a healthy diet now . But before I wasn't aware of like a summer , you know , Assad morphing the boxing , but not seeing a real impact . So , you know , part of the work , like I had mentioned before , is , you know , wanting to make sure that we improve the health care experience for individuals with developmental any disability , really , not just developmental . |
| SPEAKER32 | 31:57 | And , you know , we look at the systems that we're working with , we aren't even measuring or assessing disability . So it's hard to fix something that we aren't even aware of , like who has something or not in a systematic way . So so the first question there is , you know , should we be asking people to disclose their disability status ? I would like would you be comfortable if on a patient intake , formal registration form , if you were asked whether your son had a developmental disability ? Sure , sure . OK . |
| SPEAKER33 | 32:28 | No , I'm not sure . So do you have any concerns about him being asked that at all ? No . OK . So that's been OK . And if we were to ask that question , what would be the best way to ask you ? Should I be asking , does your son have a disability ? Tell me which one or or should it sound like something else ? |
| SPEAKER14 | 32:49 | What do you think would be the most useful to you in the field ? Because this like the question , is there a safety concern ? It's not whether you put your son have any disability or or any difference because some somebodies are susceptible with the word disability . OK , they don't like the word pressure or because your son has any of these diagnosis could be the sort of like a checklist . |
| SPEAKER33 | 33:22 | OK , but because this ability to be competitive , so avoiding that word might be helpful for some people that don't like the word , but you don't seem to be concerned . |
| SPEAKER14 | 33:32 | So for me , it doesn't bother me because they know it for me is that disability is a different reality . |
| SPEAKER34 | 33:38 | I have a friend that she she really likes . |
| SPEAKER14 | 33:45 | She avoided this whole group because for her , because she is very she got very sensitive for that war when I was in the problem . I know you're having a problem because of all the problems for me that she . No , because he just say that you has appointed myself as so I'm not wanting to acknowledge it in that negative light . |
| SPEAKER35 | 34:17 | She's alone out because you don't seem like her . She might be my best friend from school because she said like she said , you do this because , you know , my kids that she didn't like me nor my kids , that I just think my son is Archie . Hey , stop calling me . I saw her and she stopped their friendship and they were friendship while they were friends from when they were little . |
| SPEAKER28 | 34:48 | The school stopped the friendship because of how they talk about the disability as not normal . |
| SPEAKER14 | 34:56 | So like there was somebody that I really like , it's a different disability . |
| SPEAKER33 | 35:02 | So , OK , so you said it'd be good to be asked that question , maybe a checklist or tell me what specifically . So so I think that that's a good place to start . One of the challenges is especially with something like autism , people are on all different areas of the spectrum and have different needs , different combinations . So so considering that , are there any thoughts about how you like your health care providers to follow up or maybe ask additional questions for your thoughts so you can . |
| SPEAKER20 | 35:32 | Do you need a special accommodation for your song ? For you would be great . |
| SPEAKER33 | 35:37 | OK , just leave it kind of Open-Ended . Yeah . No . OK , let me show you something . Now , these are questions that were used for the U.S. Census , so they're not really designed for health care , but just as examples to see what you think . Here are a few different questions of assessing different types of disabilities . So deafness , blindness . This one asks about difficulty concentrating , remembering or making decisions on mobility , like walking or climbing stairs and then dressing or bathing or even going out and doing difficult errands alone . |
| SPEAKER7 | 36:09 | Yeah , but this question where what form , for example , do we use this question ? So that's that's a question . I mean , do you do you like being asked more detailed questions like this or would you prefer that just open ended . What accommodations do you mean ? What are your thoughts in this case ? |
| SPEAKER20 | 36:27 | For example , this would be good for the unknown up . But if you're doing like I mean , the Congress just for getting our service and looking like a specialist or not , that's a specific specifically because , for example , for the provision , I would prefer to wait in the waiting room and say they have a movie on how much better than being in a small room waiting for the rain . But that's the rule there . But if they ask asking for immigration , I would prefer to wait outside . |
| SPEAKER11 | 36:58 | So is that something that you would write on that line about what accommodations it me in the room to wait with no stimulation and they prefer there's more room for me . |
| SPEAKER20 | 37:11 | I prefer outside because of the movie and the movies . I'm not waiting in a dark room . |
| SPEAKER11 | 37:18 | So in that case , in the in the past , do you typically when you go to a health care permit and usually they have you fill out the form while you're waiting , would you want them to ask you those things ahead of time so that they could plan accordingly ? Thank you . |
| SPEAKER6 | 37:32 | Have you ever done , like , filling out at a time where they send it to email or an online portal ? No , there's no they don't ask any question . |
| SPEAKER20 | 37:43 | I usually make that woman by phone and they don't see the they bearing the name . |
| SPEAKER27 | 37:49 | They don't see because I don't know if they've lost anything . So they think my wife and I both feel like they were because there's not a difference , a difference in the patient for the kids right now . |
| SPEAKER11 | 38:04 | So so what if you could change it and make sure that those accommodations are considerations were made ? |
| SPEAKER24 | 38:09 | What would you change about the process to make sure that they were they in the a case that they should have done on their own ? |
| SPEAKER31 | 38:18 | You on the program , on the screen . So they should know that this kid has a preference , but they should know that he has no wait . Maybe they can get an appointment . Early appointments , the first appointment with the last appointment . So they the way that much usually through the first appointment . But even that doesn't always work the way it's supposed to or the way that is meant to . They should be . OK , this isn't usually the kids are more impatient . Usually I don't know any kids that like it the way , but they should have the perfect solution . So the doctors who attended first do everything want . No , wait , wait , wait , wait , wait . |
| SPEAKER11 | 39:06 | Right . So knowing when you do . Right on that . |
| SPEAKER14 | 39:08 | Line autism or know , I need to not be made to wait to kind of readjust their process or streamline it for everyone so that no one is really waiting for that and they do like they are doing that , they would be amazed and they probably want to return that because they're more effective promotional for services . They when the other parent told you more satisfaction with word of mouth , you're saying , well , actually , the interest for my son in a friend of mine told me that they think this is a new drug and his song is like low functioning to me . They were amazing . They were amazing . They were amazing . |
| SPEAKER34 | 39:54 | Because what I want to go to really amazing with my son , he the cure to say when they have kaminis or how it is with and without anything . |
| SPEAKER14 | 40:07 | OK . And as soon as we can know , because we do know that with a movie there , so they have it . |
| SPEAKER11 | 40:15 | So they had a movie kind of interesting to the movie . |
| SPEAKER14 | 40:19 | What movie do they have . A blank . He's very loving . He opened the door and they do like what do they want to do ? One guy with we're going to do the other committees a system and they they know . So they broke it down . They made it comfortable . They gave him a choice to make a movie . So those types of things . And she does she say that for him not to wait ? He's very early in the morning , I have to remove her school of the human body so that if my friend didn't told me that I didn't know him , he still would be waiting . You would think that guy . |
| SPEAKER20 | 41:00 | But if they all the doctors start doing that , it will be the emotional for me because the word about it , that's what parents are talking about , trying to find what you say . |
| SPEAKER31 | 41:09 | Parents are talking because they're trying to find the best care to share the good and bad experience . That's what I did . |
| SPEAKER11 | 41:19 | So because the thing is , like , it's a very there's a better relationship with the clients because kids in this picture that are these parents that you meet , because they they have kids , they go to school with your son as well . Where where are you meeting these parents ? |
| SPEAKER14 | 41:34 | Sometimes in school , sometimes the social group , sometimes for kindergarten . We're afraid because we do many things together and they're very I am very talkative . In the first half of this , this mother , I met him in the city , she's cute , but only once because she didn't like this . But I talked with her in the park and she didn't stop talking to me because she doesn't have any friends . She doesn't know sponging children . Everybody speaks Spanish . You know , she's like , I'm very close to her because she doesn't have many friends , because she doesn't speak Spanish . But because I talk to her , she like she open to me like she couldn't stop her . To talk with someone is difficult . But I am lucky to have a neurotypical child . And for her , my son go to regular party principal has only one kids in respect , two kids in the spectrum . There they are more isolated . |
| SPEAKER33 | 42:45 | And what about your role as the caregiver ? How do you feel ? You know , you what role do you think you should play ? Obviously , you're right now in the role of parents of a young child . But let's look to the future . When your son is an adult , what role do you expect to play as a caregiver ? Should you be heavily relied on in his health care , or should you kind of play him more of a hands off or what do you think he should be ? |
| SPEAKER31 | 43:11 | No , I didn't used to have to be all the time close to the kids because now you need to stay there . Everything is more inclusive and more open to kids in the spectrum . |
| SPEAKER6 | 43:21 | But I still need to improve and the parents have to be close to their kids to think in that sense that the health care providers need to rely on you or they shouldn't have to rely on you or what do you what are your thoughts to be open to you , to receive the the input that we gave ? We should consider your your experiences , your observations , because you're there on the front lines . |
| SPEAKER31 | 43:48 | We know best almost just anything else or is the main thing to go with it . You just have to be because my son is in the middle . |
| SPEAKER9 | 44:02 | We don't know if he's going to be independent or not , but most likely no . So I have to be close to him . Somebody has to be close to him forever . |
| SPEAKER11 | 44:17 | OK , well , those are all my questions . Any other last thoughts that I like kind of made you think of that you want to share that you haven't already know . |
