# Supplementary material for: Disability disclosure in healthcare settings for individuals with developmental disabilities: A qualitative study of patient and caregiver perspectives
Source: PLoS One. 2025 Aug 7;20(8):e0329328. doi: 10.1371/journal.pone.0329328 (PMC12331114; doi:10.1371/journal.pone.0329328)
Supplement: S1 File — (ZIP) [file pone.0329328.s001.zip › Transcripts/2020.01.27 Interview 19 Transcript.docx]

***2020.01.27 Interview 19.mp3***

| SPEAKER1 | 00:02 | You told me over the phone that you had interaction with the health care setting , is that like your like primary doctor or any other specialist or anything about . I will let you take on the night and . I main goal here was going anywhere , some point . OK , so so tell me , in general , what would you say that all of your experiences with all these health care providers have been good and bad , both good and bad ? All right . The reason why we stopped going to [continent] , why we stopped going to the . The U.S. is asking very direct questions about , OK , uncomfortable and light about my . You said like , oh , you shouldn't be asking for that , and then you started when you went to like . The car or something was that and he kept asking more questions and then I caught up and he got really upset at them because they kept doing it after he left . So we had . We're not going back there , but I like it , but doesn't definitely a one way ticket . These are the questions they were when you say that they're just like inappropriate questions . Yeah , I would like to ask you , did your dad do anything to . They overstepped their boundaries . The symptoms that they didn't even call me . OK , so you haven't been back there because of that ? Yes . OK . So what other experiences would you say ? Well , let's let's talk about guess the bad experiences . In your mind , the way you talk about the good ones . Anything else that stands out as a not pleasant or not good experience in health care ? Well , that was my primary care , that she's no longer in primary , not because of what happened , but because I . |
| --- | --- | --- |
| SPEAKER2 | 03:56 | Tap my back on my hand should always do that . Like professional , you would say , or do you think that just personally wasn't something that you you liked for yourself ? Well , my dad did apply for . And not to say I was going to tell her not to touch . |
| SPEAKER3 | 04:25 | It doesn't look like really like . Even though you told her she continues to after all , you didn't even feel comfortable talking about it , I think she said , you know , I said no , I said it , but . |
| SPEAKER4 | 04:48 | I think he did it less , maybe he just did it like a little bit like not as much obvious to us , but then he left so it didn't matter to anyone . Now does . |
| SPEAKER5 | 05:08 | It's hard for me like a doctor . Sometimes I forget why , though , am OK . But your dad , those with you in Gaza , I remember . Yeah . Yeah . |
| SPEAKER6 | 05:17 | So he is there usually there to help out . Yeah . |
| SPEAKER2 | 05:25 | And when you go to the doctors and your dad is there , does he usually so he's usually in an appointment with you unless he has to step out or for certain exams like that . Yeah , I know . I went sometimes by myself . I give it to follow up to get blood work . And I just called out my . |
| SPEAKER7 | 05:49 | So when he there with you , do you feel like the doctor or the health care provider gives their attention directly to you or more sort of him , or would you say ? I they like hearing both of us , but usually there is . |
| SPEAKER5 | 06:10 | Ask him out , you OK ? So do you like the way that they do what you think is right , the way they do it goes right to you ? Yeah , sometimes I just don't play myself very well . Like , sometimes . He'll say something that is to say , because I wasn't comfortable saying , I don't you , that it's OK . So he kind of like fills in anything else or . Yeah , but seems to be like next to you , like , OK . |
| SPEAKER8 | 06:40 | And you do feel that they they listen to you and you consider everything that you say at the importance level that you want them to . |
| SPEAKER5 | 06:53 | Like the Buddha , listen to you . I wish they had better tools to help you , huh ? OK , so tell me about that . What are they what do they not have it ? They should have . |
| SPEAKER7 | 07:05 | Like , maybe they should have , like if they say , oh , the ultrasound maybe had to have the machine there to send you somewhere else . Yeah , because the other one and if I knew they had the machines there and they were . But then that's what they usually take the same day as . |
| SPEAKER9 | 07:46 | Like machines like Salvucci . And one workstation . They don't do that , they're either . So you wish it was like the more like the for you and it was like kind of everything like one stop shopping , it just not taking forever to get an appointment . I like that and also like like if I say I don't like some . |
| SPEAKER1 | 08:25 | Or like suggest like a supplement or something , I always like medication , like , so alternatives to medication . Yeah . Like for you to choose your options . Do you think that you why do you think that they don't recommend that you think that they don't know about those things or what are your thoughts ? Yeah , I think they don't really . I think there are think that the things that are taking place , you have to think about . More commonly , it is sometimes because they have so many ingredients , you can't even read what's on . |
| SPEAKER10 | 09:22 | I'm really that way , am I swear I have no . I know , Jan . So , you know , I'm curious , you do . Tell your health care providers about , you know , you mentioned a couple of things when I asked you if you had a disability , do you tell those things to your health care provider before you have an appointment or during the appointments at all ? What you mentioned , autism , for example . They know that you tell them ahead of time and so they know it . I don't remind them every time . I just like the first time you go and see them and they put it in the record . And so they they know . Did you feel them knowing ? Do you think they treat you any different way , knowing or knowing anything you remember ? I talk sometimes maybe like , no , I really don't like to take these medications . |
| SPEAKER11 | 10:25 | They'll probably go to medications already . That's what they're more than . They like a little like they like our pension maybe . I watch videos on YouTube about people who are commercial where go to the hospital explaining their symptoms , and I just . |
| SPEAKER12 | 10:49 | Don't not tell your doctor , tell your doctor and she starts and they cut the commercial off and then she goes on . I don't know if I explain everything all at once , it's like , so does it feel like they don't know ? You can't really help you . Just like I said , you say one thing you don't have to really say everything on . |
| SPEAKER13 | 11:17 | Oh , I can't sleep because you don't really know if it has anything to do with why you're here anyway , so you're not sure what you want to share , what not to share based on what might be relevant . Yeah , no . One to confuse the dog . |
| SPEAKER12 | 11:32 | There's nothing they don't know what they're doing . It might be too much or unrelated , you're thinking . Yeah . And I don't know about it's like . It's like . I don't know like . |
| SPEAKER10 | 11:47 | I don't want to go there on your problems with . So do you do you do you find it I ask you a lot of questions and I know about autism , or do you feel like they don't really know a lot about it on . |
| SPEAKER12 | 12:04 | They deal with a lot of older people who go there for like I.V. treatments and no , I rarely see anybody in there or anything like . Like Down syndrome , I don't even see anybody go . But when I go back . But you said you don't feel like they treat you any differently knowing that you have autism . I didn't enjoy the fact that they may have one . The and I know better . In what way ? That they can help anyone . OK . I don't know . Maybe helps them learn . OK , so maybe they're so they're open to learning new things . They don't just ignore things that might be helpful to you or or ignore their maybe a little more helpful to me . I don't know . Like if I . I know one thing I don't like is that I don't actually see a doctor actually get this person . |
| SPEAKER10 | 13:15 | That's another thing I don't like . His dad did that to see the doctor that works for everyone . I never saw it . I saw him once briefly because . He actually was doing that one time and I was like across the room right now . He wasn't even sure was . And when I found that out , which was very silly , how would you say that you think that the people you see are doctors ? All right , so so tell me this this doctor or the nurses that you see , you say that you're you might be the only one or they don't see a lot of people with different types of disability . But then that's in comparison . If I you . That says a lot . So do you feel like you're treated differently , depending on whether or not they they have experience working with people with disabilities at all either . |
| SPEAKER14 | 14:08 | I think maybe apply you like they are a little more quick to help the problem that you say , like I give you your . Like like they have more tools and resources , they know that people may or may struggle with to get around to because it's hard to get them out of the house . They want to just , like , do everything I want to because their parents are busy and they don't want to waste your time because you're there for , like . |
| SPEAKER10 | 14:41 | You know , like I need a little more understanding of you then another . No , know , that's not because I don't want to . Right , right . And you say they're more understanding . Do you think that translates into them being more helpful to you in different ways ? |
| SPEAKER12 | 15:00 | Yeah , because they they're more friendly or like they're just they give you more time to just sit there longer , even if you have nothing to say . |
| SPEAKER15 | 15:10 | Just listening to I don't know if it's because of I'm just as I just feel more comfortable , I really don't know what it is , but I know it's probably true to what you know , obviously , you said that . I'm just glad I have that experience . I think it's something new that opened up like a . I don't remember . Oh , my social worker recommended me to go to . Then our second time , the first time that happened , you know , it's the second time . And you said you feel more comfortable , are you ? I think it is being more like kind too concerned at that point when we got . I didn't think it was something bad because I don't feel like there's a wrong question a doctor can ask you . But it just happened just just like this happened , like . That one incident , but I understand , like who's more , right , my dad . |
| SPEAKER16 | 16:43 | At comparing , like I say . Yes , that's right . So you said you're more comfortable there . You said you told me that they take a little bit more time and that you like how they have everything in one spot . But is there anything else you can think of that makes you more comfortable being their . Physically more comfortable to get like if you have a big lean . |
| SPEAKER10 | 17:31 | You know that the office people on the front don't speak to that off their back like a regular hospital . Because they say that this stuff . Thank you , you know . Thank you . Can you think of you know , there's always a concern when to health care providers is aware of a disability , that they might treat you differently in a negative way . Has that ever been your experience ? Sometimes it seems like when they first walk in , like . |
| SPEAKER17 | 18:26 | They just keep quiet , like generally interact with you , like they just go in and get out as quick as possible . I don't know , they deal with everyone or they're just tired and . That's what happened in my other one over here . This kind of rushing through the . We see a lot of people at one time they made me way over . And I found that was like and then when they saw me , it was like less than five minutes , but they may be late like two hours now . I would like to cry . Is just so hard . It was just so like . Shannon , it was like he didn't even say anything like , oh , the doctor is running late or anything . And they just kept me waiting and I went one time by myself . And that happened . I was thinking if I was with my dad , you and treat me , they wouldn't know that you weren't alone . That would look like sometimes discriminating because of my insurance or something like that . Make it look like maybe them seeing . Like that even happened when my dentist's office was making out this dentist and like they made us wait and I was sitting in a chair , my dad was me outside and like . |
| SPEAKER18 | 19:52 | There was like four or five of that went ahead of me and analysis in the you can get treatment and I was like , you know , it was like four people that walked in , walked out and you stood there . And I was like , what ? No way . And then that's when he said something about the shirt . And then he walked out and then we left . Because this girl , I was there before and she was in it , we were in the same room and she was a new patient and I was a new patient . And when she walked in like five minutes . I was like , what I just left and I was annoyed because she sat right next to me and I just watching her , all the doctors around , and I'm like , that's supposed to be like me , know her . And they forget that I was sitting here . So we never went back there . You ever . Then once inside a place in between member . |
| SPEAKER19 | 20:53 | I don't think you just have everybody because , I mean , did you so so you chose the people that you go to sea based on insurance and closeness to , but also by you , because as we worker mentioned , it is another option to accept my insurance . OK , with you , really ? Yeah , so , you know , one of the things that we are talking about is new . The new research tells us that people with disabilities tend to have . |
| SPEAKER10 | 21:28 | Worst health care experiences and those were found . So we're trying to understand how to fix that problem . Part of the conversation is asking whether we should be more specifically asking people whether or not they have a disability . You had mentioned before that your doctor already knew you had autism because you mentioned it initially . So the question is , is that something that you shared with them because you wanted them to ? Or did they actually ask you that like a registration or patient intake form or how do they like that ? OK . And we had them filling out some paperwork for the judge . You were going through the process of . |
| SPEAKER20 | 22:12 | Social Security . OK , so they were helping us to pay more and get like handicap's . Student staff for helping me walk closer to the store where they were , and that was the primary . |
| SPEAKER10 | 22:26 | OK , so so we were looking just at health care , independence of Social Security and that type of stuff . Do you feel . Comfortable being asked whether or not you have a disability . Is that something you would want them to ask ? They could I mean , it'd be smart if they did . OK , so you feel comfortable asking any concerns about them asking and what they do with that information at all ? Yeah , I want to know what they do and why do you want to know , like , tell me how you're going to do that ? So I guess in that sense , what you know , if they were to ask you that , what would you what would you expect them to do with that information ? What would you want them to have that information for ? |
| SPEAKER21 | 23:17 | OK , well , let's look at it in this way , and if if the goal is to share that information so that they can be more helpful to you , maybe they feel they can . Be of assistance to the person , refer me to someone . OK , so referrals to other people that would be helpful to you and they're OK . What else could they do that would be helpful to you ? |
| SPEAKER10 | 23:45 | Think of anything , you know , we're thinking about the things that have been not so good for not the best experiences , things like how it could have gone differently , what they should have been doing instead . |
| SPEAKER22 | 24:03 | They've be nicer . OK , that counts for sure . |
| SPEAKER20 | 24:15 | Maybe like I don't want to have to alter how people act , you know , because if that's how they act around , everyone is like they come and you have to be like fate or whatever can be like is a person . I don't want to feel that kind of beginning because , like , I can tell it , like you just doing it because someone said it at the time , because they always do it and then no one and they do do it next and they just don't . Just like I don't want them to have to feel like you have to remember me all the time . |
| SPEAKER23 | 24:45 | They look at me . I have to remember I was just like , I guess some people , Mahavir . Other people like Angus can they just can't hold it up their like symptoms like to act like they have like Down's syndrome , which is . Is absentmindedness Kansas still like more like they can see more , but like for me , I'm like , I don't like to talk but I like it up . |
| SPEAKER10 | 25:28 | I like I like to spend some time just like my of land there . I have something like . A little bit later , when I try to say something like they may think I'm done saying it , but then they make me feel done because they don't listen , because they kind of push on to the next thing and they don't . So . So patience and maybe . In the process of being patient , maybe even asking you follow questions , trying to coax you or help you remember why it is you're here , whatever information you want to share , is that something that would be helpful ? Yeah . But only if they do it in a genuine way that they should be acting the same with all their patients , right ? You don't want them to be treated differently from someone else is going on here . |
| SPEAKER20 | 26:16 | Yeah , just be aware right now . That there are different people that are coming up doctor's office . And I would think they would know more and more a lot of people . Only one in five kids watch . I have son , Jim . They know , but I don't know . I don't know what they're thinking about . They don't seem to have as much of a knowledge about disabilities as compared to the folks at NYU , perhaps . |
| SPEAKER1 | 26:59 | You know , I don't know if they just think , oh , I have to be nicer to her because something bad about me or they just feel like , OK . She has this or oh , or not because she has this . Let's be nice . You not like me . I'm very sure if I asked them time I know have anyone else that I'll just negotiate . They're going to say no , Springsure and the rest , I'm going to say I'm only . And how does that make you feel , knowing that ? I don't wish they had more experience or it doesn't matter to you . It probably would be a little bit different to me because people need like a little bit a little bit more patience because they don't like the work . |
| SPEAKER19 | 27:51 | You know , they don't like it . But I just don't even want my . You seem to have actually went to regular school , but it was very hard for me . So I don't necessarily think that's good for regular regulators feel like they can be themselves . |
| SPEAKER3 | 28:29 | They sure do it up and up . Yeah . Say you give me more give me more comfortable with who you are , like you go to the doctors if you had . |
| SPEAKER14 | 28:58 | If you had like a . |
| SPEAKER13 | 29:06 | I don't know . So you said you said you need more and only patience , but love . So it's kind of stemming from that idea of just care and attention . So I had something to share with you , so , you know , I asked this question , you said you feel comfortable being asked whether or not you have a disability check on a patient intake form or not . So the question that I have then is , you know , how would you want to be asked ? Would you want to be asked to fill in the blank ? Yes or no ? Do you have a disability or as an other option ? These questions , they're not meant for the health care system . These are census questions , but they're designed to to assess disability . So , you know , if I show them to you , you know , they ask different types of deafness , blindness , difficulty concentrating , remembering or making decisions , difficulty walking or climb stairs . Yeah . Do you like do you like specific questions like this versus a fill in the blank . Yeah . What what makes you prefer the . Does it kind of help , you know , kind of give you ideas about , oh , yeah , I would want them and it remind you of yourself , like your weaknesses , sometimes you forget them , too . So this would help remind you of what you want to share ? Yeah . Let me let me see . |
| SPEAKER24 | 30:30 | Let me have . So so if these were the questions , which ones would you think that you would want to say yes to ABC History and . |
| SPEAKER25 | 30:53 | OK , and another like I could see another one , but I just want to just start off , right . |
| SPEAKER10 | 31:01 | Right . So so if you said yes to those two questions , the whole point of asking you is to make sure that we give the doctors and the nurses more information to be better in giving care to you . So if you said yes to those questions , what what do you want them to do with that information to help give you better care ? Like , if we talk about just three to start , you say , you know , I might have difficulty either concentrating , remembering or making decisions , how can they help you with concentrating , remembering or making decisions ? |
| SPEAKER25 | 31:35 | Maybe they'll say maybe like a pencil and paper , something to write down , whatever you want to say on the paper . OK , so that you feel comfortable or you don't forget something . |
| SPEAKER26 | 31:48 | Yeah . And they can just read all of it , you know , whatever they want with it . OK . It's not like you said everything you could to the doctor and allow for a conversation . Yeah . OK . And what about . Yeah . I think we need to spend more . |
| SPEAKER20 | 32:07 | Oh . Even if he does write one word , we're down to three . What are you feeling . |
| SPEAKER10 | 32:14 | How great having you may or may they get you , so I like the pencil and paper idea , or maybe they can even give you other suggestions to see if you like any other options or ways that they can help you . OK , what about you said yes to six . What can you think of any I know this might be some things outside of the doctor's office , too , but can you think of any ways that they can help knowing that information , like how they might be helpful to . Maybe offering like like . |
| SPEAKER25 | 32:52 | A way to get their life away from their . Additional services that kind of help the area to give you a close by and . But actually , I live in [location] and they have just separated from the war . OK , and so you use those services . |
| SPEAKER27 | 33:20 | OK , I have . You need to go to your house , OK ? Can be more convenient somewhere private , and that is like the biggest . |
| SPEAKER28 | 33:40 | It is I the blood like . |
| SPEAKER10 | 33:48 | So you said you like these questions , do you like the wording or would you change the wording in any way ? |
| SPEAKER5 | 34:04 | I think if you add one , yes , please tell me whether that was my next question , would what is missing that you want them to ask you ? |
| SPEAKER24 | 34:29 | OK , well , I'm trying to also think , you know , if you said some of the more important things to you , obviously having the tools , that's part of it , but also the patients . Can you think of any question that they should ask to then you could say yes to that would mean I need more patients . Do you think like , for example , do you think three kind of tells them to , hey , give me more time , be more patient with me ? You would think so , but sometimes you just have to have it right . OK , so maybe something more and more direct . I had this card . |
| SPEAKER29 | 35:06 | This car is a car , right ? It's like the . You put your finger on it and it shows you where they are going and says , I may have difficulty making eye contact or that I may not be able to understand or comprehend your questions , I may have trouble expressing myself . I may prefer writing over speaking and do not assume this alone constitutes suspicion . One that's really great . Where did you get that ? I got that on . |
| SPEAKER30 | 35:42 | Disability independence . How do you mind if I take a picture just or just take down some occasionally speak to Ali to supply you with unusual . |
| SPEAKER10 | 35:56 | So so this is something that you share with your doctors . What , the first appointment or every time or how do you use this ? Now they're supposed to use it to please contact my dad . Oh , so it's not meant for health care . It's more like I'm pretty sure I can share it with you . I never thought about sharing it with . I do think those types of suggestions are bits of information would be helpful things which I like . Would you like that list of things to be somehow formulated into questions to convey or to share that information ? Yeah , I guess they really someone shouldn't say something . |
| SPEAKER31 | 36:36 | They can just say , Lindsay , are you having trouble expressing yourself right now ? And then I could just say yes . |
| SPEAKER23 | 36:44 | And maybe everyone in the room and my dad , if he's . Something or you say it for me or whatever . |
| SPEAKER10 | 37:01 | Well , I mean , those are those are my my main questions , are there any other things that you that you think I should know that you want to share with me or any other stories about good or bad or any other suggestions for how health care experiences can be better for you ? |
| SPEAKER22 | 37:20 | And we're going to hear are you OK ? |
| SPEAKER10 | 37:28 | OK , well , like I said , thank you a . |
