# Supplementary material for: Disability disclosure in healthcare settings for individuals with developmental disabilities: A qualitative study of patient and caregiver perspectives
Source: PLoS One. 2025 Aug 7;20(8):e0329328. doi: 10.1371/journal.pone.0329328 (PMC12331114; doi:10.1371/journal.pone.0329328)
Supplement: S1 File — (ZIP) [file pone.0329328.s001.zip › Transcripts/2020.01.27 Interview 21 Transcript.docx]

***2020.01.27 Interview 21.mp3***

| SPEAKER1 | 00:01 | Right , so ready to go. So, we're talking about health care experiences. So, would you say that , you know, reflecting on the health care experiences you've had with your twins , have they been good ? Have they been bad ? Both? |
| --- | --- | --- |
| SPEAKER2 | 00:23 | A little bit of both , but mostly mostly good . But there's always some issues . Sometimes when you see health care , do you mean like insurance wise or points to a doctor or mainly with the health care providers themselves or clinicians , whether it be doctors , nurses , nurse practitioners , dentists ? |
| SPEAKER3 | 00:38 | Yeah , OK . Yeah . Some good . Some bad , definitely . OK , so do you mind if we kind of go through the good and the bad . Yeah . As far as the good . I've always had a really good experience with pediatricians , with pediatricians . |
| SPEAKER1 | 00:54 | That kind of just like listening to me and getting like you need referrals for a lot of things or referrals or I mean , you're always open to listen to what I want . And if it's something that's not offered , they try and see if they can work on it . |
| SPEAKER4 | 01:09 | If that's been a good experience , bad has been kind of like with like neurology and sensitivity and stuff like that , you would think that there would be more sensitive syntheses . Children with LSD and other diagnosis all the time , but , you know , I had a specific . Issue with . Something being said in the room with the children in the room and assuming that they don't ever see it because they are non-verbal . |
| SPEAKER1 | 01:37 | But that's not the case as far as I can . |
| SPEAKER5 | 01:39 | One is doing better than the other , things like that that I think bothered . Right . |
| SPEAKER6 | 01:45 | So I had that one issue , and I just basically like rushing through these . Here , I feel like they should take more time . |
| SPEAKER3 | 01:57 | I get it , if a diagnosis happens and it takes you 10 minutes because you see what's going on , but the parents need time to digest what's going on and to feel like you're really doing , you know , everything to tell me what's wrong and giving me all options . Ten minutes at . So that's that's the next . |
| SPEAKER7 | 02:17 | So so let's pick apart this a little bit more so with starting with the good news that the pediatricians have been a good experience . What ? I guess , do you do you feel like you are getting a different type of care , a more informed type of care because your children have autism or that's what makes it good ? Or what do you think specifically as far as listening to my wants and needs ? |
| SPEAKER8 | 02:43 | I think that's the positive because . I don't think I'm getting the best type of care . |
| SPEAKER9 | 02:51 | I feel like I've had to go above and beyond , for example , and so and so then when you know certain things and you request them that they help you get it . But I think it's certain things that should have been mentioned to me from the future . So I think they're lacking . So . |
| SPEAKER5 | 03:08 | So they're willing to help you if you inquire and ask for referrals , but they don't really offer it up . And they shot everything . So not everything that can help you , they just give you an offer , maybe the basics . |
| SPEAKER3 | 03:27 | Maybe a neurologist , and that's it when he goes about that , as you know , it's like , oh , and , you know , do horse therapy , all this stuff , I think that they should have all that information . |
| SPEAKER5 | 03:39 | I don't know if he should talk to you about it or have it in your pocket or something where this could benefit the public things to benefit you so that you don't search for it took years of searching for things and finding things . |
| SPEAKER9 | 03:50 | And I think we should have had the information provided to me . So that's lacking . So do you think that they just didn't really know in the first place ? And you say so ? |
| SPEAKER5 | 03:59 | Yes , some things they don't and some things I mentioned in , they're like , okay , I'll mention it to other people like they didn't know about , which I think is ridiculous to me by [name] . |
| SPEAKER10 | 04:10 | The voice device , the the home , the AC device , it's a device to help . They had no idea about that and . I think it's of a little hard for me to say well to me , because so many kids are . Mainly to say , oh , that's cool , what is that ? You know , for me , that was like this one moment . |
| SPEAKER5 | 04:33 | You know , I of course I didn't know what it was , but I learned through their school and stuff like that . For a bit , I feel like they should have that information , all doctors of everybody who's dealing in their field , so you get a sense that they don't have that and then training for him . |
| SPEAKER9 | 04:49 | Yes , it's not enough . You guys should definitely be . To or even made the speech delayed , just in general , they should come back . Between the ages of , like , I guess three of seven . That's about to be read out the language , so if you don't know that at a young age to share to the parents that they missed . So that's what I think will surprise me . But she said they are open to listen again . Yes , based on research that you've done and for me , so they haven't . And so it might be bigger than now . You know , it might be them . |
| SPEAKER11 | 05:38 | Like they're not getting the information from whatever sources they need to . It's not their fault . I am not sure where it should start or whatever , but they should have . |
| SPEAKER9 | 05:50 | No one's ever been dismissive of information , no , never shared and questions that you never , never will . And do they ever kind of kind of do some legwork or follow up so they can answer your questions in the . And in terms of direct interactions with the . With your boys , do you feel like they adjust their their approach in any way to better suit your sons or any thoughts their . Yeah , sometimes people in the offices . I am I still feel like it's not even . Watch what happens when you see those and sometimes what types of things have they done ? Oh . |
| SPEAKER12 | 06:36 | They've you know , they've been really patient as far as you know , when you have to come in here to take your temperature , your we've they've been really . I don't think that , you know , and some people will be really , you know , totally have the to and I heard [name] . I guess every night I feel like at this point , like every health care facility is . It's probably also beyond them . They're trying their best , but I do feel that if might have wanted . |
| SPEAKER13 | 07:48 | So they're trying , but , you know , they still have a long . So they try a few things here and there , but nothing like you said , like hiring a person or changing a system . Yes , yes . It's basically just the way that it has been . It's just each individual and how they . How understanding they are to you and how pool they are to you , that part is great . But as far as making sure that everything is aligned with the . And do you ever sense that your boys are being treated differently , you know , in a way that might be more negative ? Our base didn't like Sigma assumption . So then let's talk more about the neurologist and the more the negative stuff . So tell me more about that . You said obviously something was mentioned in the room . Yeah , I was insensitive . So tell me more about that kind of stuff . OK , on our second visit , OK . You get diagnosed . We got diagnosed . Different times . Exactly like that's a serious thing . I mean , it was almost to the point where the . Just was typing on the computer and I was like , are you putting that they have . She didn't say , OK , the have of she I mean they do . I mean , and she was like , yeah , that's what I'm doing , and that's I think that . It was more like a use for her , like she stopped turned to you . Yes , she did her evaluation . She was nice during the evaluation , but as soon as she saw her third grade flight , she was going to a computer and start typing . I mean , I'm strong and able to handle it , but what's to say someone else ? |
| SPEAKER14 | 10:39 | She should have known something . I mean , we're on the fifth floor window and you could see the other building and there's cars going around and of course . Not everyone knows how to work , but , OK , he was doing that and the other one was answering questions and being like . Doing better than you know , and he's like . I'm sure you can hear you when you should not be , and it might be a mistake , but I mean , if you've been a professional . My brother , [name] . You know , he knows so and he is not a professional , so like this or , you know , things like that , I . Did anyone say anything to them because not to address it , are you going to let me go ? I left here because I thought maybe at this point we might offend somebody . Leader by my . That was an eventual decision because of that experience . Guess I have to be . Is your pediatrician present something that , you know is just going to be weird or it's like , OK , so it is a little of both ? I would love a private doctor is more aware of . It's just really concentrated in this , you know , area , but the one that I picked . I have his word of mouth that she is the best there , but that's not where I would like to be . I don't want to be for . I would like to know , and when you say she's the best to that in general or specific to working parents with autism ? Oh , no , not working with children with autism . Slowing things down and listening to me in my knees and not trying to be like , no , this is what you need to know if you're listening to me and saying , no , that's not what I want right now . We need to slow this down . And she listens to . |
| SPEAKER15 | 13:53 | If she was she the one that kind of gave you a suggestion to . Get tested and see about a diagnosis , or is that something that , you know , something observed on my home ? Yeah . Yeah , and so was it kind of like you went to get the referral and they said , oh , that's a good idea based on what you're telling me ? Everything . Yes , around like . Right , we talk to that , they say nothing until you're to . But I didn't get anything until after that , but . So going back to the neurologist and the diagnosis , you're told . How did that go about for the time being ? I mean , they sit you down and explain what that would mean , an accessory thing , or was it just like , oh , you're pregnant ? Diagnosis I gave it to you see how she briefly explained the vaccines are at for me and some speech I suggested you get back as soon as possible to some really better . |
| SPEAKER16 | 15:42 | How this helps . Why do you care ? What do you mean ? You know , what is this company ? So I feel like they need to do better . So that's what she said you need for , therefore , you should have speech therapy , make sure you find a place . We were referrals made or did they they asked me to tell you about like no hard or what did you know nothing about nothing . None of that . That all comes with a parent looking into it . I've got a researcher talking another man's . I can't because I stopped working and they're . Gives you a lot of resources , if it wasn't for their school . I was put in some other school that didn't have great resources . I will not . She doesn't even they don't even recommend . Recommend speech therapy . You just left to him on your . Which is a lot of pressure . So you said you don't you don't have any intention of going back to her . Are there any other health care providers that you all have interacted with since those two ? So the one thing that we want to know is it's like if we're going to improve the quality of health care for patients with disabilities , we obviously understand experiences , but also we're able to change the system in a way that will help facilitate that . So one of the first conversations is , well , we aren't even really , you know , assessing disability status to know who has who doesn't have to compare those experiences . So so given that background and information , you . How do you feel about your having or being asked whether or not your son does have a disability on a patient intake form or some type of documentation ? Oh , for me , it's a positive . Yeah , because I feel like there with that . Whoever asks knows they know that they have to . He said they're going to need I don't feel . So , yeah , I love it . No concerns , no , no , not at all . So I guess the question is , how would you like to be asked more like what specific information do you feel is useful to share so that when they're giving care , those things are being considered ? In person or in writing either way , because . We are just people in our community , FSD community are just so used to getting . Stares or , you know . When they can't speak . But everybody wants a health care provider . But there are some things or some ways to react if they're not . Maybe they're the sounds of maybe . And then they're twins , so sometimes it's like . All came together to be received . Yes , typically received together to make it easier for . Everything else health care providers have been pretty much . So if I asked you an open ended question , like , you know , what accommodations your funny would you tell me on ? You're asking me what what accommodations like , if any ? Oh , if you ask me that , I would definitely say that I wouldn't want to be here to be president for the entire visit . I would want to a therapist visit . That's what I would say , I would suggest and then I would say less wait time a time and then . That's wait time and just to basically . If I could have those two things that would go much more smoothly , even if they could help me out by even the Palm . They weren't able to provide the therapy , but they still wanted to be able to themselves provide some of these therapies and provide what what would those things be to you and ask them to do ? Oh , like a person . A person if these . Works in the field and understanding could help me with my . When you swim buddies or when you do like soccer , we went to yesterday . There's a . Why are there motives when it comes to sport , swimming and . So , yeah , just someone who can help a nurse , whatever , that they're just to help . And when you say how what what are the most notable things that they're doing , I mean , maintaining but increasing ? |
| SPEAKER17 | 23:30 | Making sure to like make it even to try to play with them a little bit from . And then while they're in the room , I get them down with . I mean , the doctor is caring for . I know they want to keep it clean , but sometimes even after three . I know that you want to keep it clean , but people are human and waiting it out with a one time . I have your faith has been worth the wait times are long , but they're even longer than promised during the campaign or more than . Liverpool problematic because by the time they gave them an already long and even on time , you're on time , it's a long with time . And then , you know , they don't understand the concept of . So it could cause a meltdown if there's no money to help you when your . It's often in front of my face and I'm willing to help , but there's no one there that's . They're so busy that they haven't ever approached you about . I've seen kids rolling on the floor and nobody's helping . And she was holding on to her while her mom was trying to check out . There's nobody making sure I mean , what if she was really not okay ? What if she wasn't just rolling because she had I mean , I know that she was in high school . But what if she wasn't sleeping on the floor and there's no one hiding ? Why are you kidding ? No one even to you or maybe them them ahead ? It might even prevent that by joining the Times ? I don't think so . Yeah . Nor changing to a different appointment . Time for me or anything . In general . They're not crazy long , but they're still a good amount of time waiting . Waiting for the . It's almost equal on both sides of the wait times down there , and that's I think that's the . We waited , now we're reading it here in the kids . I don't know that you prefer being moved into that room or , you know , whether or . That's right . So I think 15 minutes and then another 20 minutes on . And when the pediatrician comes in and has the appointment . Interact directly with your boy there . I think it's a lot of pain in our . She does it as briefly as she can be aware of it world . It is what it is when I feel like we're trying to get to know who they are . I think if your son was your pediatrician for five years . Their teachers know with them two weeks . Your SO should know , they don't know because it's like the in getting out , you know . Any other questions here ? So even though there's a sense of fear , you feel like you give them time that they deserve rather than just play . I mean , obviously very minor , deserve more time . I mean , do you think you should be more . Or time to kind of find out what's going on . How are things going for questions , just like genuinely getting to know ? And when I have an idea of what their personality is , by the time they're like two years old . So you think especially because they both have autism , that they probably should give more time ? Mean , I think all kids , but all kids in general . But if you have a disability and you have special needs , I think it would . Especially if they are not verbal . So as a caregiver , obviously . Boys are young . You play a larger role than if you were like the caregiver of an adult . Yeah , but you do you feel like the pediatrician or any health care provider that you feel relies on you as a caregiver ? A good amount . Too much . Not enough when it when it comes to maybe taking in information or making sure that the instructions are carried out and things like that relies on me , I think too much . I think I mean , they should be giving me more and I'm giving them . You know , the parent knows more about the child , but . There there are things that I should have known about before I went and researched and found out about , I think . So for the information that you got for them , what if people were afraid so I research information , but like and I'm not saying to . It's about to see whether he went to a research university , maybe that's why I tend to research , you know , but then what about someone who doesn't have that very well ? You're going to just tell them a little bit . They're going to go home . That's it . They're going to look at the wall , if not know that they should be searching for . So you should be that person and give him a reason to be the best and . So in terms of research , what about also down the road in the appointments , perhaps , like you said , there is no therapist , or are you heavily relied on to reduce anxieties or explain blood draws or this or that ? How do you how do you feel ? Do you feel like how they're asking you to interact is appropriate or they should be doing it differently ? What are your thoughts in that room ? I think it's appropriate . I just don't think it's either . I think . And Facebook should be offering so much more . You know , you know everything , let's do everything , let's see how we can lessen this , make this better . You know , it's not just send you to therapy . There's a physical part time . His job to see how we can help with the jump job child maybe has something to do with it . I think that more needs to be looked at instead of just saying , hey , I'm reading your speech . I mean , you know , because that does help . Other stuff does help . It's been proven . It helps . Some people might not believe it , but it's been proven that it helps . So let's look at all of that . I mean , I shouldn't have to . |
| SPEAKER18 | 32:13 | OK , so my son is . Verbal sometimes can yell out and we don't know why he's OK , and then he just lunges for . And it was kind of dismissed . It is OK , they don't have to . You sit still , you know , a company that I feel and let's go see , you give the referral and let's just , you know , rule it out . You should never be an issue with . Let's check it out so that to me is a huge . Yeah , it's just because you're so beautiful doesn't mean that you're not suffering the . And then once it comes out OK , right , that's great . That's . I've never been able to get out , so it's just so many that you never know . And so that's how I think we could change . Also , let's do whatever we need to do . You know , since the child can't speak and doesn't know exactly what what's going on . And someone is not suffering . So before I asked you , you know , an open ended question of , you know , what accommodations would you need if any form you fill out , another way to think about it is , you know , a specific question . These questions are here , come from the U.S. Census , so they weren't really intended for the clinical setting , but just as an example . OK , so these are questions that were written to assess different types of disability . |
| SPEAKER19 | 34:07 | So deafness , blindness issues or serious difficulties , concentrating , remembering , making decisions , mobility . Can you dress and bathe yourself or can you go out on your own doing certain hair ? And so so what do you think ? I mean , would you prefer to be asked specific questions or would you like more of an open ended where you can fill in or what are your what are your general ? I would like to have this specific question and have it open . And also because I do see quite a number two , that would be . Yeah , I see some numbers that would be me , and then I would like to put it , you know , be have . |
| SPEAKER20 | 34:50 | Yeah . And then that would be someone's choice to see . They want to mention the specific . So , yes , I would like to have this plus a line to write it . Then this helps you . |
| SPEAKER21 | 35:05 | To help me whatever way I need , you know . And you said you've got two questions that stand out here , first for you , the answer is I think three to . That would be for them at five . There would be six . OK . Yeah , and you said you like the questions as written , or would you change them in any way or whatever ? Well , you know , I like them . I don't think there's anything wrong . Middle and then I think if someone wanted to elaborate , they should have that line . Because especially if what if an adult is coming to town , they want to put Gary . |
| SPEAKER20 | 35:53 | So you should you you believe that they should probably you have a series of questions like this , but not only this , the open ended and where you actually get to see what type of disability . I always like to get on everything . Everywhere . Yeah . There's so many people should be able to save more , even if they want to right out , I don't know . I know something is wrong , but I don't know . We are a couple years ago , he hurt himself and killed himself and he had a traumatic reaction , but he left a note that said something about . |
| SPEAKER22 | 36:28 | In to find some things , OK , so , you know , but I'm sure big . There's something for everyone to know what or if I do know what , yeah , this is what the . And before you said , you know , you can have it on paper , you could be asked , did you still feel like either way would work ? Or what do you what do you think ? Is the IMF ready for is the best in general ? |
| SPEAKER21 | 37:06 | Someone might not be comfortable with saying it was worth it there , they might use that chair to put it out there so they could see how they could get all of this that they need . So I think that's most important to have a . And then , you know , maybe in another open ended , if you feel like you want to talk to some . You know , so yes or no , whether you want to discuss this or what I did , if it says yes , then if it starts to be discussed and that would be good because I fill out the forms when I go to the pediatrician . And it's asking questions about the safety of the hold the smoke detector . Do you feel like you're safe ? And if you were to check that yes box , I know we've kind of touched on some ideas , but what would you expect to happen ? You answer yes to these questions and yes , you want to discuss it . What do you, what are your expectations of what happens next ? If it's my first time , I would expect to be seen like . And then after that , I guess they've already had to check up on make sure that you're able to . |
| SPEAKER19 | 38:29 | You know , all of your resources and you're continuing to . You mean strike frustrated , you know , by . Express , another negative experience you're reminding me of is . The second neurology appointment . Because there's a Medicaid issue and they're not aware of that . So they didn't yeah , they don't know what . Did he know about it ? I don't know . OK , there was a Medicaid issue last year because Athie therapies were still . |
| SPEAKER23 | 39:11 | Huge . So they were lying about the game , they were they were saying they were with some kids for 80 hours , like really ridiculous , I guess . And , you know , you get paid by hour . So we're talking about , like , shut down people getting any new A.B.C. , which we already like , therapists in Florida and everywhere , and that they wanted to investigate and check out that every therapist in Florida is doing their job in that process . Kids could get therapies . |
| SPEAKER24 | 39:50 | In this process , I go without therapy for a year and a half a year in which it's important to have in between these years so my child's entire life is affected by . Because if they go a year without it now and , you know , after they're like seven years old , the brain kind of slows down . So . So that major issue should be mentioned to every health care professional . You know , when I tell you I have . And I actually get it . You know , this is the reason why . Right . Yeah . Yeah , it was out of it . So things like that . And I briefly told . It was very hard and she was like , OK , you know , it's really important . |
| SPEAKER21 | 40:41 | You need it . You got to find one . And this is someone who I'm not working just for looking and looking and looking everywhere for , you know , medical issues or any sort of not knowing what you should do , go forth and do it . Yes . Never referrals , never an idea why what's going on ? Just like you need to figure this . So things like that , I don't know how they could . Make sure that it doesn't fall through the cracks that everyone has home care provider , those parents . You know , things like that . So do you feel then that it's really they should be able to refer you right in there rather than kind of send you to a card and hope that you get all your connections there ? |
| SPEAKER24 | 41:34 | Yeah , or if they can send me the card , they don't even do that much . I was sent to [disability services] by [name]. But not everyone goes to my place. |
| SPEAKER25 | 41:46 | To the doctors , so I think that's where . And if I was working , I would not have . So are there any other instances that kind of popped up that other kind of lessons learned ? Good , bad stories that you want to share . At the end of my question . Oh , no . Those to the most , I think I've had the most issues kind of like with with their college . Yeah , that's it . Pediatrician . I just wish that they were a little bit more . Well , I guess one question , I mean , kind of get a sense , but I'll ask it directly is since you've been with your pediatrician for over five years , do you feel like she's gotten better with time more knowledgeable and more helpful ? Yes or yes . She's been very helpful . She's more knowledgeable about autism specifically , but I also feel they can get a little bit more . Of knowledge about . Those that the signs are kind of like . I don't know how to explain . As you know . Yeah , yeah , because I have some questions about another half hour . So I have some questions about my . Help for her . Not sure what it is yet . Maybe something like . Something is there , but when you have other children and . They do pick up on some other things and you're trying to get the person help , but she's fine . She's just not to . And I'm telling you , there's muscle issues , biologically hardwired , things like that , that I'm noticing only because it was addressed with the other . It's just her just because hers is like a little bit small or whatever it is , I feel like I'm not getting enough help because they're not providing . |
