# Supplementary material for: Disability disclosure in healthcare settings for individuals with developmental disabilities: A qualitative study of patient and caregiver perspectives
Source: PLoS One. 2025 Aug 7;20(8):e0329328. doi: 10.1371/journal.pone.0329328 (PMC12331114; doi:10.1371/journal.pone.0329328)
Supplement: S1 File — (ZIP) [file pone.0329328.s001.zip › Transcripts/2020.01.31 Interview 20 Transcript.docx]

***2020.01.31 Interview 20.mp3***

| SPEAKER1 | 00:00 | So I'll concentrate on my own experiences with my kids because I've been more involved in their health than mine . |
| --- | --- | --- |
| SPEAKER2 | 00:11 | I haven't had any . But , you know , I can give you a couple of minutes with my kids . So how do we start ? |
| SPEAKER3 | 00:21 | So in general , I usually just ask people , do you ? Would you say , based on your experiences getting health care for your children , would you say that you've had good experiences ? |
| SPEAKER4 | 00:32 | Both . I have more bad than good . |
| SPEAKER5 | 00:35 | OK , so let's go let's go through each of those . And maybe you have specific examples that come . And take away . You want to start with good or bad? |
| SPEAKER2 | 00:44 | I'll start with the bad because I'll go with the timeline . OK , how are we from progress over time . So when my kids got when my oldest got diagnosed , he was two years old . |
| SPEAKER4 | 00:55 | We went to the well , my I started to show concerns to my pediatrician , you know , and one of the one of the opportunities that we had is for pediatricians to be more aware of the early signs of autism that you don't see now , or at least I didn't see nine years ago . I don't know how the early signs are being detected now by the pediatricians , but I kept telling my pediatrician he's 18 months old and he doesn't speak a word . He doesn't even say , mommy , you know , he doesn't even call me mom . She says , don't worry about us . He's a late bloomer . But then , you know , I kept going and I kept telling her just to feel right . You know , people are telling me that . |
| SPEAKER6 | 01:45 | And I see my friends' kids and I see how they're starting to verbalize and it doesn't happen with parties . |
| SPEAKER7 | 01:54 | So she says , look , she looked at me in the face and she told me , look , your son is not autistic . I can assure you that she's not . |
| SPEAKER4 | 02:05 | But if you insist and you want to go to take him to a speech evaluation , I'll give you the refer my my teachers that need the referral . So I went I tried to get a speech evaluation . They are super expensive . They could run like a couple thousand dollars without insurance because without a diagnosis . |
| SPEAKER8 | 02:29 | You know , you have to it out of your pocket and the insurance will pay it and it's like a couple of thousand dollars , you know . |
| SPEAKER4 | 02:42 | The pediatrician is assuring me he's not maybe I'm just overreacting , but then somebody told me about 30 steps or two steps is a great first step . Great early stuff for us parents . So I got an appointment and I went to the steps . They have an evaluation . |
| SPEAKER9 | 03:04 | And that's where we first heard he exhibited some symptoms of autism and you perhaps need to go see a neurologist . OK , you know why the pediatrician couldn't catch on that I you know , I don't know . You know , I know autism is early , but there's need there needs to be an increase of awareness in the whole medical field because you're going to have kids with across the board . So we went to a neurologist we picked supposedly the best in the city , Dr. [name] . He gave us a diagnosis . He was very kind when he gave us a diagnosis . He spoke to us very nicely . He stressed the early intervention , the stress , the therapies , the ABA , the occupational . And he talked to us about an early intervention program that if you was conducting and a TV program intervention , behavioral early intensive behavioral intervention program to . And then he said , you know , if you had family members that would benefit from me telling them , you know , about autism , you bring them over , you know , I'll talk to them , you know ? |
| SPEAKER10 | 04:27 | So , you know , we felt welcomed . The next time we went . |
| SPEAKER11 | 04:31 | I took my parents because my parents played a pivotal role in the caregiving of my kids . My husband and I both work . So and my parents were retiring at the time so they would help us out with them . So we brought them to the judgment , completely ignored them . |
| SPEAKER12 | 04:51 | I had , you know , forget about welcoming our first baby sitter , but a different experience . |
| SPEAKER11 | 05:00 | Yeah , he completely ignored my parents . I told them , look , they're my parents . They play up . They played a role and they were shocked to hear the diagnosis was we were fine . He didn't even direct himself to them . |
| SPEAKER13 | 05:14 | So he said in the first appointment , you know , if you need to bring family to do that and then you did that . Yeah . It was kind of like completely ignore them completely . |
| SPEAKER14 | 05:22 | Ignore that you didn't even speak Spanish . I mean , because you speak Spanish , didn't even speak Spanish . And so , you know , why do you think that was ? I mean , I had no idea what it was he like . Does it seem like it was rushed for time or wasn't even that or . The first time he was in a rush with time . |
| SPEAKER1 | 05:42 | I don't know , you know , I , I don't know . You know , we kept going . Then it became his doctor . Visits to the day is like you go in and they ask you , what is the . You know what , what what do you need me to what do you need me to request ? What do you need me to do ? |
| SPEAKER7 | 06:07 | You know , what is the problem , right ? What is the problem ? What is the problem with [name] ? And I've always felt that we were doing all this work , you know , from all the therapies that he had refers to and he doesn't even ask , what have you achieved ? It's just concentrate on the back . |
| SPEAKER2 | 06:30 | You know , tell me the you know , don't waste my time . What is on me ? What do you want to tackle ? You know , what's the problem ? You know , so it doesn't really follow up on what what's been accomplished . What exactly is it ? No , he doesn't focus on what is positive with the child . |
| SPEAKER15 | 06:47 | Right . So then he got really busy . We saw Kathy , which is a nurse practitioner that he had , and we began seeing Kathy in . |
| SPEAKER2 | 06:58 | You know , I always felt like like every time I would go to him , like I needed to go to therapy because it was like this , he was all feeling . Yes , this feeling of not like you are my kids urologist . Shouldn't you know the positive and the negative , the things that we have achieved with him and the things that the challenges that we're experiencing . And then , you know , record that . |
| SPEAKER5 | 07:25 | Because in that sense , do you feel like , you know , he just doesn't understand that ? I mean , you you had the good the first experience with him . That was a positive experience . So does that lead you to believe that he interacts with enough patients where he understands about on autism and what he's supposed to be ? |
| SPEAKER16 | 07:43 | You know , the good almighty in town , right ? They seem knowledgeable , you say , about autism . He seems knowledgeable , but his bedside manners are not fair . You know , he's all you know , maybe he thinks this his time is worth a lot of money . You know , I like and we're getting on his way or he just wants you to , you know , be leaving his practice and then , you know , he goes away . So anyways , we stay with Kathy in one of the ABA therapist evaluations . They go through the evaluation with you . You know , I notice some OCD surfacing and I notice in the trial and he came on the evaluation and and then , you know , I had my my last this was the last . |
| SPEAKER17 | 08:31 | I just couldn't go further with with [name] . |
| SPEAKER2 | 08:36 | And she says , OK , you know what ? What are your challenges ? What are your plans ? How can we help you ? |
| SPEAKER17 | 08:42 | You know ? And I told her , you know . |
| SPEAKER16 | 08:47 | I brought this evaluation because there's some things here that are surfacing that I'm concerned about and he goes , this is a matter . So , you know , I took my evaluation , I put it in my purse and I told her I don't know why I'm here . |
| SPEAKER18 | 09:03 | I honestly can tell you that I don't know why I'm here , because if this is a matter , you send me to an ABA evaluation and you're telling me that this evaluation that I'm showing you , that I'm concerned about the OCD that is surfacing in , you tell me that this . You know , and then I unloaded my two , three years of frustrations with her right there in there , she told me that I was probably mad for something else and that I was taking it out on her , that she was going to give me five minutes and she was going to come back in so we could start all over . And I told her , you know . |
| SPEAKER1 | 09:40 | You can step away five minutes , but this is the treatment that you guys have given me . And at that point , I was just bringing in one child , bringing two kids . |
| SPEAKER18 | 09:49 | So needless to say , you know , she came back , I . Just didn't give her anything , I just wanted that , you know , you can go ahead and do your thing so you can bill for this visit , but I'm not going to give you any more because I don't believe I'm in the right place . |
| SPEAKER19 | 10:06 | OK , so I spoke to the secretary . So how do I go about transferring my medical records just so that I can you how can I go about transferring my kids medical records so that I can go to another just because I just you know , I didn't bother explaining why that report wasn't and she didn't tell me that it was just an evaluation that I was . |
| SPEAKER20 | 10:38 | Popular that he's also that spectrum . OK . |
| SPEAKER21 | 10:46 | No , she told me that those evaluations have no weight for them . I mean , they mean nothing to them . And did they tell you why they sent you to do it in the first place ? Or I told them , I mean , you're sending me private therapy . |
| SPEAKER22 | 11:01 | You're sending me for occupational therapy . And the therapist is the ones that know my kid best because we only come here every six months . |
| SPEAKER21 | 11:11 | I told her we only come here every six months . You see my child for , what , 10 , 15 minutes at the most ? The ABA therapist comes to my house . They are kind of part of my family because they come to my house . They we have therapy 15 hours a week . You know , they know my kids . They come with me to the IEP meetings . |
| SPEAKER23 | 11:34 | They you know , and then , you know , we I went over with them over these evaluation things that are that I see are surfacing in there . And , you know , and you're asking me my concerns , but you want me to tell you you don't want me to show you because just like . No , I think that she felt that she couldn't read that report , that evaluation , and she felt that . I don't know that if I'm going to explain something that she was not familiar with . |
| SPEAKER24 | 12:03 | So you felt intimidated ? I don't know . Like I don't know if she felt that I was showing a report that she couldn't read , but I was just going to point out she didn't even give me that chance . |
| SPEAKER23 | 12:17 | It was just like that doesn't that didn't give me give me a chance to show her a graph . It was not even like . No , it's just the graph of things that you are pointing up . |
| SPEAKER25 | 12:30 | So anyways , that was the end with itself . As we say , it's another neurologist . She [name] with the difference with SARS and say she's the mom in the spectrum . So she has a teenager by now that , you know , it's in the spectrum . |
| SPEAKER6 | 12:52 | So she speaks my language and the way she runs her practice is very different than what he does . So when we when we come in , number one , it's very little wake time in the lobby . You know , she gets you right in . She has an assistant that comes into the room , into the office , and he starts going through . |
| SPEAKER26 | 13:23 | OK , so how's school ? How is house ? |
| SPEAKER27 | 13:27 | How's everything , you know , positive and negative , you know , and he types it in for her treatment and she comes in , she takes a look at it , and then she starts engaging in a conversation as to , OK , so I see this is working out for you guys . And , you know , what is the next step ? You know what people have thought , you know , how's the therapy ? |
| SPEAKER23 | 13:51 | How's the speech ? How's the occupation ? |
| SPEAKER7 | 13:53 | She goes through a holistic you know , it takes more time . |
| SPEAKER23 | 13:57 | And I know time is money for doctors , but but then , you know , she has given me feedback on vitamins that we can use . She has . So it's just not prescription type . |
| SPEAKER27 | 14:12 | You know , she has told me , like , you know , things that work out with her son , you know , I give him the fight and maintain the fervor . You know , if he eats Gerba , you know , mix it up , you know , she gets mad is because we , like Curtis is a picky eater . He will not eat a gummy . He will not be candy . So how do we give him vitamins , you know , mix it up with fruits or things like that ? |
| SPEAKER28 | 14:33 | And so she's she's very helpful . When you think that helpfulness stems from professional training or more from her personal experience , I think people look more from more from the personal because she understands the frustration of . |
| SPEAKER13 | 14:52 | So I think it's far more empathetic to where we come from . Do you think it's possible for health care providers who aren't known , who don't have that personal experience to also achieve that level of empathy ? Or what are your thoughts there ? |
| SPEAKER15 | 15:08 | If if they if they they have to make the effort to make their . |
| SPEAKER29 | 15:18 | Because , you know , for example , we had a nurse in the pediatrician's office that she knows that both my kids are in spectrum , right . |
| SPEAKER6 | 15:27 | So every time she would see me along with both of them , she would call me in and she will park me , you know , because cordless phones , one way or the other , you know , I'm trying to , you know , get them , control them , you know , to sit . They can sit even though I try , you know , you just sit down . She has a cousin who has a son in the spectrum . So she kind of knows the challenges . So every time she would see me , she would call me and support me , not in in a room . And then , you know , she'll tell me , you know , I know you'll be better here , you know . So , you know , these things made me emotional because it's a lot , you know . |
| SPEAKER30 | 16:12 | So , you know , she she thought that if I guess if you're not if you don't if you're not really aware of the different challenges , the different patients encounter , and I guess you cannot I don't know . You know , sometimes the waiting rooms are a big challenge for our big time because you're trying to control the behavior and then you see other parents judging you . And even though you try not to care , sometimes it's . |
| SPEAKER31 | 16:48 | Sometimes you just depending sometimes you're more sensitive than others . |
| SPEAKER28 | 16:55 | So I try not to make appointments along with both of them . That's my number one rule , but unfortunately , they're sometimes like one of them is sick and my husband is working and I have no other choice but to take the fence . And then then I take the . |
| SPEAKER32 | 17:14 | Or it could be easier just , you know , instead of going to ask you for permission for two doctor's appointments at work to just ask for one , if I know that the accommodation will be there . |
| SPEAKER33 | 17:27 | You know , when you say accommodation , what do you mean ? Like getting us into , like a place where we can wait and and I could control them better ? Is that something that they the accommodation really or is . |
| SPEAKER32 | 17:39 | No , that only that that nursing a pediatrician's office has done it first and then the neurologist trying not not to overload her lobby . |
| SPEAKER6 | 17:49 | So it's as you come in you or like I pick the appointments . Right . I think the first appointment after lunch so I could they can go to school , I can pick them up early and then make the first appointment after lunch . I know that we can get there 15 minutes early . They come back from lunch and then we're in . So I have learned to navigate the system . No way that works for me . |
| SPEAKER13 | 18:11 | Okay , so it's more about actions that you're taking , more so than what the clinicians or the staff are doing to accommodate you . Yeah . |
| SPEAKER26 | 18:22 | And then . And then so that's where we ended up with the neurologist , the neurologist sent me to a genetic geneticist because she believed that we could benefit from a genetic test because there's something she says that they surface genetic test that can you know , sometimes there are diets that can help and things like that . |
| SPEAKER34 | 18:50 | And that's where we met Dr. [name] , kissing , hissing , the mashu that by all he went the worst bedside manners . I went to him because my urologist sent me there . You brought your sons with you at the time I brought my office to . |
| SPEAKER35 | 19:17 | And . At first , he said , so why are you here ? I mean , you know , my neurologist believes that genetics exam could bring out things that she can work with . |
| SPEAKER1 | 19:31 | She's like , no , there are no treatments right now with genetics . There's no genetic therapy right now provided . |
| SPEAKER35 | 19:42 | So , you know , it's not going to help you . So what do you want to know ? What do you want this test ? In which which genetic tests do you want and I told you , I don't know . You know , she's just sent me here and I guess she is going to rely on your expertise . So he gave her car keys like she wants the most comprehensive test . And she's like , what are you looking to find out ? You know , you told me there's going to be good and bad things in here . There's going to be you know , and I've had couples that have divorced because there is a gene present in the woman . And the guy wants to an opportunity to have a son or a daughter with autism and they end up getting divorced . And , you know , and there's going to be you know , you're going to see if he's going to have Alzheimer's , if you're going to have Alzheimer's , if you could have cancer , you you know . And I told him . Well , you know , if if breast cancer surfaces , for example . |
| SPEAKER21 | 20:45 | And then they told me then you're playing God , then you want to play God , you know , and then the conversation goes . And then he told me and then , you know , your son is already what ? He was eight years old at the time . |
| SPEAKER9 | 20:59 | He told me it's too late . You should have come earlier , told for the doctor to give me the diagnosis . Never mentioned a genetic . He never said the benefits of it . He never said , you know , if if he recommended it . |
| SPEAKER20 | 21:14 | So , you know , we rely on the expertise of the people that are treating our kids . And that's why I'm here . I'm not here because I told her I want a genetic test . She is the one telling me . |
| SPEAKER34 | 21:30 | And then , you know , he goes , well , you know , your son is already eight years old . He's clearly affected by the autism . You know , most likely will not be independent . Most likely I'm not going to get married , you know , and that's where I just , you know , I just couldn't say anything . You know , he would he he went outside like there was nothing to bring me back . You know , I said , you know what ? |
| SPEAKER26 | 22:03 | If you were in my place , what would you do then ? What would you do if everything I have told you , I've given you my life story , everything I've told you , what would you do ? So he left the room and he came back with the kid to do the genetic test , you know , but he put me through so much hell , like he made me cry . |
| SPEAKER36 | 22:26 | He told me , you know , I don't a lot of people tell you , you know , your kids are not going to be independent . They you know , they put you down . |
| SPEAKER37 | 22:38 | And as a parent , you are doing the best that you can for that being dependent because you're going to live forever . |
| SPEAKER38 | 22:47 | And to hear from somebody that you trust , somebody that you go for , help me tell you this now , because this is our next . |
| SPEAKER24 | 22:58 | For somebody , they're very hopeful and for it , for any writer , for any human being reader to pick that up , you know , it's either you are very , you know , into your paper and you have lost the humanity or , you know , I don't know what or you think that you're above everybody else . |
| SPEAKER34 | 23:21 | You know . |
| SPEAKER39 | 23:25 | So you could reimagine that experience going correctly . How would it have gone differently ? Does he say whatever he says differently or does he say things are different entirely ? |
| SPEAKER40 | 23:39 | Say he could he could have given me the same amount of information in a different way . |
| SPEAKER20 | 23:45 | Why ? They had to tell me . And thank God no one . I'm not there because I want to be there . |
| SPEAKER24 | 23:50 | I am being sent to him because he's a genetic counselor . And that my neurologist says that in order for the interest to consider me as a genetic test is very expensive . |
| SPEAKER41 | 24:02 | And I have two kids , so you want to the insurance to consider pay , I need to go to a genetic counselor . So that's why I was there . But the things they could have done better just . Tell me if you believe and I guess he has trauma's because of couples that have gotten divorced or I mean , I don't want to have any more children , and I told him that I don't want to have any more children . |
| SPEAKER36 | 24:30 | I have my two kids and that's it , you know , and it is what it is , you know . And I've already made an arrangement with my husband that whatever we hear from whatever side of the family , we're not it's going to be it's going to stay with us because we don't want the families . We don't want , you know , a bigger problem , because if his family knows it's my family that I have it or my family knows that he's he's better the family , you know , we don't want that from him . So he was going to we didn't want to know . But if we were to know , it was going to remain among us . So , you know . He he could have put the positive in the negatives , right ? You know , in a more kind way . |
| SPEAKER41 | 25:21 | OK , because this is you know , if we're going to learn about all these things . You know . I don't want to , you know . My dad just passed away from cancer , you know ? So . |
| SPEAKER29 | 25:37 | And I told him that , you know , and . You know , I know that my grandmother and have studied the family had a history of ovarian cancer . We don't have a history of breast cancer . But , you know , I you of know , and and you can't kind of tell your doctors and they keep track of that . So what is different ? |
| SPEAKER42 | 25:58 | You know , it's not that I'm trying to take on . So , you know , he took my sample , he took Carloss . |
| SPEAKER43 | 26:10 | I didn't pursue it , I didn't pursue it because I didn't want to seem like . |
| SPEAKER44 | 26:18 | They do want to see him again . |
| SPEAKER34 | 26:20 | I didn't want him , you know , because the results he had to read up to us , I , I couldn't I couldn't , you know , now , next month we go to our neurologist . |
| SPEAKER44 | 26:33 | I'll let her know what happened and see what the next step is , you know , because like , you know , and I told my husband , appointments like this , I can't go along . You know , you need to come with me because I never know if I'm going to get you know , now I have to be , you know , emotionally ready to go see a specialist because that's how I feel . |
| SPEAKER45 | 27:03 | I have to be emotionally ready . You don't know what the experience is going to be like because I don't know . |
| SPEAKER46 | 27:07 | What experience do you think also that you should have said things differently ? But you also you know what he said . Do you feel like you was grounded in in reality and that he had a correct assessment of of [name] ? Or do you think that it was more grounded in stereotype or stigma of autism or what are your thoughts there ? |
| SPEAKER41 | 27:29 | You know , some people tell you you're never going to walk away and guess what the person was . |
| SPEAKER23 | 27:36 | You're never going to read and guess what , you know , the person means , you know , and I've never I've never allowed teachers or therapists to tell me and therapists are very careful . They don't tell you what the future . Nobody knows . Nobody knows . |
| SPEAKER37 | 27:58 | You give this kid intervention and you wait and see how he blossoms . But I don't think that no matter how much no matter how much titles you have , you cannot foresee the future . How many times what does he know , what if [name] becomes really independent ? |
| SPEAKER21 | 28:25 | What if [name] gets married ? He doesn't know that this is not for him to decide . It's not for him to say and , you know , we've seen him achieve his milestones late , but he does them . |
| SPEAKER40 | 28:43 | And I think that I do believe that once he's able to control his sensory overload , he's there , there's a child there and he learns , he learns without me even touching him , like we never taught him how to use a remote control in the TV . |
| SPEAKER35 | 29:03 | And just by watching us , he knows how to use it . He knows how to use the kind of navigate through Netflix . |
| SPEAKER27 | 29:10 | He knows how to put YouTube in the TV . He knows . And he he has gifts . You know , he's very musical . He likes to play . You know , I've never he never had he has never had a piano player , but he'll find a note , you know . He became obsessed with Jingle Bells and he tried , you know , started with a piano in the building , Jingle Bells by himself . |
| SPEAKER47 | 29:40 | So . So , yes . You know , he has his . Big challenges , but he also has these things that worked with him and and . I believe that together , you know , he'll find a way to find a way . |
| SPEAKER43 | 29:58 | And if I don't believe in my son , Ashley , who's going to be giving , you know , so my job for him is to advocate for him , you know , have to I don't know . Create a little bit more of awareness of what we go through and maybe tomorrow will be better for us or for the next time I get diagnosed because it's a big shock . |
| SPEAKER1 | 30:24 | This is a big shock to get diagnosed and I . We even need therapy to be able to navigate because it's it's a lot , it's a lot . |
| SPEAKER24 | 30:39 | But so , you know , getting like a better experience is because we go to doctors tomorrow and our kids go to med school . |
| SPEAKER48 | 30:53 | So most of our time is spent in a therapist or doctors shrinks . |
| SPEAKER49 | 30:58 | So would you say that kind of covers the good and the bad ? That kind of stick out for you ? |
| SPEAKER50 | 31:03 | OK . And what about your role as a caregiver ? Obviously , you have younger son , so it'd be a little bit different if I was asking you this when you when you have adult children . But you know what ? What is your interaction with the health care providers ? Do they tend to focus their attention on your sons or do they rely more interacting with you ? |
| SPEAKER34 | 31:25 | Well , my kids are not they don't express , you know , how they feel . They they first interact with me . |
| SPEAKER51 | 31:36 | And then once they get , you know , all the information they need , you know , then they go down to their level . |
| SPEAKER52 | 31:42 | Well , so you feel like those interactions , the way that they they're they're handling are appropriate for the circumstance with their current doctors . So . Our pediatricians are very kind to them . The neurologist was very kind to them , and I know a therapist , you know , they're all very kind to them and they would say that they adjust in any ways that you feel would be helpful to you towards giving care to your son's . Yes , they do , because . |
| SPEAKER32 | 32:18 | They you know , they hear me out and they see them and then they make their judgment and then they , you know , share that their you know , their feedback . |
| SPEAKER35 | 32:27 | And I'm always open , like , I don't think that I'm going to defend , you know , every time I go . But my attitude is open and I'm going to hear you out because you have an expert in a field that I'm not familiar with . You know , my expertise , accounting , your expertise in medicine or genetic studies . And I'm here to listen you out . |
| SPEAKER53 | 32:56 | You know , I'm not here for you to judge me or to tell me that I'm playing God when it's far from what I'm doing . You know , a second judgment and . Or done , let's not talk about the future . Let's talk about now with this benefit me now , because he could have told me , you know , it's not worth the money or you're going to get there's nothing highly significant that you're going to see from here . |
| SPEAKER36 | 33:34 | You know , or like there are other studies that are being conducted at universities that perhaps you might want to look into . You know , like resources , like options . If he really didn't , he didn't believe that I should have written . |
| SPEAKER49 | 33:50 | Do you think that was coming from a point of this lack of information about like training , about disability in general , or what do you think that that was stemming from ? |
| SPEAKER30 | 33:59 | I think it was . I think it was a mix of his bad experiences , I guess I think he feels guilt for a couple of that divorce and he's carrying that with him . So to me , kind of it felt like he had personal issues that he was just applying to making , you know . |
| SPEAKER2 | 34:24 | And so it was that has been the worst thing that was wrong . |
| SPEAKER54 | 34:33 | He even talked to you ? I thought to be . |
| SPEAKER7 | 34:40 | But , you know , I do hope my hope as long as is and that I know I'm not going to be with my kids , you know . All my life is done . You know , empathy and knowledge have to play a role in the medical field because . |
| SPEAKER14 | 35:04 | We only see a medical professional like for 10 minutes once in a while , and the impact that you have , you know , on our you know , we trust that we trust our help in the hands of the experts or the people that we are referring to , you know . |
| SPEAKER55 | 35:24 | So it's like knowing this person is coming here because he's been sent to me because he has concerns or because he's looking to better his life or because he has you know , there's a reason why he exists , you know , because we need to feel comfortable opening up to somebody that we don't know , somebody that's going to keep , you know , the confidentiality of these things , you know , so eat . |
| SPEAKER34 | 35:57 | And on some of that , you have to go . So I know it's . But he has to play . He has to play the test . Clearly , you know , the way it's . |
| SPEAKER56 | 36:09 | When you say you , again , are looking at your role as caregiver , that they know that they rely on you the appropriate amount for assistance . And let me give you an example . You know , it's like , you know , are there certain things that you think they should be doing versus you as a caregiver is that they're not doing and they're relying on you ? Or do they kind of do everything you would expect them to do when providing care for your son ? |
| SPEAKER53 | 36:36 | You know , we caregivers play 99 percent of the role . |
| SPEAKER24 | 36:41 | So we look for an indication if we are doing it right or if there's another way of doing things that can help us . |
| SPEAKER57 | 36:52 | So , you know , I wouldn't expect a doctor to like I would expect for them to give me take , you know , like , you know you know , this is not this is not getting better , Doctor . |
| SPEAKER29 | 37:06 | You know that . You know , like , my son has eczema . Right . |
| SPEAKER57 | 37:10 | And . He would his eyes would get really puffy , and it was , you know , they would tell me allergic is an allergic reaction and he would come back and see what is going on . And until somebody told me maybe you need to take him to a dermatologist , sometimes you don't think , oh , my God , you know , I took him to an allergist , but I didn't take him to a dermatologist , you know , just like that . |
| SPEAKER26 | 37:36 | And then I took him to a dermatologist . She saw him . |
| SPEAKER36 | 37:41 | If you know , in 10 minutes , she devised treatment plan . We've never had any episodes , you know , so it's you know , I said , oh my God , you know , we went through like four puffiness for urgent care visits because I thought he was having an allergic reaction to something bigger . |
| SPEAKER13 | 38:05 | So you think it should have been on the commission to give you a better direction to get you to the dermatologist ? |
| SPEAKER55 | 38:10 | Yeah , it's like , you know , have you considered you know why ? |
| SPEAKER58 | 38:15 | So do you think that that was just something that would have happened to anyone ? Do you think that happened more specifically because your son has autism or . No , I think it would happen to any that you know . |
| SPEAKER59 | 38:28 | They knew he was eczema . But . |
| SPEAKER6 | 38:34 | Yeah , but , you know , I guess the dermatologist treated completely different than how they they were treated . |
| SPEAKER60 | 38:41 | So . I guess , you know , I don't know if he's one of those things that maybe I'm so overwhelmed that I can't think of that , you know , I didn't think to take him to a dermatologist , you know , why didn't I think of that ? |
| SPEAKER61 | 38:54 | You know , and . |
| SPEAKER62 | 38:59 | So , you know , one of the things we're talking about when we talk about trying to improve the quality of care given to patients with disabilities is where do we start ? And one of the questions , one of the thoughts is that we can't even tell if we're doing a good or bad job , if we're not even assessing disability status . So one of the questions is , you know , how do you feel about being asked whether your sons have a disability or not in a patient intake or registration form ? I think it should be OK . |
| SPEAKER63 | 39:30 | I mean , if you ever been asked that question in a forum , I always disclose that he has autism spectrum disorder always , because if you're going to be treating my child , you need to you need to know everything . And when you disclose is there a certain section that you put out on paper or is it just something that you tell them verbally or how , you know , it's on a paper on the intake , you know ? On the registration . So is there like a specific area that asked you about disability or are you where , you know , what are you diagnosis is right ? |
| SPEAKER6 | 40:05 | And then they say other autism spectrum disorder . |
| SPEAKER62 | 40:09 | So usually in other categories . OK . And so you don't . So you think it's a good idea . Do you have any concerns about them going that and treating your sons differently in any way ? Because , you know . |
| SPEAKER29 | 40:27 | By treating different . I think that if you're going to treat different by property , it's OK . |
| SPEAKER60 | 40:36 | But if you're going to treat different for , you know , choosing not to see them or sending somebody else or , you know , so if you have any concerns based on your experiences of anything like that happening . |
| SPEAKER7 | 40:54 | No , no , I have not . But , you know , I . I do hope that accommodation's , you know , play a big role in our lives . |
| SPEAKER32 | 41:09 | So having , you know , like maybe the receptionist , not the patients coming and socialism and just think , OK , you know , they see if somebody is having trouble containing , you know , because sometimes kids don't feel right . Even though my son knows and we teach them , you know , a doctor's office doctor's going to make you feel better . But like when and that's why I try to go along , because I don't want to put myself in a position that I can't control . But it's just , you know , the norm . And I guess the person that welcomes you sees that something is out of control with somebody struggling with something . You know who the men you know , this person needs to be . You know , let's do something for them . You know , this empathy , empathy first all . |
| SPEAKER63 | 41:58 | So so you you think that the person reading would be a nice person to kind of observe ? |
| SPEAKER24 | 42:03 | And I would that's the first person that kind of sees , you know , the person who's waiting patiently and who is having an issue with their kids . And it's not that you're going to put me ahead of the light or just get me in , you know , so I can control this scenario . And , you know , I'll wait my turn right . I have , like , a room for those special special needs , you know ? |
| SPEAKER49 | 42:30 | And so , you know , I guess one of the questions is , you know , how would you like you know , if you're going to write the information , you say you check other and you put that information in . But given especially something like autism , where there is a spectrum or maybe different accommodative means , how would you want them to convey that you want then to to rely on them to kind of make their observations ? |
| SPEAKER62 | 42:51 | Or would you want to specifically say , like , these are the types of things that would be beneficial to to my sons during their appointments ? |
| SPEAKER10 | 42:59 | I think that there's got to be a starting point . Right . So it's . Yeah , maybe one or two things , you know , what would you what what can the practice help ? How can the practice help you feel more comfortable ? |
| SPEAKER63 | 43:15 | How can we make you feel more comfortable , you know , believe it kind of like open ended so that you can express what you want to say . Yeah . |
| SPEAKER10 | 43:22 | Like shorter or wait time outside , you know , not to see a doctor , but shorter hour . Wait , come outside if you you know . |
| SPEAKER43 | 43:33 | If possible , because sometimes you might have another kind of hearing in the room that you have available for analysis , but , you know . The person that's in the front can you know , after the initial visit , then they can they can start looking , you know , and seen his programming of those , not because unfortunately , the spectrum are surfacing a lot . And I could see it in the playground and I could see it , you know , and ah , and there are different like you come along with [name] and he comes down , but we just don't know . |
| SPEAKER1 | 44:13 | So , you know , you're coming . So like , I never go out alone with them anywhere . Because I think about and I sometimes ask about the disability part . |
| SPEAKER14 | 44:26 | Some doctors believe in it , some doctors don't necessarily say what the disability has to causing the disability because let's say you need to go to a supermarket and I park for and Carter such a tantrum to walk him from , you know , the exit of the supermarket to find my car . |
| SPEAKER24 | 44:49 | It's a big challenge . So I told one pediatrician , you know , I know some parents have it . Can we explore ? |
| SPEAKER64 | 44:59 | And I told her it's not that I'm going to use it all the time . It's just for opportunities where I choose not to leave the house because I cannot live with them , because I'm alone with them . She said , no , she didn't believe that , you know , that warranted . That warranted . I think he's right . And I respect I respect your opinion , but I invite you to become more aware sometimes about tantrums of kids . Maybe she might not a day when they don't , you know , when they tantrum , it's like 40 minutes and pulling a nine year old through the parking lot . It's challenging , especially when you have groceries , perhaps where you're pushing a cart and you have the other child , so , you know , it was kind of like something that he has a mental disability . |
| SPEAKER65 | 46:02 | She didn't see any physical disability . |
| SPEAKER66 | 46:06 | So I'll try to get it done . I don't see my point . I don't think want to be . |
| SPEAKER67 | 46:15 | I know there's a few , but when you deal with families that need it , we're more I would say we're more appreciative of the benefit and we don't want to lose it . |
| SPEAKER24 | 46:30 | So we use it responsibly . |
| SPEAKER1 | 46:32 | Right . Know I'm not going to use it if I'm going to the movies a la . You know , it's hard things like that , I , I can't go out because I just I don't I choose not to . |
| SPEAKER12 | 46:47 | What if something happens ? Right . That scares me some something that I can't control . So we we talked about , you know , having this question of like , what what can you do or how can you better improve the quality of care ? What what what do you need from that type of stuff ? And open ended ? I want to show you another way of doing it , just to kind of get your thoughts on this . These are a series of questions that they're not meant for health care . |
| SPEAKER52 | 47:16 | These were designed for the census , but they assess disability status . And so I wanted to see what you thought about them . You know , do you prefer the open ended ? You like , you know , the specific things that you think might be helpful . So obviously , he talks about his deafness , blindness . |
| SPEAKER49 | 47:34 | You have difficulty concentrating , remembering or making decisions , mobility , issues of walking , climbing stairs . So what are your thoughts about these questions ? You find if you were asked this and you answer them , that they would be helpful to you . |
| SPEAKER31 | 47:55 | Yeah , the experience . |
| SPEAKER68 | 47:59 | Yes , I think it will be helpful at first when you read them , when you say death or have serious difficulty hearing you . I don't know if it's me , but I immediately think , oh , this is affection for the other . OK , good to know . And then , you know , I start reading faster . You know , you pay less attention because you write because , you know , maybe not start with the death , maybe move that down to the to the bottom . Maybe start with number three . OK . |
| SPEAKER44 | 48:36 | You know , because they just , you know , yeah , because you're starting with deaf and blind and you immediately associate that with the other people . So , yeah . You know , our number six is when you're when you are preparing the person , this is a section for your physical , mental or emotional condition . |
| SPEAKER49 | 49:05 | So maybe you have like something before the questions that kind of give you an idea of what these are aiming to answer . |
| SPEAKER63 | 49:11 | I think the questions are pretty good . I mean , does that I mean , would those prove so if you said yes to any of those ? What happens next for you ? How can we make how can we accommodate your visit ? |
| SPEAKER56 | 49:24 | So the follow up would be the open ended , where you can give more specifics about why you answered yes . With disabilities , you have a whole array of disabilities , right ? You have people in wheelchairs and you have people , you know , that walk with difficulty and you have . |
| SPEAKER49 | 49:49 | Cerebral palsy policy that are movements and , you know , our bigger wheelchair's as they get older , coming along the . So do you in that sense , you do like these questions in addition to open ended , or would you actually just prefer the open ended ? |
| SPEAKER14 | 50:11 | This this is kind of like identifying that you do have somebody and the open and that is the specific . |
| SPEAKER42 | 50:19 | What would work for you ? Yeah , because we have established you have a disability . What will work for you ? How can how can this practice accommodate your needs to meet the shorter wait times . You know , the rest I can manage or sometimes my son , he gets very emotional and also the wait time that he has outside , he thinks something's coming . What is coming ? You know , where am I going to be taking you know , and he starts getting anxious and he builds anxiety . By the time we go in , he's like he's on level . He's up to here versus when he's already in the room . |
| SPEAKER1 | 51:05 | And it's quiet because outside in the lobby you have the TV going . You have other kids either playing or talking or especially in a pediatrician office . |
| SPEAKER63 | 51:14 | So those other things are good . Well , that is too much for him . All that is playing at a higher pitch for him because he's very sensory . |
| SPEAKER3 | 51:23 | He can get it . He has very sensitive sensory inputs . You know , he he can get overloaded . |
| SPEAKER69 | 51:30 | And then so by the time he goes in , you know , and we start calming him down . |
| SPEAKER49 | 51:35 | Then , you know , it takes and is it is it left to you to calm him down or do the stuff help to . |
| SPEAKER69 | 51:44 | It's kind of left to you or whoever comes to the first thing they do is to take the vitals . So if he's not ready for the finals , then go to the room and she takes the other patient . |
| SPEAKER58 | 51:56 | And then , you know , I come down , either give him my phone or we play some music and then , you know , we try to go back out into the violence . |
| SPEAKER56 | 52:06 | And do you think that that's that that is the ideal situation because you know your son better than anyone else ? Or do you feel like they should play a more active role in because they just can't . |
| SPEAKER69 | 52:14 | Oh , they don't have time . You can't have you can't hire somebody to come because she has to get the other patient ready for the doctor to see them . Like , I wouldn't expect that accommodation . Very nice if just give me the space and I'll come to you and then we can move forward . There are some things you can write . You know , there's some things that we think nobody's going to hire somebody to come . |
| SPEAKER70 | 52:46 | You know , even like a sensor you like , if you have more kids on the spectrum , if you practice alternatives to the way you're saying you see it now . |
| SPEAKER28 | 52:58 | Yeah . What would be in that room ? Like a cushion , like the airport . The [airport] has considerable power . You can see pictures of it . It's like a ball . You know , the lower lights . You know , those sensory lamps , the lamps that have the bubble go up and down , you know , like a sweet girl , but I don't know , depending on what , depending on what the breakfast is , you're providing a speech about speech therapy . |
| SPEAKER61 | 53:29 | But , you know , speech is in and out , in and out of . |
| SPEAKER49 | 53:32 | Depending on what you practice , if you're a neurologist , maybe perhaps that would make sense . You're going to have your your patients wait . All right . So would you say prior to an appointment , you do a lot ahead of time for them ? |
| SPEAKER33 | 53:48 | I mean , you say you'd be right on the on the form of autism . And do you write anything else besides I like some of those no diagnosis . And do they ever kind of follow up us to learn about what that might mean for you or what you might ? |
| SPEAKER14 | 54:03 | I could see I could see them in their attitude , like I could see that , you know , they write it , they know what you know , what they are dealing with . |
| SPEAKER69 | 54:12 | And , you know , they try to see they're trying to do something like a formal thing . Yeah . To , like , get to the point . And , you know , like , dermatologists have never touched them . We have never touched the topic . She knows . And I know she knows because as soon as we get into that lobby , we're in safe hands and . |
| SPEAKER54 | 54:38 | And there are two people , you know , usually coming in , doing all the questions in and out , you know , and then she comes in and then she asks the questions and then says and then she goes down to the level . And , you know , because we have to take off their clothes because , you know , she checks in . So , you know , you can leave and then reject to sit on the exam table . So she checks in , just she goes , fine , that's fine . |
| SPEAKER49 | 55:11 | I can I can see you on the chair here . You're sitting on me , you know , so you can see she's modifying . She will not force him to say , this is my exam table and this is where I need him to be , because this is how I need to check on the patient . |
| SPEAKER63 | 55:29 | So she never asked for anything . She's come over and new things to do that seem to be the work . |
| SPEAKER33 | 55:37 | Well , yes , she has never asked , but she knows so . So that is acceptable to them . So if it works , you know , how do you feel ? I know you've talked about some unpleasant experiences where you didn't return . Have you ever kind of shared feedback in the moments or after the fact to be like , no , that wasn't no , that wasn't the best experience ? Or do you just kind of declined to do that ? |
| SPEAKER70 | 56:02 | I like for the genetic geneticists . When I called to cancel the appointment , I let the girl know . I don't know if she took the note or not . Sure . But I did talk . I did tell her that emotionally I couldn't . Right . And definitely that other nurse you want to give her in your home . Yeah . That he wasn't trying to work on his bedside manner for treatment . I , I've never but when I do hear a parent complain about treatment , I told her , you know , how long you know , I know what you need to consider finding a better somebody better that it's going to fit your family . |
| SPEAKER69 | 56:47 | And I'm not telling you to come over to my neurologist , but I'm just saying maybe it doesn't matter if he's the good or my eminence urology in the city . Maybe that's why the reputation doesn't align with the experience sometimes . Yeah . And maybe he's you know , [celebrity] believes in him . He treated his son . All right . But , you know , he also has you know , I guess he's the director of the [name] Center and he has other places he needs to be that he shouldn't be seeing patients . |
| SPEAKER71 | 57:28 | And I meant to write to [celebrity] about my experience because I wouldn't . |
| SPEAKER33 | 57:36 | And I don't think it's funny . Very important , you know , so so any last thoughts , I mean , that's all my questions . Any last thoughts , something that you wanted to say that we hadn't gotten to yet ? |
| SPEAKER71 | 57:53 | No , just you know , I think I've covered my what my good experiences look like , you know ? And , you know , as my kids get older , it's going to be different . The challenges are going to be different . But for now , I think that it's hard enough to get a diagnosis . So I have to have a little bit more of a , you know . |
| SPEAKER70 | 58:16 | But other than that , you know , I I do appreciate you gathering data and . You know , giving us a little bit of hope and allowing us to participate in this opportunity . |
