# Supplementary material for: Disability disclosure in healthcare settings for individuals with developmental disabilities: A qualitative study of patient and caregiver perspectives
Source: PLoS One. 2025 Aug 7;20(8):e0329328. doi: 10.1371/journal.pone.0329328 (PMC12331114; doi:10.1371/journal.pone.0329328)
Supplement: S1 File — (ZIP) [file pone.0329328.s001.zip › Transcripts/2020.03.05 Interview 22 Transcript.docx]

***2020.03.05 Interview 22.m4a***

| SPEAKER1 | 00:00 | I love you because this isn't the first time I've been following and spreading and growing . Yes , I was very much on the beginning . |
| --- | --- | --- |
| SPEAKER2 | 00:08 | So when I started , like because at the beginning , they needed speech therapy , occupational therapy , yes , they needed behavioral therapy . They had so many sensory issues . They had everything related with behavior about ADA wasn't a thing then . I'm not there yet . |
| SPEAKER3 | 00:30 | So speech wasn't Dr. [name] OK ? And I've always had Medicaid because I'm not working . And so they always have Medicaid . And at the beginning it was a it was nothing . So they said no . Then somebody applied and I got a letter like six months later . Yeah . |
| SPEAKER4 | 00:50 | And they said and now back then , like , OK , they gave me like fifteen hundred dollars for the child to have therapy , speech therapy. |
| SPEAKER5 | 01:00 | OK , then it was little by little like OK because so many therapists starting coming up from [location]. So, then we started going to the [name of children’s] Hospital and that was the new one around here . It was [name of children’s] Hospital back then , guys and [name of 2^nd^ children’s hospital] . So, we started receiving OTTI occupational therapy and speech therapy . |
| SPEAKER6 | 01:29 | Then we kept on doing that . Then we started doing them . |
| SPEAKER3 | 01:34 | Then we changed because it was it was too far , I think . Then we started going to a place that is called [name] Center . And that was around here actually on the drive . And I don't know , in 92 and 96 , I'm OK . |
| SPEAKER7 | 01:51 | I think we were there for so many years . Then later on , we being always , like going to , um , [service] and going to that's why I get all the information from you because I'm been a registered nurse since . Yeah . I don't know , like ten years already . So we started , we went to see the neurologist and all the specialists , the [name] center we went to . Then we found the therapist , the doctor who was trained at the moment . |
| SPEAKER8 | 02:23 | And so he was the one who told me that they will really benefit from my therapy , which is behavioral therapy . |
| SPEAKER2 | 02:31 | And, we said it like they were four and at home and that's at home . Yet everyday at no . Three times a week from five to nine p.m. . OK , so with the little one , I needed a little a little bit more . And at school they do certain things at to the beat one . |
| SPEAKER9 | 02:54 | It's already he had a prior profession . And I'm telling you , revolutionary years , you had a population of the little one , wasn't in the room for so long . So he's been on the out of that type of setting only from one year . But he had the therapies going from my insurance , going to the school , OK , they had Iot of school . They had speech therapy , a school day . Well , right now they're doing the counseling and all that , you know , like you emotional support . Yeah . At school , they do that right now . But at home , that's what we're doing right now is focused on being a therapy . OK , we struggle . That's our struggle . The big one is socially OK . |
| SPEAKER10 | 03:44 | So first I need to get reviewed this informed consent form with you just . Oh yeah . Make sure you're OK with the study . |
| SPEAKER11 | 03:50 | So I just explained to you the purpose of the study . The procedures is just participating in this interview . And we already filled out this questionnaire that I've I have a few questions about risks and discomforts . There really are none is just participating in the interview benefits . |
| SPEAKER12 | 04:07 | Don't worry . I participate in anything related to you . I've done brain study . I've done actually that is ongoing . I'm ready to go public . I'm going on advocacy . I'm doing a training on the management center for Advocacy and everything . And I'm very much up to this . Yeah , I'm feel like here like eight dollars for your time at the end and then confidential . |
| SPEAKER11 | 04:28 | Of course , if you have any information that you want to contact someone about , you would contact [name], and I can provide you a copy of this . Would you like . |
| SPEAKER13 | 04:38 | No , that's OK . I mean , I give you and you have something by email is OK otherwise . Yeah . Right , OK . |
| SPEAKER14 | 04:43 | And then I just need you to sign right here and then put your name and date . Yeah . |
| SPEAKER15 | 04:50 | What is your name from? Very hard to pronounce. |
| SPEAKER16 | 05:08 | Those people have difficulties pronouncing your name all the time . Yeah , I was like this today , the . OK , perfect . So . |
| SPEAKER17 | 05:27 | And then we'll get to this at the . So let's start with all this , and you said you're OK with being recorded , correct ? So tell me about your typical day , like with your two kids . Is it like school ? Yeah , behavioral therapy . Yeah . And that's about it . |
| SPEAKER12 | 05:53 | OK . Well , right now . OK . Right . What's going to change . No , no , no . |
| SPEAKER18 | 05:58 | Because when they were it changed recently because we were doing so many more therapies before . OK , so it was driving here . Driving . They're coming back home in school . Mm hmm . And then therapy at home . |
| SPEAKER9 | 06:11 | OK , so . Right now is going to school because the school is most of the day . OK , they start at nine and then they end up at four . |
| SPEAKER18 | 06:23 | Then we started going out with five and we ended up at nine o'clock . OK , perfect . |
| SPEAKER19 | 06:29 | And who is like coming to your house to do this therapy ? |
| SPEAKER13 | 06:34 | Is it the ABA ? It's OK . |
| SPEAKER20 | 06:39 | The behavioral therapy works this week . There are a few Sivas which are behavioral certified and the day are the ones that designed the program . They observe , they decide to program according to the child . Then they work with an assistant , which is the ABA . |
| SPEAKER4 | 07:03 | That assistant is the one that is taking care of our kids right now . |
| SPEAKER3 | 07:08 | But because they're very high functioning . OK , so they need other type of its basic needs , but we cover it with them . It's a little more , you know , living skills , social skills and this kind of thing . |
| SPEAKER4 | 07:24 | But in other cases they go like the let's say like the lowest level is the team , which are registered behavioral technicians . |
| SPEAKER1 | 07:35 | OK , so those are the ones that I used to have in the past . But since they are more challenging because the you know , based on the plan and everything they need , they have . |
| SPEAKER21 | 07:47 | So [name] is the girl that is coming to my house and she supervised by the coordinator , which so . So we have and they have a lot of support that ECPA is able to observe the things that are going on and then to implement because ideas like to to have the plan like incorporated in every setting , you know , like not just the home , but socially work . |
| SPEAKER6 | 08:17 | They have to struggle . |
| SPEAKER19 | 08:19 | OK , and so in other health care places , you see health care services like you are a hospital setting . Yeah . Um , yeah , things like that . Would you say it your experiences are overall positive or . |
| SPEAKER6 | 08:30 | My goodness , I love you . Really . You care . And the [name] Center . That's why I'm doing my advocacy training in there with them . Perfect . It's it's been like number one for me and I've seen them very they are very pioneering . It is kind of the feel of the spectrum , OK , they are very always reasons . |
| SPEAKER5 | 08:56 | OK , very much in development too because they the [disability service name] is very active , very active in anything related with the spectrum . And I see a lot of programs coming up and coming up and more information and more people like gathering to they're like now there is a very powerful source of information for people with families under the spectrum of autism . I think it's a huge resource . |
| SPEAKER22 | 09:23 | OK , and then what do you think , like the specific examples that make your experience as positive , whether it be your physicians , that your kids already know , the disability that your kids are coming in with , even if they're being seen for something like a broken leg , like understanding that they don't know , OK . |
| SPEAKER18 | 09:46 | [name] , OK , the [disability services], that's what they know about my pediatrician . If he knew when he learned everything because of me . Because you told me . Yeah , OK . Because when they were little , he knows them since they were born OK . |
| SPEAKER23 | 10:03 | Then when they started talking , I got the regular age . I said something is wrong . And now kids later . Don't worry , you know , like I said , no , I'm very pushy . |
| SPEAKER6 | 10:14 | And I was like , no , I know something is going . And then , well , then they say , OK , what do you want me to do ? You know , like he was very combative and he said , What do you want to do ? And I said , I want to referral . I want to go to a neurologist . I want to go see another specialist when I have their hearing tests . I want to have , you know , like I want to know what's going on . Yeah , they help me with all that . And nowadays he's like he's a big fan of me because I , I was very observant . And he , you know , he's been he he goes like , you are my role model . Like , OK , now he encourages other moms because again , like then like ten years ago he was in big thing . It wasn't that much awareness as it is right now . So now he is very much like , OK , you know , like and he he goes like he mentions me and my kids because now he sees them and because we start mean their pensions . They were very little . That made huge progress . |
| SPEAKER10 | 11:10 | And you think it should be something so far in your case you had to bring it to his head . Oh yeah . That oh you that I was wrong . OK , let's say you had switch pediatricians . Do you think this is something that should be asked before you even enter like the physician's clinic ? |
| SPEAKER24 | 11:27 | Is it something that should be acting like a screening questionnaire , like an intake form ? |
| SPEAKER6 | 11:30 | No , let me tell you a couple of experiences I had when I took him to the emergency room a couple of times because of an ear infection or a high fever or something like that . They asked me , OK , they ask me , they say , is it like the like the nurse ? |
| SPEAKER23 | 11:48 | Yeah , well , yeah , that's the thing that westerner's she told me . Is he does he have any condition then . I said yes . |
| SPEAKER25 | 11:58 | Why . Because like because he's hyper sensitive and he's over reacting over , you know , like the little things that I'm doing . Yeah . |
| SPEAKER6 | 12:07 | I'm like yeah but nobody is like nobody , I mean nobody does nothing but yes . |
| SPEAKER16 | 12:13 | And I will be very much easier because if they know , you know , they can have some considerations at least like in the way to approach . Yeah . Yeah . What , what are those considerations . Do they put them in a quieter area . No , no , no , no , no , no , no , no . After that they just left us alone in the room and they should have come back . No , then she came back and then she she changed like this tone of voice and everything she says she was explaining to him , like , OK , now I'm going to do this . And , you know , like she said , OK , yeah , yeah , yeah . |
| SPEAKER26 | 12:52 | So and also , like , hypersensitivity has to do with , like , noise , hustle and bustle in like the hospital emergency . So overwhelming . Yeah , overwhelming . So like do you think once they became aware of him being on the spectrum they like changed their approach to like , oh , making sure like not too many people are seeing him so he doesn't get like oh no , no , no . Things kind of like proceeded as normal . Yeah . |
| SPEAKER27 | 13:15 | Besides like explaining . Yeah . OK , makes sense . And then do you think that's like so she had to like she noticed some behavioral differences and then she asked you in person like do you think that's something that should be like on a form that you receive like immediately type form like the same one . |
| SPEAKER6 | 13:34 | Like yeah that will be easy . And I think any way they review the history before taking him , like before taking him up in the room . |
| SPEAKER19 | 13:45 | Yes . Because you might be comfortable answering , but like maybe some other people aren't comfortable and would rather have it like not face to face but rather on the form or something . |
| SPEAKER6 | 13:53 | Well I was that's why I asked her , like , do they need to know , like she has a high fever , do they need to know ? But then I realized because I didn't know , I wasn't thinking like , you know , I was there because they had fever and I was worried about him . And then , you know , they cause exact I didn't think that that was going to interfere . And nothing nothing . I wasn't making any connections of anything . And I said like , oh , wait . I mean , like , tell me , what did you notice ? Yeah . And then when she said about the behavior , I said . |
| SPEAKER10 | 14:23 | You're telling me , right ? OK , so now going forward , do you think even if it's something unrelated to being on the spectrum because like that's kind of handled with the behavioral therapy , like if he is going for maybe like a stomach bug the next time , would you want them to know that like that time around because it might change their approach ? |
| SPEAKER23 | 14:41 | OK , so what ? Because behaviors aren't really . Yeah . And now they're they are very much noticeable because they're big , you know , when they're little . That's why I asked him , how do you notice . |
| SPEAKER12 | 14:55 | Yeah . Because little kids behave , you know , like they are now like very well behaved . |
| SPEAKER28 | 14:59 | Yeah . They're learning and everything . And now that they're bigger , every little thing is like magnified because of their size and their physical appearance . I don't think that I have to explain every single time . I have to say , OK , just let me explain . Like , yeah , we do this . We are on the spectrum . Does you see anything odd ? You know , like even I've been working super hard , like I'm telling them , like with social stories , like we're going to the doctor . This is what he expects . We are supposed to be quiet . We are supposed to be cooperating because he's going to help , you know , like things like that . This is what you're telling your kids . Yes . OK , makes sense . |
| SPEAKER19 | 15:39 | OK , and then like , um , so we're the staff and health care providers you interacted with , aware of their disabilities before you had arrived ? Oh , no , no . OK , so you always had to make them aware of it . OK , and how and they became aware , like I think you kind of like already explained , but like how did they become aware of your disabilities . It's always word of mouth behavior . |
| SPEAKER6 | 16:02 | Yes . Like no to them is because the . Yeah , yeah . |
| SPEAKER28 | 16:07 | They , they kind of come to conclusions and they asked me then and like , is there any condition or anything that I have to explain . |
| SPEAKER29 | 16:17 | And you kind of also mentioned this , but like what accommodations , if any , were made to provide for a more positive experience was there . So usually it's like a bunch of people coming in and out , like I said . And you see multiple people along the way . In your case , did you maybe have one person that stuck by your side , like one nurse that facilitated . No , no . |
| SPEAKER6 | 16:37 | OK , that would have been much better , you know , like . Yeah , because for them it's like to have one person , especially if they if they have connection somehow . Trust me , I've been traveling all Miami in different schools , so many schools every single year because of that , because one person making connections makes a big difference . Yeah , it's a more comfort . Yeah . Instead of having like a fight every single day . Yeah . To everybody . Yeah . Yeah . Gotcha . |
| SPEAKER29 | 17:03 | OK , and what actions if any do you think should be taken to ensure a more positive experience . So whether it be a quiet or emergency setting for an emergent case like a stomach bug or specific assistive technologies like that will be very helpful because they always get entertained with something that is . |
| SPEAKER4 | 17:29 | Yeah , if you don't have a cell phone like OK , like Muslim people , we do have cell phones . Right . But yes , they get very entertained with with assistive technology like something to that . It's really in my case , it works really well . OK , keep them occupied . Yes . Everything . |
| SPEAKER14 | 17:45 | Huh . OK , and now if we can speak to any negative health care experiences , you can if any . |
| SPEAKER30 | 17:52 | Have you had any that like , oh , they didn't understand your children's condition and it made for like an unpleasant experience on your partner , on the kids for . |
| SPEAKER13 | 18:03 | Well , it's funny because it has to do with pediatrician . OK , but it wasn't in a health care setting . That's one . Yeah . And . |
| SPEAKER1 | 18:14 | I wish I will know the name of that person because she was yelling at me , you know , my kid in a park in front of everybody , OK , and saying that she was yelling like , if that kid if my kid was autistic because he didn't understand a word that what she's saying and there was a baby around . So she was like , I don't maybe asking him to stop or something . |
| SPEAKER16 | 18:36 | He didn't . And I was with my other baby because I have them both . Then it was sitting with my friends around the park . Then she was yelling so loud that I couldn't even stand up from the grass and I got sick . My friends had to take me home . I couldn't even say anything . It was one of my friends who came to that , to the doctor . She was a pediatrician because she said they have come to talk to a person like that . You don't know what is going through and what kind of professional are you ? Because she said , I'm a pediatrician . I know like he is optimistic or something like that . |
| SPEAKER30 | 19:10 | So this just in like a public setting , not your kid's pediatrician , you know , and . |
| SPEAKER31 | 19:16 | Oh , no , no , no , no , no , no . |
| SPEAKER6 | 19:17 | My pediatrician has been always very like again , very cooperative and very on my side of his being adorable . I mean , I love him , but yes . Like that kind of thing . And I've seen a lot of now it's different . But back then , like a lot of it . |
| SPEAKER32 | 19:38 | No , no . I mean , he's a sensitive issue for us , like parents or caregivers . It's our life . Yeah , but for the doctor side is another patient . |
| SPEAKER28 | 19:49 | And I know it's now like as important , you know , like as it is for us on the other side . Yeah . And and in this training , I've seen it because I was doing the training with doctors , a lot of them . |
| SPEAKER32 | 20:04 | And they see it like , like a waste of time , maybe learning about how to cooperate with the family or how to . |
| SPEAKER2 | 20:11 | It's very little deep because they are very under pressure because of time , because of the money , because of insurance , because of these and that , you know , that's their profession . I understand that . But yeah , it's very hard , like especially when delivering news or something , it's not going well . It will be very nice to have , like , at least like some kind of , I don't know , violent moment or something to explain or to let you know I know as a parent or caregiver or a person involved and what's going on or so it's more like , OK , how to deliver that news in a more flexible thing . |
| SPEAKER16 | 20:52 | I mean , to me , it was an otti at the moment . She said she gave me a paper piece of paper . I'll draw this draw , I don't know , length . Yeah , I didn't do it . Then she took all these things and I was like , what ? Just like very graphic . Yeah . I never forget the home . And I was like one . |
| SPEAKER33 | 21:12 | And then then they started and they said , like , what's going on ? Then I said in my own research and I said , it's like back then . |
| SPEAKER27 | 21:19 | So it's more like sensitivity training physicians . And Otis in that case need to be more sensitive to your teeth and everybody . |
| SPEAKER6 | 21:27 | Yeah , I have any therapist . |
| SPEAKER2 | 21:30 | I mean , no , no to not no penis is no . That is just like awareness . Yeah . We are all different . Right . And some people are more than others . So just like try to observe what you see and be more sensitive . But in that way I'm , I'm not saying that you need to cry with a parent or with a no , but you just need to like try to have a better approach because to me it was terrible . Yeah . |
| SPEAKER16 | 21:59 | No , I cried for a week trying to find out an answer , and then I start asking myself , I who is hurt ? Like , why did you tell me that ? Why now might be that reason why not . And that's when I started like Journey looking for so many doctors and and talk to other people because I was and I never go back . |
| SPEAKER27 | 22:15 | A lack of transparency in the process was like , oh , the hottie just told it . Told it to you ? |
| SPEAKER16 | 22:20 | Like bluntly , I think it was wrong . Yeah . Coming out of there . Yeah . That's not going to give you too much concern . I think she had to put it at least until the pediatrician or the main physician , the one that takes care of us . Yeah . They say , OK , look , at least we know him . Yeah . But she was evaluating him and she said , oh he's OK . There was no like showing a ulcer . No . OK , zero . |
| SPEAKER27 | 22:50 | OK , so it's more like tone of voice , the language . The people like the language that the health care providers use , like whether it be an OK to your physician , be more understanding , show concern . |
| SPEAKER12 | 23:02 | And yeah , I think that will be more human . Yeah , of course . |
| SPEAKER27 | 23:07 | That's more like it's not any other patients treated us like and you like put a . |
| SPEAKER13 | 23:14 | And keep your word evil somehow , and I'm like , you care , yeah , you care because it's your profession , but you can't . |
| SPEAKER10 | 23:20 | So you saw that first with the hottie . |
| SPEAKER29 | 23:22 | Have you experienced that elsewhere with any other , like , doctor , emergency department nurse , a nurse , a technician that that the one on the experience that I already told you . |
| SPEAKER18 | 23:32 | But she was more . Like she just asked me , like on the side , like very respectfully , any other type of setting , no , I'm being very defensive ever since . Yeah . Like so I go three steps ahead before anyone says anything . |
| SPEAKER6 | 23:49 | You could make them aware of them . Yeah . Yeah . OK , yeah . Makes sense . OK , perfect . |
| SPEAKER19 | 23:58 | So do you think there are any aspects of your disability , of your kid's disability identity that your health care provider may not be aware of ? So what that means is like , oh , they understand autism spectrum disorder , but they don't really know the full breadth of like what that entails . Like it involves behavioral aspects , speech . |
| SPEAKER6 | 24:16 | They don't know . I think everybody's learning . Everybody's learning . |
| SPEAKER28 | 24:19 | I was the only person that I so really , really like knowledgeable about this was Dr [name] at the [name] Center , OK ? He was the one who gave us a very excessive evaluation , like four hours for each of my kids . |
| SPEAKER23 | 24:39 | And he he made sure to evaluate every single card , like physically , emotionally , every , you know , every everything , every part of the check that that was needed to do it , to be done . |
| SPEAKER29 | 24:55 | He did it . Do you think that should be par for the course for every physician should be that thorough or one physician and then they should relay that type of information ? |
| SPEAKER3 | 25:05 | You know what I mean ? We're going there . You're going we're going there because it's going to be needed , OK ? Every physician should be that through every session . But if you are involved in neurology , if you are involved like pediatricians now , they have to be more knowledgeable because that is the very perfect time to catch up with anything . |
| SPEAKER33 | 25:27 | Yeah , and the sooner the better . |
| SPEAKER28 | 25:29 | Yeah . And at least pediatrician . Yeah . Yeah . Because when they get when you are diagnosed at a later age , it's a different story . |
| SPEAKER29 | 25:39 | So especially the physicians that are kind of related to the autism spectrum , like neurology , otti , hate speech therapy , all those . Yes . |
| SPEAKER6 | 25:49 | They should be more normal . Yeah , absolutely . And that could be done with a more thorough screening when you first visit . |
| SPEAKER29 | 25:58 | Absolutely . OK , and what about like your pediatrician ? They like they need to be referred to something unrelated to autism spectrum , like let's say it was something like gastrointestinal related , let's say , and the physician that the pediatrician had to refer you . |
| SPEAKER30 | 26:18 | Would you want your pediatrician to , like , send all that information on your like related to the autism spectrum disorder ? |
| SPEAKER4 | 26:25 | But also you want that information to me , never had a problem like sharing information between like the medical care for my kids , never had a problem with that . |
| SPEAKER25 | 26:35 | And I think that helps actually , because it makes my life easier . Yeah . |
| SPEAKER28 | 26:39 | Whenever they ask me for something everybody already know , like if I am doing this therapy pediatrician knows I'm doing this survey because he receives like Colbys or he knows what am I doing . OK , so I don't have to be going to be there and explaining there and explaining here and explaining someone else . And so it makes my life easier for shared information . |
| SPEAKER30 | 27:00 | So that brings me to my next question . So your role as a caregiver , do you think that the health care providers should rely on you a lot ? Yes . Or do they have they relied on you too much , too little ? Or is it like just the right amount and your experiences ? No , I think it didn't start that way . |
| SPEAKER6 | 27:21 | And he was like , no relying on anything , you know , because he didn't see it that way . |
| SPEAKER8 | 27:30 | But then after I said it with all the referrals and that well , the after diagnosis , after one test here , another test there , another visit to the doctor , to another specialist , you said you're totally right . |
| SPEAKER6 | 27:46 | Like , what are you doing ? So he helped me on the on the medical care , like the regular one , because it's not visible like again , for them it's just behavioral and neurological . |
| SPEAKER4 | 27:58 | Yeah , I think . But other than that , he is very supportive and he is very like after that . Yeah . Let me point before he didn't know me , you know , like he didn't know anything , he just were checking a patient . Yeah . But after that he realized like how much I was right because I was the one seeing it . Yeah . So off today he goes like no nowadays he's like again like he's a big fan of metal and that I'm out of a lot of pademelons in there . Yeah . |
| SPEAKER27 | 28:30 | And takes your input more than let's say . Absolutely . A mom without a kid with developmental . Possibilities . Yeah , so what what types of responsibilities does the physician kind of put on you like is it kind of like speaking between different like you're the one to be responsible , to speak between different health care providers that your children might see or like ? |
| SPEAKER20 | 28:54 | What types of responses ? It depends what I find . You know , what I'm saying is like , if I need a referral , let's say for Otti the first I mean , for the first time , because you see Vrba , until the doctor sends different parents to medicate . |
| SPEAKER4 | 29:11 | OK , so I needed the doctor to do it . |
| SPEAKER3 | 29:14 | And he told me , OK , what do you need ? Again , like his very much knowledgeable about my case , you know , our life , our you know , he's seen them since they were very little and and he's just like , OK , yeah , these works . |
| SPEAKER6 | 29:31 | And you're doing it right . Yeah . And they're growing up and developing and everything . So he trusts your about . Exactly . OK , gotcha . |
| SPEAKER22 | 29:40 | OK , so here are some things that maybe other people have complained about . So it's it's no secret that people with developmental disabilities experience health care disparities and in receiving health care , if related to their condition , are not related to their condition . Sometimes they just receive like less quality care because , oh , they're not sensitive to people with autism spectrum disorder being hypersensitive . So , like , they just throw them around from room to room . And the patients I'm comfortable with , say , or I've seen it . Yeah . |
| SPEAKER16 | 30:22 | So like there it's no secret that there is a lack of sensitivity and it results in lessening lack of knowledge , lack , to be honest with you . Yes . |
| SPEAKER24 | 30:33 | And it results in like less quality care . Yes . So some of those areas could be the physical environment . So whether it be having a wheelchair ramp in a hospital for people who need to or wheelchair accessible places , having assistive technologies like , is there a weighing scale for people who are in wheelchairs versus a weighing scale for a person who's not in a wheelchair , just can stand on a wing scale , you know , and then screening . There's a lot of problems with screening . Sometimes you go into the , like , physician's office and then they're made aware of your disability . But that could have impacted the way that they prescribe you a treatment . The language that the physicians use , are they insensitive ? Like are they saying disabled person instead of person with disability , like the right tone , the right person , first language and then . And then . Is this is the language problem on the part of the physician , on the part of the nurse , on the part of the receptionist , staff , receptionist , the receptionist , and then are they dismissive ? So , oh , you have nothing to worry about , like , don't worry about it . And they're not really hearing your concerns , like things like that , an institutional policy . So like of all these items you see , you see problems in those aspects . |
| SPEAKER18 | 31:58 | Oh , does it come like the dental care like that . Yeah . Yeah . Examination Steidl . Because especially there they don't know . And for them for the dentists , I have to tell her , like just because they put out a show and everything and it's it's very complicated . |
| SPEAKER8 | 32:20 | She had access to it . So I have to make a huge preparation . But still , they're not used to it . Comfortable . It's very unpleasant . And and I have to explain . But no , they don't . Oh , OK . |
| SPEAKER13 | 32:37 | Yeah . Because they didn't go that way . No , no , no , no , no , no . That's the only part that I think that it's like it doesn't matter . |
| SPEAKER27 | 32:45 | Like if there is a hypersensitivity maybe they might put like earphones on to like get rid of the sound . |
| SPEAKER13 | 32:50 | Yeah . Or something . No or no . Because one of them he was , she was checking on something and threw it out . But then she was so disgusted . But then , you know , I was like , oh my gosh , I'm so sorry . I'm so , so sorry because of the hypersensitivity , you know , like so maybe you can let him get used to the flavor or something or just be a little more patient again . Time is again . Yeah . This kind of issues . Yeah . That as a health care . Yes . Because they need to kind of like to relax and to get used to it like that . It's very hard for them like to go somewhere and then then get the check up like that or whatever they're going to do . Yeah . And they're terrified of medical things because they can be painful and going like to take the shots . It's it's a thing now with the pediatrician , they do kind of a trick because the nurse goes like , OK , I'm going to disappear when the time of this comes , I'm going to come and find him . |
| SPEAKER16 | 33:46 | But by yeah . So , you know , I appreciate the fact that the president is never and they have a very hard time . No , no , no . OK , so maybe I always put in the intake . Yes , they are a bit of you know , like it really . They don't make anyway . No know . OK , that's interesting . |
| SPEAKER29 | 34:08 | Maybe something that they can like another like advocacy type of thing in the future , huh . Help those people out . Yeah . OK , so um . |
| SPEAKER11 | 34:20 | So from what I understand , you're comfortable with sharing your disability status with health care providers . It's not something that you're hesitant to share . I know some of them . Um , and where does this comfortable nurse come from ? Is it because you'd rather than be aware them unaware you'd rather them know so that they can change their style of practicing ? |
| SPEAKER13 | 34:44 | I yes . I rather have them to be aware , you know , like they know because that's what I feel like . They don't really know what they're dealing with . Yeah . And it's OK . |
| SPEAKER31 | 34:56 | I mean , like I understand that they don't know because it's very new . It's coming up , it's spreading out . |
| SPEAKER2 | 35:01 | And actually I've had this conversation with so many people like this throughout the school system . Yeah . Because they don't know . Yeah . So they were literally telling me that they couldn't have my son at school . I said , no , you have to take him because that's the right he has . Yeah . I'm going to do what I have to do . You're going to do what you have to do . Yeah . |
| SPEAKER32 | 35:20 | That was of every single day problem . And then I said , listen , there are a lot of kids coming . Yeah . Behind me . I'm telling me that was like a prophecy because I was . Twelve years ago . Yeah , eleven years ago . |
| SPEAKER2 | 35:36 | And it's happening . Yeah , exactly as I said , it is happening is a huge amount of kids coming under the spectrum and more people realizing , you know , like because of the awareness . Yeah , more people like understanding what's going on . |
| SPEAKER31 | 35:52 | So , yes , just just the awareness needs to be needs to be more I don't know , it goes little by little . |
| SPEAKER34 | 36:00 | But on the opposition side , I think that it's a very little amount like and I've heard it because I've been there doing this training like they just receive like eight hours or something . And training about these abilities are especially . |
| SPEAKER16 | 36:16 | And these abilities of at all . Yeah . So the spectrum of autism , maybe two hours or something out of nothing . And it's a huge world . Yeah . |
| SPEAKER2 | 36:26 | And so I mean , I will be beneficial for a lot of physicians just to get more knowledgeable about that . Yeah . Makes sense . OK , so . |
| SPEAKER30 | 36:42 | So this is kind of what we have already kind of discussed , but in a more like ABC kind of manner , I'm going to ask you , so what suggestions do you have for appropriately , appropriately identifying people with disabilities and their needs within the health care setting ? So how should individuals be asked ? What would it be ? A question , face to face computer , an intake form ? What would you prefer face to face to face ? OK . |
| SPEAKER35 | 37:10 | And then when should they be asked ? Should it be before beginning ? At the beginning , like before they even see their like at the receptionist desk . Yeah . You just just like , OK . Is there any condition that you should we should be aware of just before seeing the doctor . Yeah . The doctor's aware before . Absolutely . Yes . And then where should they be . Should it be in a more private setting . Yeah , well , you don't have to yell in front of everybody . You can't ask because they they call you to do the intake and everything . So that could be part of that . |
| SPEAKER2 | 37:41 | Like but just , you know , when they call you to the counter to sign or whatever , like , yes . I was there any condition that we should be aware of . Yeah , that would be perfectly covid . OK . And then . |
| SPEAKER30 | 37:54 | And how would you expect a health care provider to react to someone who discloses they have a disability ? Just you said your knowledge , lack of I mean , more awareness , sensitivity , those types of things , or at least more patient patients . |
| SPEAKER36 | 38:09 | OK , that's a good one . Mm hmm . |
| SPEAKER13 | 38:11 | OK , because with kids , it's very complicated . And , you know , you suffer as a caregiver with , you know , like you want to help them , but you're going to help your kids . Do you want to you're right there in the middle and it's super hard . Yeah . I had so many episodes when they're crying and the doctor , like , waiting for me to manage the situation . Oh , my gosh . Yeah , very hard . They were waiting for you to manage the situation . |
| SPEAKER27 | 38:36 | OK , so maybe their help in aiding your visit , like . |
| SPEAKER35 | 38:42 | Yeah . Not just leaving it up to you . Exactly . |
| SPEAKER13 | 38:45 | Because they don't know what to do and everything because of my ability and I understand that . So but if they know . Yeah . They can use any strategy or play a little bit of play therapy or anything , you know , like to add therapy . |
| SPEAKER34 | 39:00 | That's interesting . Yeah . Yeah , yeah . I know there are so many therapies related to sensory issues that can be really used . |
| SPEAKER37 | 39:09 | OK , a healthcare setting . OK , so let me ask you about this specific questionnaire . |
| SPEAKER38 | 39:19 | This isn't something that's like an intake form at in a health care setting , but it's more what the census asks about disability status . |
| SPEAKER30 | 39:28 | So are you deaf or do you have a serious difficulty hearing ? Are you blind or do you have serious difficulty seeing even one wearing glasses because of physical ? I'm kind of impressed . I'm reading this upside down . |
| SPEAKER16 | 39:41 | Yes . Because of a physical , mental or emotional condition . |
| SPEAKER39 | 39:44 | Do you have a serious difficulty concentrating , remembering or making decisions ? Do you have serious difficulty walking or climbing stairs ? Do you have difficulty dressing or bathing because of the physical , mental or emotional condition ? Do you have difficulty doing errands alone , such as visiting a doctor's office , shopping or office or shopping ? |
| SPEAKER38 | 40:01 | So do you think that these questions are kind of all encompassing ? |
| SPEAKER12 | 40:05 | Does it kind of assess what disability your kids have fun ? But do you think this is an adequate questionnaire ? Yeah , yeah . Because , I mean , like , again , I'm the caregiver . Like , it will it will make it more difficult , like emotional condition , like mental or emotional condition . That will be my my case K3 . And yes , they have made a lot of . |
| SPEAKER18 | 40:32 | Busiek visits to the doctors are things very difficult . But do you think there should be more specific questions for invisible disabilities ? |
| SPEAKER4 | 40:42 | I think that one covers , unless there is a specific interest on knowing if the because , again , spectrum in my case that it's not physical , that is not visible , I will go under mental and emotional foundation . So when I read this , I will say I'm covered . Yeah . You know , but nobody will know if they're under the spectrum of . |
| SPEAKER14 | 41:05 | Yeah . You know what I'm saying . |
| SPEAKER13 | 41:06 | Like , you need something that kind of addresses mental and emotional to cover your your age should be a is the only like a very specific question like as like again you're asking here like people who are deaf or do you have serious difficulty hearing I under the and . OK , so that should be a blunt question . Are you part of the spectrum . Mm hmm . |
| SPEAKER14 | 41:33 | Yeah , because the spectrum . Itself , it's a huge world . Do you think caregivers like you , like caregivers of people with ASD , would agree ? Like would they be would they be offended , people ask ? |
| SPEAKER4 | 41:47 | No , no , no . Because it's it's a question . It depends on how do you act ? Yeah , I don't like the term . Like I say , he's autistic . No , no , no , no . OK , yeah . That end of the spectrum . Yeah . |
| SPEAKER5 | 41:59 | And it doesn't mean that because all symptoms are different and they can be very severe symptoms , they can be like no visible and more mental or emotional or behavioral problems . So yes I you are your are a relative in this case . Yes . Because we are the caregivers under the spectrum and are the spectrum of autism . |
| SPEAKER13 | 42:27 | Yeah . Because that , that will cover everything . Yeah . Autistic is a very I think it's improper because it's is a bunch of things , you know what I'm saying . And chose me . I've been studying this like for very long . Yeah . So yeah . It's not just like when you're didn't know you had a bunch of things under that umbrella . |
| SPEAKER30 | 42:48 | OK , I think we're done unless there's anything you'd , you'd like me to know specifically . No . No nothing else . |
| SPEAKER13 | 42:55 | Yeah . Yeah . But it's very like this question . It's very , I think that Yeah . I think that bluntly make Yeah . You part of this I think OK that will make everything easier because then people who now then like the doctors , the reception is the people behind the health care visit will now . Yeah . And hopefully do something about it at least consider it . Yeah . You know . Yeah . Makes sense . |
| SPEAKER16 | 43:23 | I'm not opening it . OK , here you go . Raise your fifty dollars being here and then I just need you to initial right here on the reimbursement . |
| SPEAKER13 | 43:37 | Not how long does it , though , dunno how long does it take the study ? |
| SPEAKER40 | 43:43 | Its kind of ongoing . I would say we're kind of right now like working on collecting this information of qualitative data . |
| SPEAKER13 | 43:52 | And then we're hoping to find ways to make the changes on the on the health care . |
| SPEAKER40 | 44:00 | So, we're trying to , like , create a 30 minute type of video course for . |
| SPEAKER37 | 44:10 | That's amazing . That's , of course , very much needed , of course , for physicians to take to kind of make them aware of these situations , you know ? |
| SPEAKER6 | 44:18 | So that's great , because this is part of what I'm doing at the same time . And then again , I'm starting with doctors and residents in the outreach and advocacy training that you're doing . |
| SPEAKER4 | 44:31 | Oh , yeah . So those kinds of things you learn , it's basically like [name] , I'm on the other side . I'm not on the medical field , but more like all the students are in the medical field . Yeah . So that's what I'm saying in psychology as a pediatrician and ideologies I learned . |
| SPEAKER41 | 44:47 | Yeah . So yes , they and they are all very surprised . |
| SPEAKER32 | 44:51 | When I was sharing my stories they were like , oh wow . You know , like those kind of things . So that's why I think it's very important for them to learn . Yeah . You learn . Yes . And , you know , I've kind of changed their perspective . Yeah . |
