# Supplementary material for: Disability disclosure in healthcare settings for individuals with developmental disabilities: A qualitative study of patient and caregiver perspectives
Source: PLoS One. 2025 Aug 7;20(8):e0329328. doi: 10.1371/journal.pone.0329328 (PMC12331114; doi:10.1371/journal.pone.0329328)
Supplement: S1 File — (ZIP) [file pone.0329328.s001.zip › Transcripts/2019.10.22 Interview 13 Transcript.doc]

# **Interviewer:** **Alright, so it's on record and just to confirm, uh we went through the consent process and you agreed to participate and be recorded.**

**Person:** Mhmm.

**Interviewer: I just a verbal yes, will do.**

**Person:** Yes.

**Interview: Alright, thank you so I'm gonna ask you a few questions. So just to start off, thinking about all your experiences in healthcare doctors all different types, nurses, the whole experience would you say you've had good or bad or good and bad experiences?**

**Person:** I don't have a right answer for that.

**Interviewer: No? Tell me, tell me, tell me more.**

**Person:** Because I feel like if they've been with people with a lot of whole problems that make is they should know more or have more experience, of what to do with them and how to work with them and socialize with them.

**Interviewer: Okay, so it sounds like okay so you just gave me a lot of stuff right there, so let's unpack age that so... So you're telling me that they don't have enough information about how to interact with you and how to communicate with you**

**Person:** Exactly.

**Interviewer: Okay, so tell me, there's a few different things I wanna learn more about there. So, what don't they know that you think that they should know?**

**Person:** It's like they think they know what's wrong with me, but they really don't because I'm the one sick and so they don't see the pain or the pain that I’m feeling. ... And feel it because when I, if I go in the hospital, or I go to the doctor, they do test whatever they're gonna tell me that everything is fine, no... Everything is not fine because I'm still sick and there's something wrong and it's why I finished investigating was wrong with me, I will not stop it because it's my home and it's my life, I'm not gonna run my life over somebody that doesn't wanna help in the way that they should.

**Interviewer: So, so they run test and the test results save that there's nothing wrong and then they go by that. But you're saying that they're still something more so and so is it because they don't understand about your condition-**

**Person:** Exactly.

**Interviewer: but you're saying that... But they think they know so they think they know, but they don't really... Because they're not asking you questions. Or what, what should they be doing that they're not doing to figure it out to learn more?**

**Person:** For example, I already been, this year to times admitted in the hospital this year, for the same thing my whole body was rolling from head to toe. I am still going through the swelling of my legs, my upper body already got back to normal. But my legs are still swollen and we don't know if it's a real... or something wrong with the vein or the blood, or something or to be my kidneys, because I only have one kidney functioning from birth. So one, I have no circulations and no moments and I don't feel nothing from the waste staff so I don't know what's really wrong with... So if you're a doctor, the way that I see it, you by just looking at me, or in tune, you shouldn't know what's wrong with too and the test should come out saying that there's something wrong because there has to be a reason why my legs are getting stolen. They’re not just gonna just gunna get swollen just for the fun, the...There's something wrong. So until you finish investigating, was wrong, I would not stop coming here for the hospital. That's how I see and I will not leave and I will not et discharged from the hospital until everything is done. That’s just the bottom line of how I am.

**Interviewer: Mhmm. So, so tell me more about that. So let's say they run whatever test that they think they should run they get the results to say nothing is wrong. But you're saying that, "Hey there's still a problem, what happens next, how do you... What do you do?**

**Person**: I keep going until I get an answer.

**Interviewer: And how do they respond like what do they do next in that case,**

**Person:** I keep doing more tests until they figure it out and do they normally figure it out or in some people, some doctors do yet, but somethings don't because they, on the way that I see is that they don't know the... What to say to they may have an answer because of the results, but they won't give me the right answer. So in my case that's what I'm thinking that that's why they're saying that there's nothing wrong with me because they don't wanna tell me the actual true of what's really wrong. So, I'm in and out of hospital, every day I’m in and out of doctors every day, and now I have to go back to the doctor to have my heart checked because of the swelling of my legs, because I don't know if it's the blood of my heart that's not working well or what's going on.

**Interviewer: So you said that they don't know how to communicate with you. Tell me more about that, what are they doing that is not good, and what should they be in that the way they should?**

**Person:** And they're not giving me answers the way that they should and they need... That's why they became doctors or nurses. They can help others, a need because yeah, they're... Well, they're doing whatever they gotta do, but they don't understand why there's a patient sick, or why the patient is in bed all day. So if you don't go test and you don't figure out what is wrong, how are you gonna know what I should be wrong with the patient what if the patient is in bed, and is having a chest pain, or a heart attack or whatever, but if you're standing and you don't do nothing, you just gotta tell the patient. Oh okay, no you're fine, there's nothing wrong. The test is fine. Because if you do the test and you're saying that this is fine, and the patient ends up having an actual heart attack and dies, you're responsible, you're responsible for that person, is that he... Because you didn’t told the patient what was really wrong with that, right. So that's how I feel, and that's how I see it.

**Interviewer: So, when you first go to a doctor say someone that you haven't seen before, what is that in that first interaction, like what questions do they ask you? Do they ask you for example, why are you, why are you in this chair?**

**Person:** Yes, What I'm here for, why I am in the chair and what's the condition that I have and a whole bunch of questions that I have to do with my health, and if I would be okay with that if I have to sign for papers for them to do other types of tests that I've never done and all of that.

**Interviewer: And what do you tell them when they ask you, "why are you in the chair”?**

**Person:** I tell them that I have a health issue which is called a spina bifida. We say it's ‘spinabifida’, but it's actually two words, but we just break it down, to ‘spinabifida”. And it's something wrong with my spinal cord since I was born, and then I have a, uh a (inaudible) in the left side of my brain, that's because I was born with not enough oxygen on the left side of my brain and that caused me to have a lazy eye also and to having the learning disability.

**Interviewer: And when you, when you tell them that, do they seem to know what that means? Do they understand spina bifida?**

**Person:** Some people do, some people don't, because they either they know the patient for so many years, or they've done operations on that patient for so many years. Like my doctor that I know, since I was born, works at (hospital). His name is (name) , and he's done my (inaudible) over seven times or reading I'm thanking God that I haven't had the operation anymore. Because he saved my life, I almost died and he came and he saved my life from dying because he only took one good, look at me and he knew automatically what was wrong with me.

**Interviewer: And how do you think he knew that?**

**Person:** Because I was all swelled up. My whole body was swelled up, but my face was actually more swelled up... So my face being actually more swelled up, He knew automatically that it was the"pupation that he had to be removed.

**Interviewer: so it was more about that medical condition more so than the spina bifida that he just had experience with.**

**Person**: I had 17 hours of spina bifida surgery. Yes. 17 hours. And I’m still alive. Incredible that I'm still alive because some people, some people don't appreciate what they have, they don't see what they have, and people think that just because they're sick or they're in the wheelchair they, they can’t do anything. Okay, I understand that most people that are in wheel chairs that they can’t do anything, because they have Down syndromes or a public whatever, and they can't do anything for themselves, and they have hope, but there's others that can move around and they can do things on their own, they can go out to the community, meet other people do have their own actual life, and not just be stuck there thinking that they can’t do anything.

**Interviewer: So do you think that that perception that mentality is shared by the doctors and the nurses that treated you in the past?**

**Person:** Mhmm.

**Interviewer: Yeah? And how do you think that that affects the health care that you receive?**

**Person:** Live right now in a group home I just moved out of my mother’s home. And I live in a group home, so I'm getting taken care of by the estate now and I'm doing pretty good here. I've been living there for a year and half, already, and I'm gonna go now on December 17, to two years of living there with them.

**Interviewer: Okay, so would you say you're saying it's a good experience that you think the people there that are caring for you there don't feel that way. About the fact that people in a wheelchair, for example, can't do anything?**

**Person:** Yes, and no because some people... I wanna be on top of you all day long or you're trying to do something and yes, they're trying to help you. But you're trying to do it on your own so that they can see like for yourself. You can see that I can do things and I don't have to depend on you, all day long and have you do things for me. You need to see that I'm able to do things and not have to be waiting on you.

**Interviewer: So it's like those individuals, they mean well, by trying to, like you said, be on top of you.**

**Person:** Exactly!

**Interviewer: But that's based on the assumption that they don't think that you can do things on your own. I, okay, but then you want to be able to show them-**

**Person:** Exactly!

**Interviewer: So when, if you get the chance to show them, do they ever change their behavior?**

**Person:** Yes.

**Interviewer: Okay, and then, they give you more freedom to the... Yes, okay, okay do you ever... Is that always just showing them by doing or do you ever have conversations with people to have a conversation, to me? Tell me about that.**

**Person:** Yes, I sit with them, I explained them they can't be on top of me 24/7. I need to do my own things, and I need to be able to move around. Uhm, They are able to help me with certain things, like if I have to watch my clothes or make my food in the stove or in the oven and if I need help moving around, I don't if I don't have... God says that permits that I don't have STS uhm on day and I have to go somewhere they take me but then they end up saying no, because we need the car person, for other things or we need to take someone else somewhere, and all the promotions. So the one that stays behind and has to struggle is real.

**Interviewer: Mhmm. So you tell them when you need assistance**

**Person:** exactly

**Interviewer: How they can be helpful in a assistance. Okay, so tell me... So that's these nearly two years being where you are now. What about doctors and nurses before then they...**

**Person:** I've never lived in a home before. I've always lived with my mom.

**Interviewer: Okay, so, when I... When you live with your mom and you were still going to doctors and I and what not,**

**Person:** and I was taking my mom also on STS with me to her doctor appointments because she can’t drive she’s 49 years old, with epileptic seizures.

**Interviewer: Oh epileptic seizures, okay.**

**Person:** And she doesn't know what she gets them because she doesn't feel it so she just faints and doesn't know anything, so I...

**Interviewer: And is that from birth, or did that happen on that?**

**Person:** Just started happening in 2011.

**Interviewer: So let's talk about your appointments when you bring your mom with you and I am a... How is that often the case or your mom a company or come with you?**

**Person:** Yeah, okay, but I have some of the doctors' appointments, I go by myself because I know the way around and I know the doctors, but it is like a new doctor that I never seen that when I bring my mom with me, but most of the time I go by myself because I know them already, right? But all the time that my mom has to what her appointments I have to go with her on because she has (inaudible) and she doesn't know what's going on when she has the seizures. So I have to keep an eye on her so that she doesn't have a seizures. Because the drivers don't know nothing about the sickness of people.

**Interviewer: So when the first time you're meeting a new doctor and your mom comes with you, do you feel that how you're treated is different because she's there with you or is it the same yet?**

**Person:** Yes, not the same is different.

**Interviewer: Okay, tell me about that.**

**Person:** The way that I see it is that if I'm the patient, you as being my mom, yes, okay, you couldn't speak for me at a certain time, but don't answer everything all at once because I'm the patient, I'm the one that needs to be answering. Now if there's something that I don’t understand okay. That's where you come in, a new answer, and you speak for it, but not all day long, because that's how I need to show people that I am capable of talking and moving around, with things, and I don't have to depend on others to go anywhere with me, being with 24/7 like a caretaker or anything because I go out a lot and I do things all on my own.

**Interviewer: So is your mom answering a lot of questions because they're asking her instead of you?**

**Person:** At some point. Yes, and at some points no. Because if they ask me questions that I've never heard of or I don't have the answer to that's where my mom answers for me, because I don't want the right answer.

**Interviewer: Okay, but then they normally talk directly to you, or...**

**Person:** Yes.

**Interviewer: Okay, so it's more just when she... And search questions she might give too much over so she needs to also not get in the way of you, answer for yourself. Okay, okay, yeah, tell me anything that we haven't already talked about in terms of what other things might be making your experiences with doctors or nurses of the healthcare setting in general, not that ideal or not the best experience.**

**Person**: So I just feel like I eat. Yeah, you did what you have to do. But at some point to me I see it another way, because it's like you don't know what you're doing, you're looking at a patient, you don't know what to do with that patient, you don't know where to put that patient or how to treat that patient, so there's no right or wrong there. That's how I see it,

**Interviewer: So what more do they need in order to treat you?**

**Person:** Right, they need to learn more about my case, or be more on top, and responsible on my case of knowing was wrong with me or not knowing in other case. And then figure it out if you don't know what's wrong, then you figure it out by running a lot of tips and that's how you figure out what's wrong. That's how I see it, right?

**Interviewer: And before the running of tests, is there anything else you would recommend that... How should they know more about you specifically?**

**Person:** Going over my records, speaking to me more and I the way I'm gonna say it. But they need to go back to school and he and get a better education in doctors or nurses, whatever. Study more about the sick people.

**Interviewer: So it seems like we don't get the right enough training regarding spina bifida... When they're in school, exactly, okay so if they don't get it then they should get them or they say, the training, but they should also, talk to you on exactly to ask you questions.**

**Person:** Exactly. that's how I see it because I was told one day that I was gonna die at three years old, and I did it so they said Okay. Well, actually has 10-11 years to me and that was when I was five and I was back. And here I am 31. why are you telling me all this mumbo-drama of me dying?

**Interviewer: So you were young at the time, I think obviously you were young at the time, so maybe I've been talking to your mom or whoever I was with you. Is that because they didn't know enough or all?**

**Person:** This is from the doctor.

**Interviewer: But the doctor's not know enough and that's why they said you only have two more youth's.**

**Person:** That’s how I see, so I don't know. And I was told that they collapsed one of my lungs, so now I have from birth that I was just out the 31 years that I have longer connection because people that are born with spina bifida have a lung infections and I never knew that until now.

**Interviewer: And so, now you said that doctors and nurses they don't seem to have enough information and they don't always ask you everything. So do you do anything on your end to educate them, even if they don't ask you questions about or tell them about it.**

**Person:** So then most of what I know from when I was little, but it's like a repeating myself over and over. It's like if you have my record already with you, you should know what I have, the sickness. I have the other health issues that I have or that I'm getting. I should not have to be telling you about this when you have everything there,

**Interviewer: so but you make sure that they get your records so they should technically have everything they are already re-exactly, but they still don't seem to know.**

**Person:** Exactly.

**Interviewer: so it's not like they have four questions because they're confirming a nation, they actually don't know the...**

**Person:** Now if it's in the next case is if there's something that's not on there or something that's new that's happening to me, that's when I would go up to you and I will tell you, so that you put it in the record. So, next visit I already have her in the record and you don't have to tell me for my other visits.

Interviewer: So you said that you go to the doctor often so the ones that you go to by yourself, because you've been there enough, do they get better as far as now, they know more or to about that?

**Person:** A that's (hospital) I only have two doctors there, one is the (inaudible) and one is for my bladder and I've been with them, since birth, and they pretty much know what's wrong just by looking at me So I don't have no problems with those two doctors.

**Interviewer: Did they start out not knowing or they seem to be better from the start?**

**Person:** They’ve been perfect since birth.

**Interviewer: Okay, so do you think that they had training abounds in a bifida, and other conditions, more so than other people or what's different about them?**

**Person:** It's like they know more and they have a lot of experience with that are within of spina bifida and bladder problems, but then the others who say, but in reality they don’t. it’s like they don’t know what to do or what to say or how to move. And that’s how I see it.

**Interviewer: Great, and when you, you say that you have to tell them the same information a lot of the time. So when you tell them, do you feel like they're hearing and listening to you?**

**Person:** Yes, at some point but then again, why do we have to keep repeating myself to you every time I come here? If you already have that in the record. Just go through thte papers or the computers and you’ll see all the information. Now, if there's something new in me, that I have other health issues that are... Now that's when I will go up to you and I will tell you.

**Interviewer: So they... Do you say they do listen in and hear you? So do you think that they respect the information you're sharing with them?**

**Person:** At some point, yes, and at some point known because they're asking personal questions, to me that they should not be asking. Because it's let one day I had someone come to my house from HRS and she was asking me questions. And one of the questions she was asking me is, have I ever had a baby before? And how would I take care of that baby with the condition that I'm in and I... How would I support that baby? And it's not all about supporting the baby. Okay, it's about the condition you're in number one and the condition the baby is because not only people that are sick, like me have babies but God permits that their baby comes out with the same problem. How do you help that baby... You spina bifida a bio for example, and you're able to move a little bit. Yeah, you could take care of the baby, and you could do things with the Bailey but you gotta see it and realize it for yourself, because... But you were just asking me that You're not gonna know the truth, you have to see it with your own eyes, to realize what you are able to do with that baby and when you're not able to do. I have taken care of, of almost a whole world of babies because I have a guy daughter, and I have my little sister who's 29 years old that has three kids, I see, and so at every three birth that she's had, I've been with all three of her kids, since birth, and it's a thing that I've always wanted since a middle school that I've always wanted to be with little kids or with babies, because that was my dream and I never got to that dream because of all my health issues but that is one of my dreams that I've been wanting. So they can see once they start growing up why I'm in the chair. And they can learn so that when they're older, they know what to do and how to move with others and socialize with others that are sick. So that’s how I am.

**Interviewer: How you think that that might be as far as, we're talking about needing training that might be part of the training is interacting with people that have different types of disability yet that you learned by okay, interacting with it?**

**Person:** Yes.

**Interviewer: okay, tell me another thing. One thing that pops up with people when we have these conversations. With equipment and access things like that. Do you have any challenges when you go to the doctor about not being able to do certain tests, or get certain services because of lack of access?**

**Person:** No they do everything because I give them the right to do anything.

**Interviewer: Okay, for example, some folks have said, in the past that because I'm in a wheelchair, they don't have any way to weigh me. Is that one of your experience, or to a...**

**Person:** At some point, yes, because I can't get up but they use a ramp, a little ramp to put over the scale to put me on, and that's how they weigh me.

**Interviewer: So they have a way to... To still.. support you. So nothing else that comes to mind whether it be a table that’s too high or no assistance so there’s, there’s assistance but transferring all the stuff is okay?**

**Person:** I do transferring in and out of bed by myself but with very little help to like push me back. I get help by my pants by the back. To pull me back. But all the strength is me. I don’t let them do the strength I just let them grab me by the pants and help me to pull back. But everything else I do on my own.

**Interviewer: Okay, and tell me because we talked about before you know people having these preconceived ideas about what you can or can’t do. Uhm, do you, does the doctor/nurse ever make any assumptions about what you’re able or not able to do?**

**Person:** No.

**Interviewer: Okay so they usually just ask you instead of just assuming?**

**Person:** Yes.

**Interviewer: Okay. In terms of can you transfer can you get on the table?**

**Person:** Yes.

**Interviewer: Okay, that sounds like a positive then. Okay, okay to see where we are here. So one of the things that we're looking to do with getting this information learning from you is how to make the healthcare experience is better for people that have developmental disabilities, because we know from the research that it's usually that they get less or quality or you care or less satisfied with the care that they receive is that... Would you agree that that's your experience or no?**

**Person:** Yes.

**Interviewer: I think part of that is the question of how exactly do we do that, how do we make it better? And one of the suggestions is that we have to first know who has a disability, and who doesn't? So that would involve asking Do you have a developmental disability? So my question to you is, are you comfortable with someone asking you a question like that?**

**Person:** Yes.

**Interviewer: Okay, and then, how should they ask the question? Should it be as simple as that? Do you have a developmental disability or should it be asked in a different way?**

**Person:** No that’s perfectly fine.

**Interviewer: Okay, and when you prefer it to be on a form that you fill out, like a patient form?**

**Person**: Don’t matter if it’s on paper or if you say it with your own words.

**Interviewer: Okay, and the thought behind asking that question is to be able to better inform the doctors and the nurses that they know how to provide care. So if I ask you, "Do you have a developmental disability? And you said yes, would I have enough information to give you better care, or is there something else I should be asking you?**

**Person**: Just by you knowing that I have a learning disability should tell you at what level of certain things I am on or that I can do .

Interviewer: Okay so tell me more about that. If I ask you do you

**Person:** If I read you for example, that paper, I will read you have of that paper because I know most of the words, but they are words if it's a long or I would have to look at the word. And sound out each the letter of the word to be able to get the work correctly.

**Interviewer: Okay, so right, so do you think that they would know that by just asking you if you have a disability or would you have to tell them more specifically that information?**

**Person:** Some people, yes. And some people no.

**Interviewer: Okay, okay, so then, okay, so that gets us to the next point. So I have a paper here that has a few questions on it. This is not meant for the healthcare, world, it's actually from the US Census questions that are asked to determine if someone has a disability, cost. So this is kind of a starting point to see what do you think about these questions? So, I'll read them to you on... So you, you deaf you have serious difficulty hearing?**

**Person:** No.

**Interviewer: Are you blind or do you have serious difficulty seeing even when wearing in glasses?**

**Person:** Uh, I wear glasses, to see from afar.

**Interviewer: But those are fine when you wear them, you can see fine.**

**Person:** Yes.

**Interviewer: Because of a physical, mental, or emotional condition. Do you have serious difficulty concentrating, just remembering or making decisions?**

**Peron:** Yes.

**Interviewer: So, okay, do you have serious difficulty walking or climbing stairs yes, do you have difficulty dressing or bathing?**

**Person:** Yes.

**Interviewer: Okay, because of a physical, mental, or emotional condition. Do you have difficulty doing errands' alone such as visiting the doctor's office, or shopping?**

**Person:** Yes and no, okay. Sometimes, yes, if it's to do grocery shop, yes, that's when I would need to help. Or if its to do my regular shopping is at the more or something I can do that on my own.

**Interviewer: So those are the six questions. Do you do like those questions?**

**Person:** Yes.

**Interviewer: Is there anything you don't like about those questions?**

**Person:** No.

**Interviewer: And do you feel like they give more information than just asking? Do you have a disability that more information that would be helpful to them giving you better care?**

**Person:** Uh... They should know, just by knowing hearing disability and a lot of health issues. Yes, at some point you need help or you don’t need help. or yes, you don't need to help all day long, if you have Down Syndrome or in (Inaudible) that the mines and on the world that I don't know what you guys call that, but yes, I've seen cases like that.

Like you have to be with that person 24-70 to take care of that person.

**Interviewer: Right, right, so if they learn that you have a learning disability, for example, what should they do differently for you, that they wouldn't do for someone that didn't have a learning disability?**

**Person:** Try not to help so much so that you can see with your own eyes that I am able to do the stuff on my own and just go on to the next person who you know you think that may need the help better or more than what I do.

**Interviewer: So they should kind of learn by observing whether or not you need help.**

**Person:** Exactly.

**Interviewer: And if they don't or they could just ask you?**

**Person:** Exactly.

**Interviewer: Okay, so what help? So, for example, you said that when you pointed at my paper, and you said if you were to read that you would read only half of it, so is that something that I should just let you read it or should I ask? Can you read it, what would be the best way too handle that?**

**Person:** However, you are either way, is fine either way.

**Interviewer: Okay, okay, But I should maybe ask you questions to see that you understand. So for example, if I am your doctor, and I want you to do something, whether it's take medication or do something else for your health should I ask you a follow-up question to make sure that you understand what to do?**

**Person:** Yes.

**Interviewer: okay, Do you to... Your doctors typically do that?**

**Person:** Yes.

**Interviewer: Okay, do they always do that?**

**Person:** Yes.

**Interviewer: Okay, awesome, look at it. And in terms of a spina bifida ... What specifically would you want them to like what would you tell them that they should know or do to make your experience better?**

**Person:** Learn more about it. Or have more experience in knowing what to do with a spina bifida person or with a baby that you already know that is about to be born with spina bifida.

**Interviewer: Okay so do some research. Let me back up before we even go to a new doctor. Do you do anything ahead of time to let them know that you have a developmental disability or do you tell them when you get there?**

**Person:** sometimes yes, sometimes no, because they already have my record.

**Interviewer: Okay, so if they have your record, you don't bother to see as you expect that they already know whether they do or not, or whether they read it or not.**

**Person:** Mhmm.

**Interviewer: Okay, but when you don't think that they have your records?**

**Person:** That’s when they come up and say..

**Interviewer: Okay, you make a point to say-**

**Person:** Yes.

**Interviewer: Do you think that you're treated differently when they realize you have spina bifida?**

**Person:** No.

**Interviewer: No? Okay. So everything else is as it would be for anyone?**

**Person:** yes.

**Interviewer: Okay, great, so I read these six questions and you said you were okay with them, is there anything that I should be asking that isn't there the thing else you would add when you think about other ways that doctor and nurse could help or support you as their patient?**

**Person:** and just to have more experience and care for sick people or people with spina bifida.

**Interviewer: So they should get more training and more exposure to a one, and that should probably hopefully be done earlier in-**

**Person:** Exactly,

**Interviewer: okay, okay, let's see here, what else do I have, and how often if you were asked these questions, again, would you like these, if these were beyond just the... Do you have a development of disability? Would you want these in writing, or verbally asked you?**

**Person:** However, and it's comfortable, I don't matter for me.

**Interviewer: Okay, but you wouldn't mind you, you like those questions?**

**Person:** That's fine.

**Interviewer: And how often would you wanna be asked just one time or so that it goes into a metro, medical record?**

**Person:** Exactly. one time and I, from there you put it on the record and just keep it there in-

**Interviewer: Okay. I think that that's all my questions. You wrote these notes down, for me, before you came here. Is there anything in here that we haven't talked about yet, that we wanna talk about that really we have was covered at all?**

**Person:** I just wrote them down to see like Who do you think?

**Interviewer: Okay, well, let me, I'll pause, I'll pause this for a good thing.**

**TAPE SKIPS TO THIS*****

**Person:** A walker and the chair, the Chair and the walker is just there to help you, but in your mind, you need to see that you're not on there. You're on your own. Don’t let the chair or the walker bother you. Because the more you ;et that bother you the more stressed or the more sicker you can get. So that the charter you don't frustrate about other people that don't wanna be around... You don't care about you or that are talking bad about you or a treat, you wrong. Because, there’s no right and theres no wrong. Everybody here is the same, no one here is perfect. And sooner or later that person that's not... And that's working and has a better life than what you do because of your health that person may God forbid, and the one date in your shoes and feel the pain that you're in because of the way they've been with you treating you. That's how I feel, and that's how I see.

**Interviewer: So you're trying but you're also trying to educate people exactly, so that they don't treat anyone differently.**

**Person:** Exactly.

**Interviewer: Cool. I think that’s, that’s, pretty-**
